# Supplementary figures and images for: Gpr54 deletion accelerates hair cycle and hair regeneration (part 1 of 2)
Source: EMBO Rep. 2024 Nov 25;26(1):200–17. doi: 10.1038/s44319-024-00327-y (PMC11724127; doi:10.1038/s44319-024-00327-y)

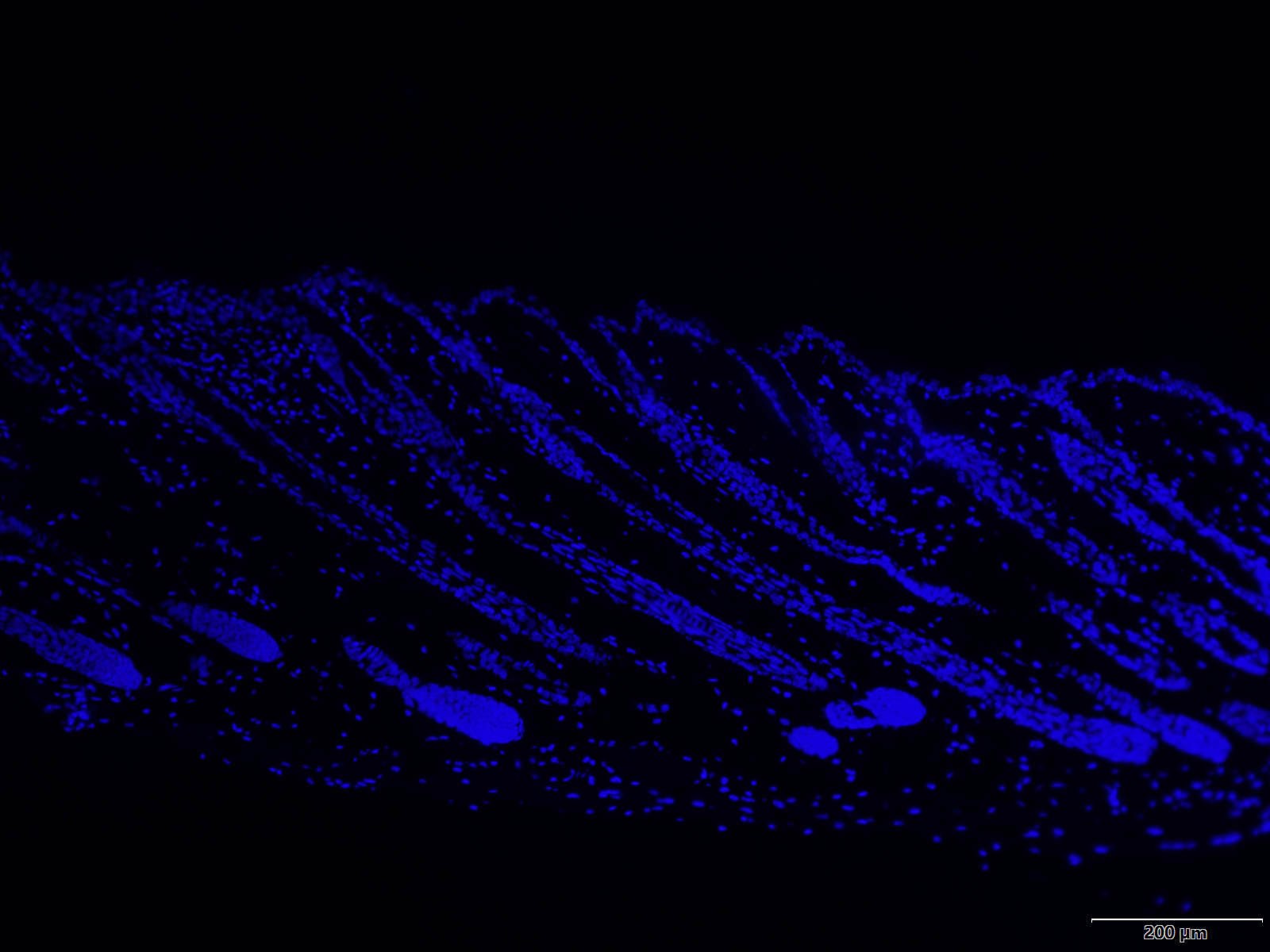

Supplement: Supplementary file 2 — Source data Fig. 1 [file 44319_2024_327_MOESM2_ESM.zip › Figure 1/1A/Anagen/1 (1).tif]

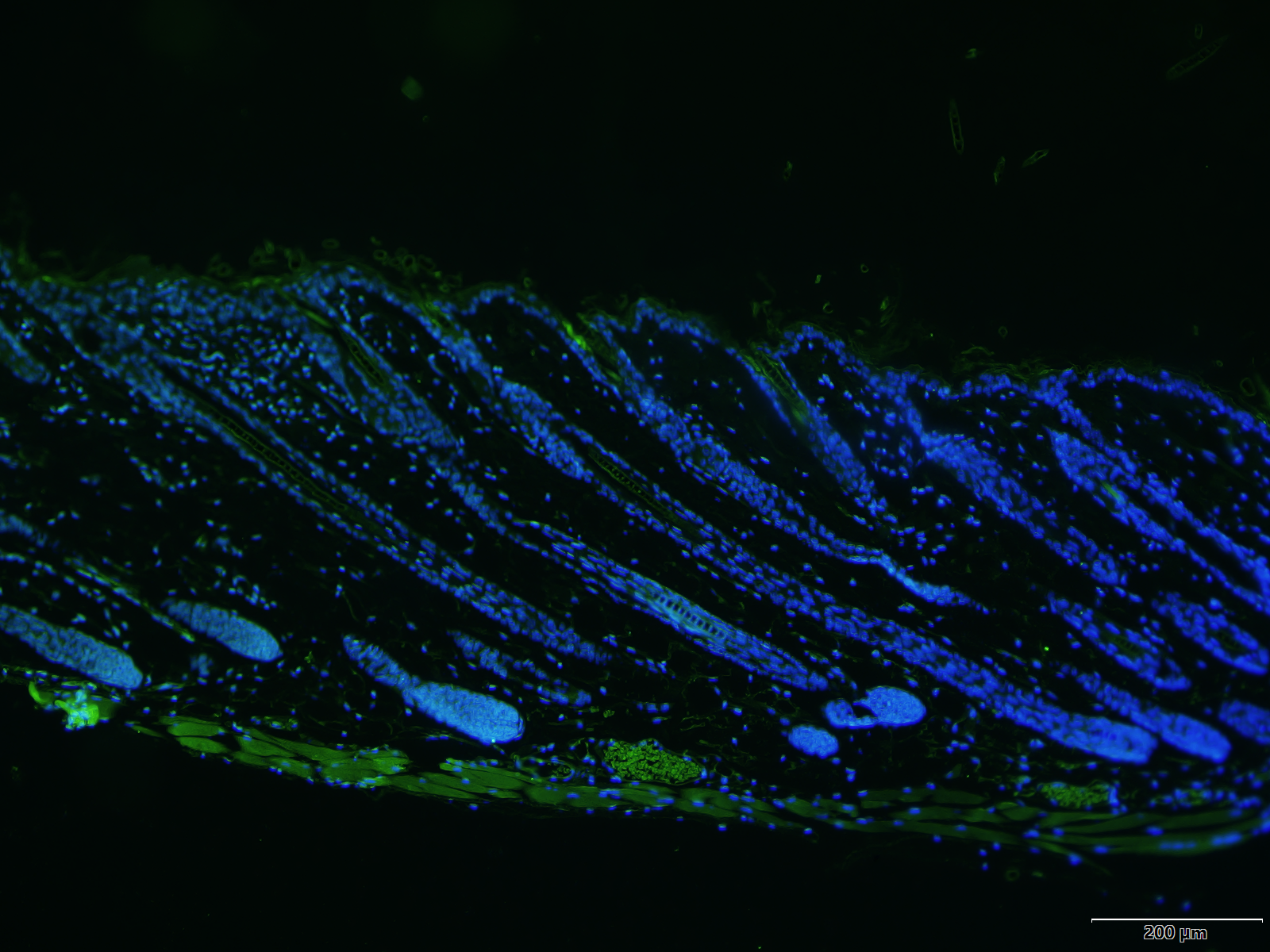

Supplement: Supplementary file 2 — Source data Fig. 1 [file 44319_2024_327_MOESM2_ESM.zip › Figure 1/1A/Anagen/1 (2).tif]

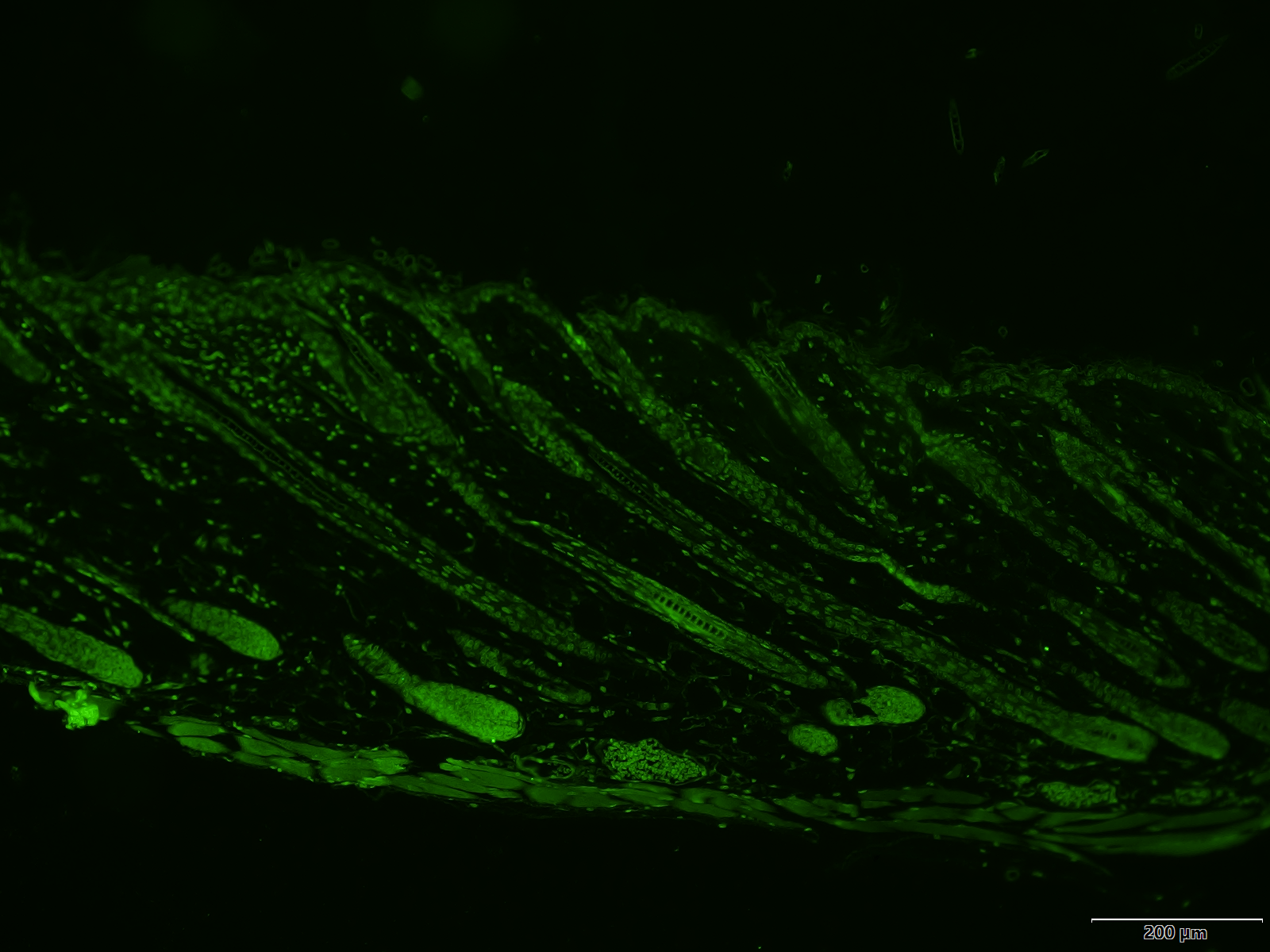

Supplement: Supplementary file 2 — Source data Fig. 1 [file 44319_2024_327_MOESM2_ESM.zip › Figure 1/1A/Anagen/1 (3).tif]

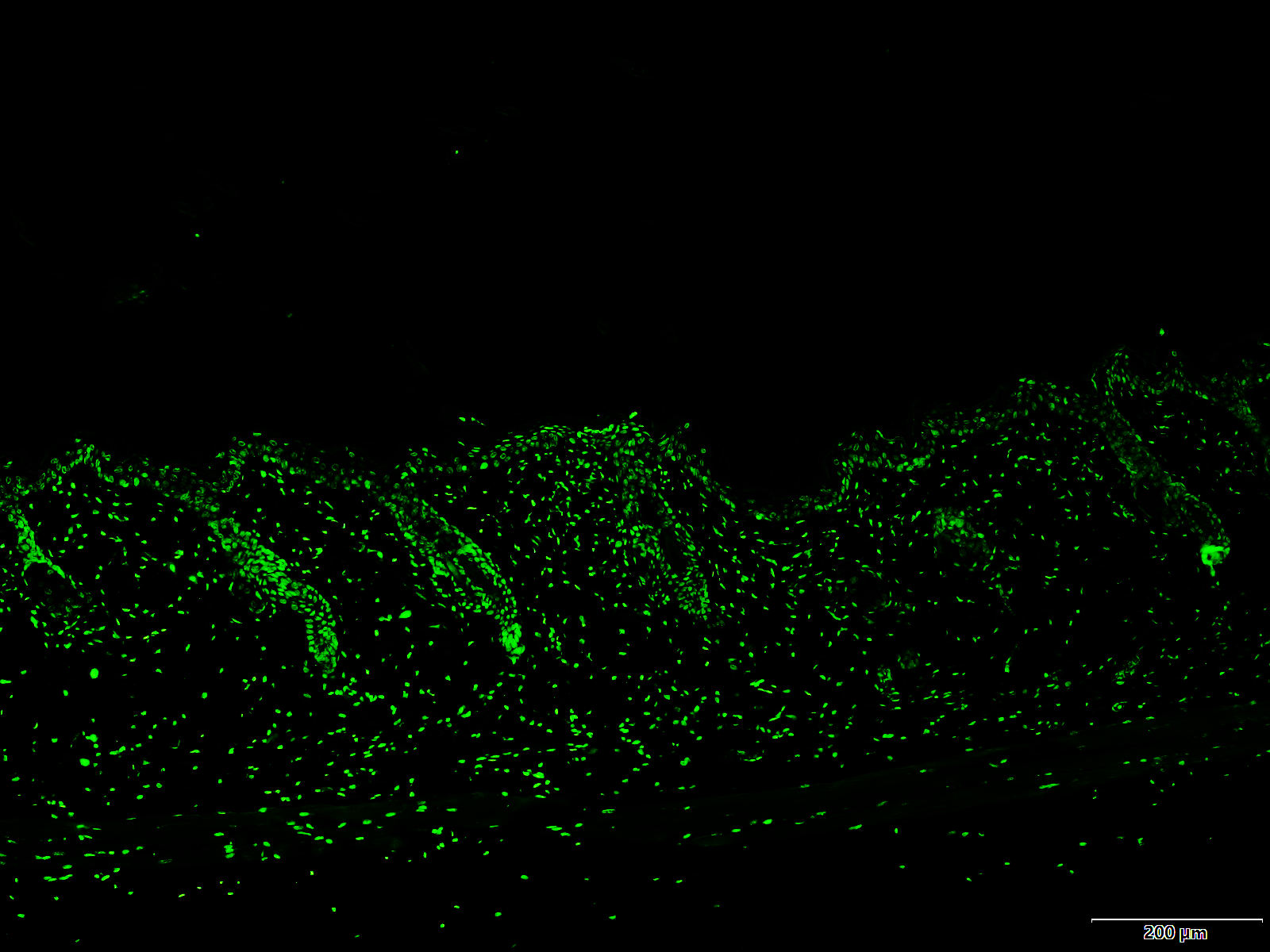

Supplement: Supplementary file 2 — Source data Fig. 1 [file 44319_2024_327_MOESM2_ESM.zip › Figure 1/1A/Catagen/1 (1).tif]

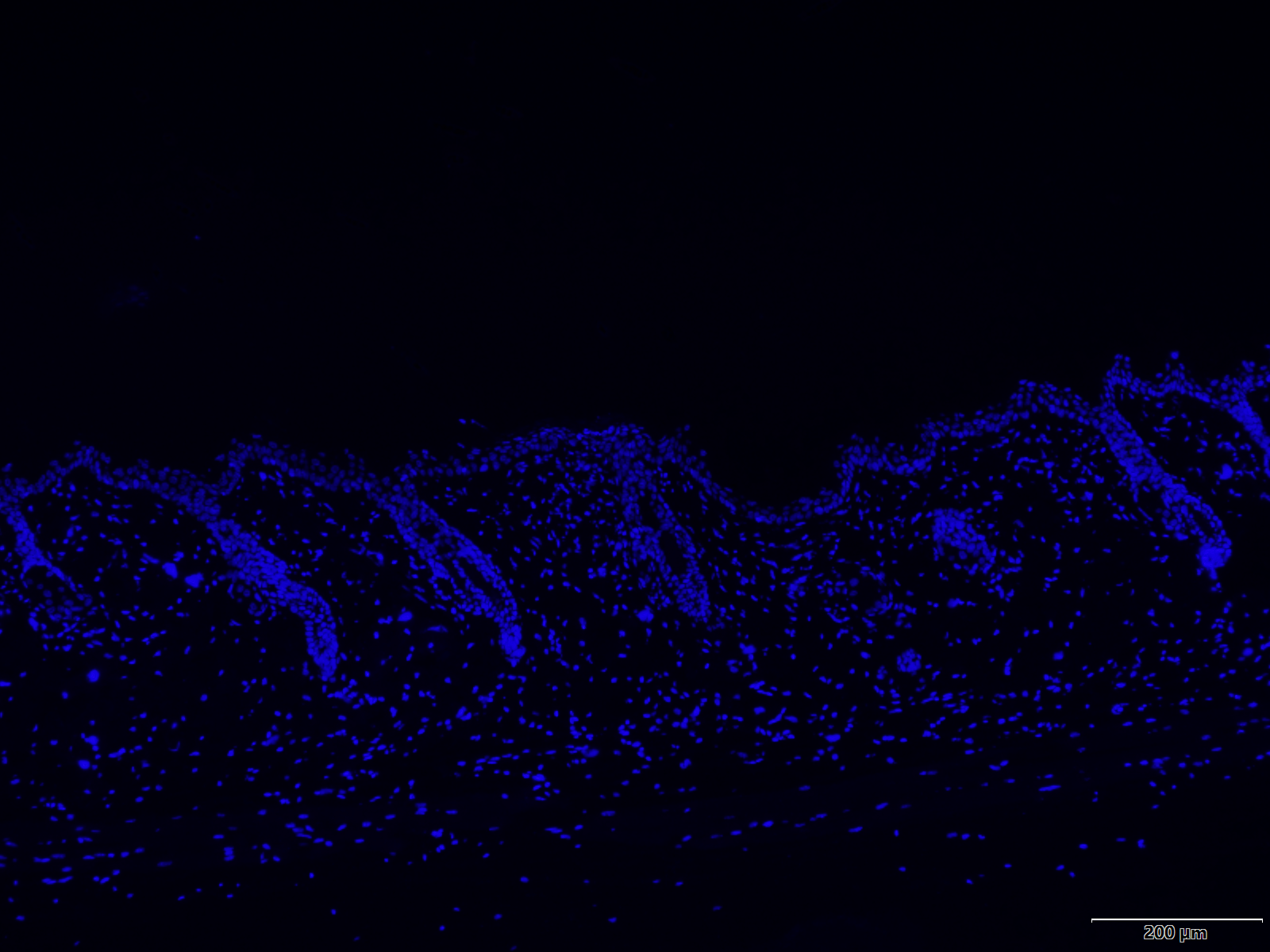

Supplement: Supplementary file 2 — Source data Fig. 1 [file 44319_2024_327_MOESM2_ESM.zip › Figure 1/1A/Catagen/1 (2).tif]

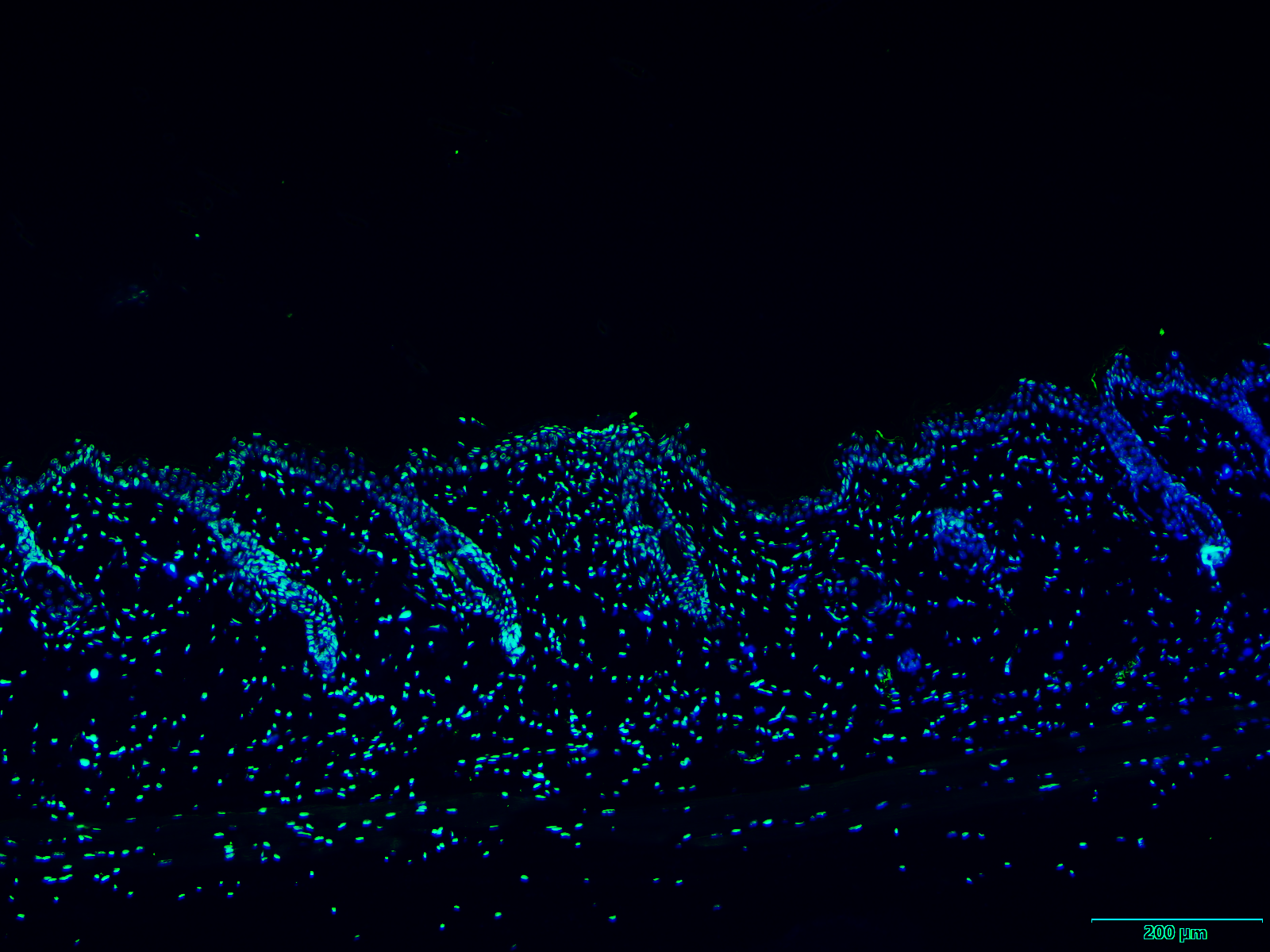

Supplement: Supplementary file 2 — Source data Fig. 1 [file 44319_2024_327_MOESM2_ESM.zip › Figure 1/1A/Catagen/1 (3).tif]

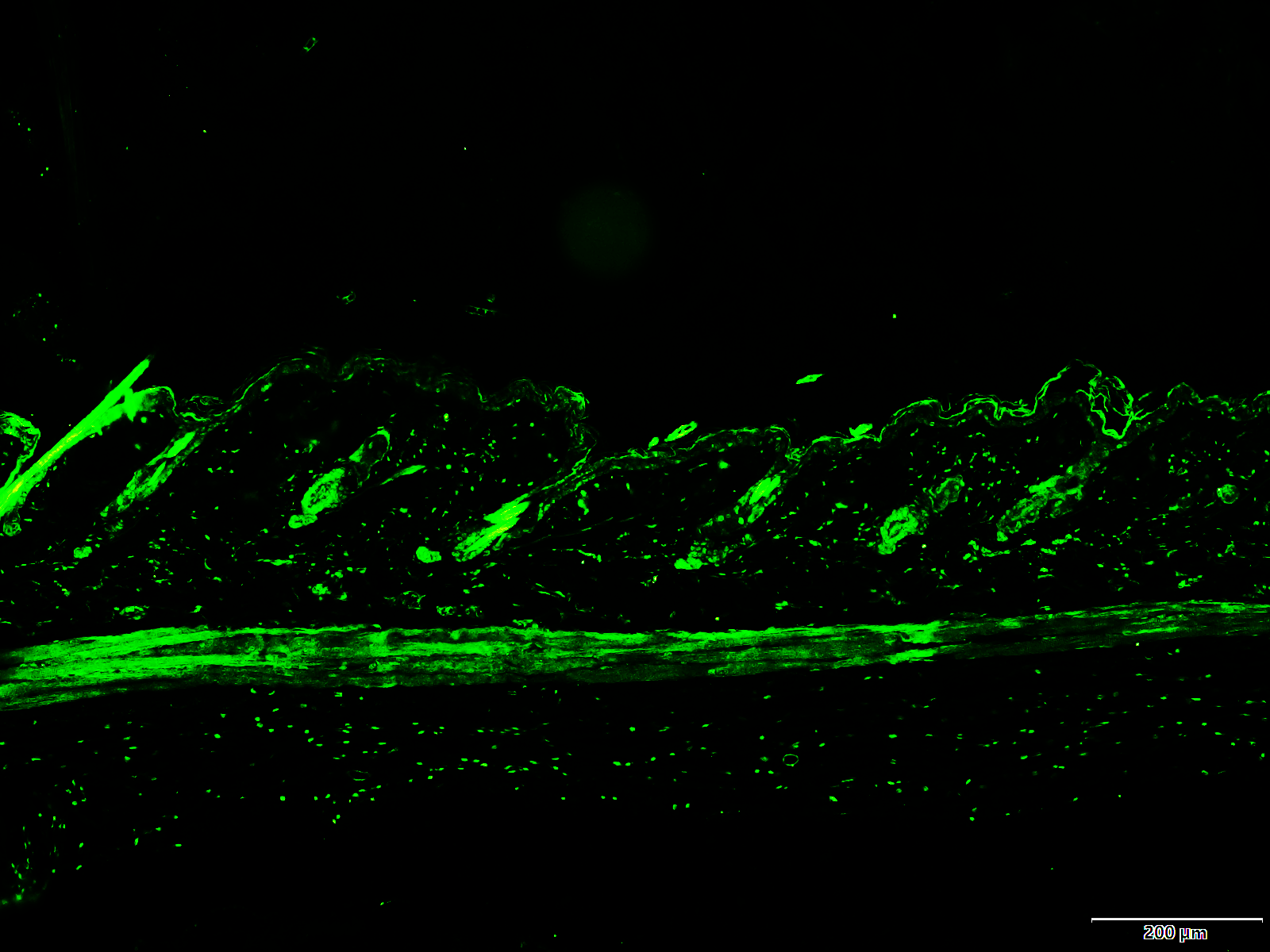

Supplement: Supplementary file 2 — Source data Fig. 1 [file 44319_2024_327_MOESM2_ESM.zip › Figure 1/1A/Telogen/1 (1).tif]

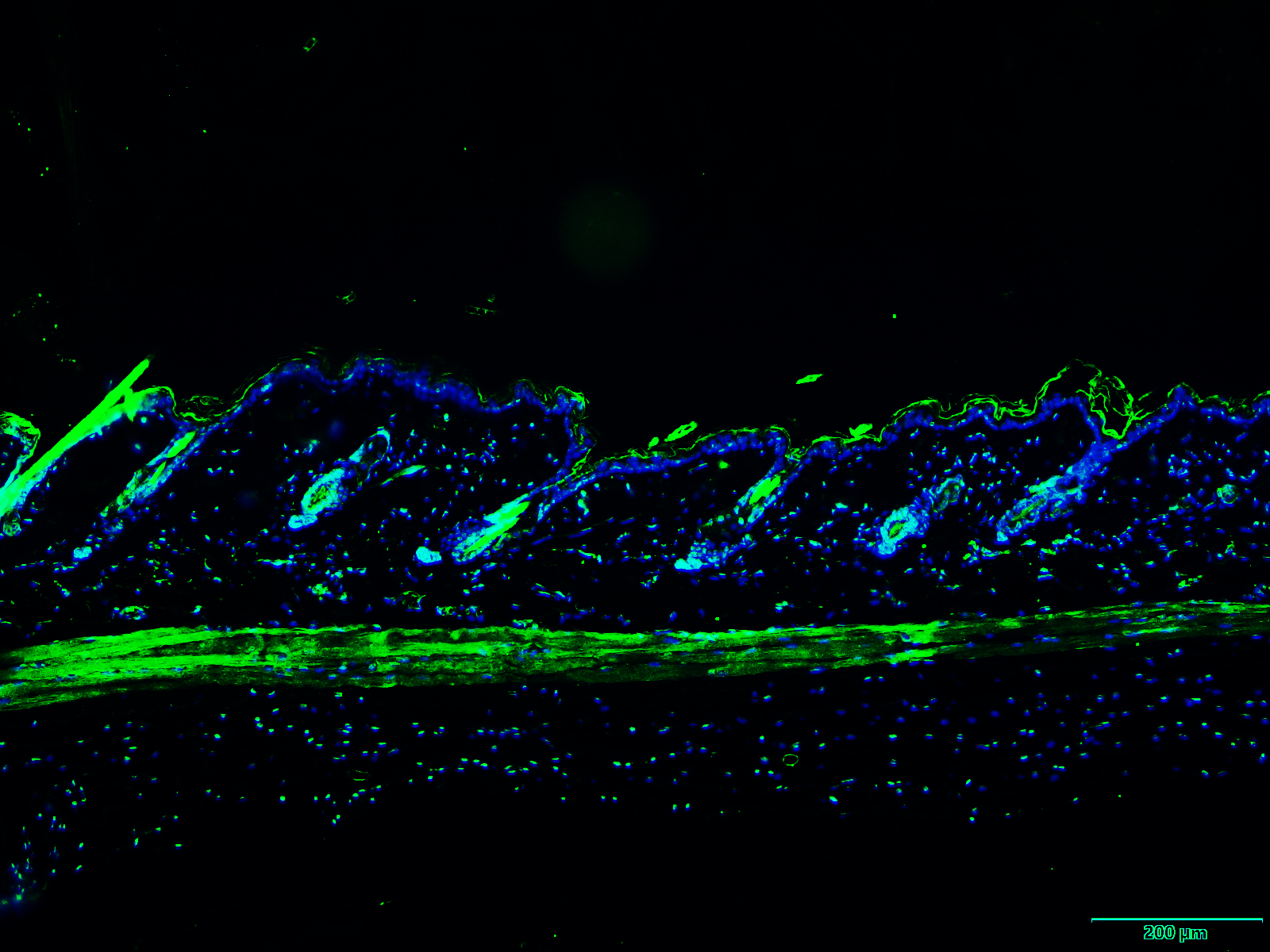

Supplement: Supplementary file 2 — Source data Fig. 1 [file 44319_2024_327_MOESM2_ESM.zip › Figure 1/1A/Telogen/1 (2).tif]

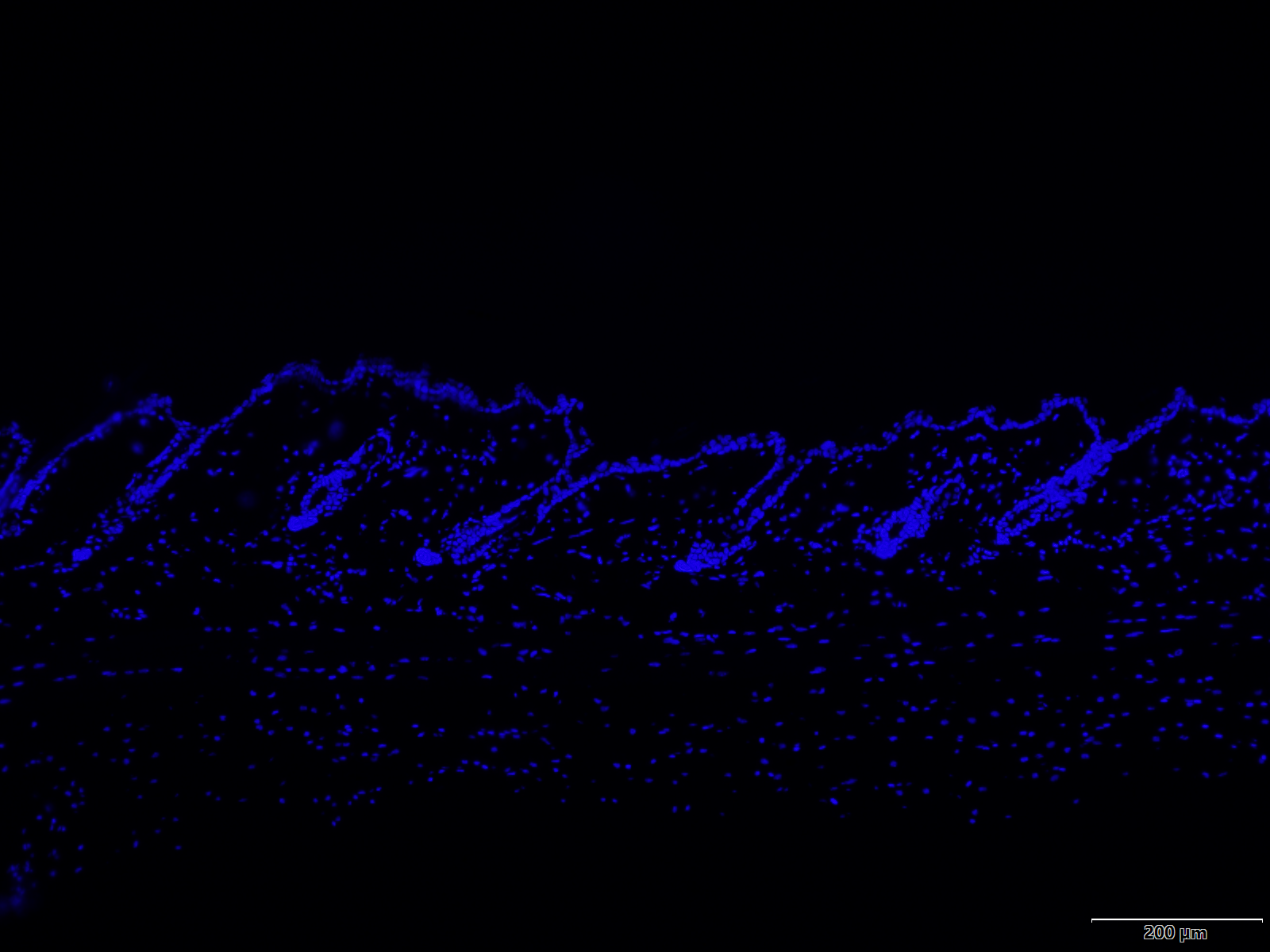

Supplement: Supplementary file 2 — Source data Fig. 1 [file 44319_2024_327_MOESM2_ESM.zip › Figure 1/1A/Telogen/1 (3).tif]

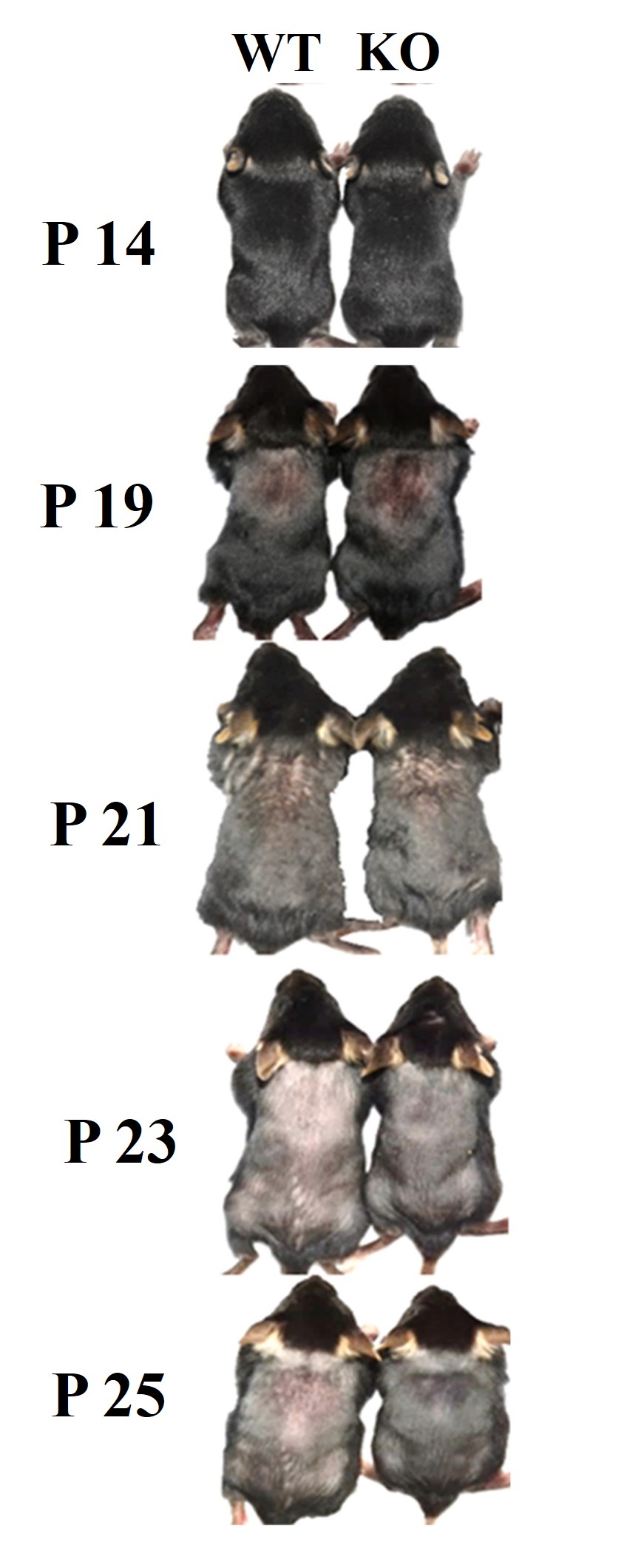

Supplement: Supplementary file 3 — Source data Fig. 2 [file 44319_2024_327_MOESM3_ESM.zip › Figure 2/2A/1.jpg]

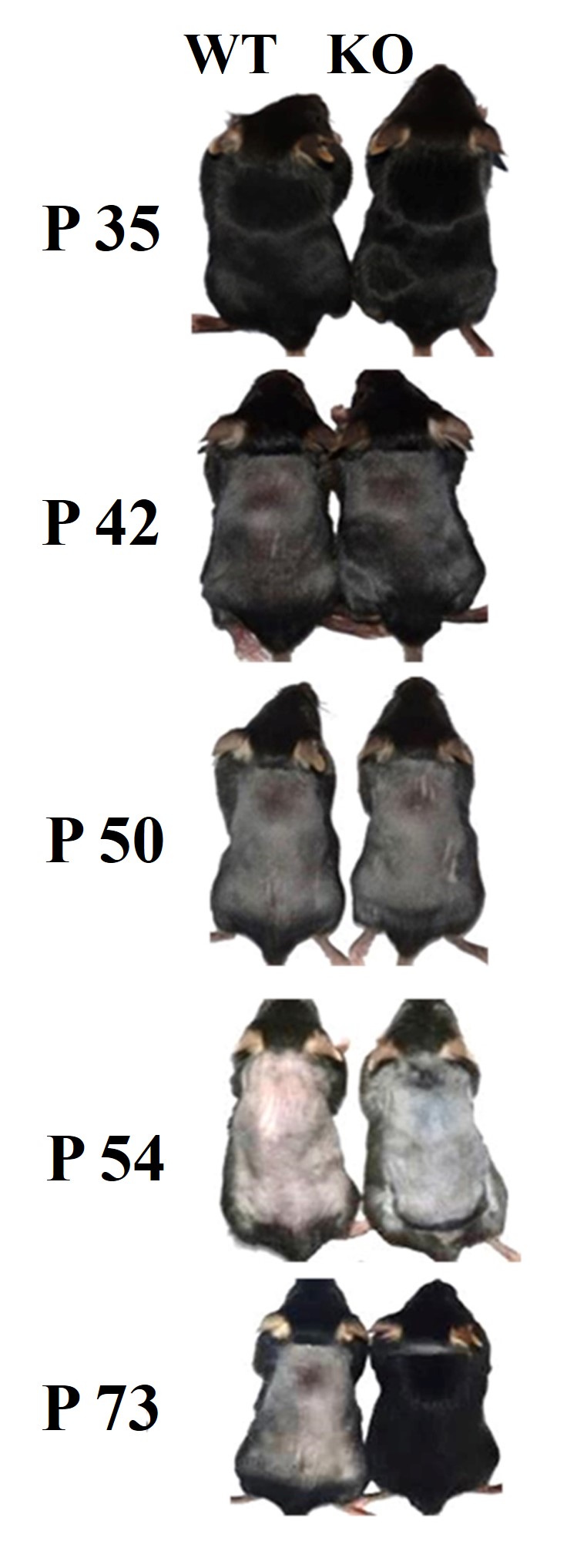

Supplement: Supplementary file 3 — Source data Fig. 2 [file 44319_2024_327_MOESM3_ESM.zip › Figure 2/2A/2.jpg]

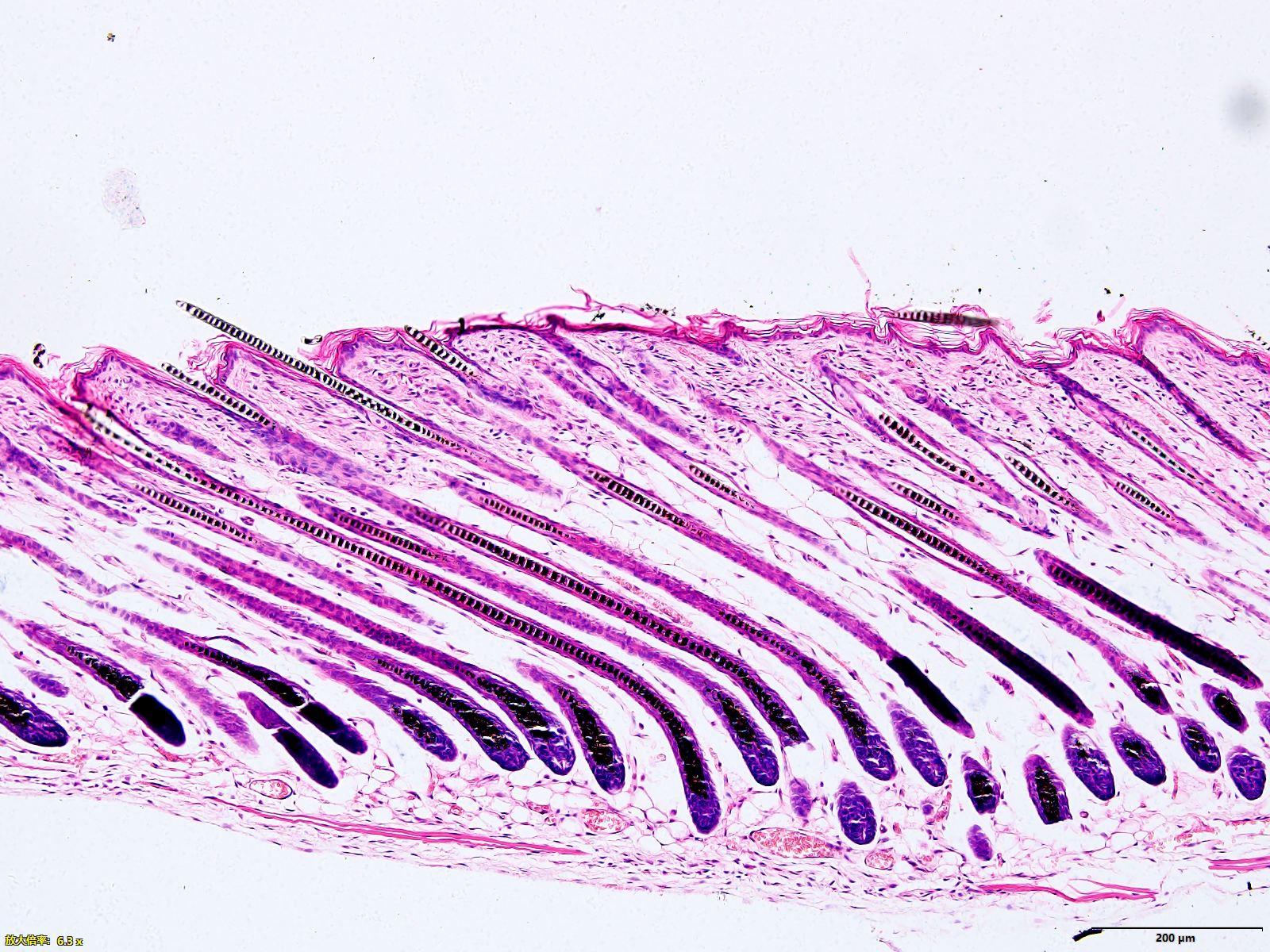

Supplement: Supplementary file 3 — Source data Fig. 2 [file 44319_2024_327_MOESM3_ESM.zip › Figure 2/2A/P14/KO/1.tif]

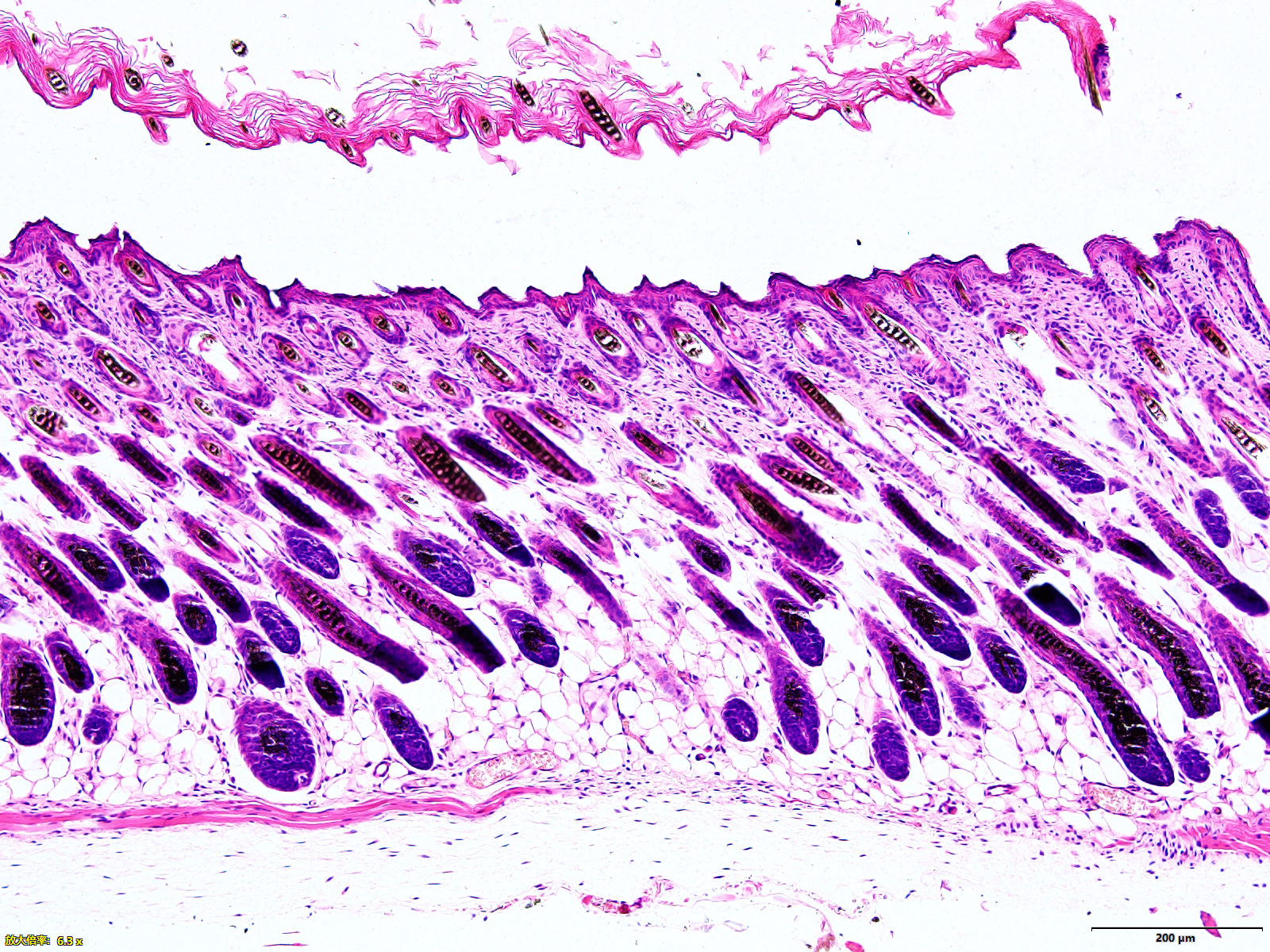

Supplement: Supplementary file 3 — Source data Fig. 2 [file 44319_2024_327_MOESM3_ESM.zip › Figure 2/2A/P14/WT/1.tif]

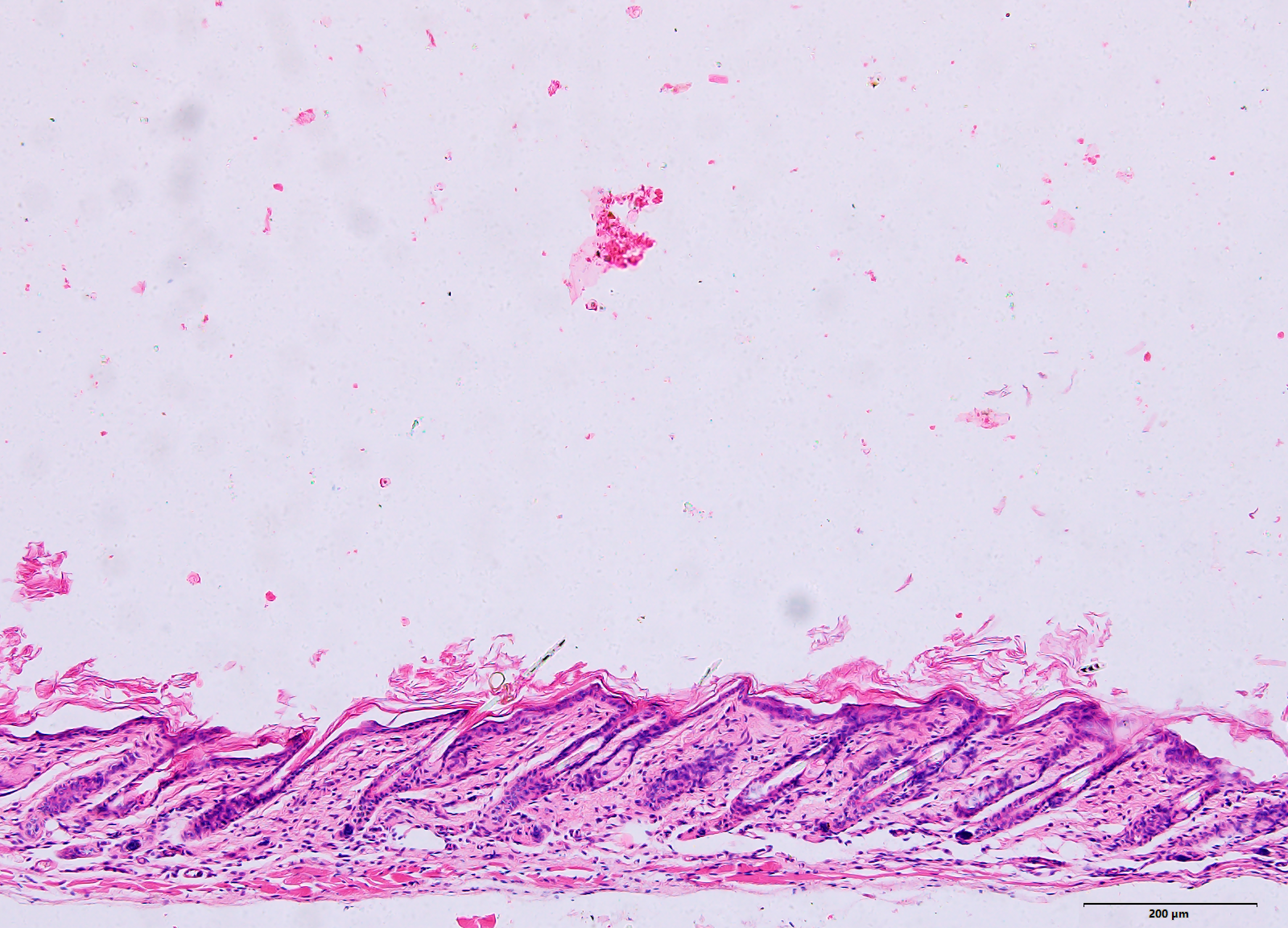

Supplement: Supplementary file 3 — Source data Fig. 2 [file 44319_2024_327_MOESM3_ESM.zip › Figure 2/2A/P19/KO/1.tif]

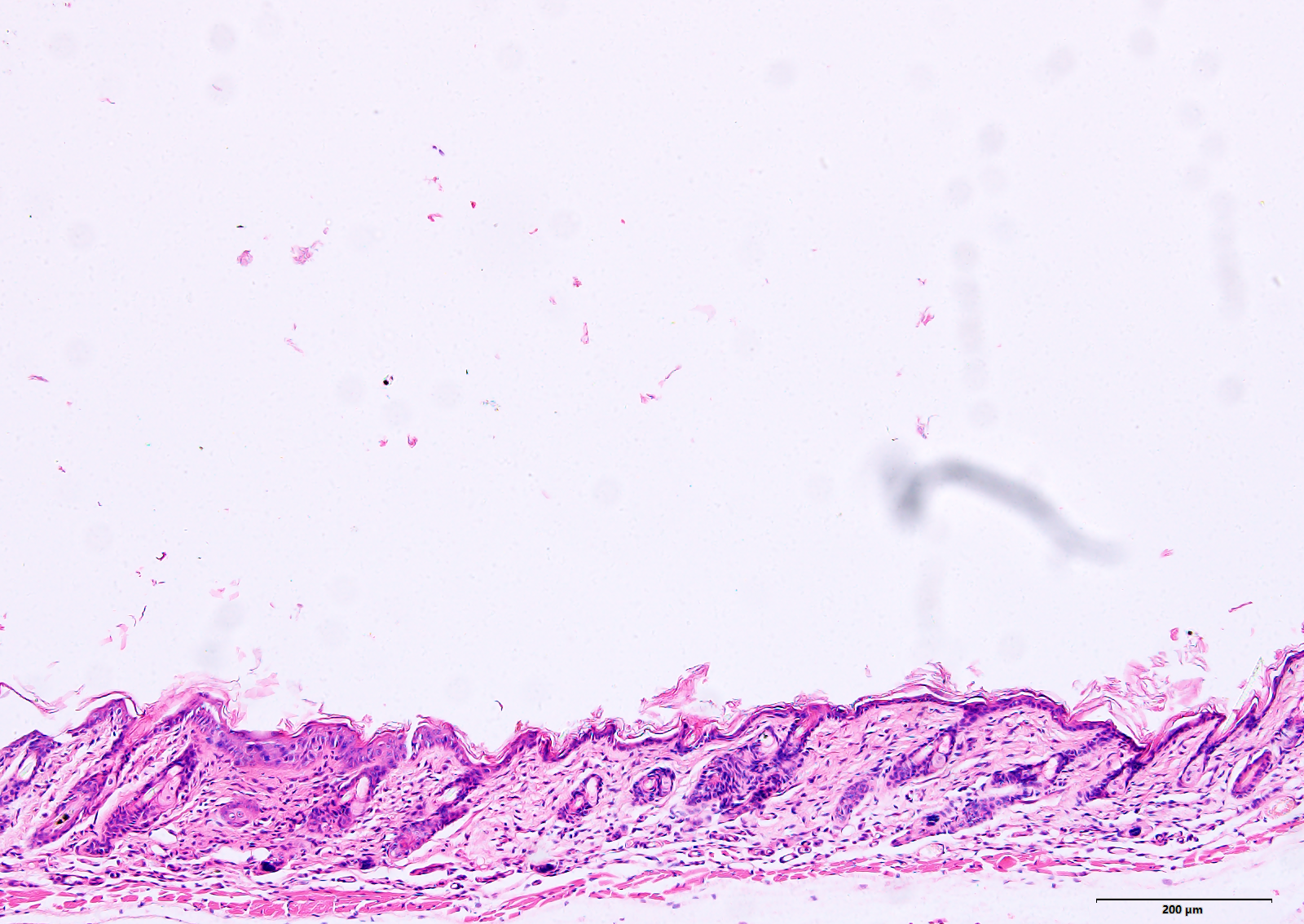

Supplement: Supplementary file 3 — Source data Fig. 2 [file 44319_2024_327_MOESM3_ESM.zip › Figure 2/2A/P19/WT/1.tif]

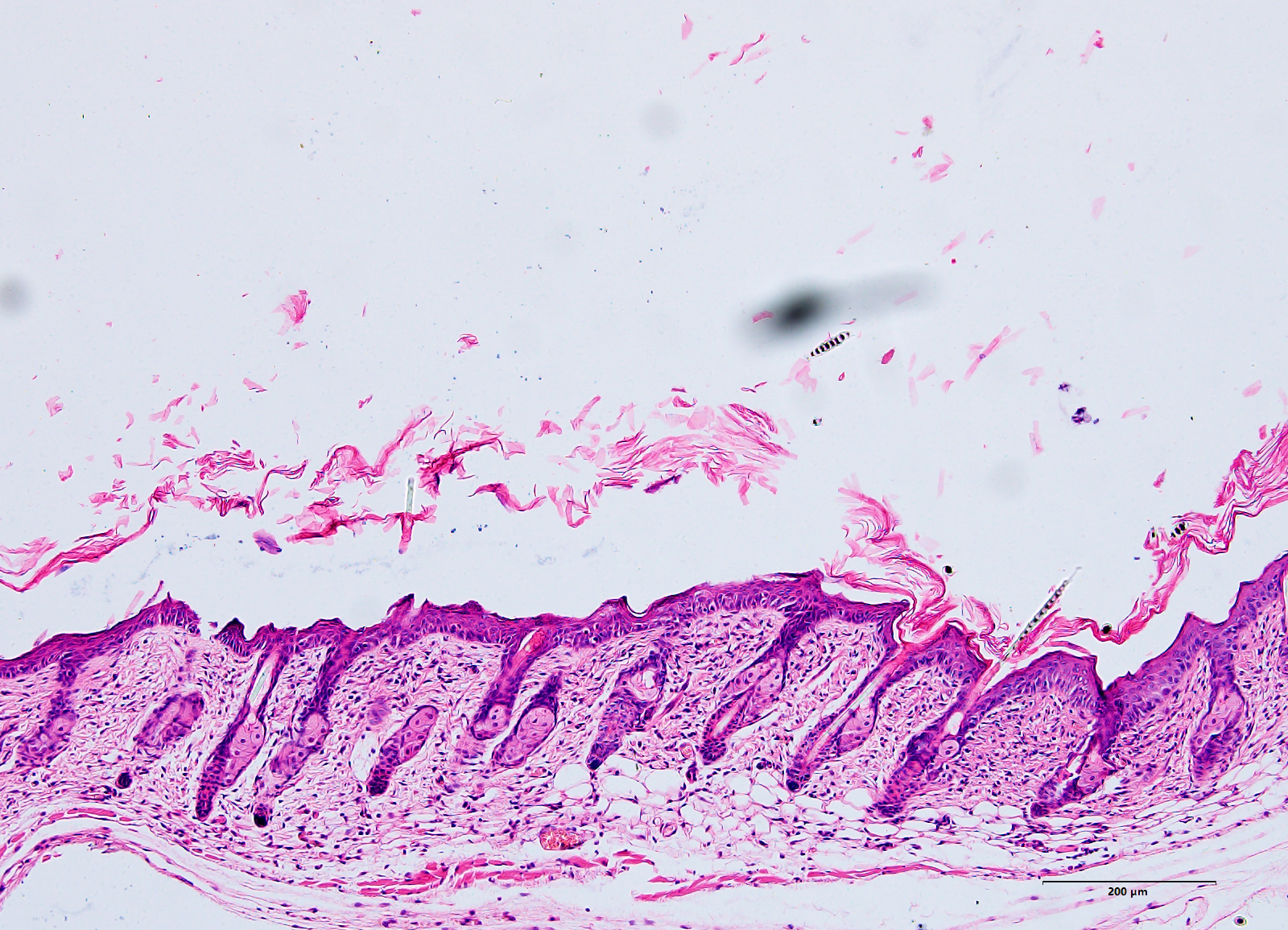

Supplement: Supplementary file 3 — Source data Fig. 2 [file 44319_2024_327_MOESM3_ESM.zip › Figure 2/2A/P21/KO/1.tif]

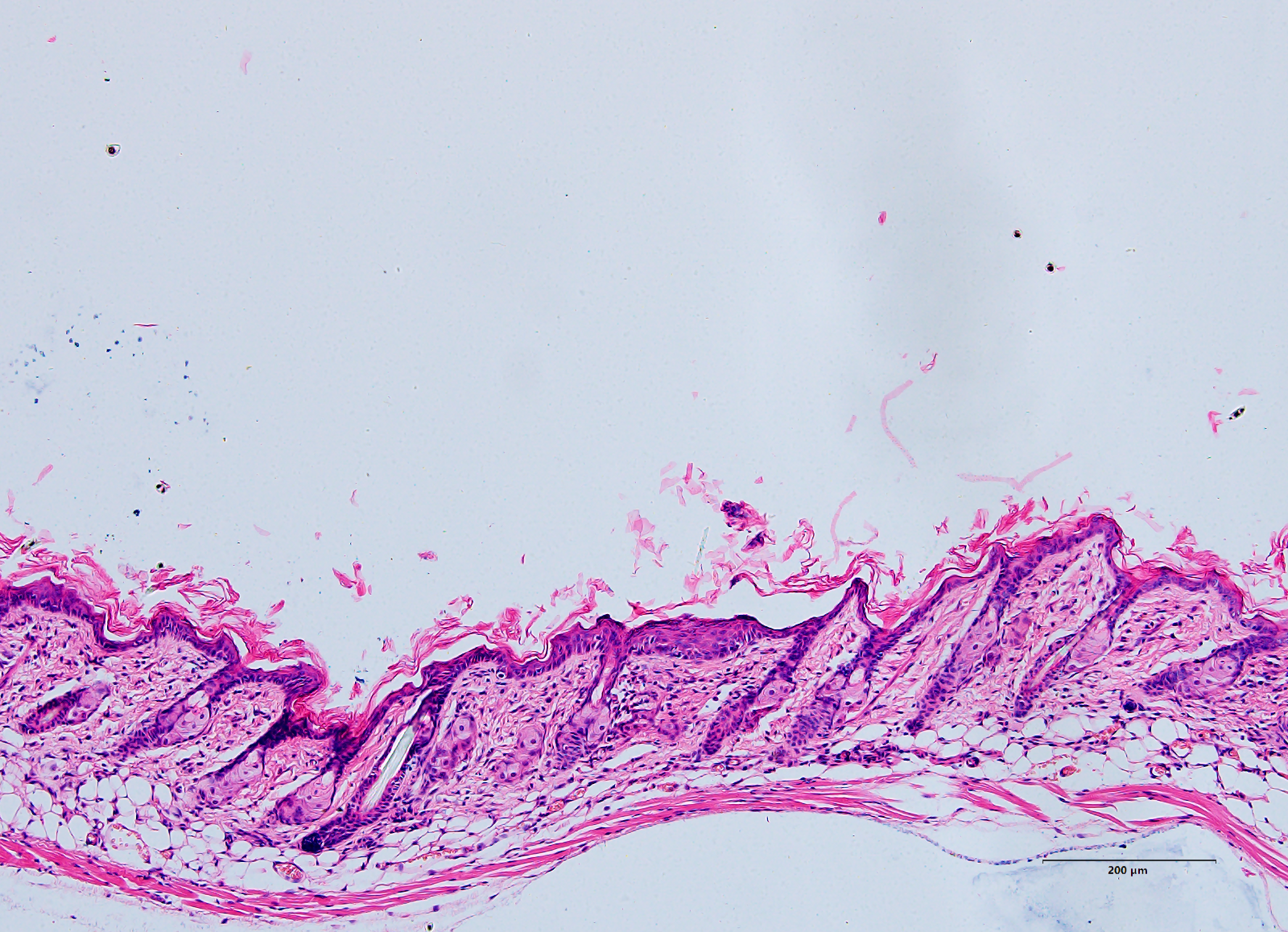

Supplement: Supplementary file 3 — Source data Fig. 2 [file 44319_2024_327_MOESM3_ESM.zip › Figure 2/2A/P21/WT/1.tif]

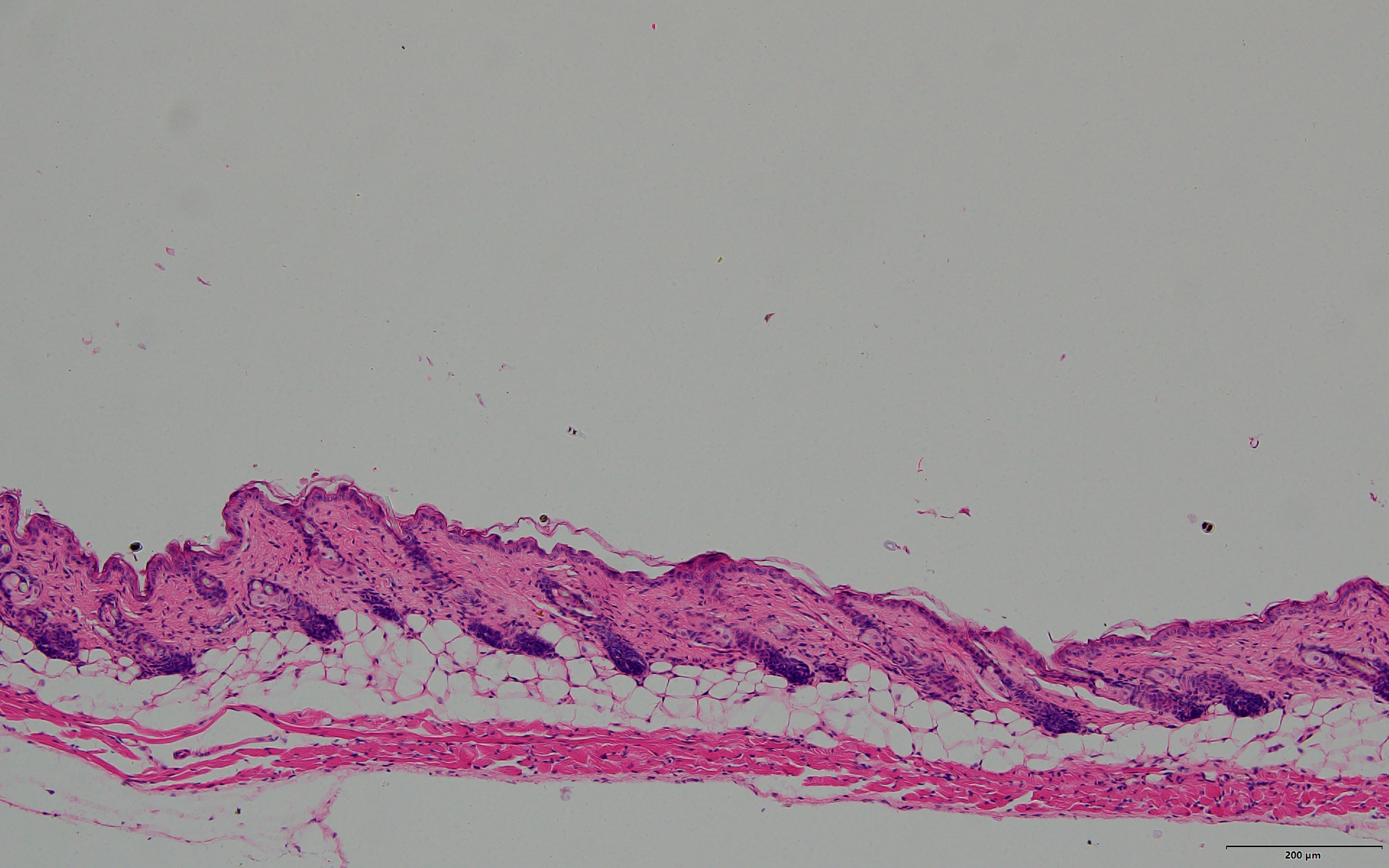

Supplement: Supplementary file 3 — Source data Fig. 2 [file 44319_2024_327_MOESM3_ESM.zip › Figure 2/2A/P23/KO/1.tif]

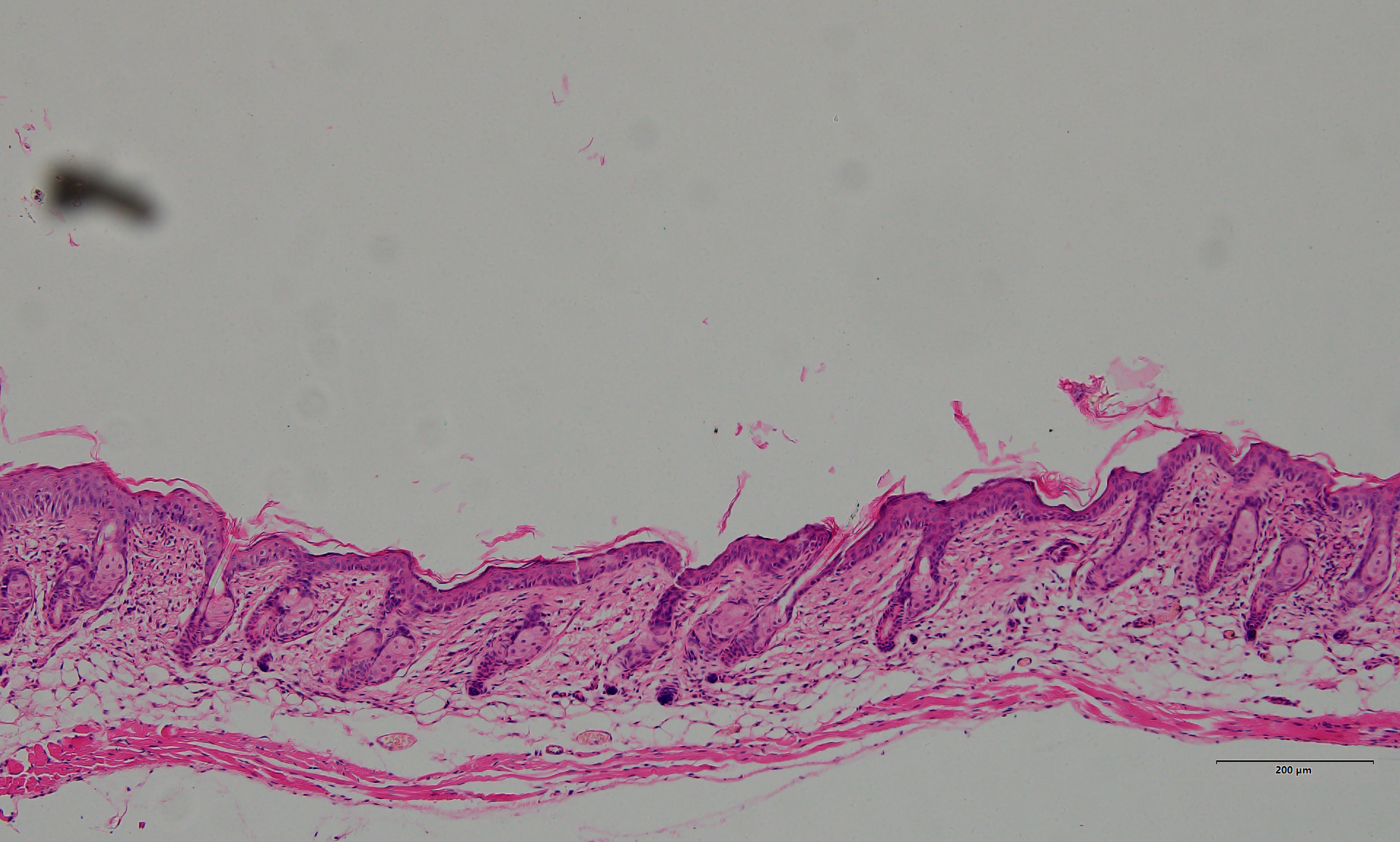

Supplement: Supplementary file 3 — Source data Fig. 2 [file 44319_2024_327_MOESM3_ESM.zip › Figure 2/2A/P23/WT/1.tif]

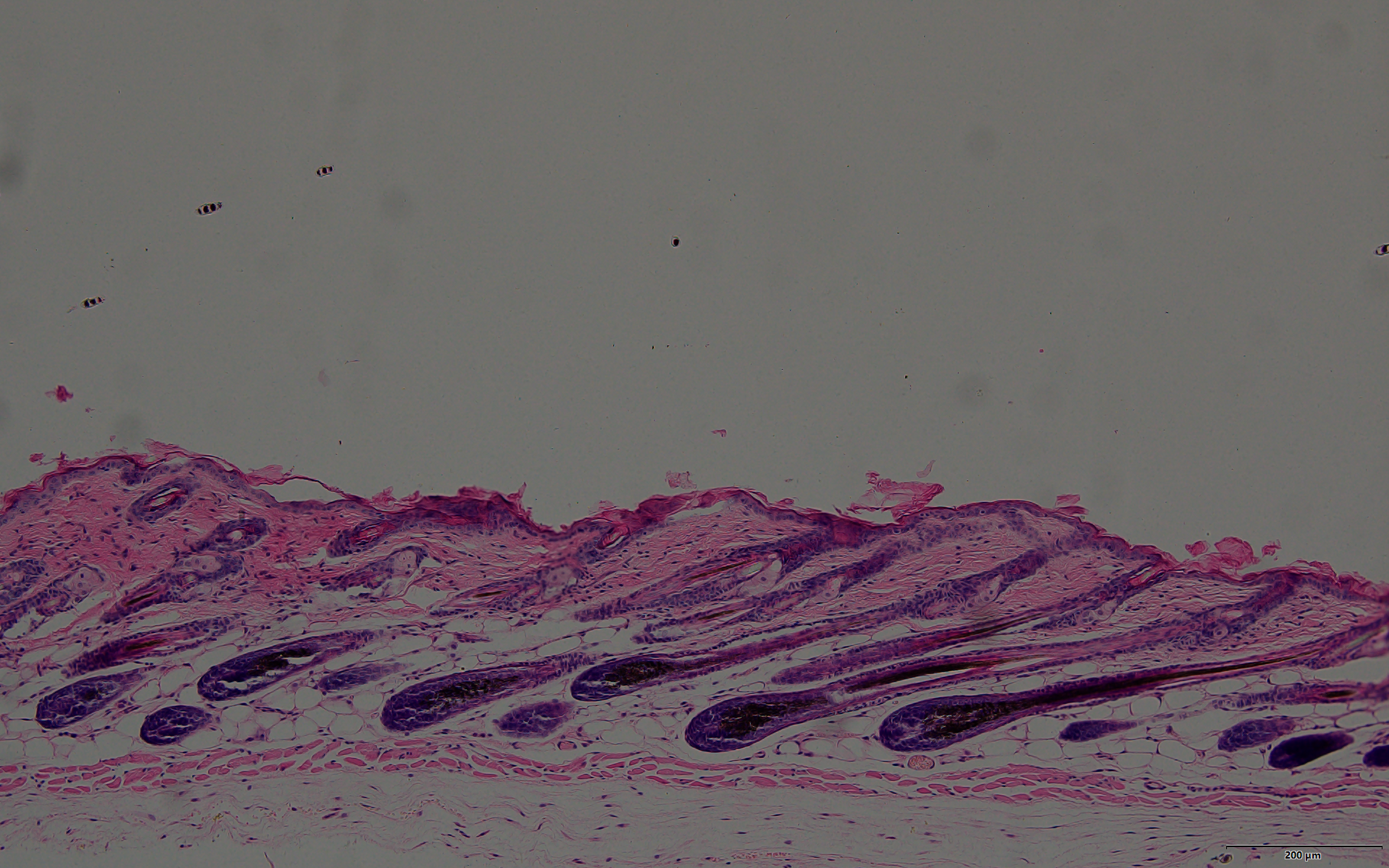

Supplement: Supplementary file 3 — Source data Fig. 2 [file 44319_2024_327_MOESM3_ESM.zip › Figure 2/2A/P25/KO/1.tif]

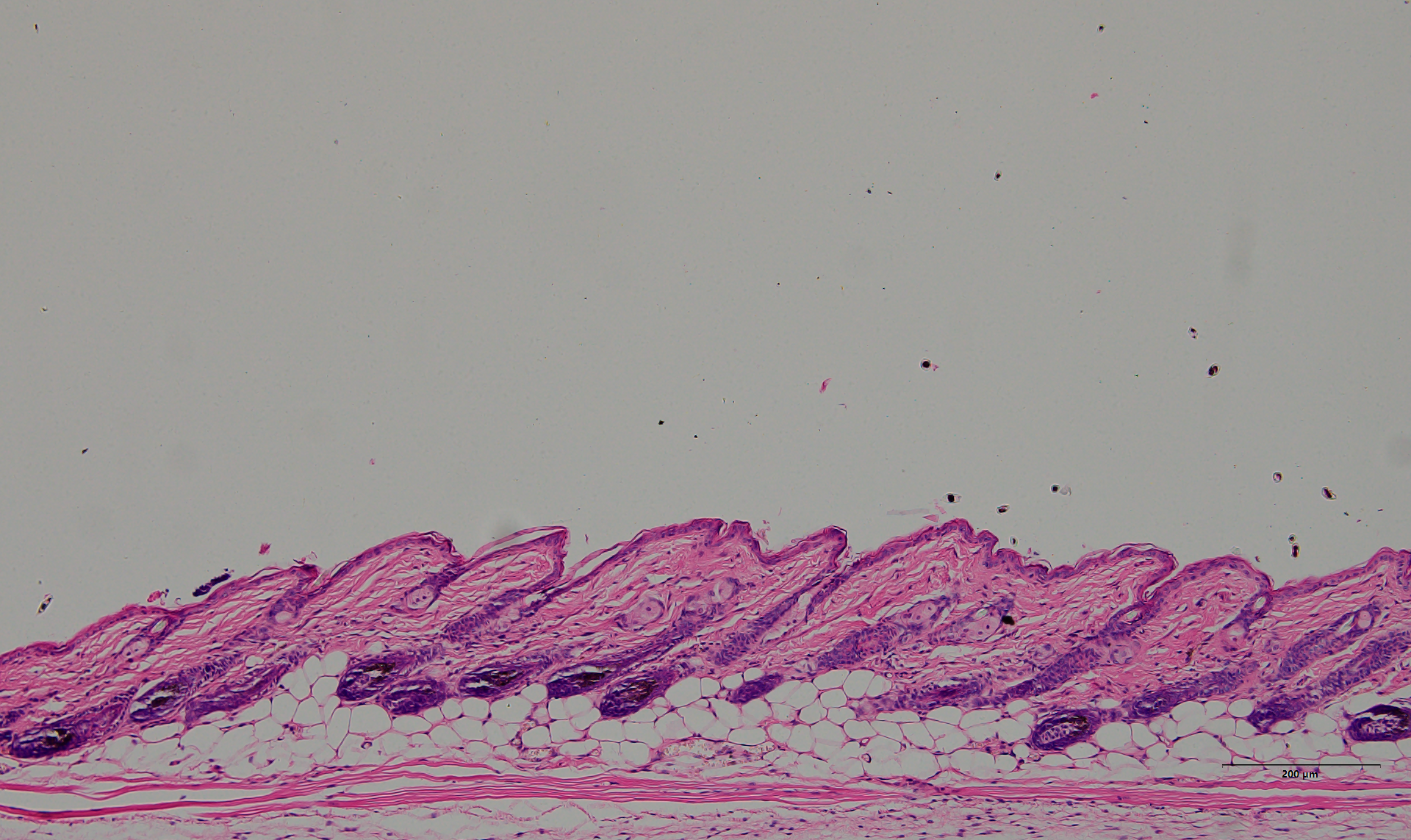

Supplement: Supplementary file 3 — Source data Fig. 2 [file 44319_2024_327_MOESM3_ESM.zip › Figure 2/2A/P25/WT/1.tif]

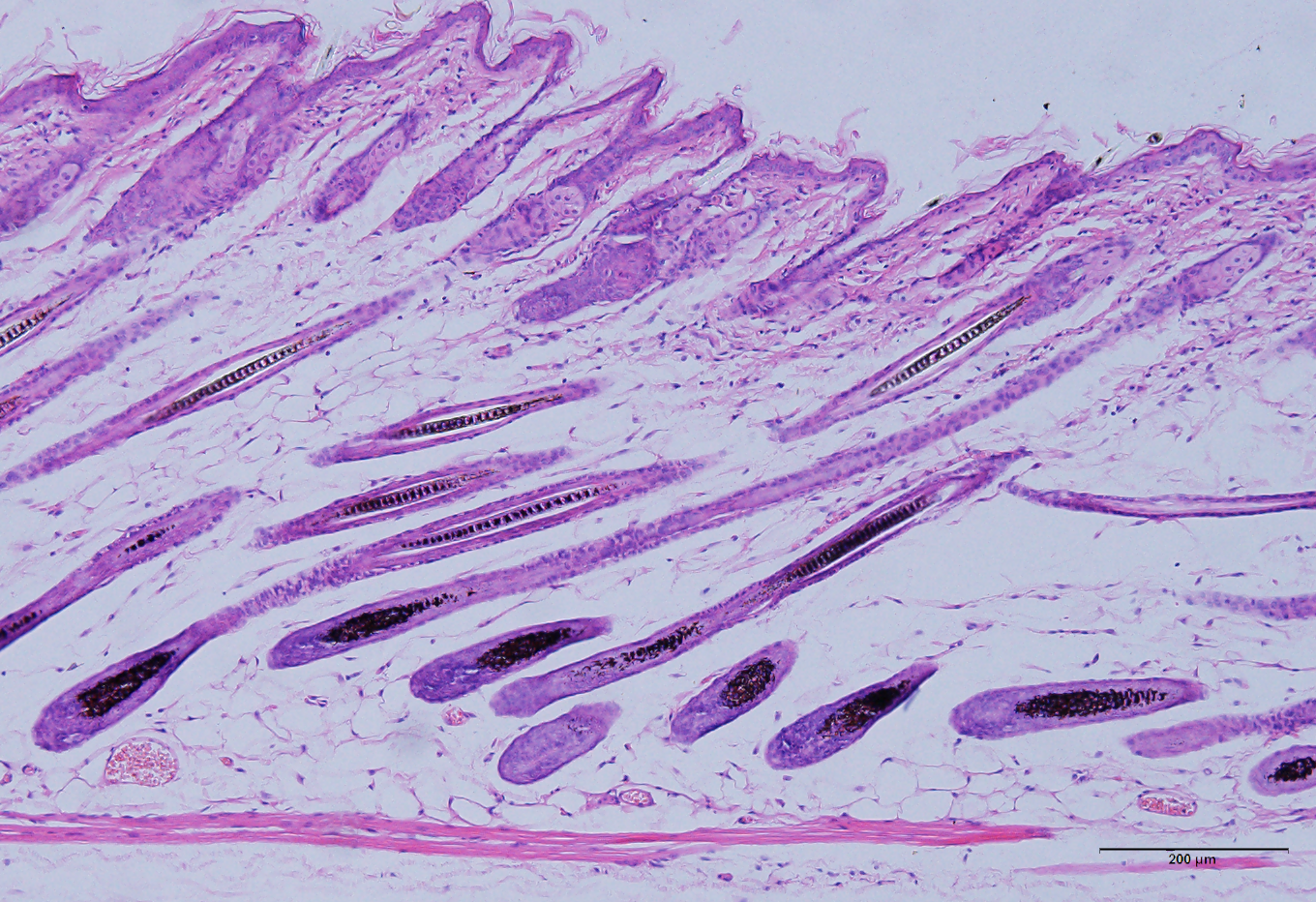

Supplement: Supplementary file 3 — Source data Fig. 2 [file 44319_2024_327_MOESM3_ESM.zip › Figure 2/2A/P35/KO/1.tif]

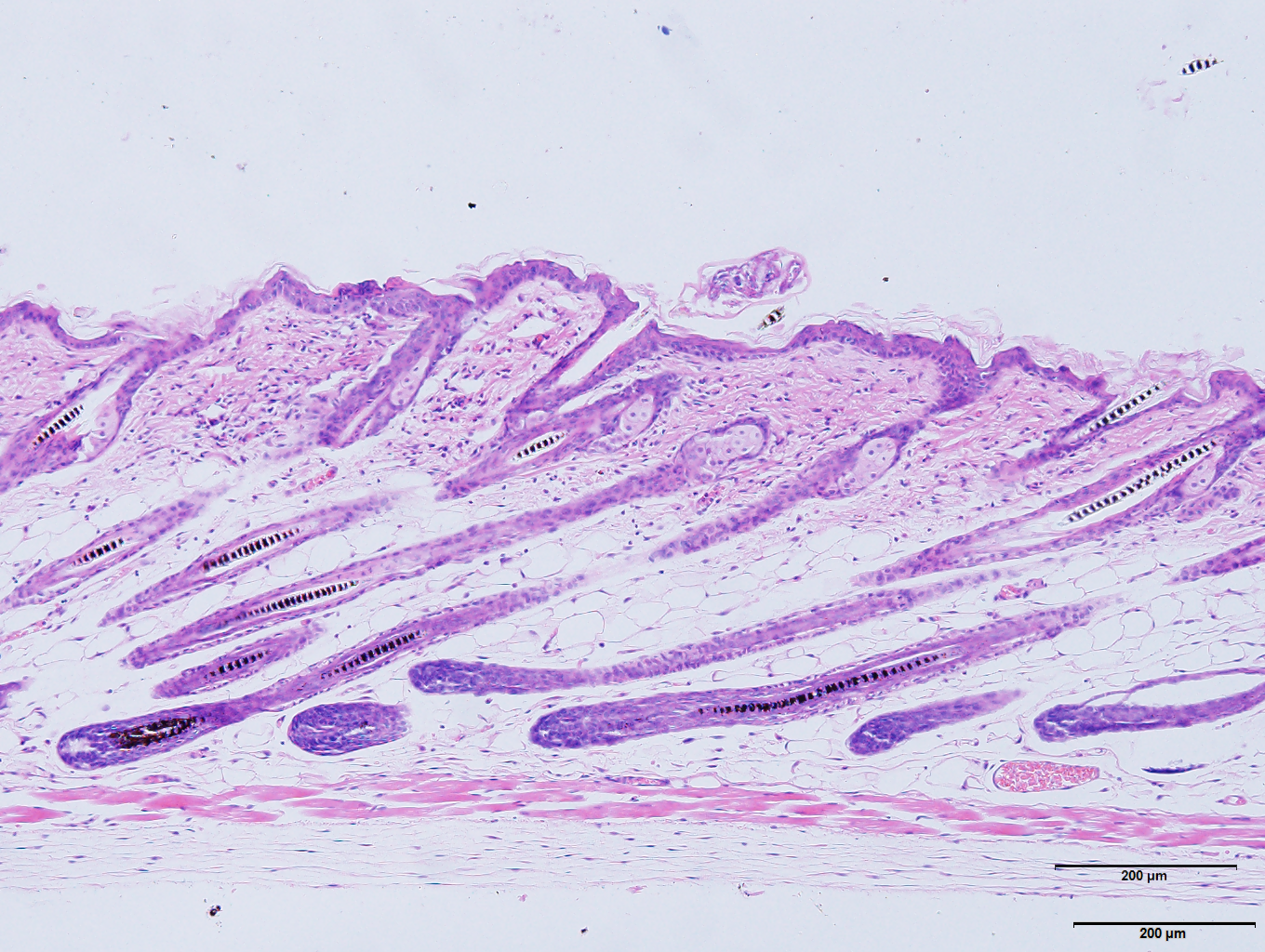

Supplement: Supplementary file 3 — Source data Fig. 2 [file 44319_2024_327_MOESM3_ESM.zip › Figure 2/2A/P35/WT/1.tif]

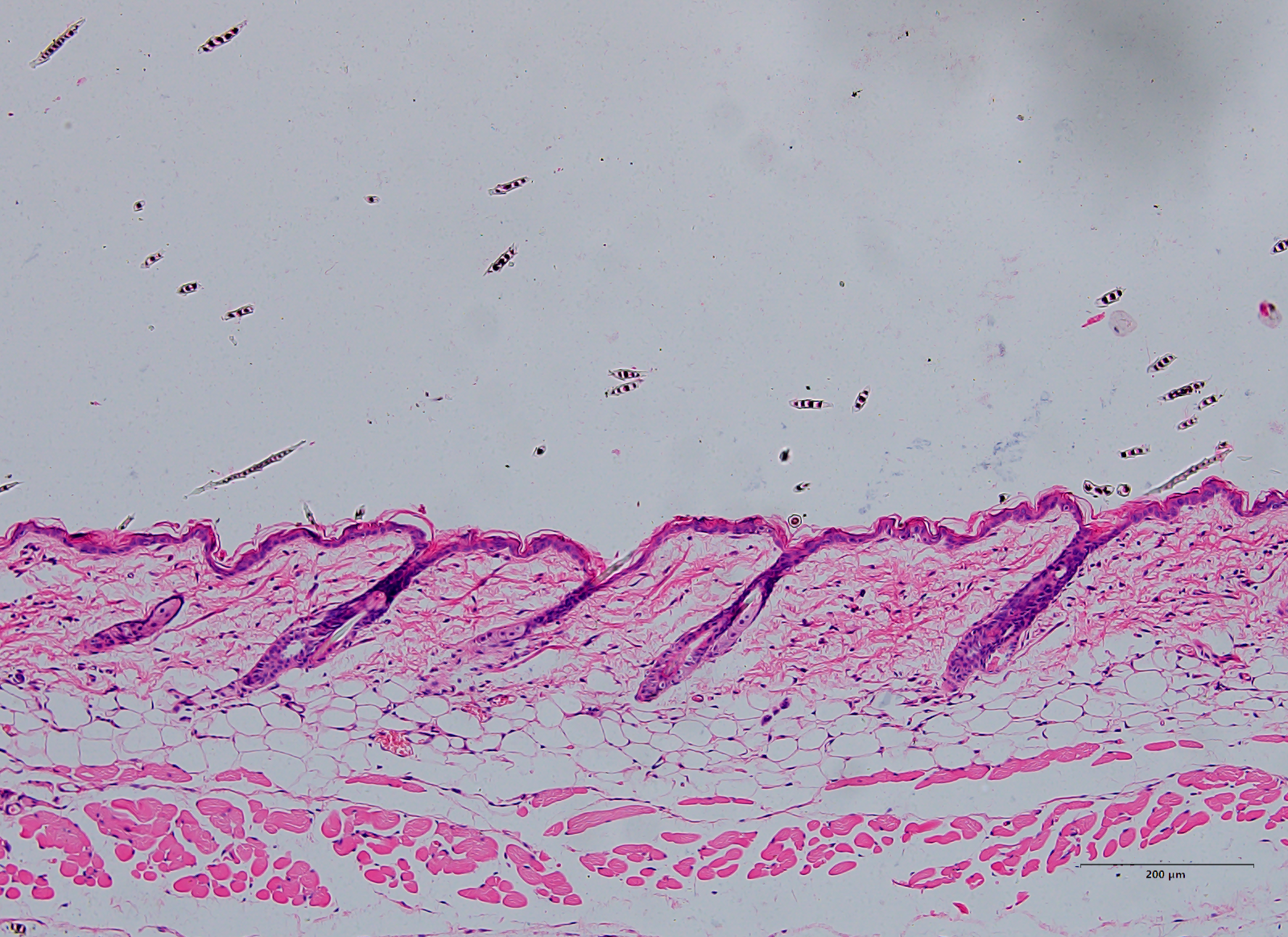

Supplement: Supplementary file 3 — Source data Fig. 2 [file 44319_2024_327_MOESM3_ESM.zip › Figure 2/2A/P42/KO/1.tif]

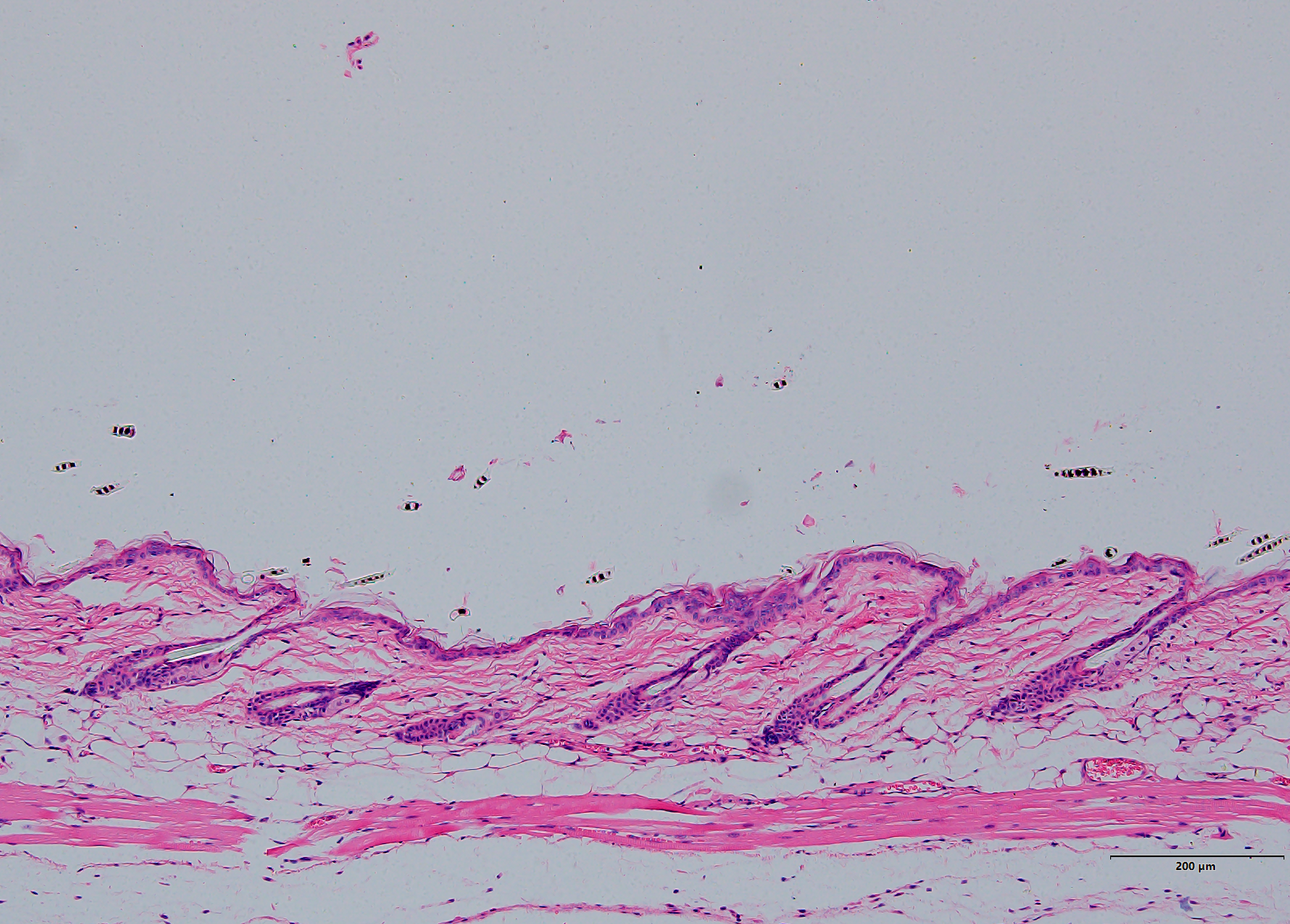

Supplement: Supplementary file 3 — Source data Fig. 2 [file 44319_2024_327_MOESM3_ESM.zip › Figure 2/2A/P42/WT/1.tif]

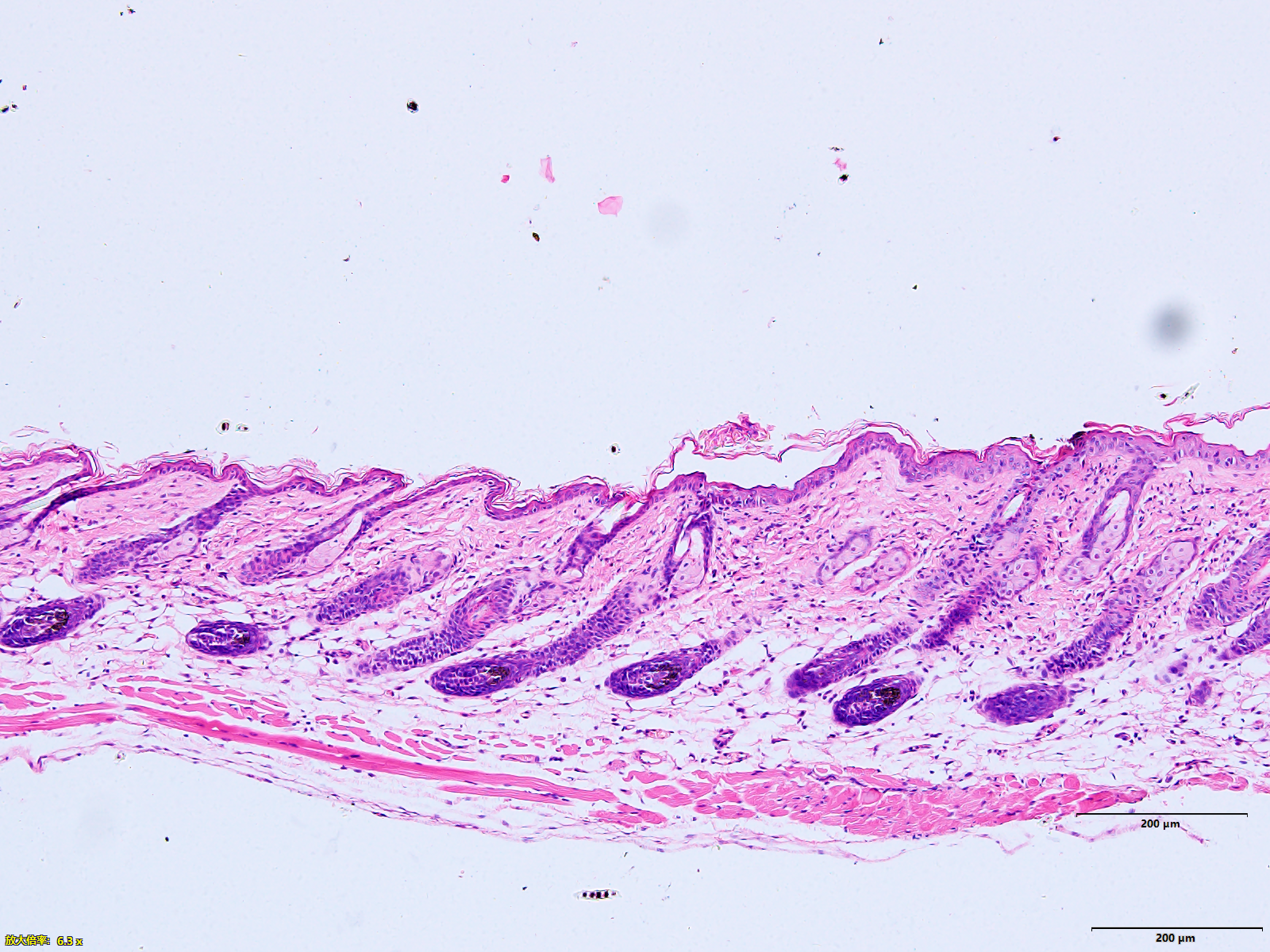

Supplement: Supplementary file 3 — Source data Fig. 2 [file 44319_2024_327_MOESM3_ESM.zip › Figure 2/2A/P50/KO/1.tif]

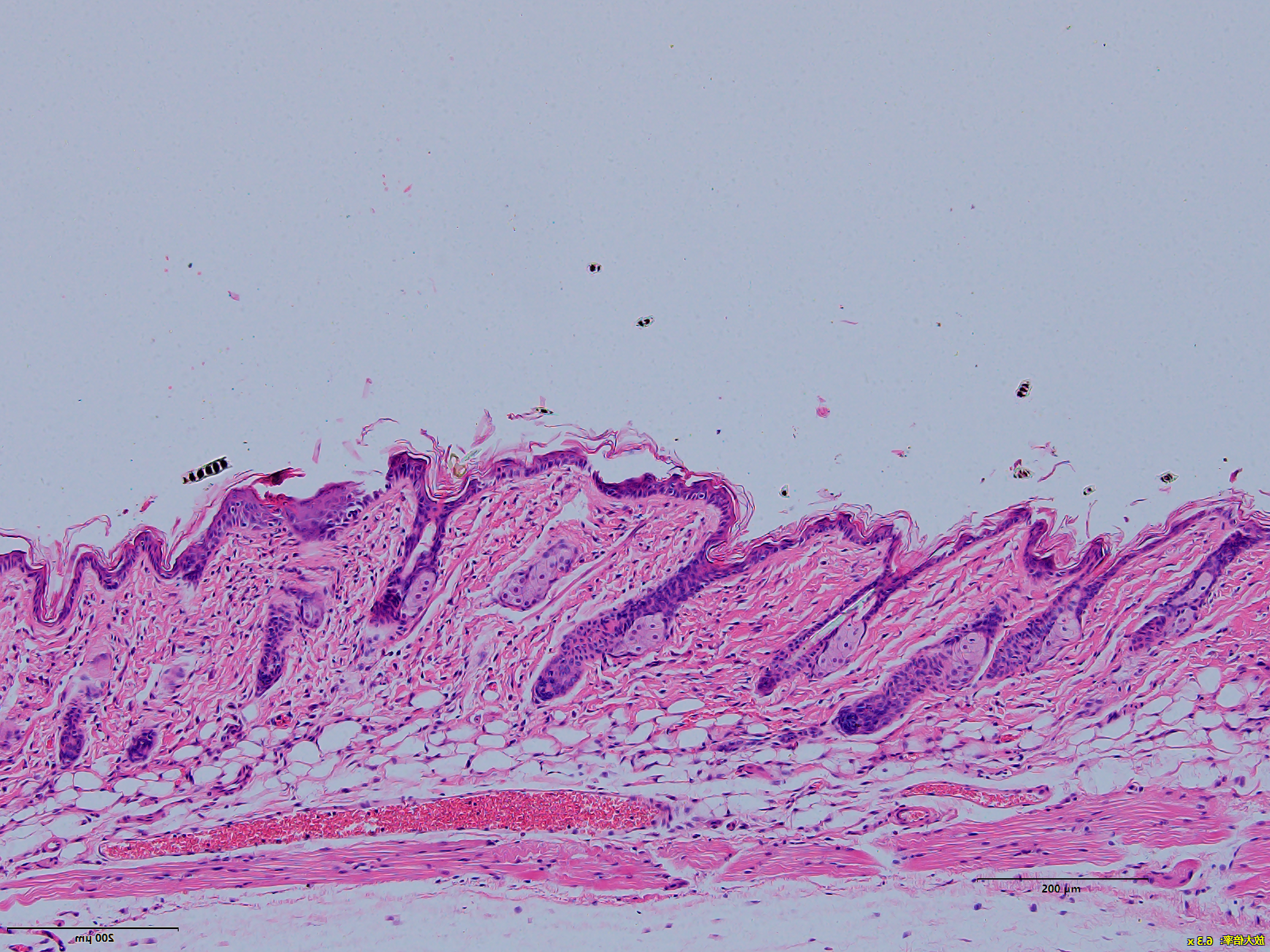

Supplement: Supplementary file 3 — Source data Fig. 2 [file 44319_2024_327_MOESM3_ESM.zip › Figure 2/2A/P50/WT/1.tif]

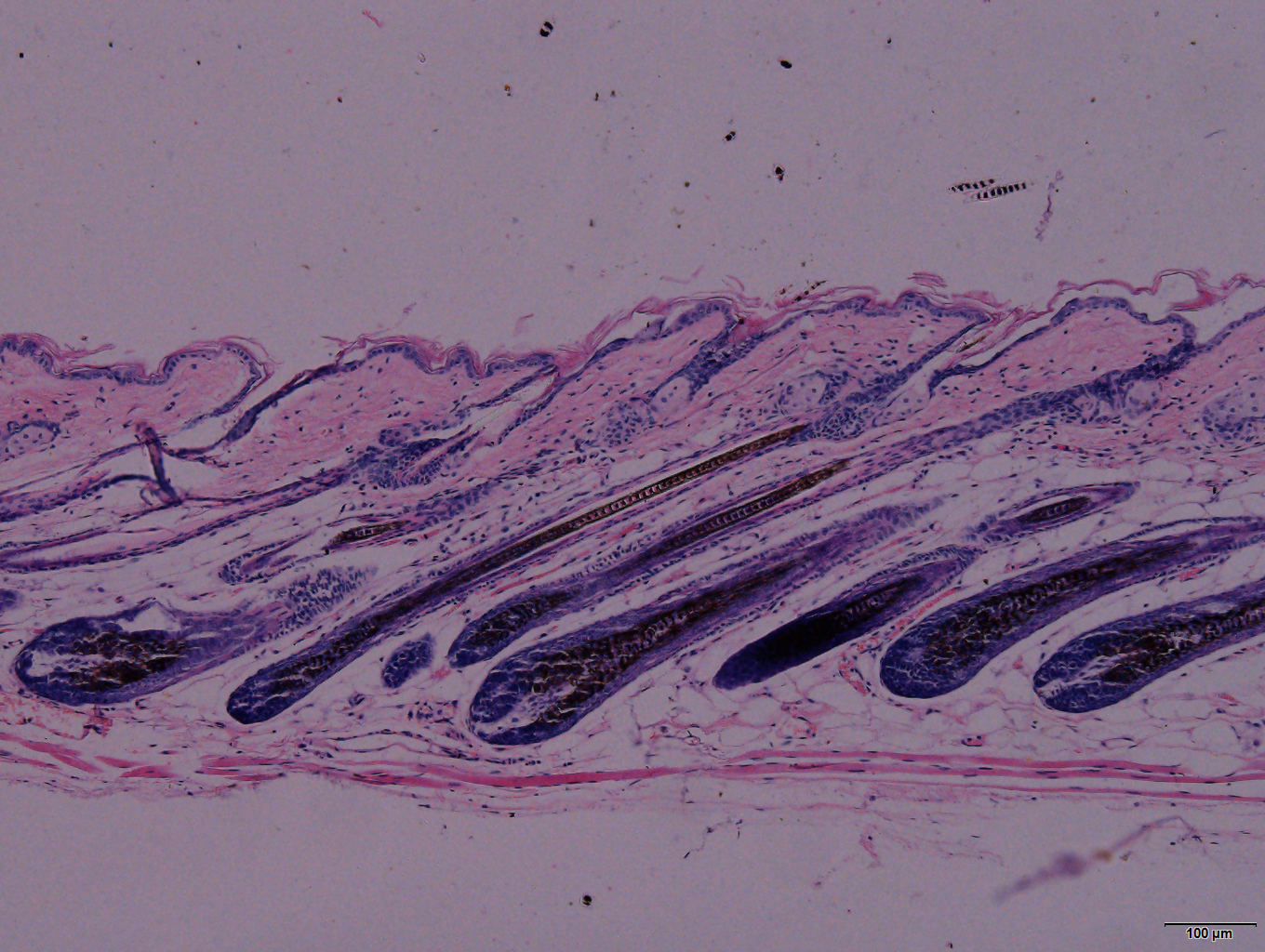

Supplement: Supplementary file 3 — Source data Fig. 2 [file 44319_2024_327_MOESM3_ESM.zip › Figure 2/2A/P54/KO/1.tif]

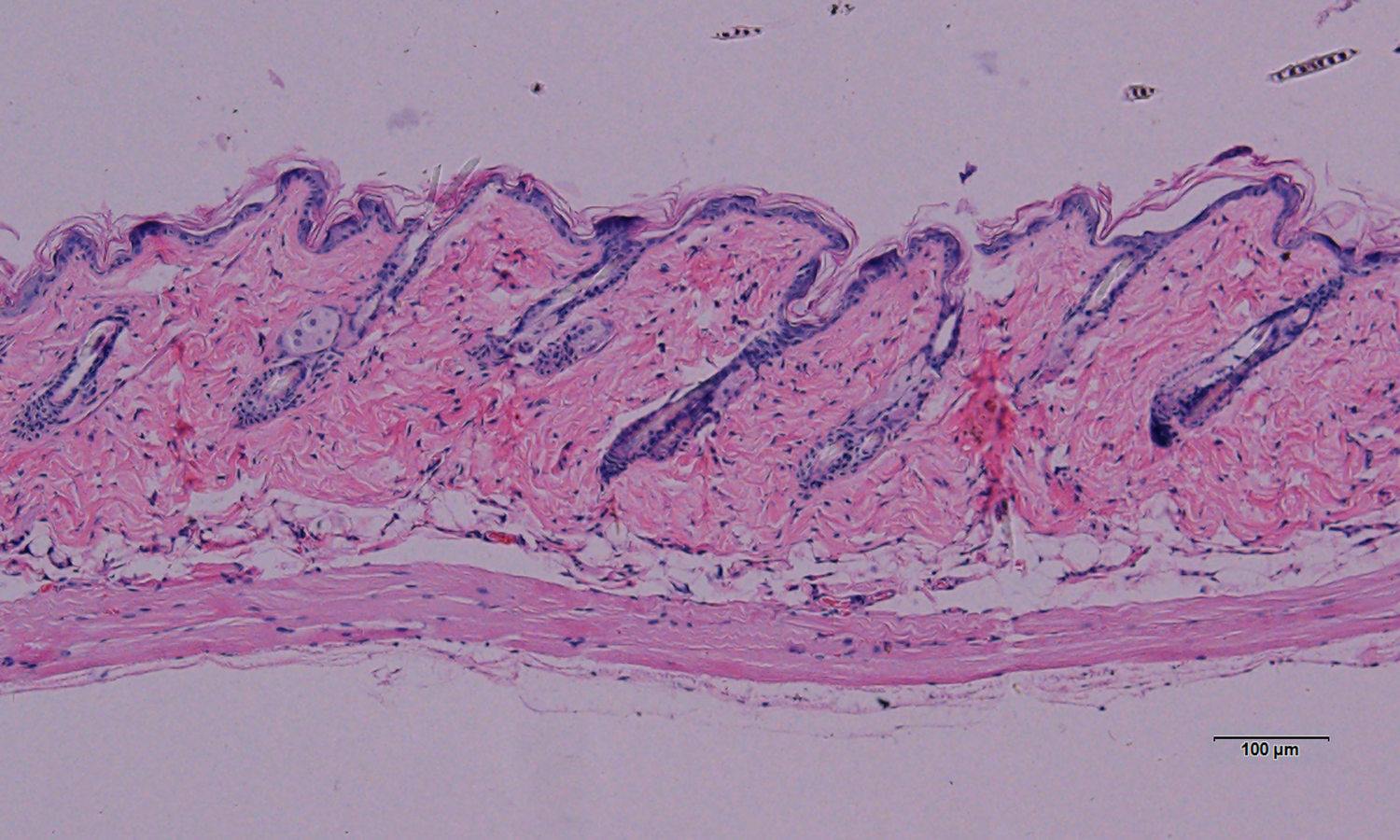

Supplement: Supplementary file 3 — Source data Fig. 2 [file 44319_2024_327_MOESM3_ESM.zip › Figure 2/2A/P54/WT/1.tif]

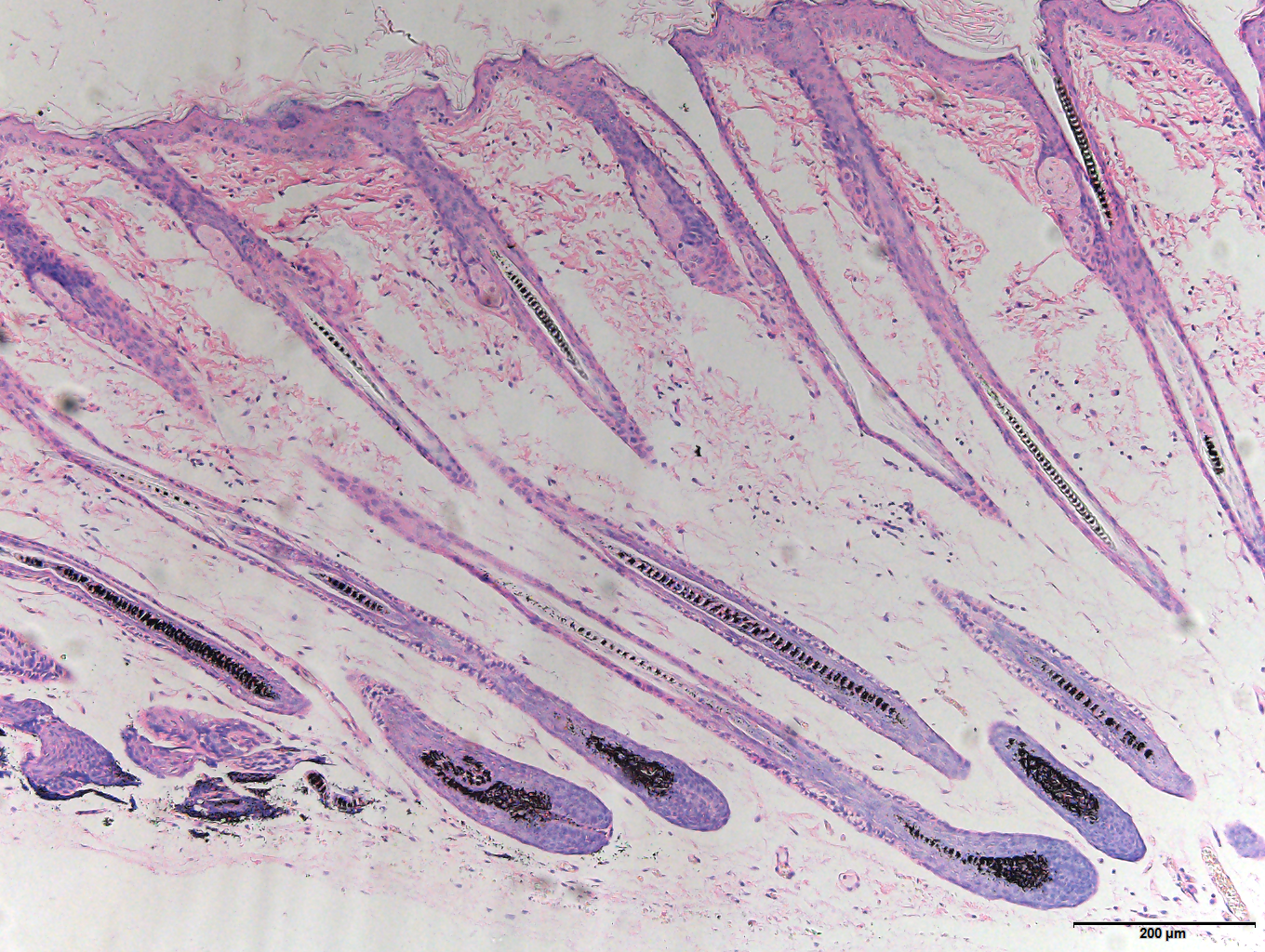

Supplement: Supplementary file 3 — Source data Fig. 2 [file 44319_2024_327_MOESM3_ESM.zip › Figure 2/2A/P73/KO/1.tif]

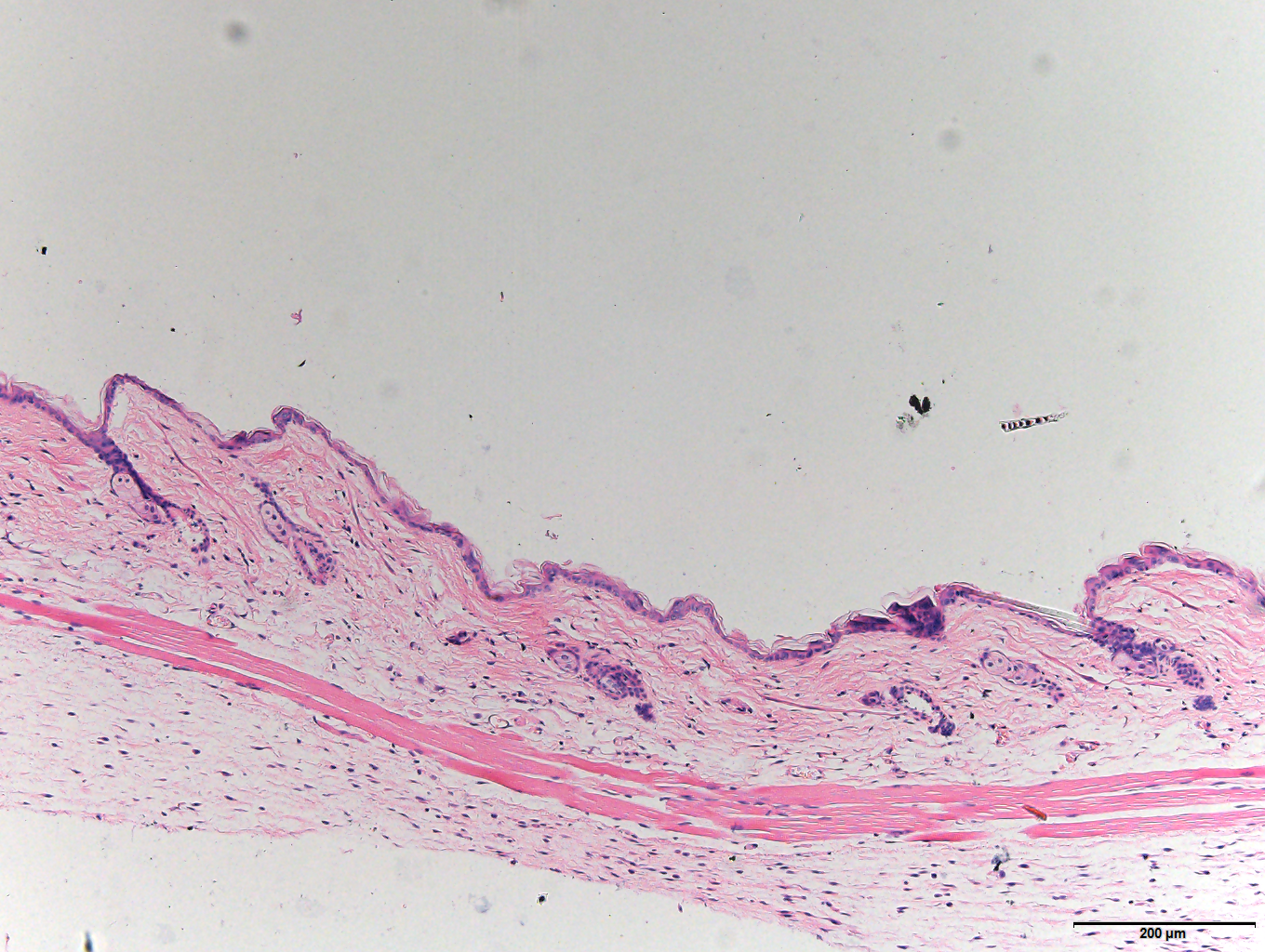

Supplement: Supplementary file 3 — Source data Fig. 2 [file 44319_2024_327_MOESM3_ESM.zip › Figure 2/2A/P73/WT/1.tif]

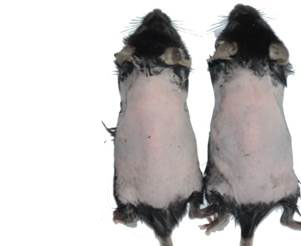

Supplement: Supplementary file 3 — Source data Fig. 2 [file 44319_2024_327_MOESM3_ESM.zip › Figure 2/2B/Day1.jpg]

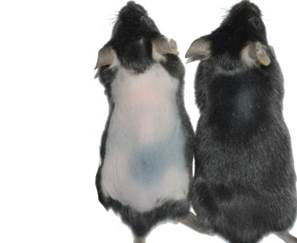

Supplement: Supplementary file 3 — Source data Fig. 2 [file 44319_2024_327_MOESM3_ESM.zip › Figure 2/2B/Day13.jpg]

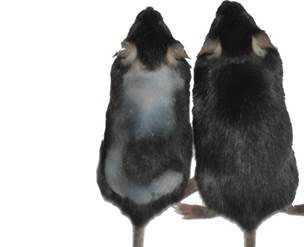

Supplement: Supplementary file 3 — Source data Fig. 2 [file 44319_2024_327_MOESM3_ESM.zip › Figure 2/2B/Day17.jpg]

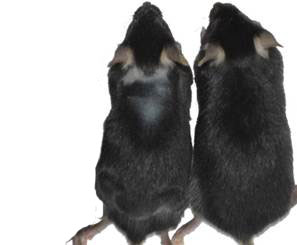

Supplement: Supplementary file 3 — Source data Fig. 2 [file 44319_2024_327_MOESM3_ESM.zip › Figure 2/2B/Day21.jpg]

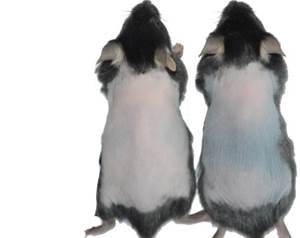

Supplement: Supplementary file 3 — Source data Fig. 2 [file 44319_2024_327_MOESM3_ESM.zip › Figure 2/2B/Day3.jpg]

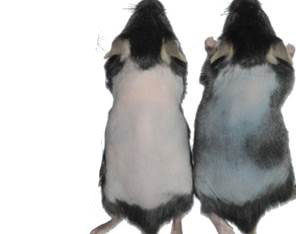

Supplement: Supplementary file 3 — Source data Fig. 2 [file 44319_2024_327_MOESM3_ESM.zip › Figure 2/2B/Day6.jpg]

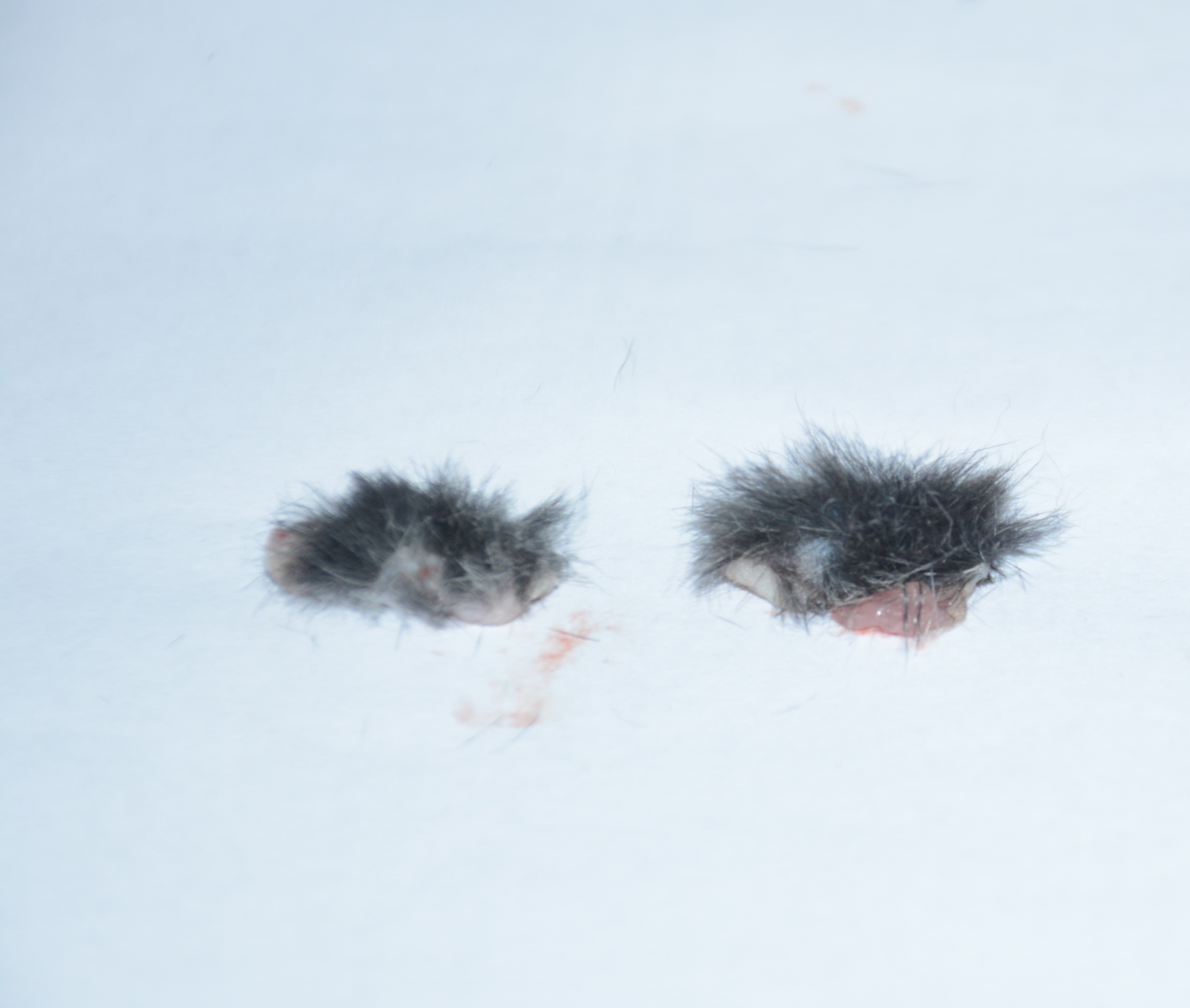

Supplement: Supplementary file 3 — Source data Fig. 2 [file 44319_2024_327_MOESM3_ESM.zip › Figure 2/2C/1.JPG]

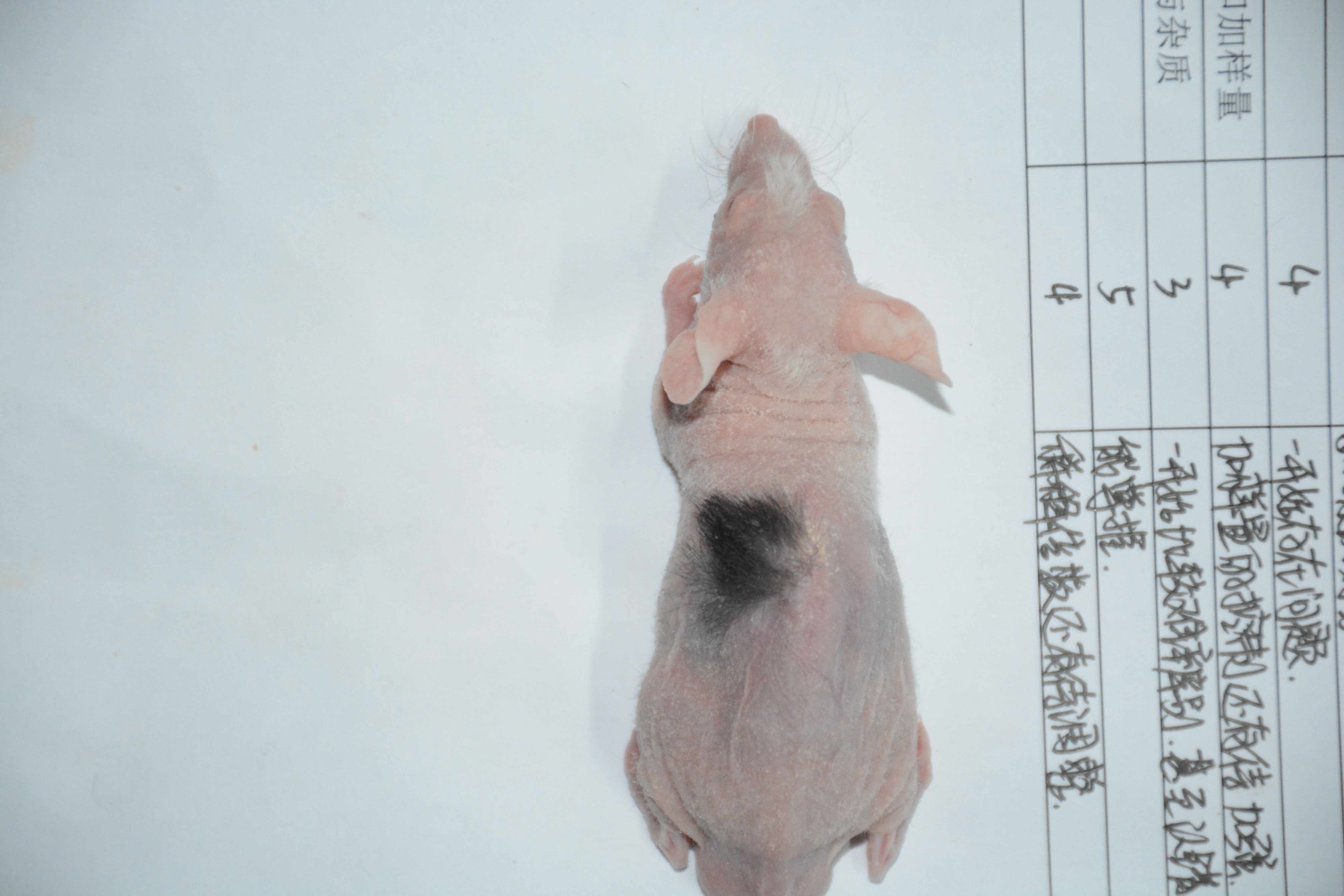

Supplement: Supplementary file 3 — Source data Fig. 2 [file 44319_2024_327_MOESM3_ESM.zip › Figure 2/2C/KO/1.JPG]

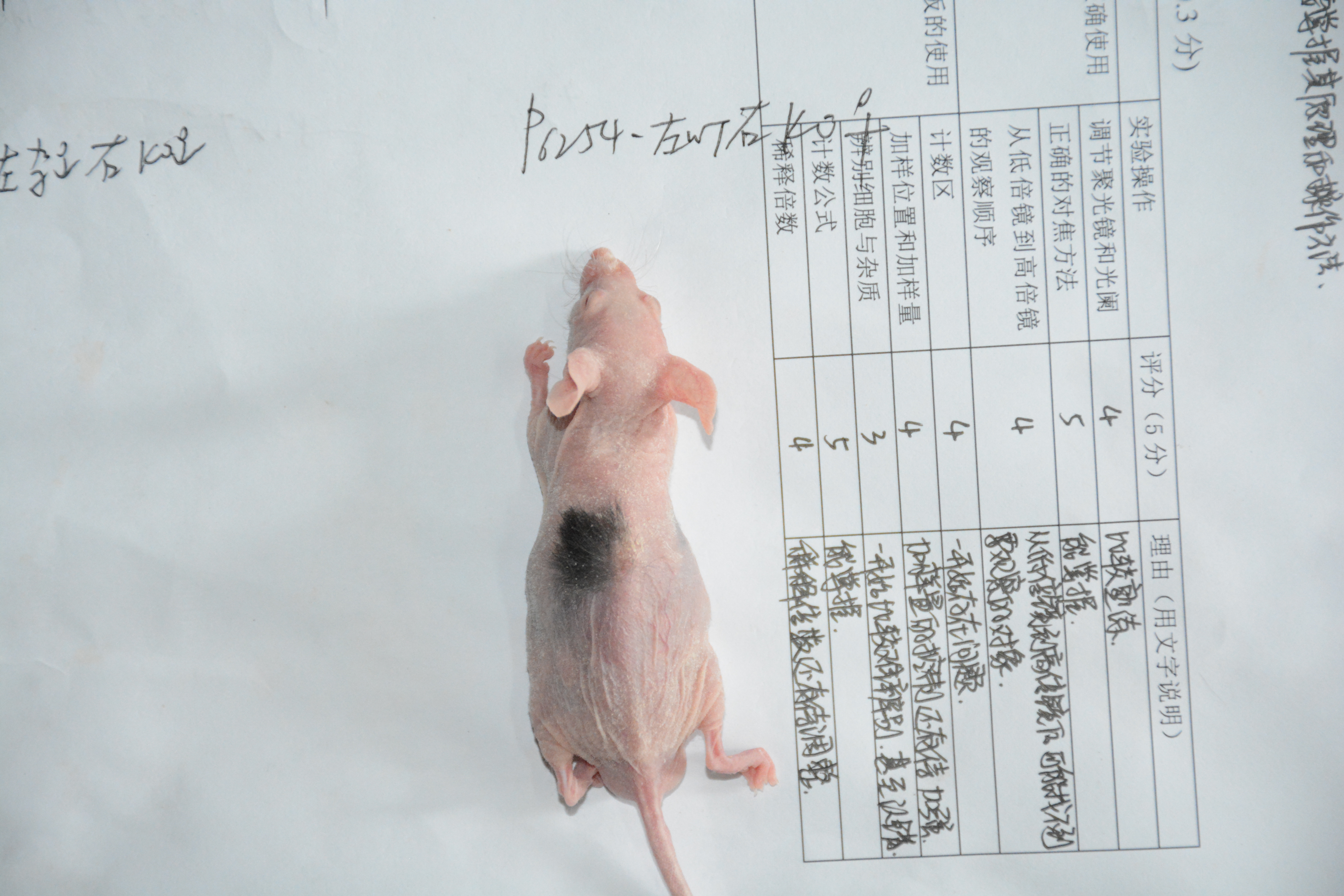

Supplement: Supplementary file 3 — Source data Fig. 2 [file 44319_2024_327_MOESM3_ESM.zip › Figure 2/2C/WT/1.JPG]

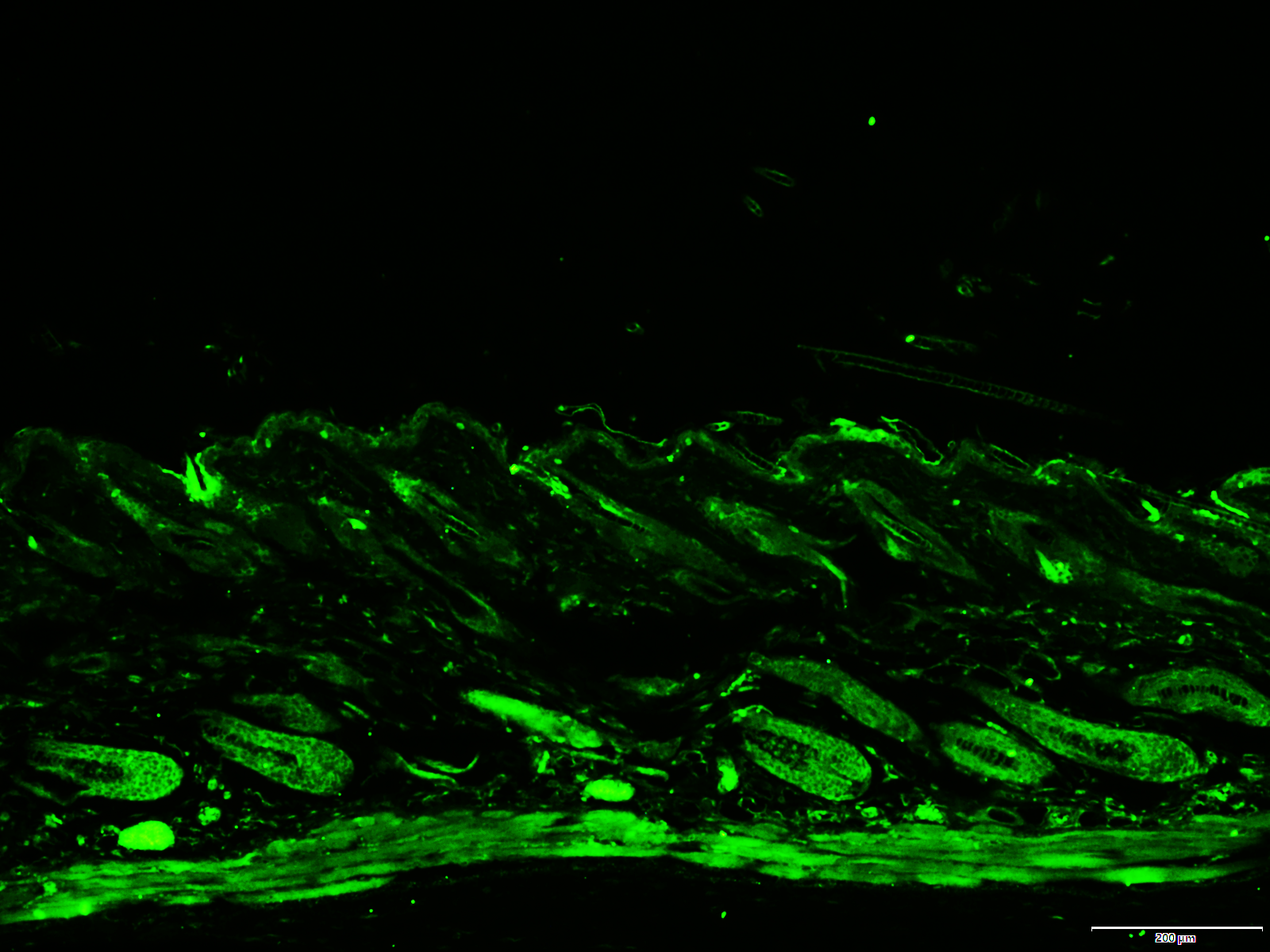

Supplement: Supplementary file 4 — Source data Fig. 3 [file 44319_2024_327_MOESM4_ESM.zip › Figure 3/3A/Anagen/1 (1).tif]

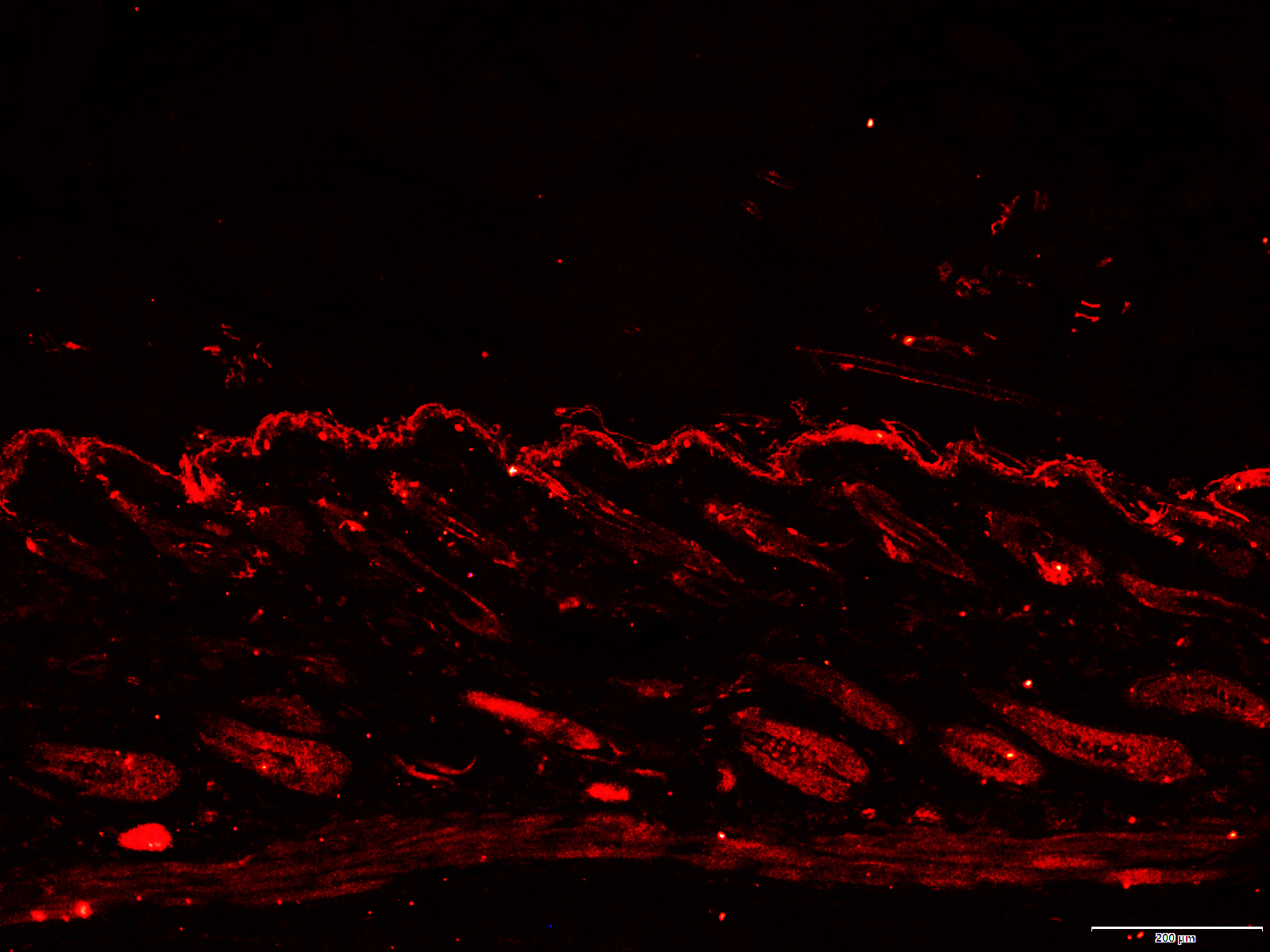

Supplement: Supplementary file 4 — Source data Fig. 3 [file 44319_2024_327_MOESM4_ESM.zip › Figure 3/3A/Anagen/1 (2).tif]

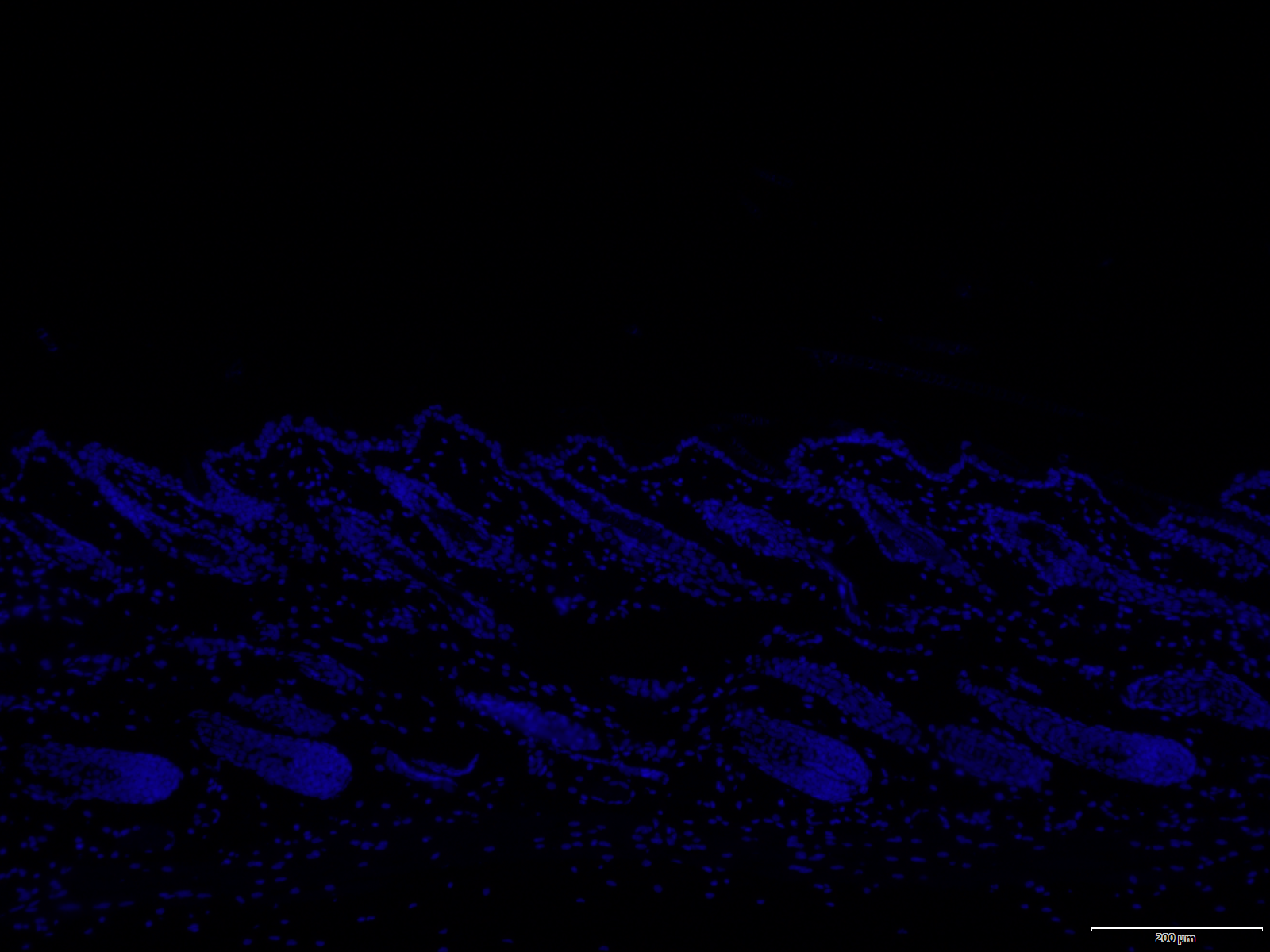

Supplement: Supplementary file 4 — Source data Fig. 3 [file 44319_2024_327_MOESM4_ESM.zip › Figure 3/3A/Anagen/1 (3).tif]

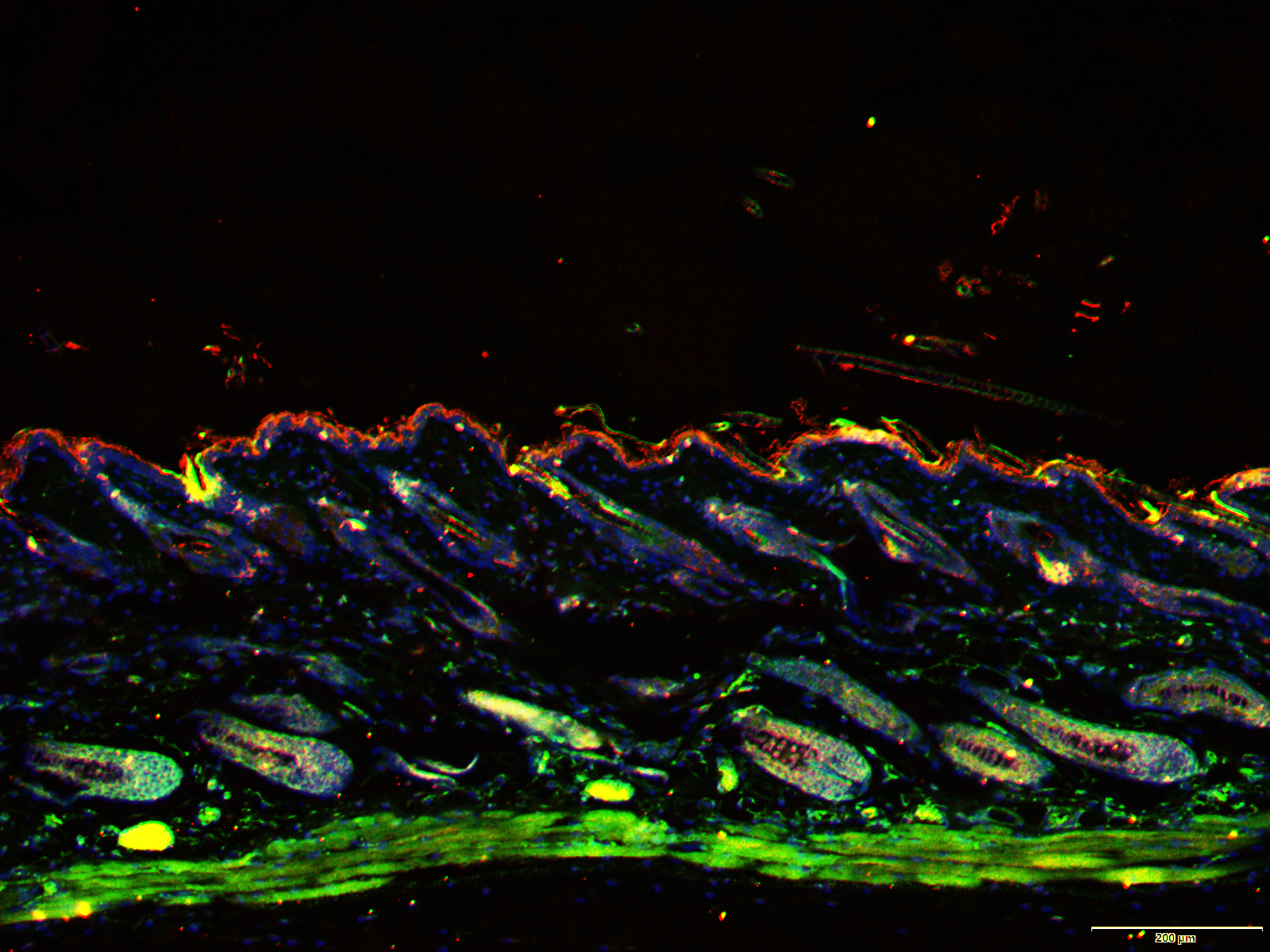

Supplement: Supplementary file 4 — Source data Fig. 3 [file 44319_2024_327_MOESM4_ESM.zip › Figure 3/3A/Anagen/1 (4).tif]

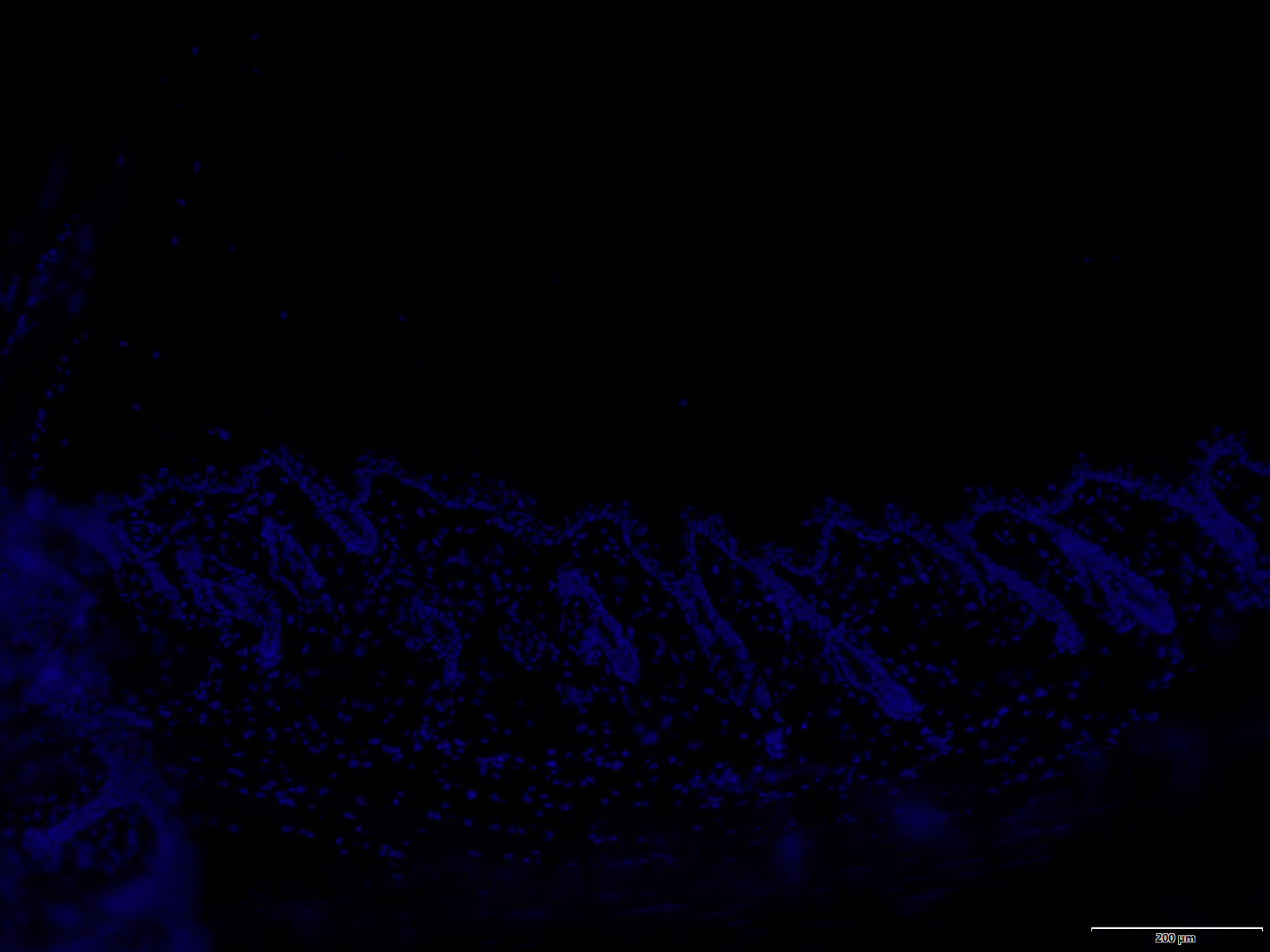

Supplement: Supplementary file 4 — Source data Fig. 3 [file 44319_2024_327_MOESM4_ESM.zip › Figure 3/3A/Catagen/1 (1).tif]

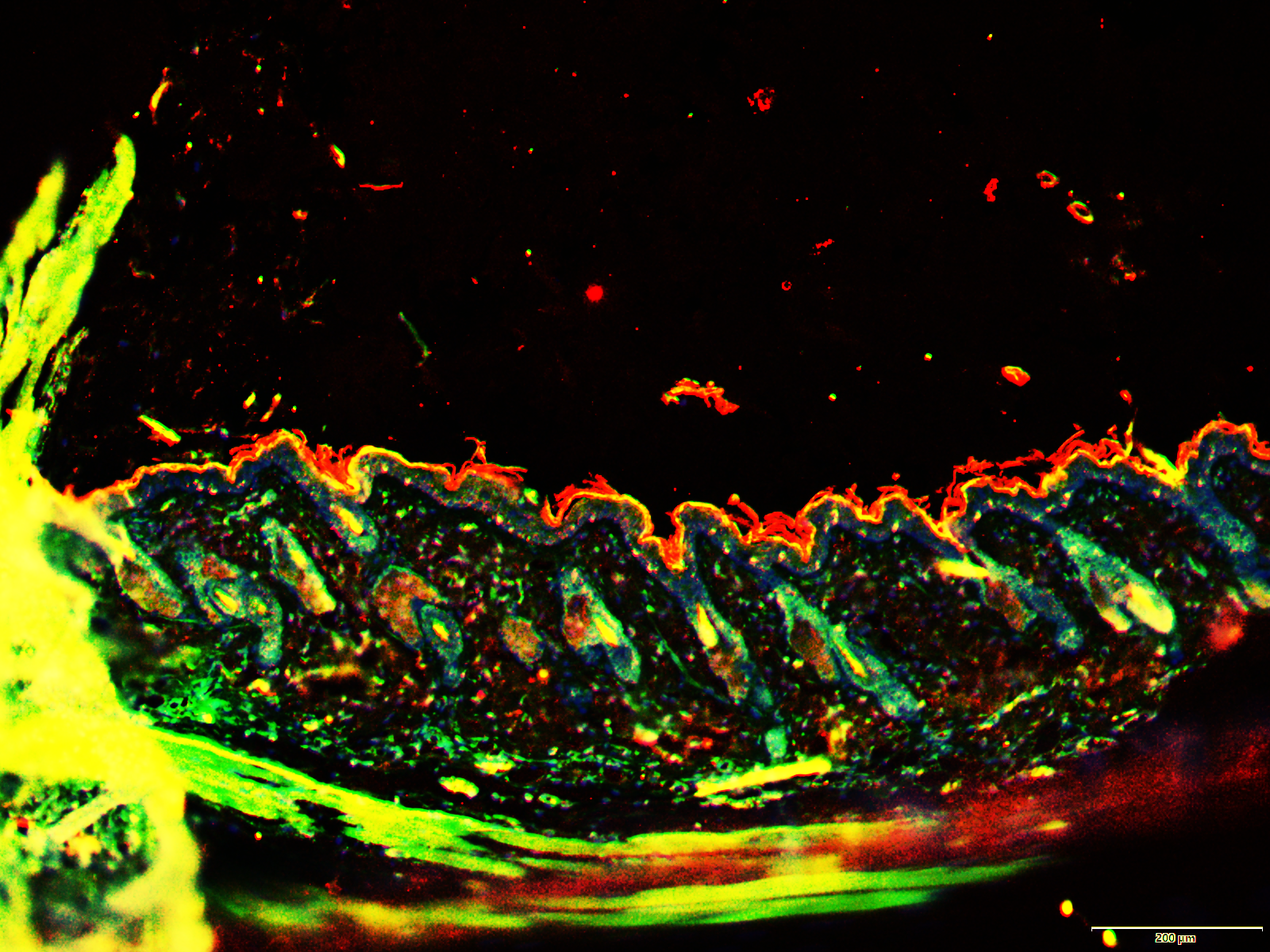

Supplement: Supplementary file 4 — Source data Fig. 3 [file 44319_2024_327_MOESM4_ESM.zip › Figure 3/3A/Catagen/1 (2).tif]

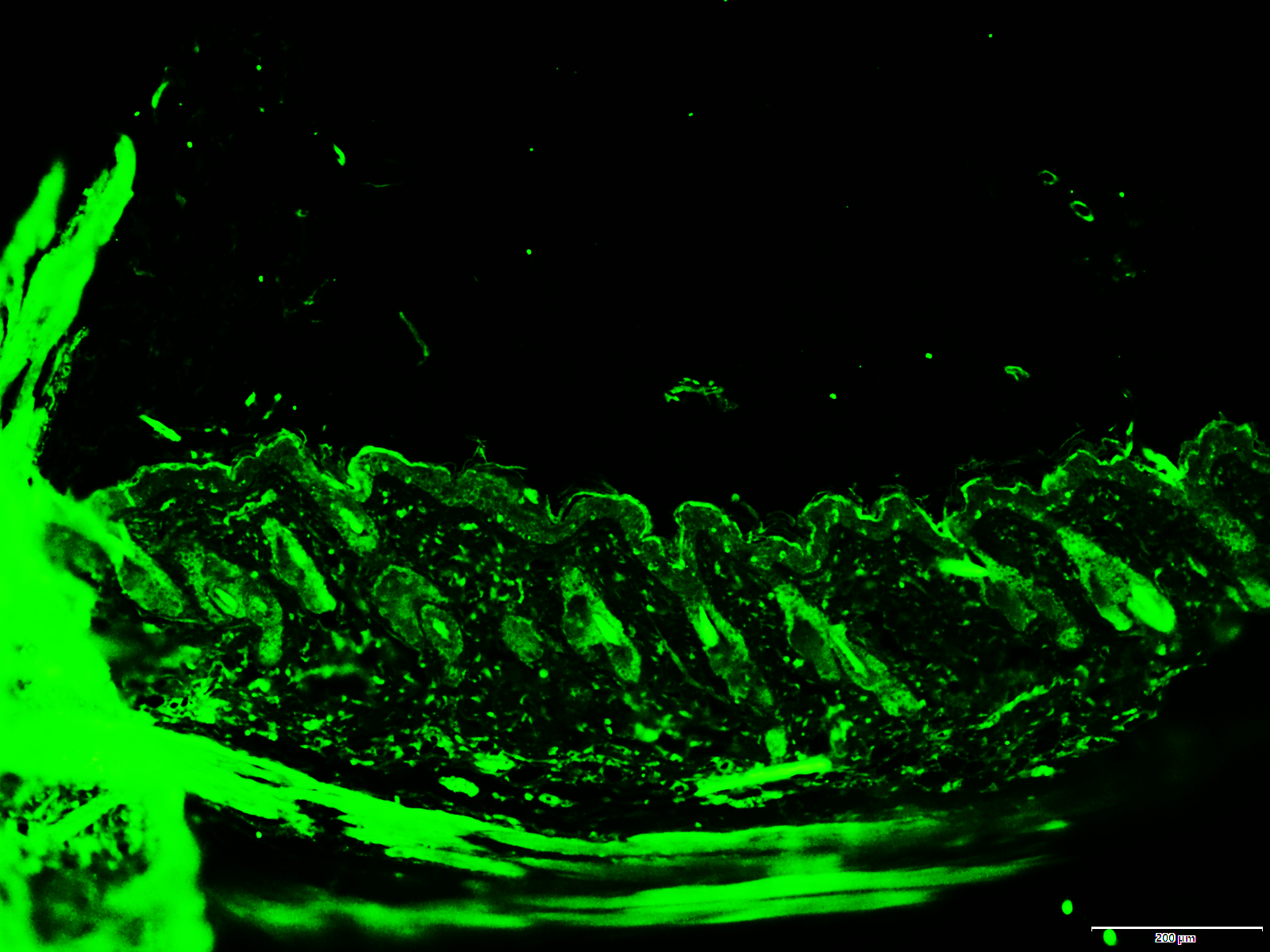

Supplement: Supplementary file 4 — Source data Fig. 3 [file 44319_2024_327_MOESM4_ESM.zip › Figure 3/3A/Catagen/1 (3).tif]

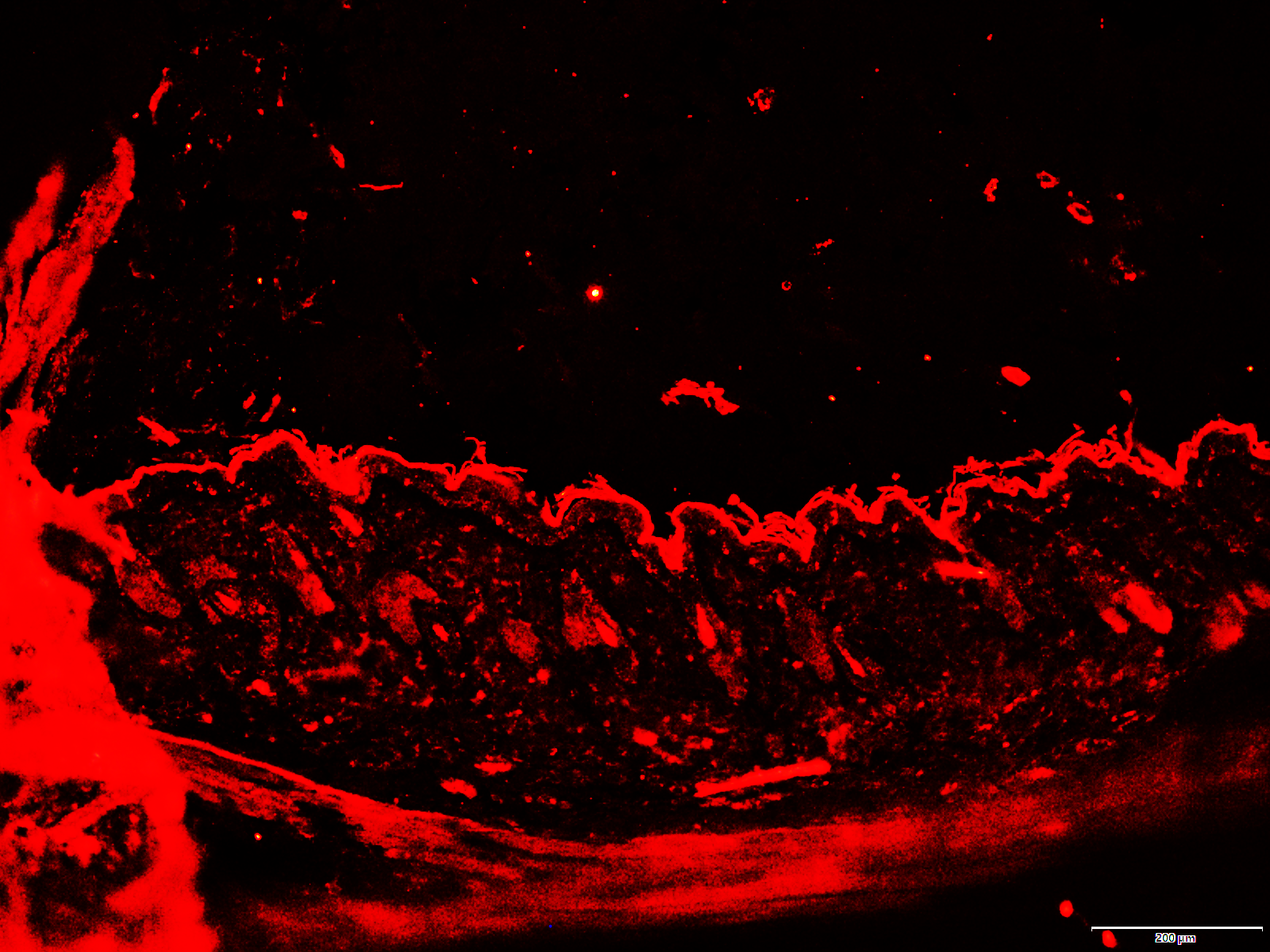

Supplement: Supplementary file 4 — Source data Fig. 3 [file 44319_2024_327_MOESM4_ESM.zip › Figure 3/3A/Catagen/1 (4).tif]

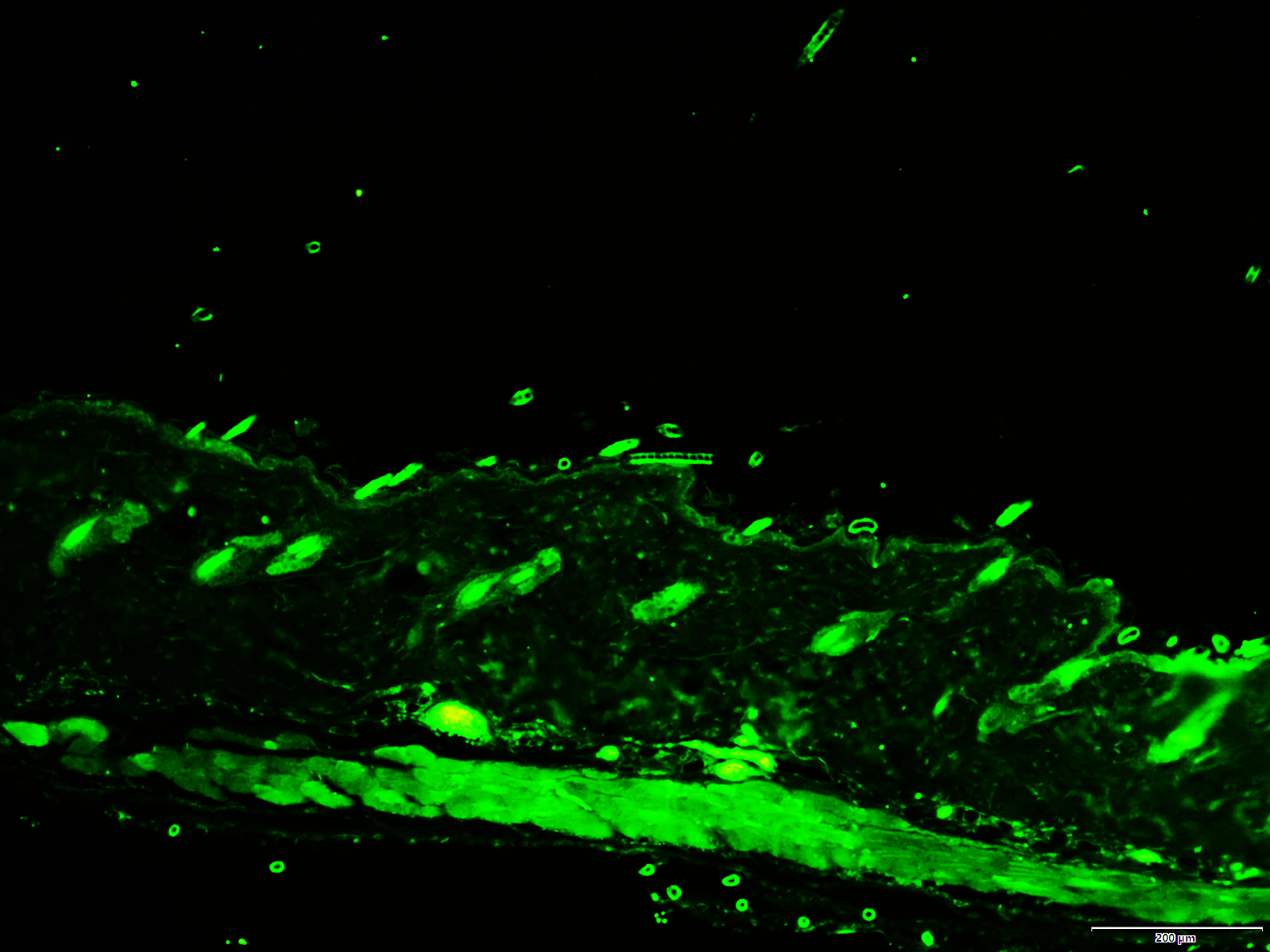

Supplement: Supplementary file 4 — Source data Fig. 3 [file 44319_2024_327_MOESM4_ESM.zip › Figure 3/3A/Telogen/1 (1).tif]

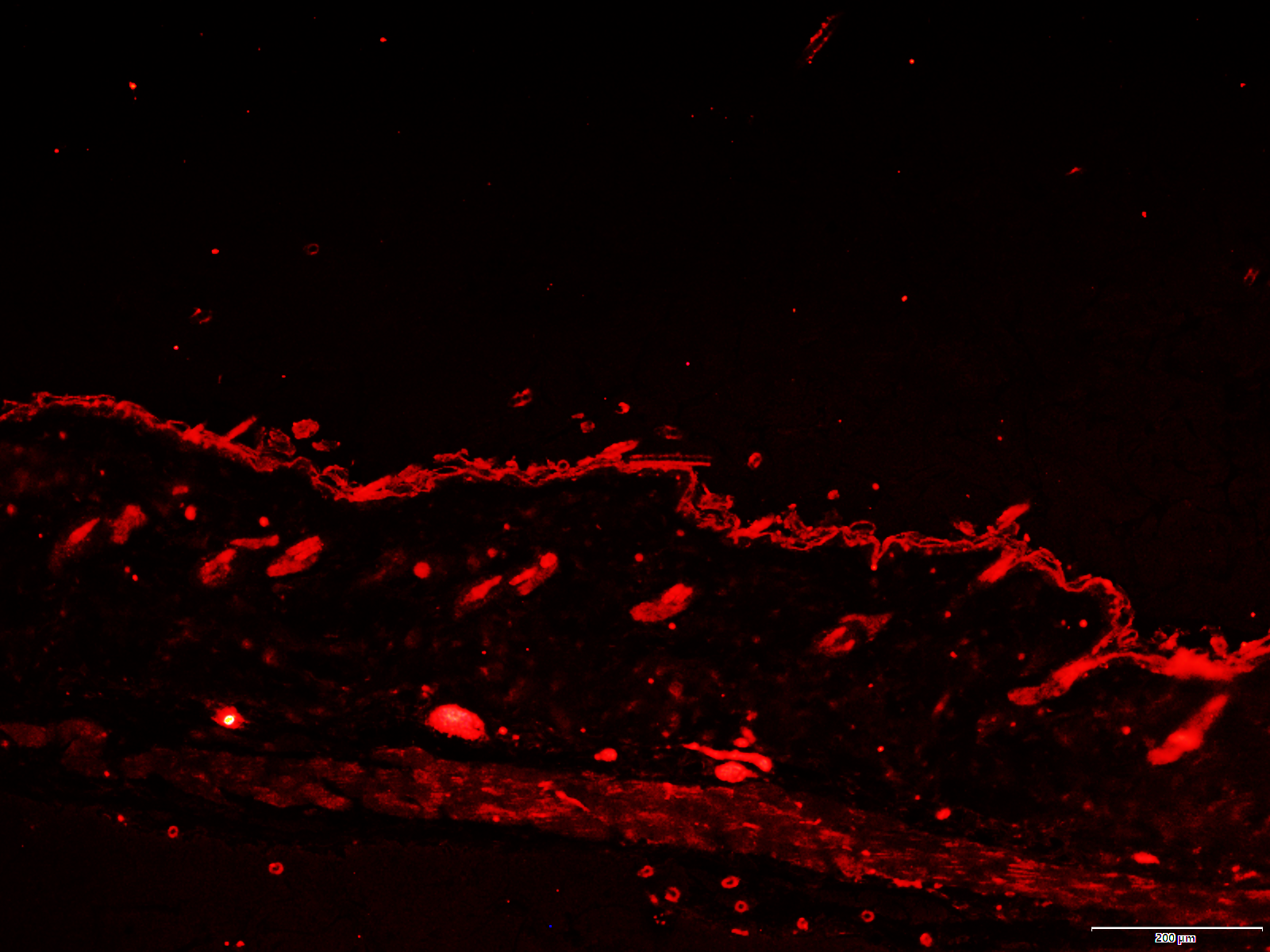

Supplement: Supplementary file 4 — Source data Fig. 3 [file 44319_2024_327_MOESM4_ESM.zip › Figure 3/3A/Telogen/1 (2).tif]

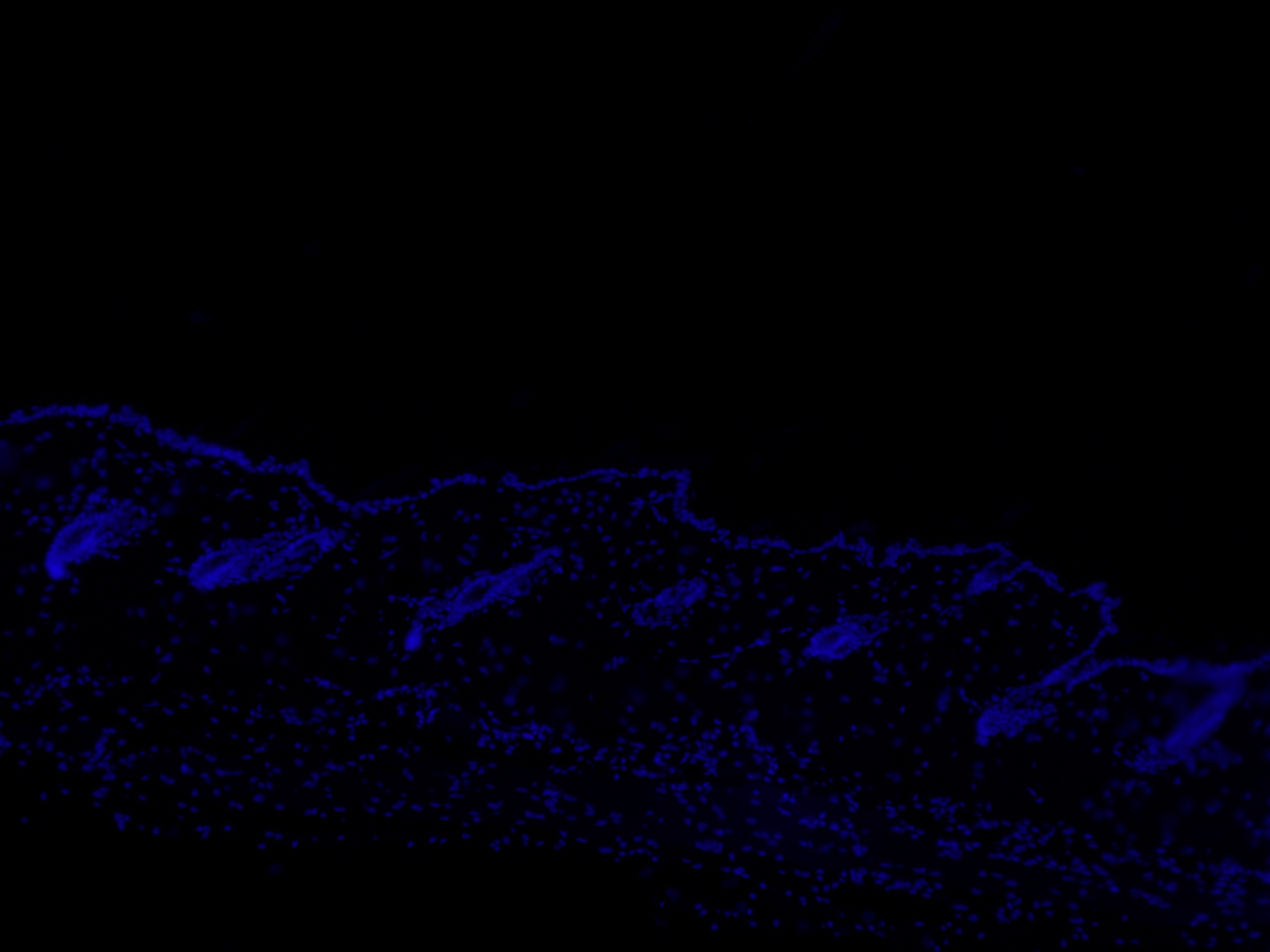

Supplement: Supplementary file 4 — Source data Fig. 3 [file 44319_2024_327_MOESM4_ESM.zip › Figure 3/3A/Telogen/1 (3).tif]

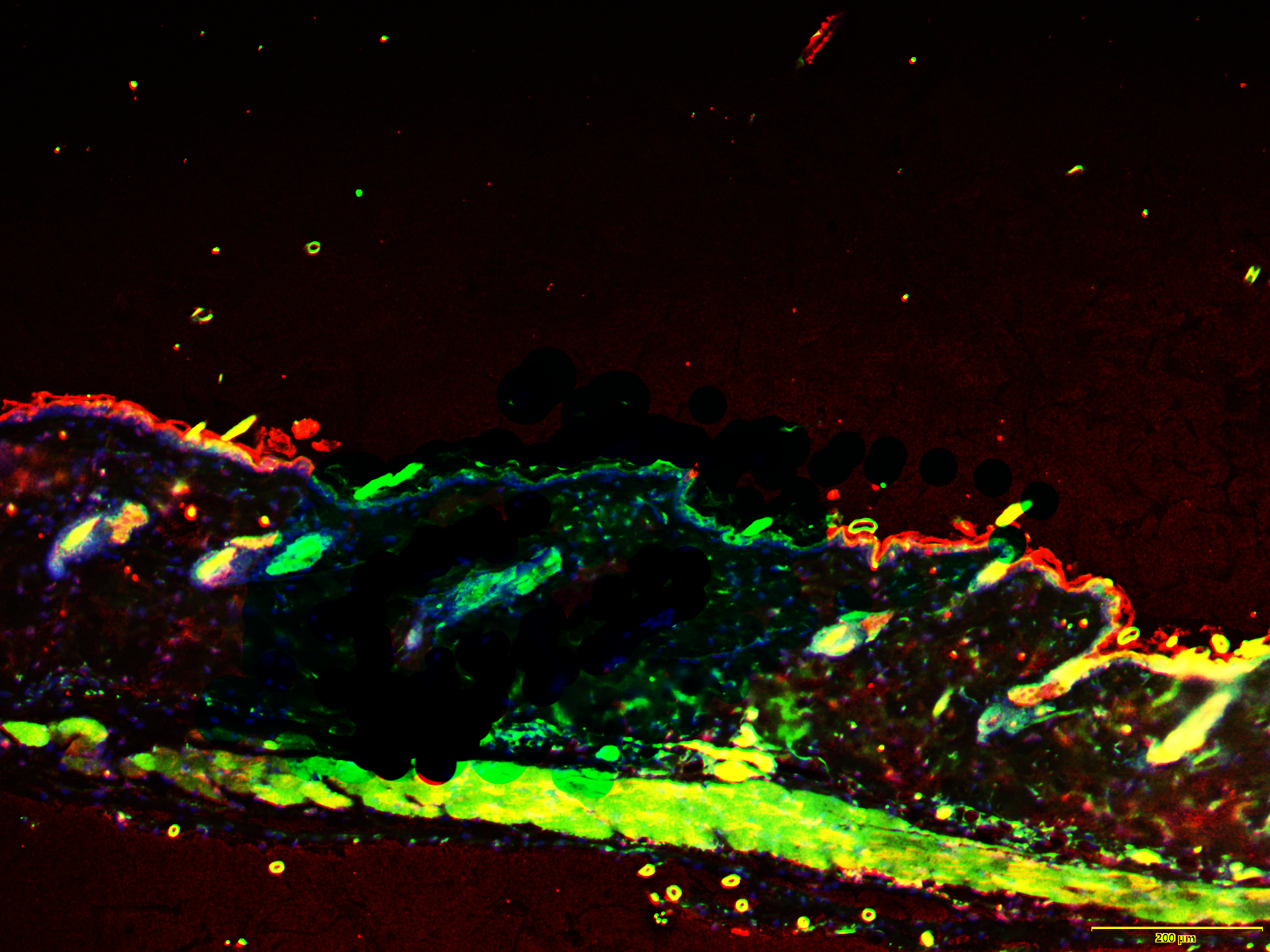

Supplement: Supplementary file 4 — Source data Fig. 3 [file 44319_2024_327_MOESM4_ESM.zip › Figure 3/3A/Telogen/1 (4).tif]

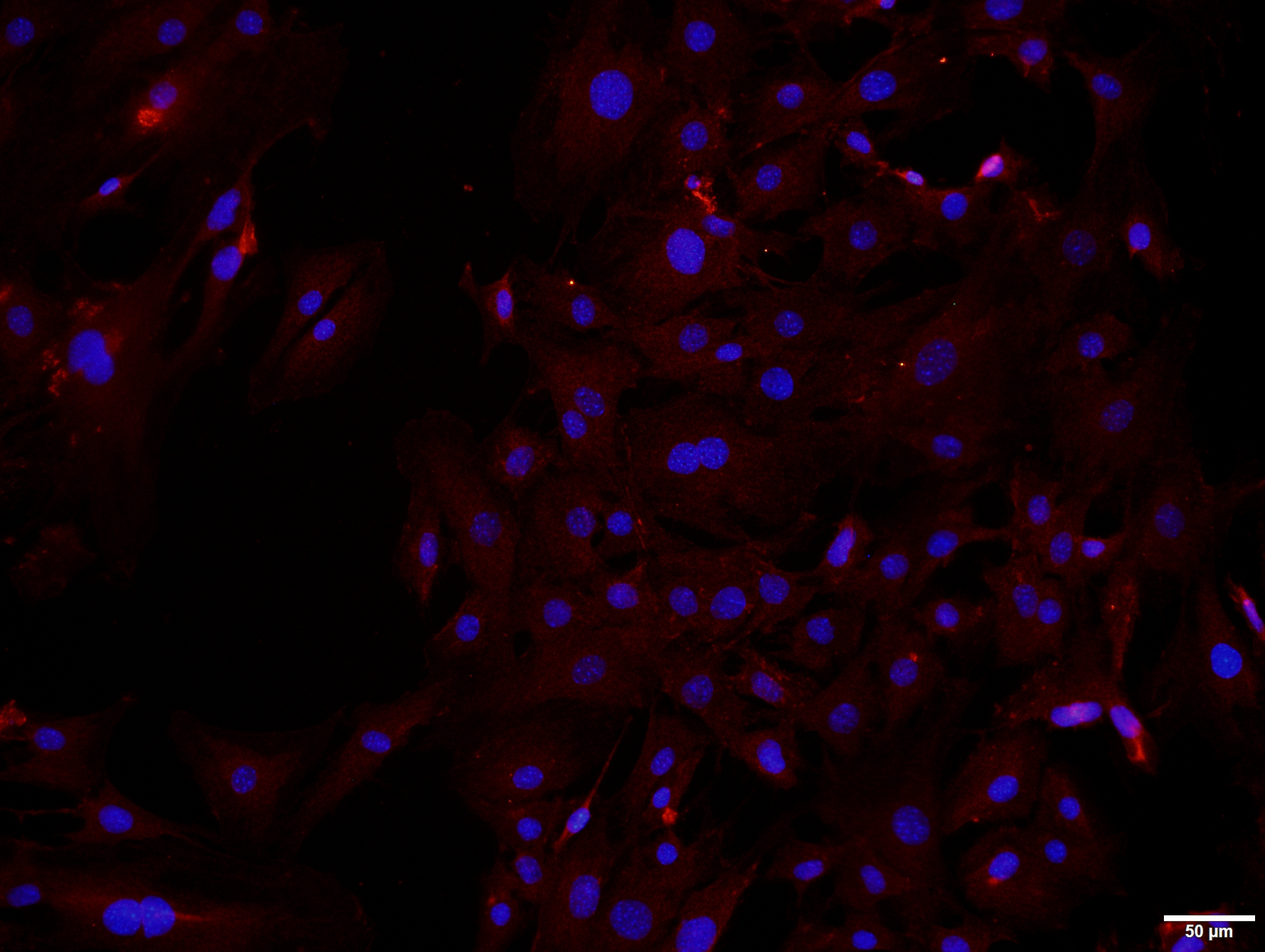

Supplement: Supplementary file 4 — Source data Fig. 3 [file 44319_2024_327_MOESM4_ESM.zip › Figure 3/3B/KO/1 (1).jpg]

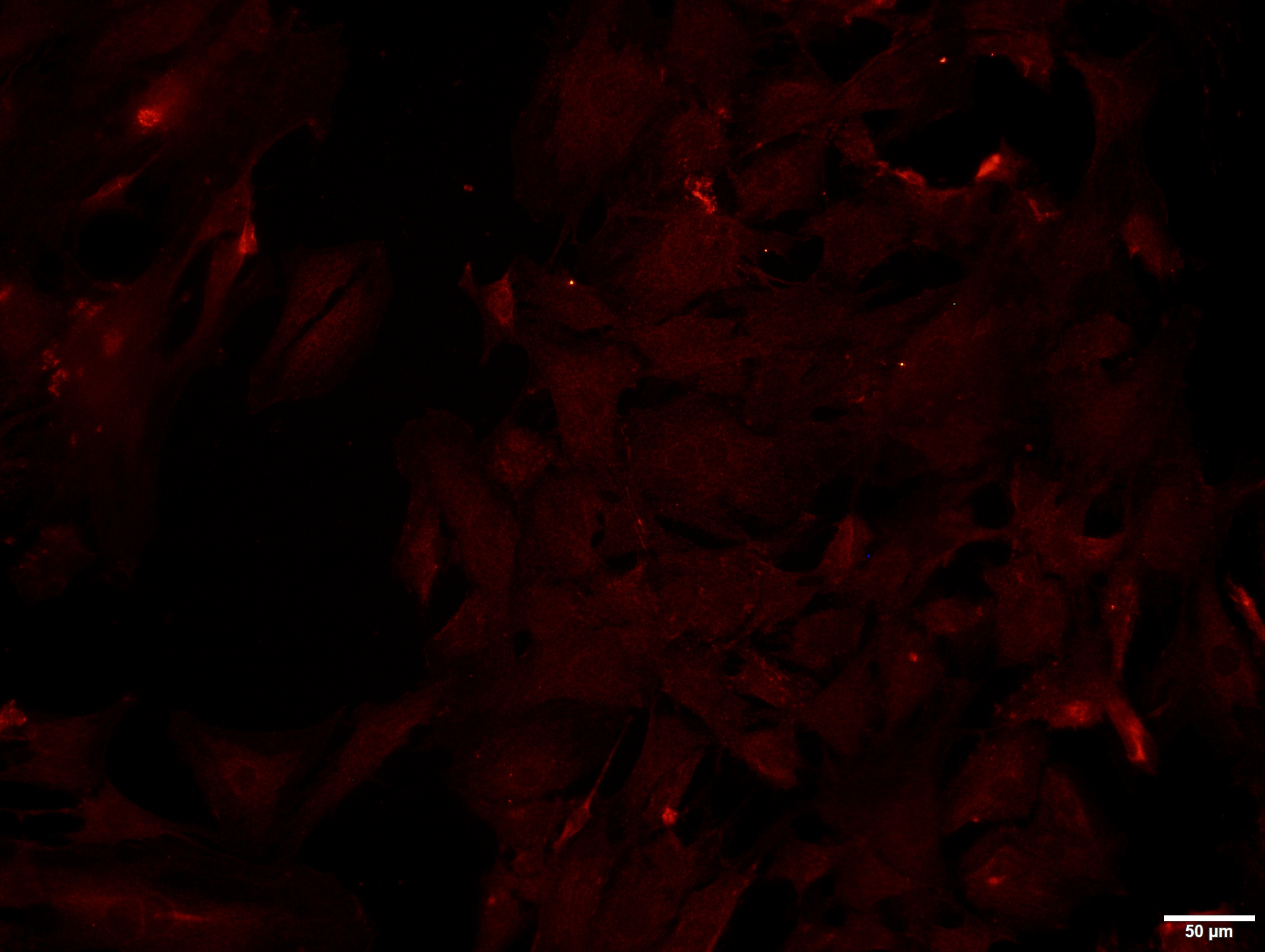

Supplement: Supplementary file 4 — Source data Fig. 3 [file 44319_2024_327_MOESM4_ESM.zip › Figure 3/3B/KO/1 (2).jpg]

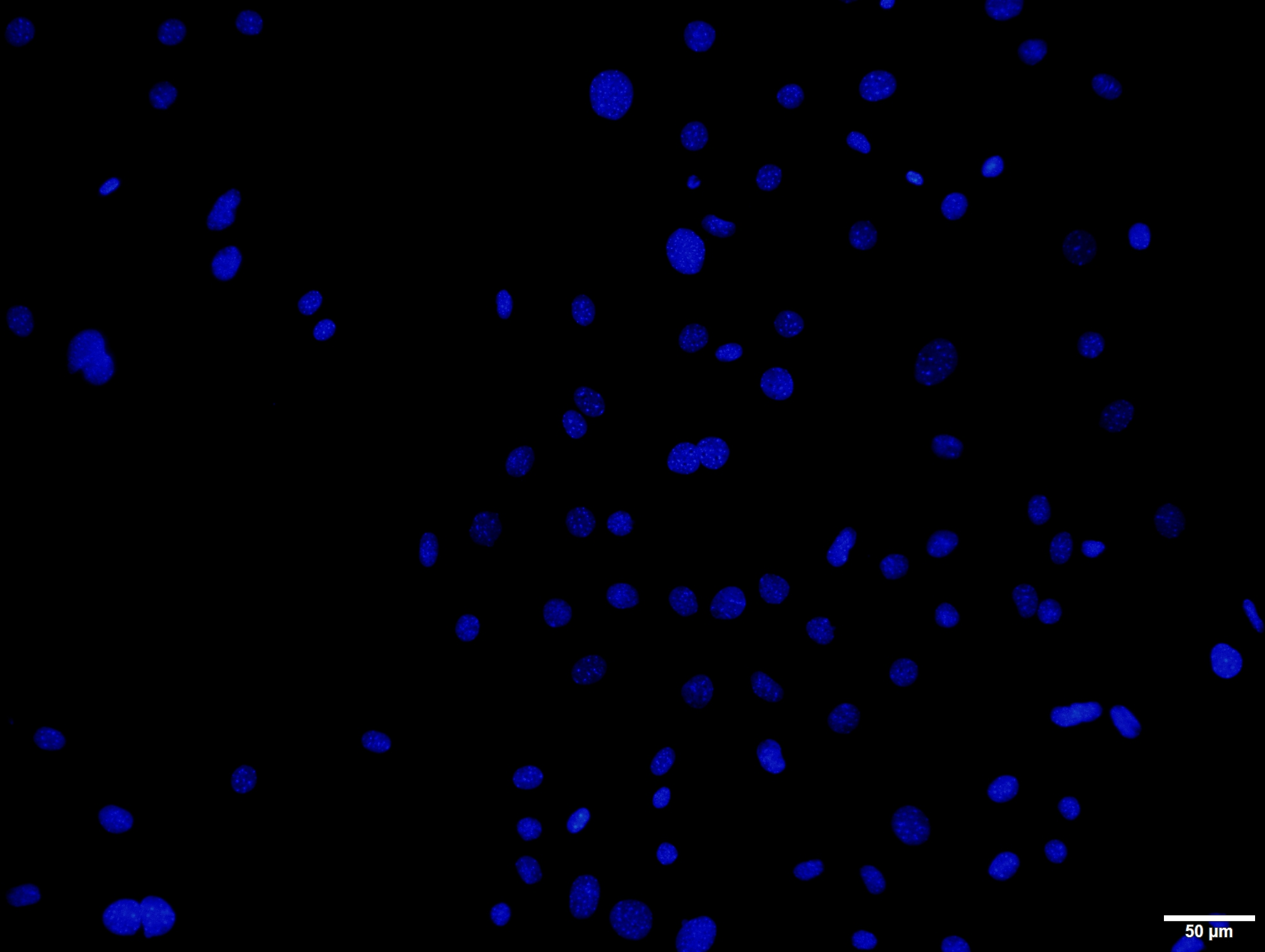

Supplement: Supplementary file 4 — Source data Fig. 3 [file 44319_2024_327_MOESM4_ESM.zip › Figure 3/3B/KO/1 (3).jpg]

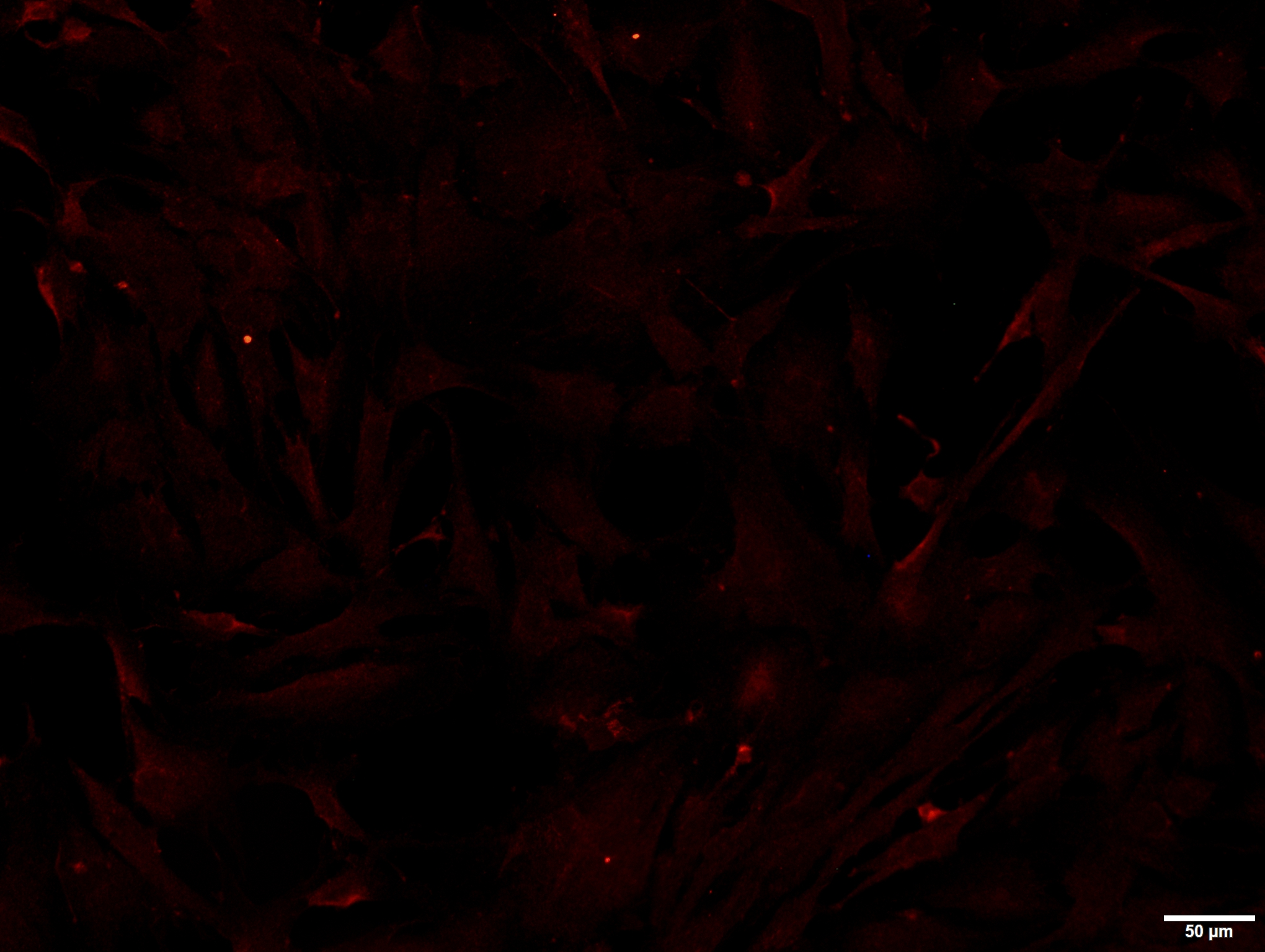

Supplement: Supplementary file 4 — Source data Fig. 3 [file 44319_2024_327_MOESM4_ESM.zip › Figure 3/3B/WT/1 (1).jpg]

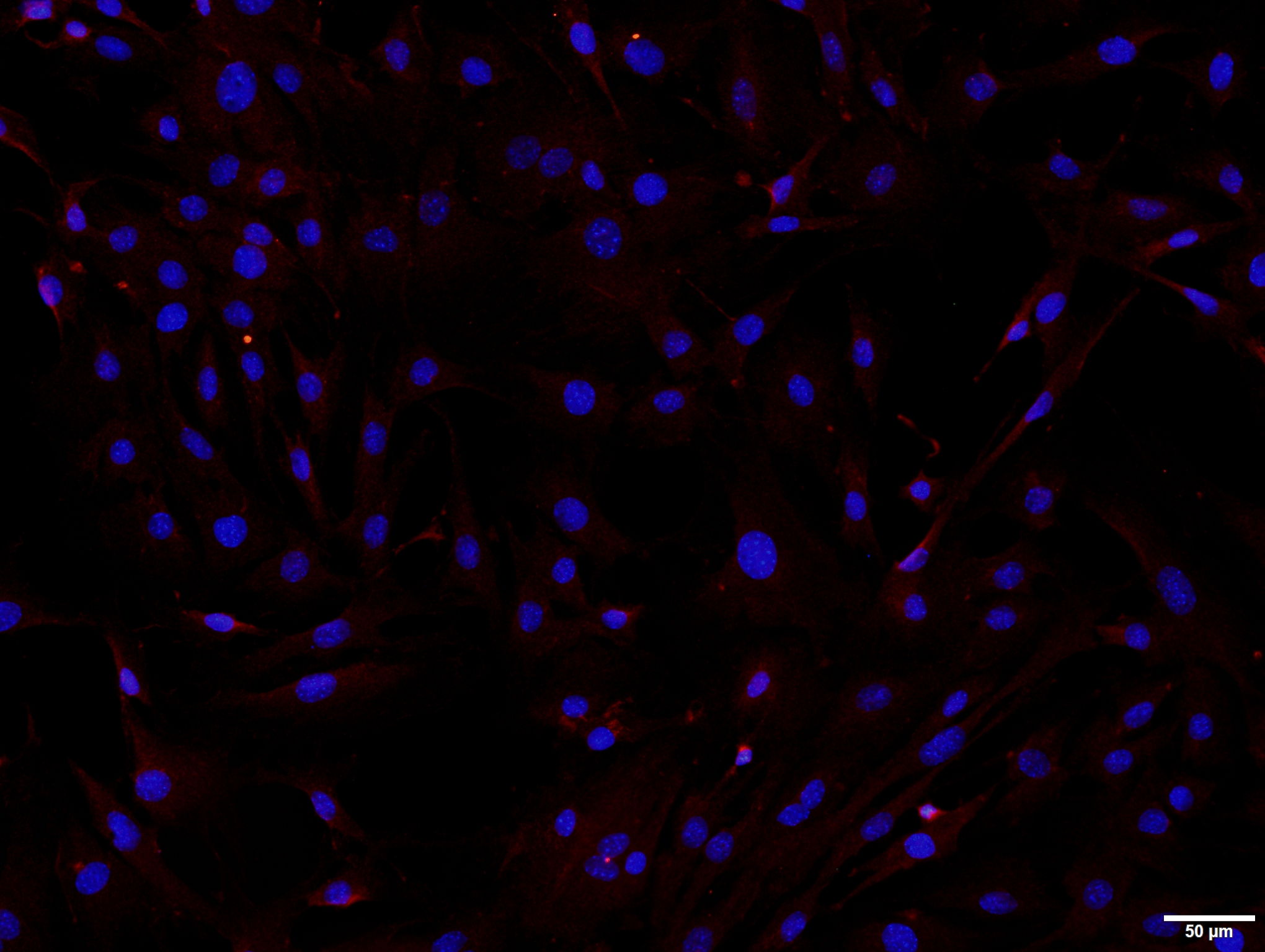

Supplement: Supplementary file 4 — Source data Fig. 3 [file 44319_2024_327_MOESM4_ESM.zip › Figure 3/3B/WT/1 (2).jpg]

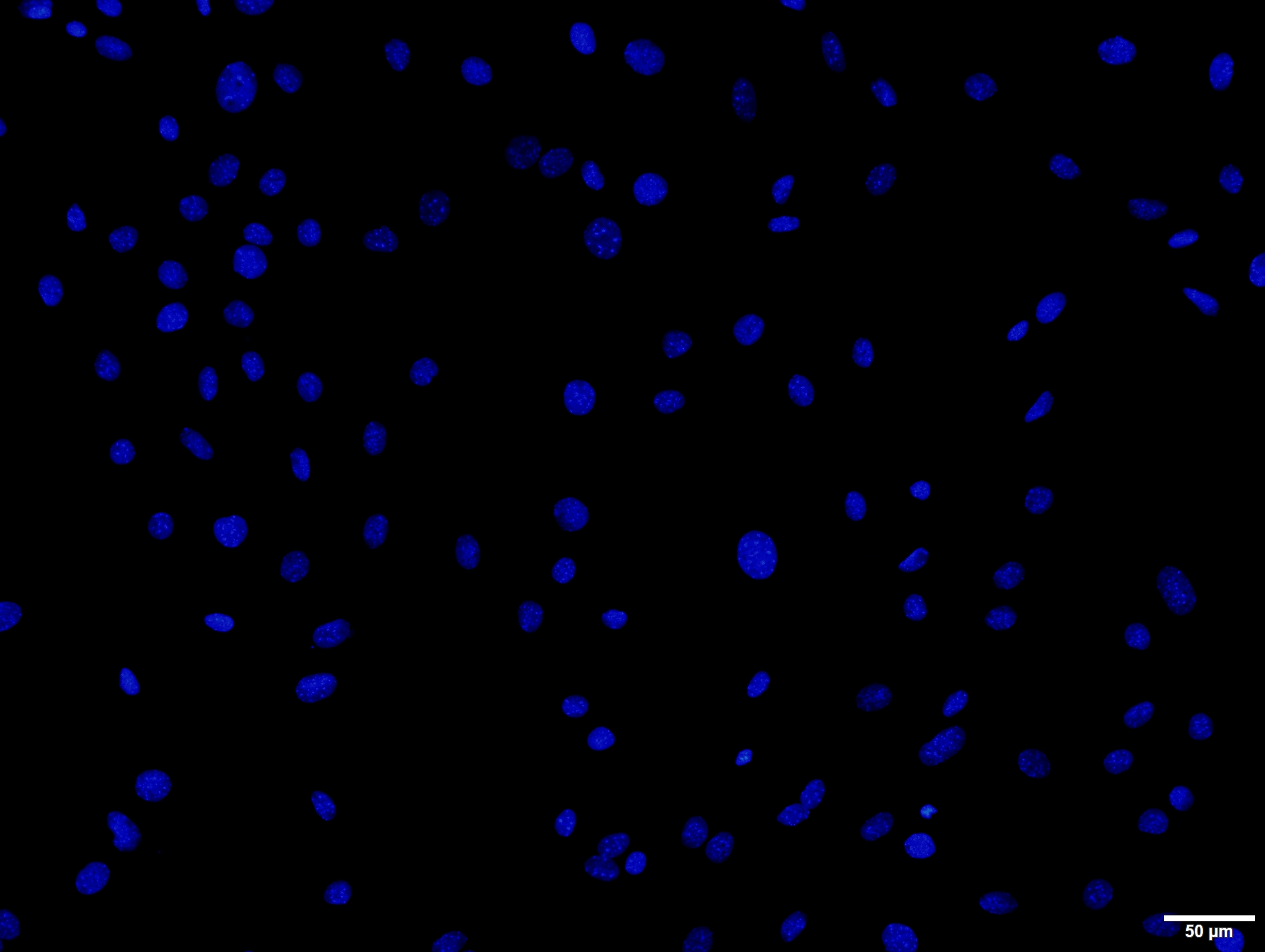

Supplement: Supplementary file 4 — Source data Fig. 3 [file 44319_2024_327_MOESM4_ESM.zip › Figure 3/3B/WT/1 (3).jpg]

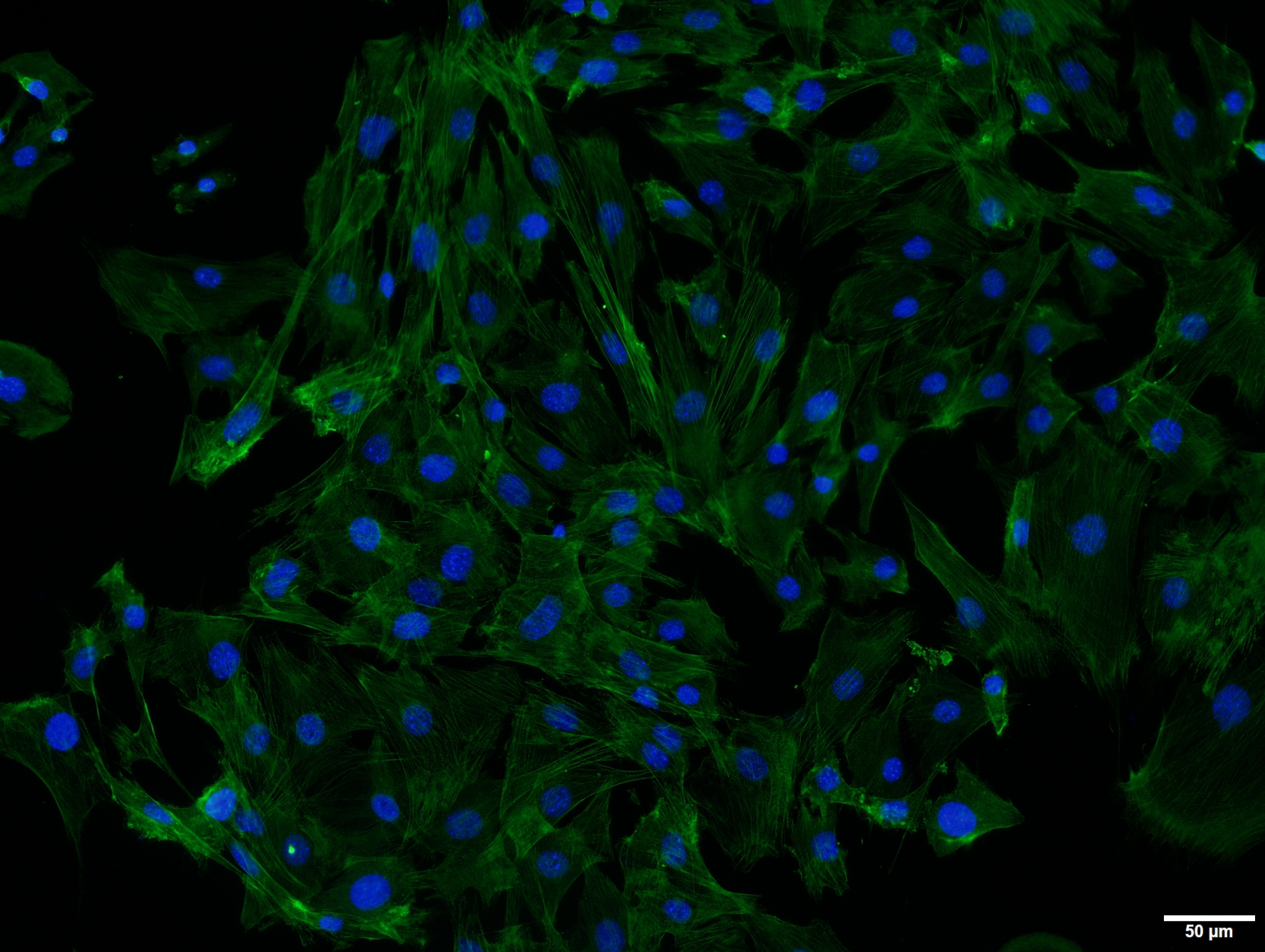

Supplement: Supplementary file 4 — Source data Fig. 3 [file 44319_2024_327_MOESM4_ESM.zip › Figure 3/3C/KO/1 (1).jpg]

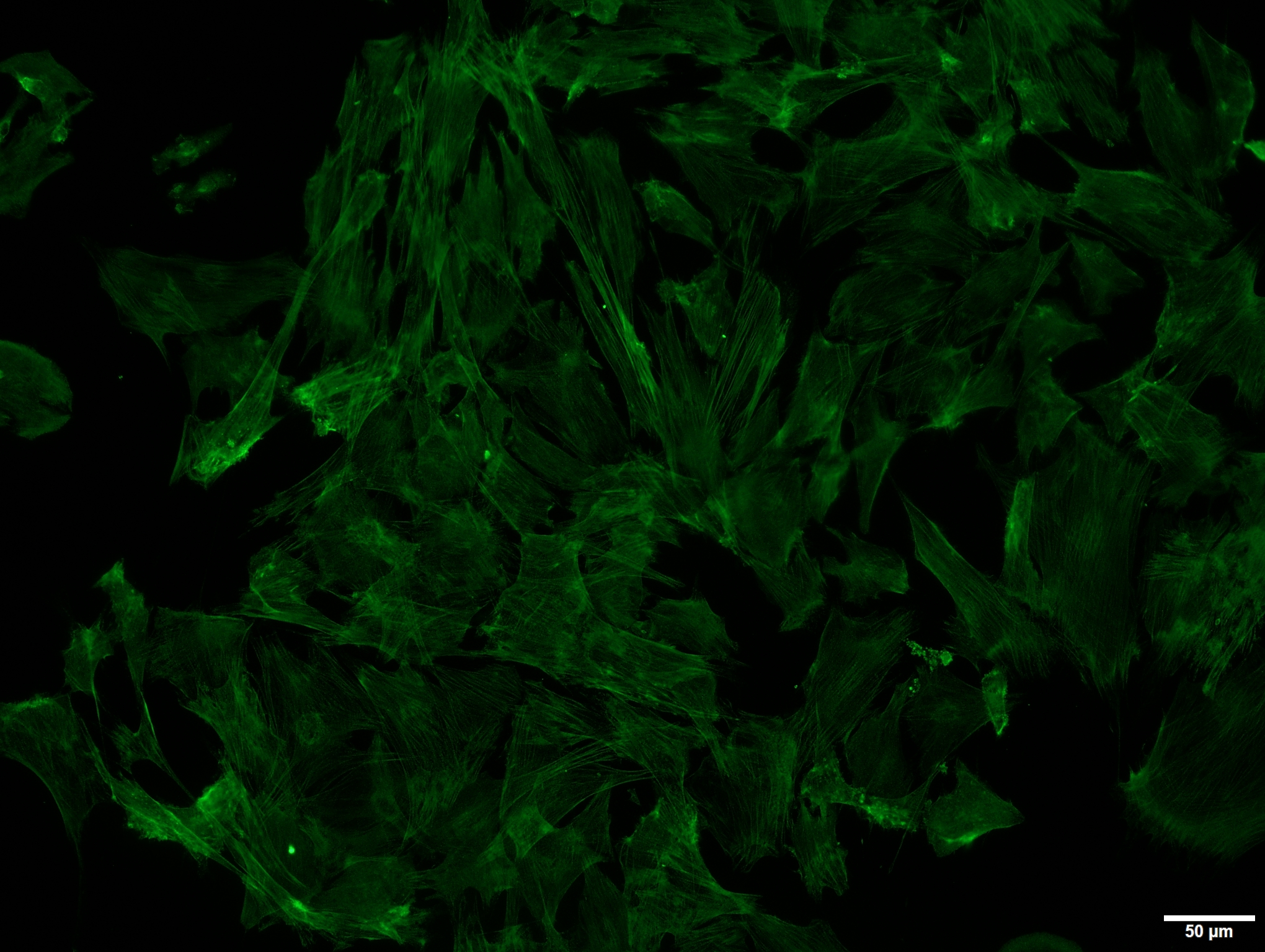

Supplement: Supplementary file 4 — Source data Fig. 3 [file 44319_2024_327_MOESM4_ESM.zip › Figure 3/3C/KO/1 (2).jpg]

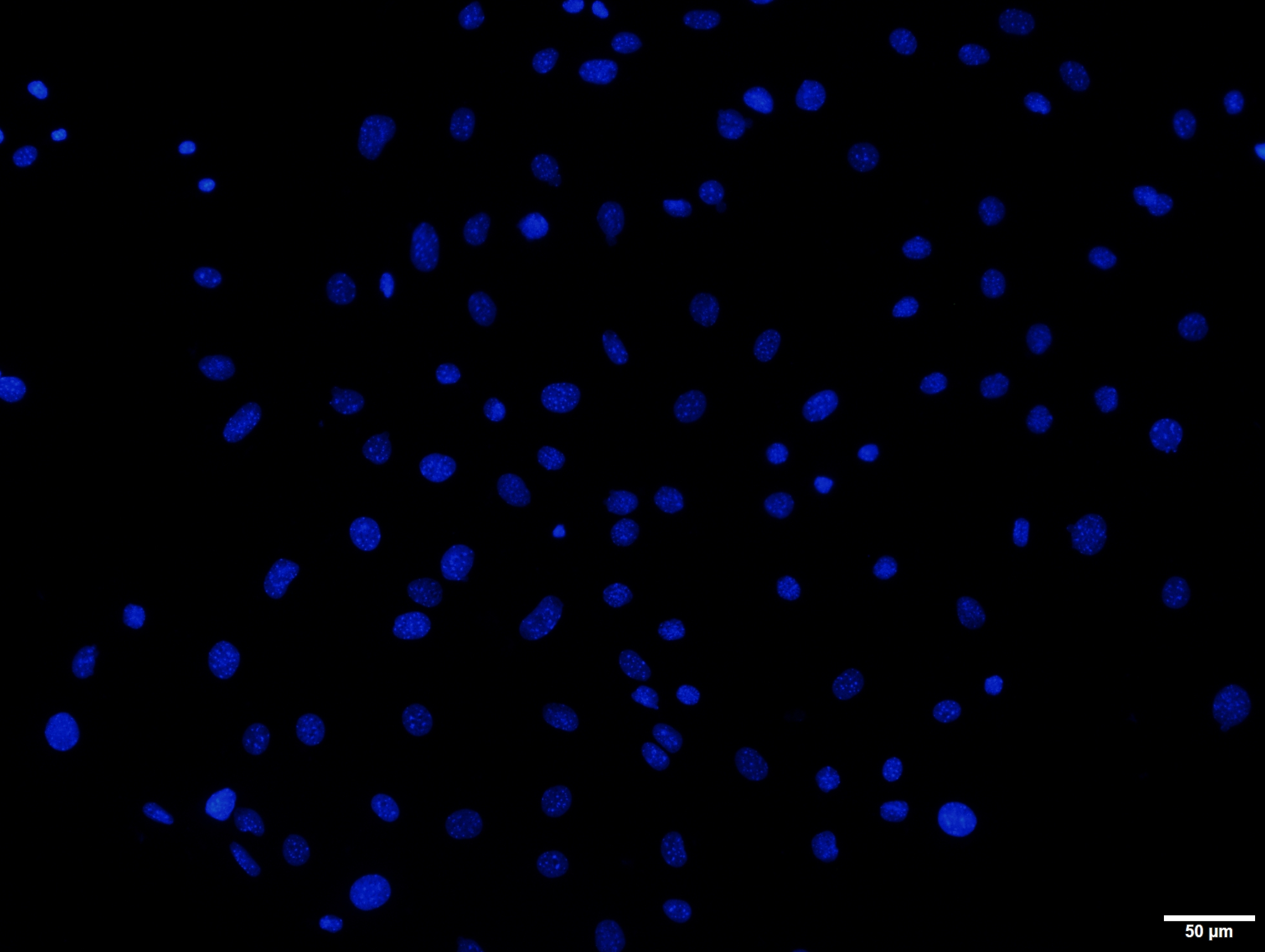

Supplement: Supplementary file 4 — Source data Fig. 3 [file 44319_2024_327_MOESM4_ESM.zip › Figure 3/3C/KO/1 (3).jpg]

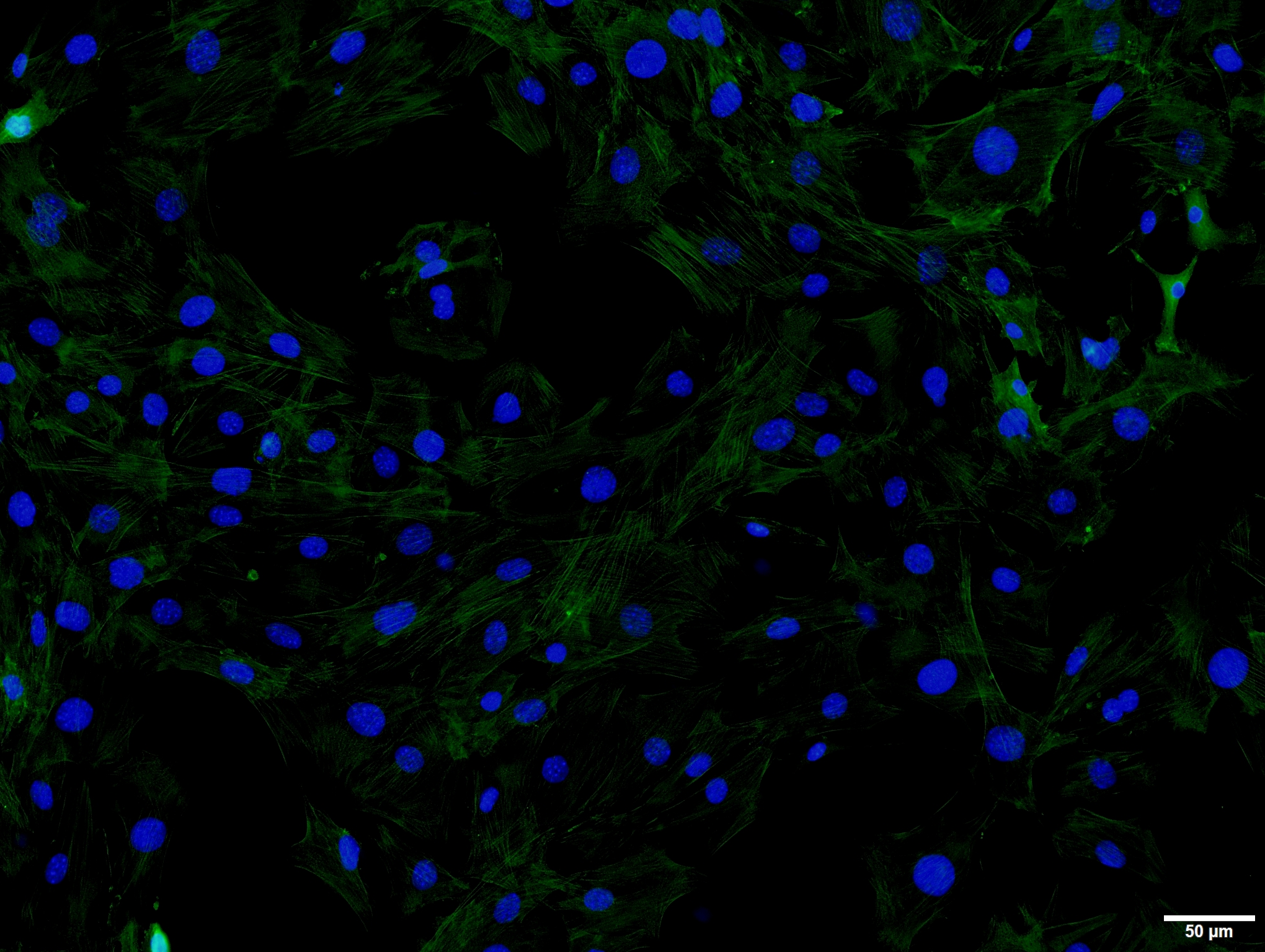

Supplement: Supplementary file 4 — Source data Fig. 3 [file 44319_2024_327_MOESM4_ESM.zip › Figure 3/3C/WT/1 (1).jpg]

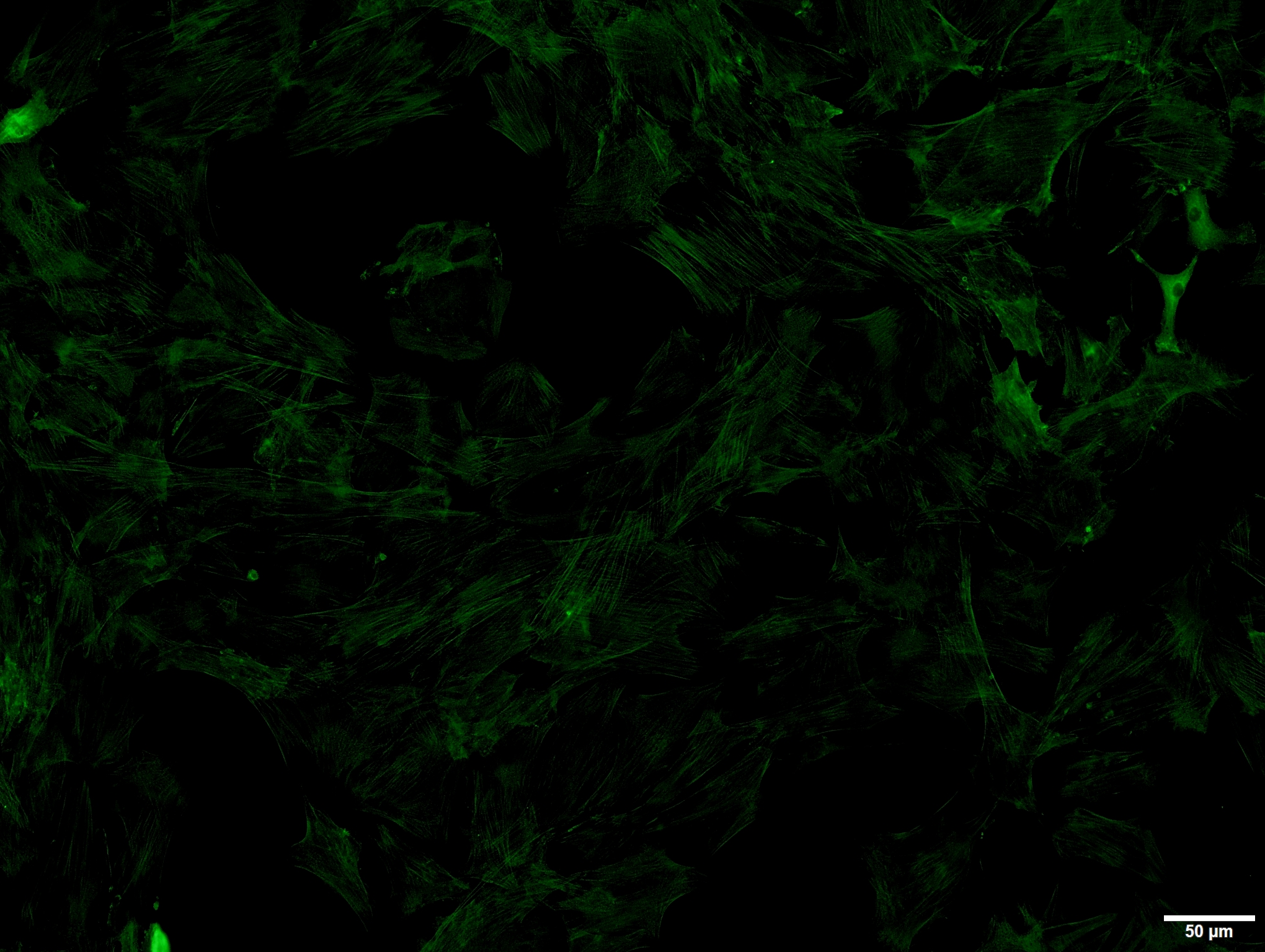

Supplement: Supplementary file 4 — Source data Fig. 3 [file 44319_2024_327_MOESM4_ESM.zip › Figure 3/3C/WT/1 (2).jpg]

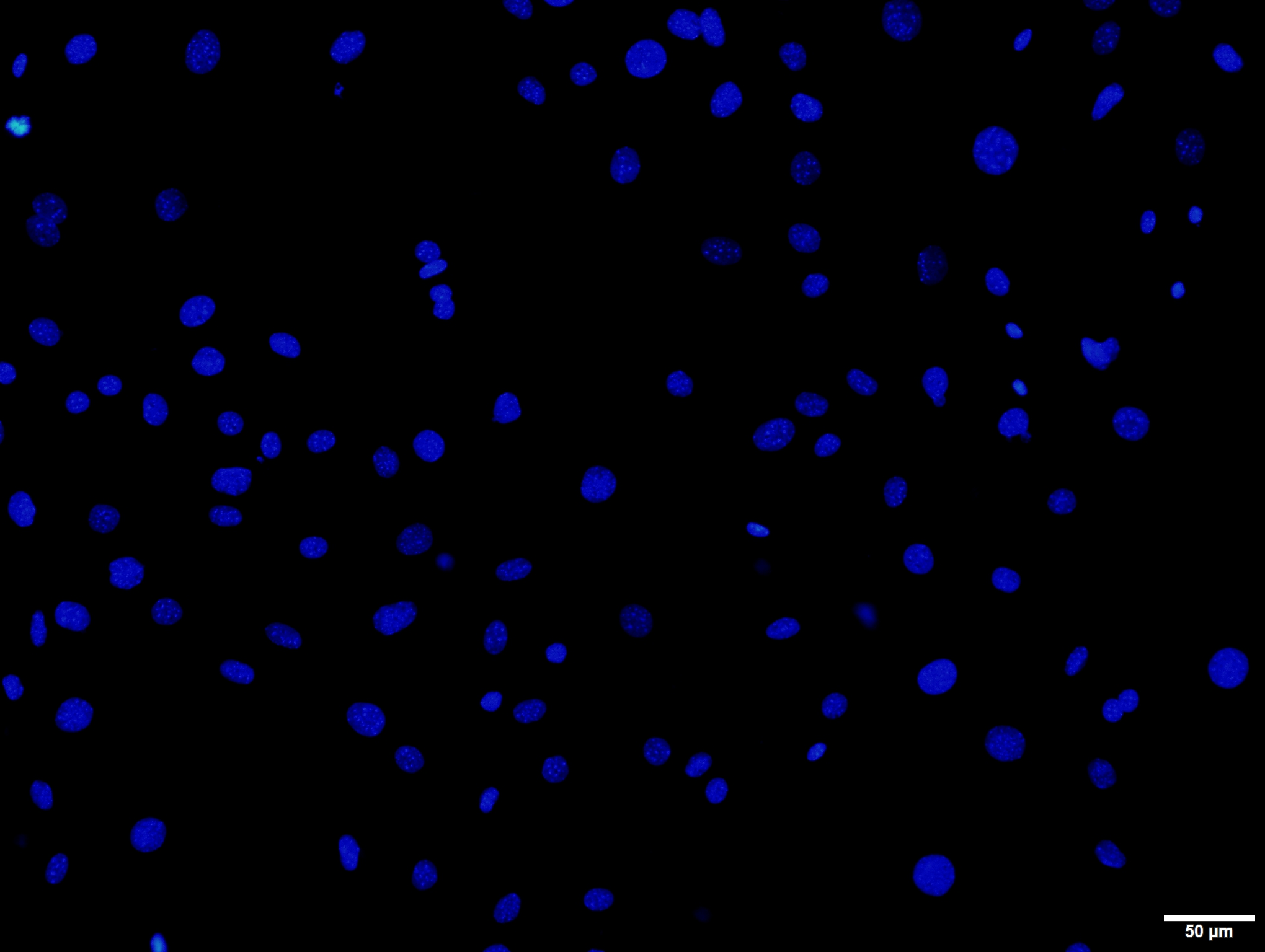

Supplement: Supplementary file 4 — Source data Fig. 3 [file 44319_2024_327_MOESM4_ESM.zip › Figure 3/3C/WT/1 (3).jpg]

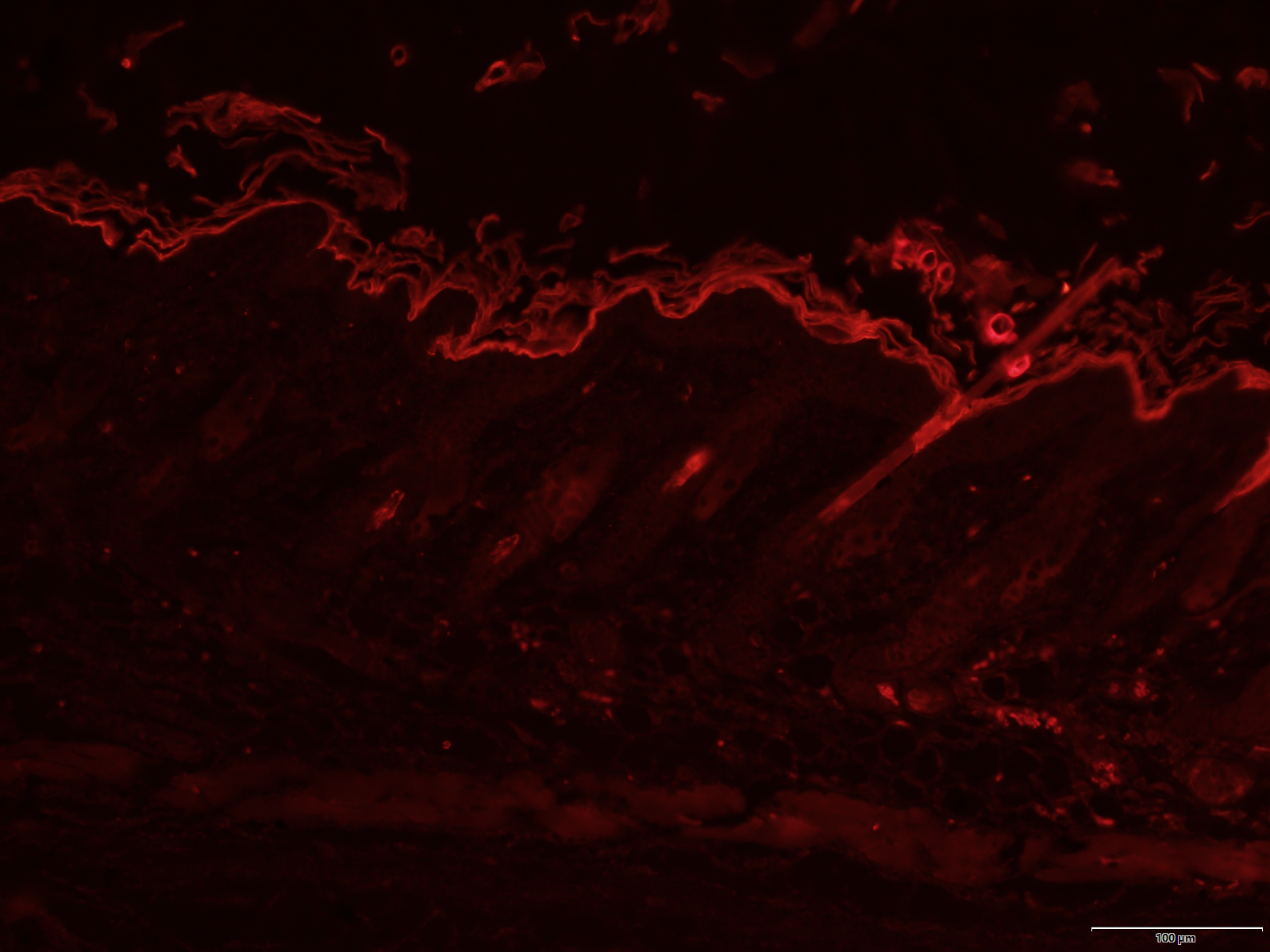

Supplement: Supplementary file 5 — Source data Fig. 4 [file 44319_2024_327_MOESM5_ESM.zip › Figure 4/4B/KO/1 (1).tif]

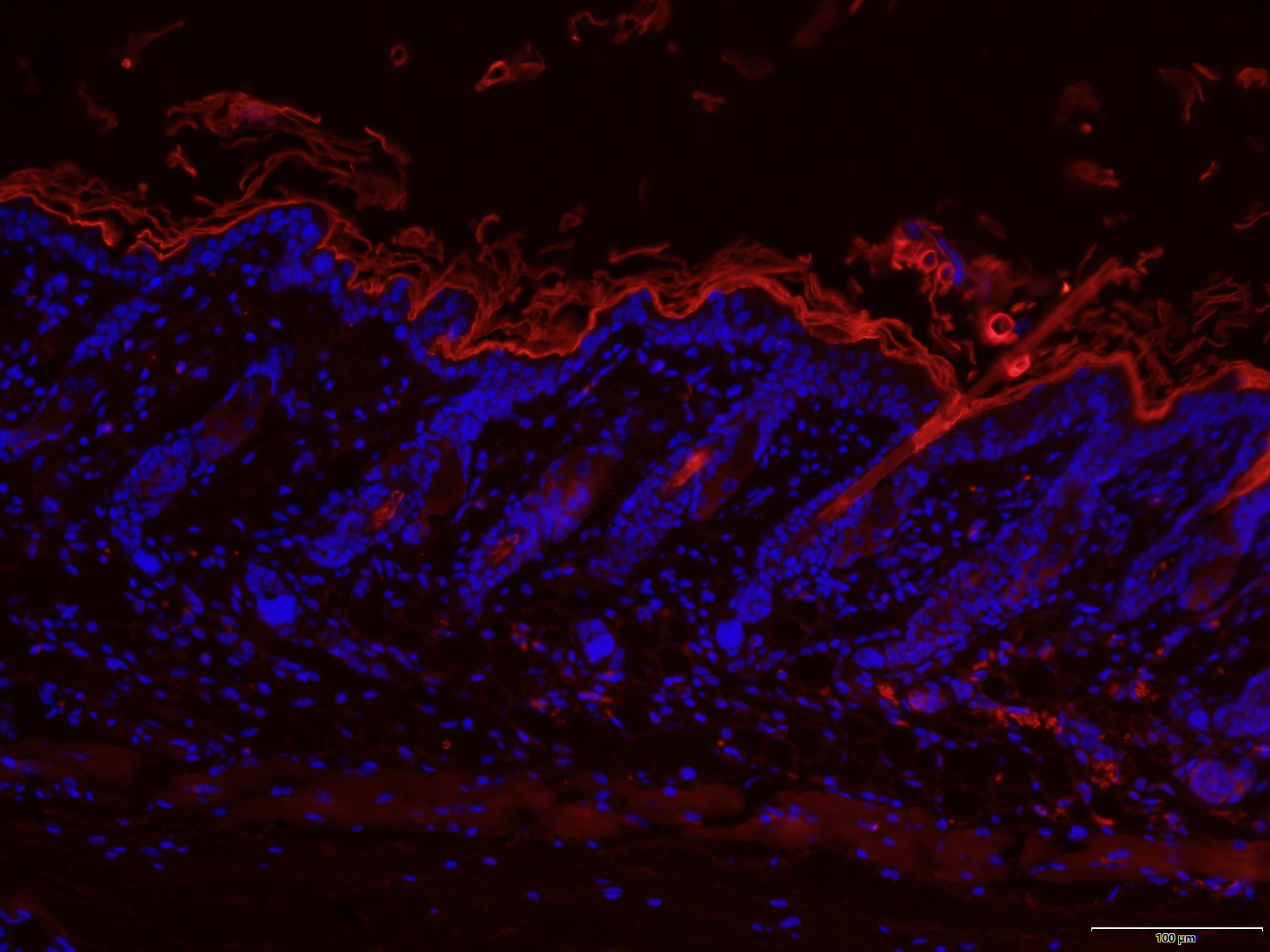

Supplement: Supplementary file 5 — Source data Fig. 4 [file 44319_2024_327_MOESM5_ESM.zip › Figure 4/4B/KO/1 (3).tif]

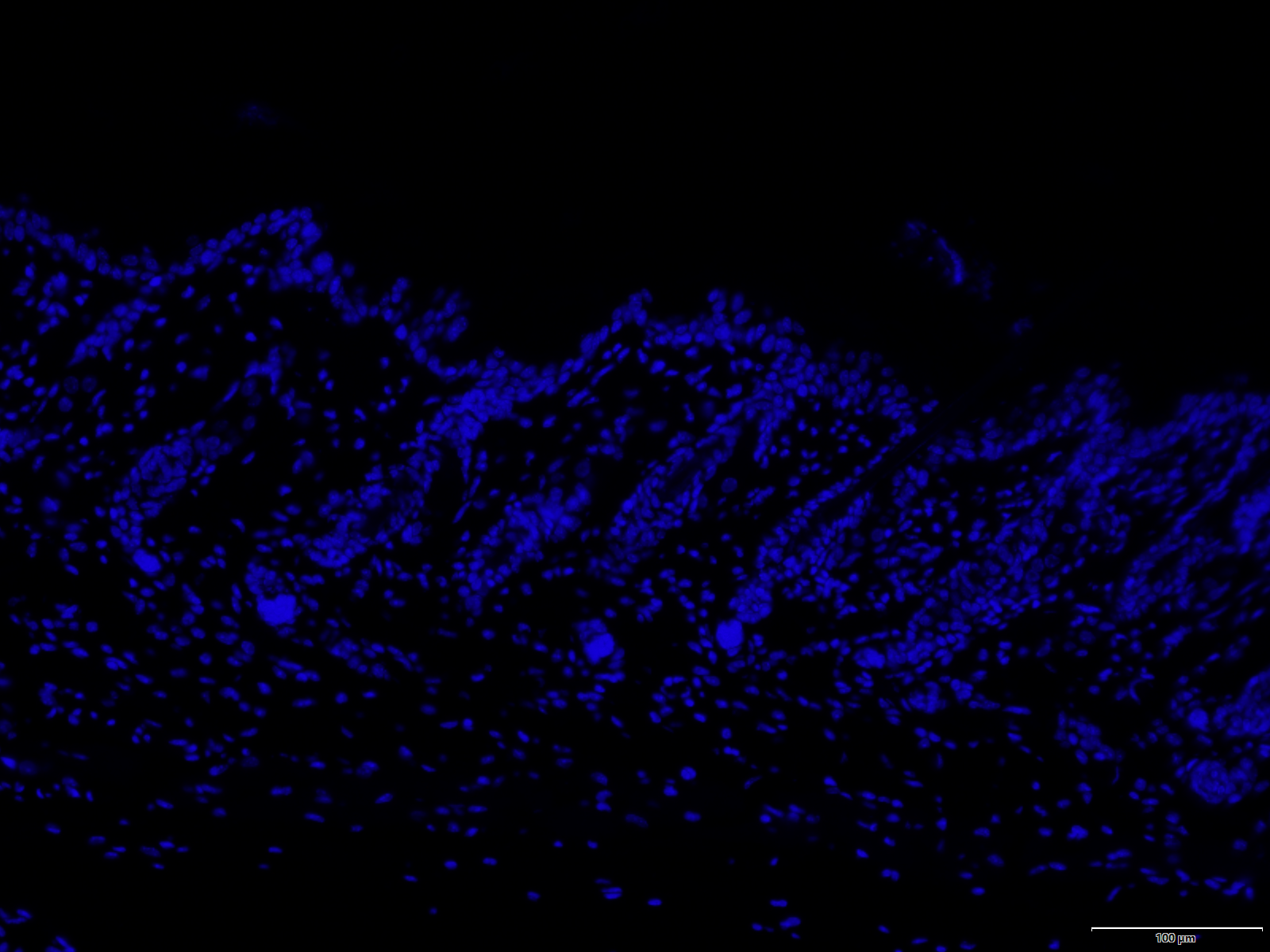

Supplement: Supplementary file 5 — Source data Fig. 4 [file 44319_2024_327_MOESM5_ESM.zip › Figure 4/4B/KO/1 (4).tif]

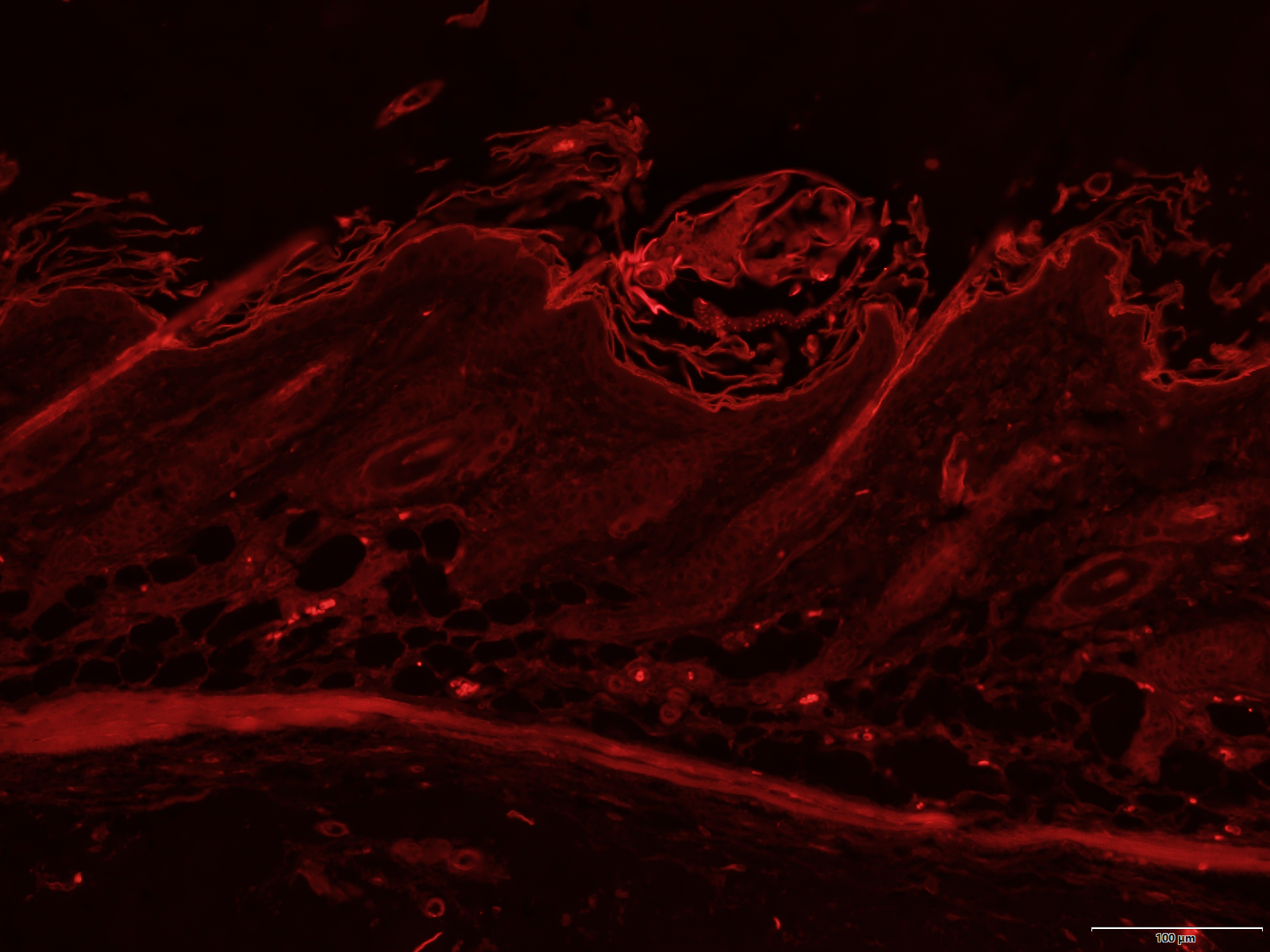

Supplement: Supplementary file 5 — Source data Fig. 4 [file 44319_2024_327_MOESM5_ESM.zip › Figure 4/4B/WT/1 (1).tif]

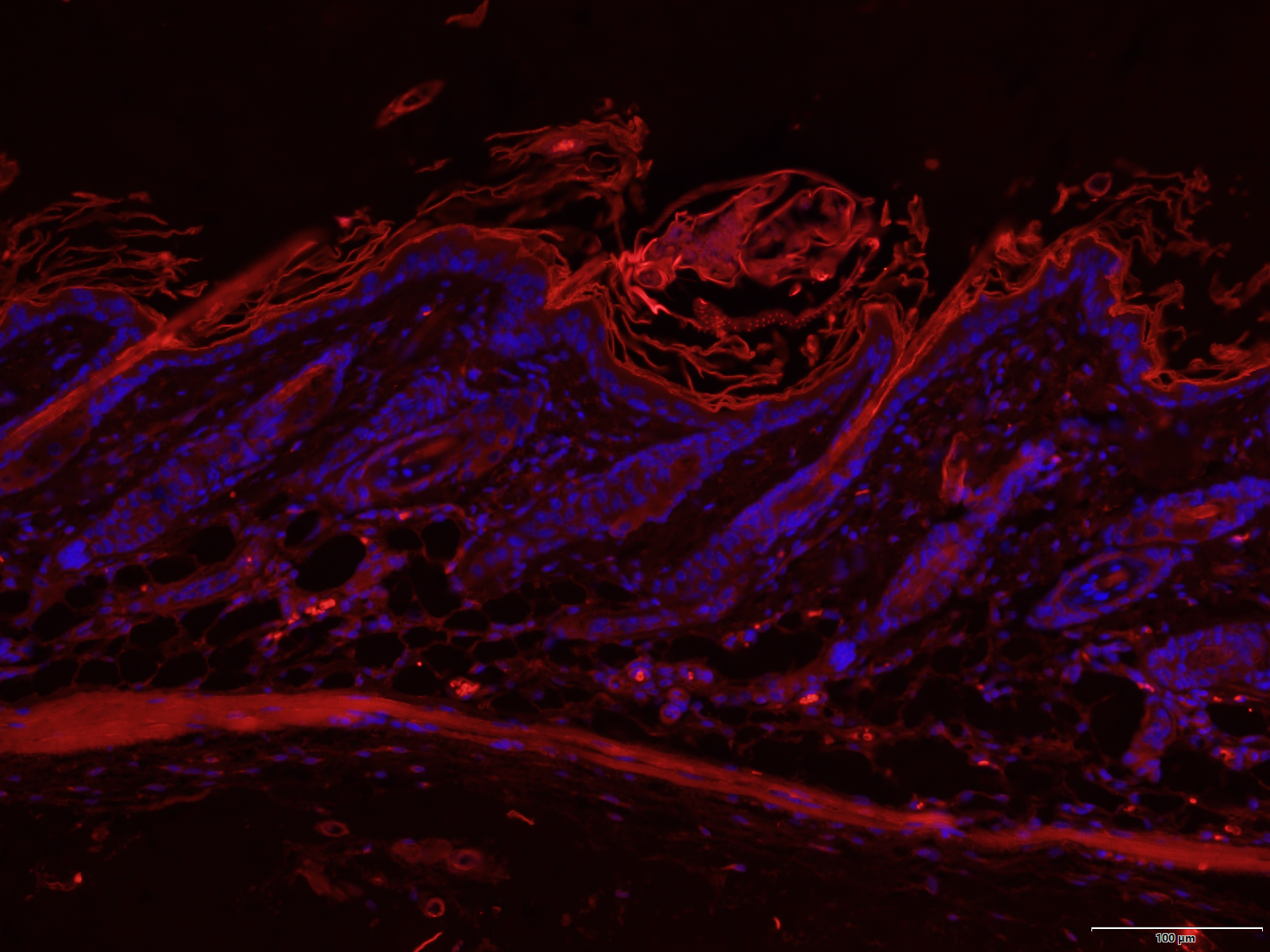

Supplement: Supplementary file 5 — Source data Fig. 4 [file 44319_2024_327_MOESM5_ESM.zip › Figure 4/4B/WT/1 (2).tif]

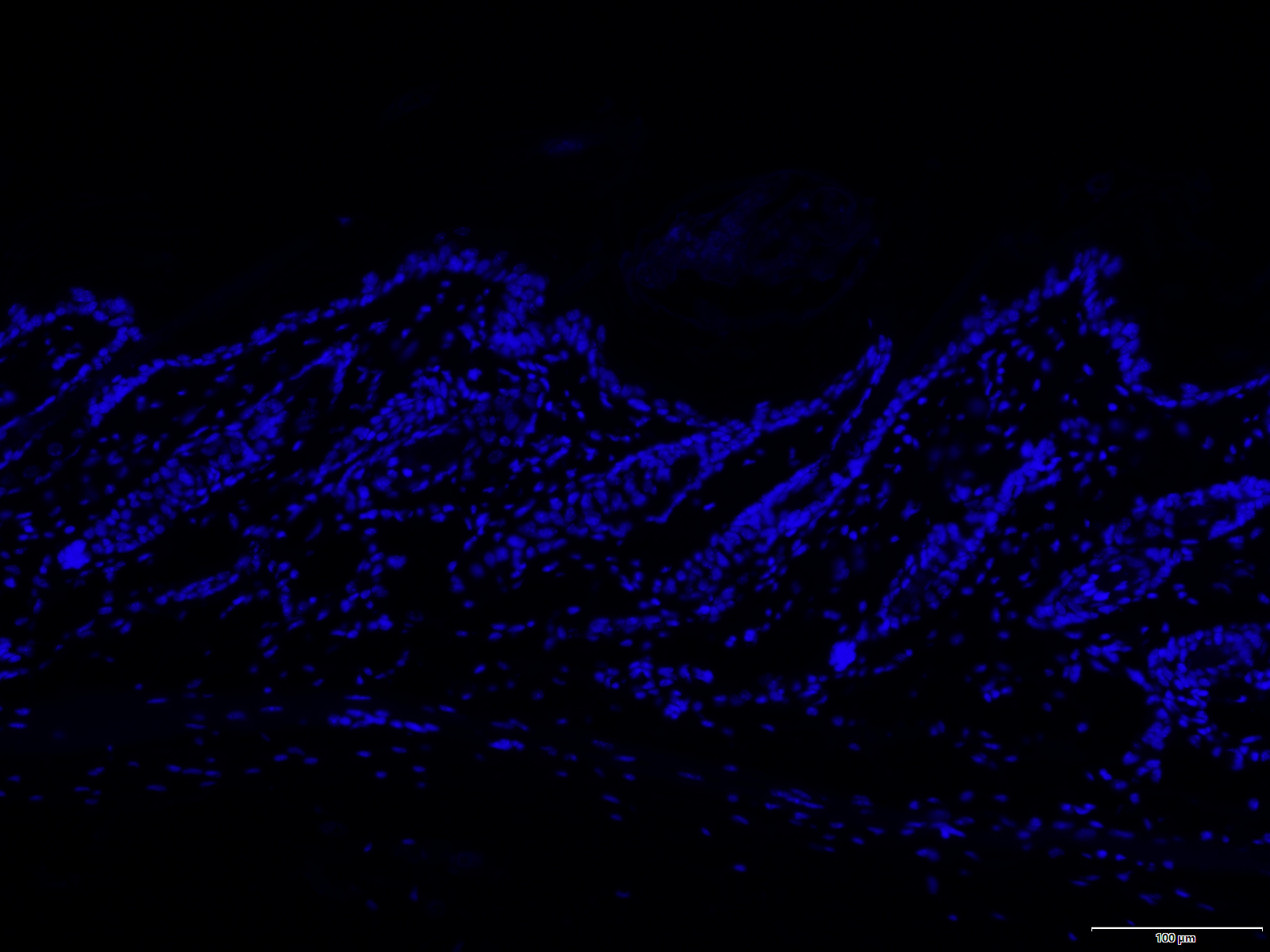

Supplement: Supplementary file 5 — Source data Fig. 4 [file 44319_2024_327_MOESM5_ESM.zip › Figure 4/4B/WT/1 (3).tif]

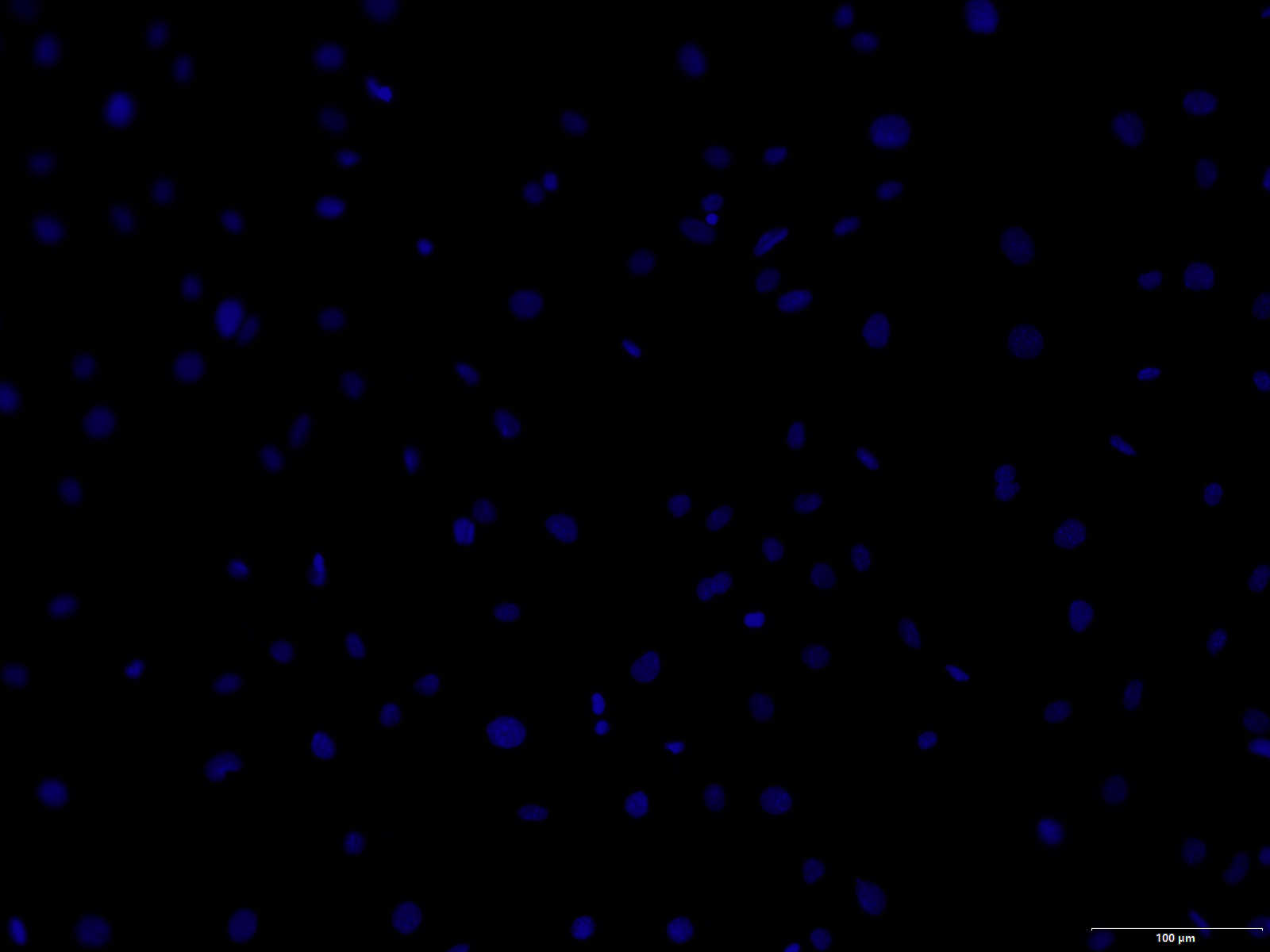

Supplement: Supplementary file 5 — Source data Fig. 4 [file 44319_2024_327_MOESM5_ESM.zip › Figure 4/4F/KO/1 (1).tif]

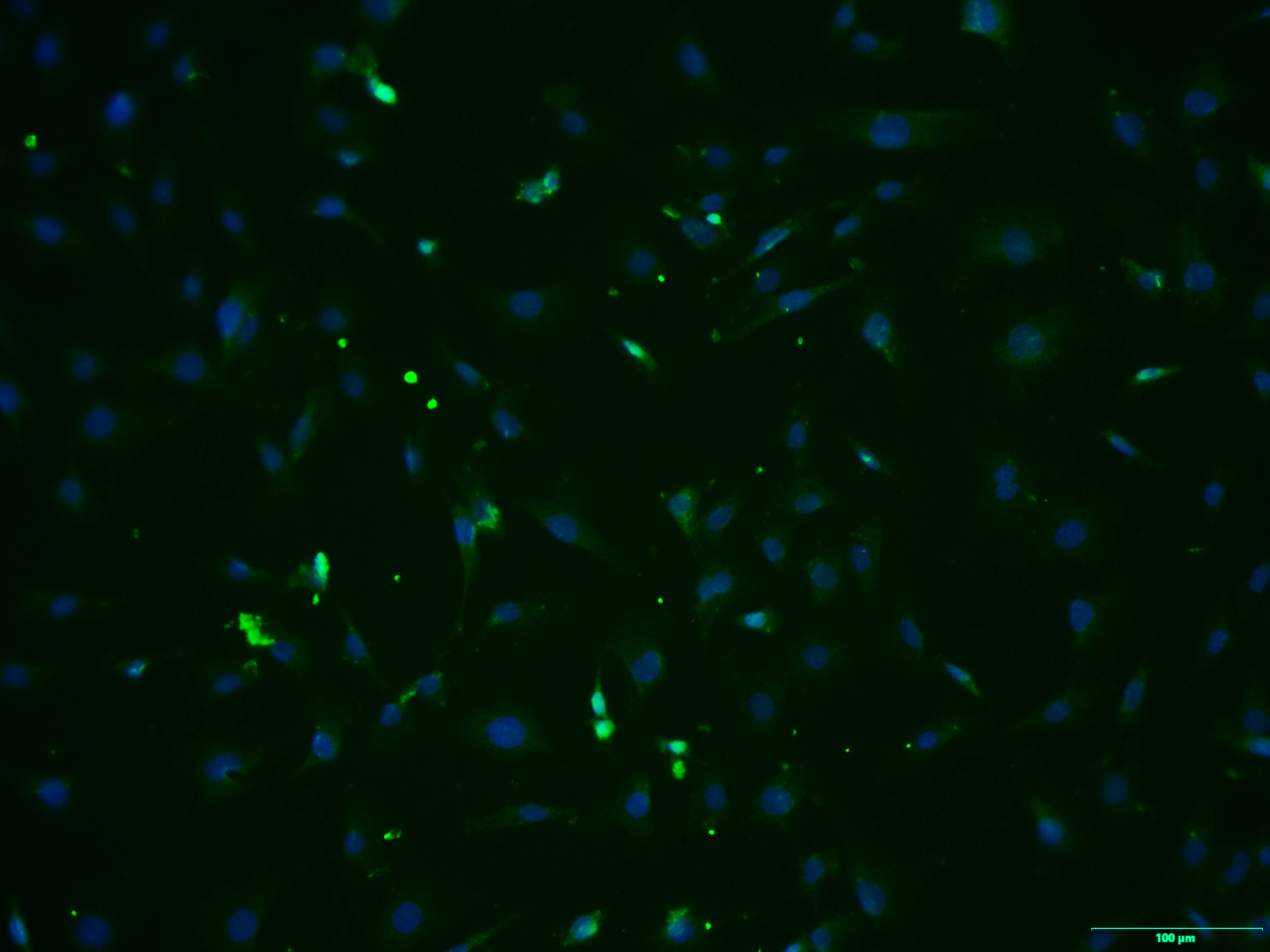

Supplement: Supplementary file 5 — Source data Fig. 4 [file 44319_2024_327_MOESM5_ESM.zip › Figure 4/4F/KO/1 (2).tif]

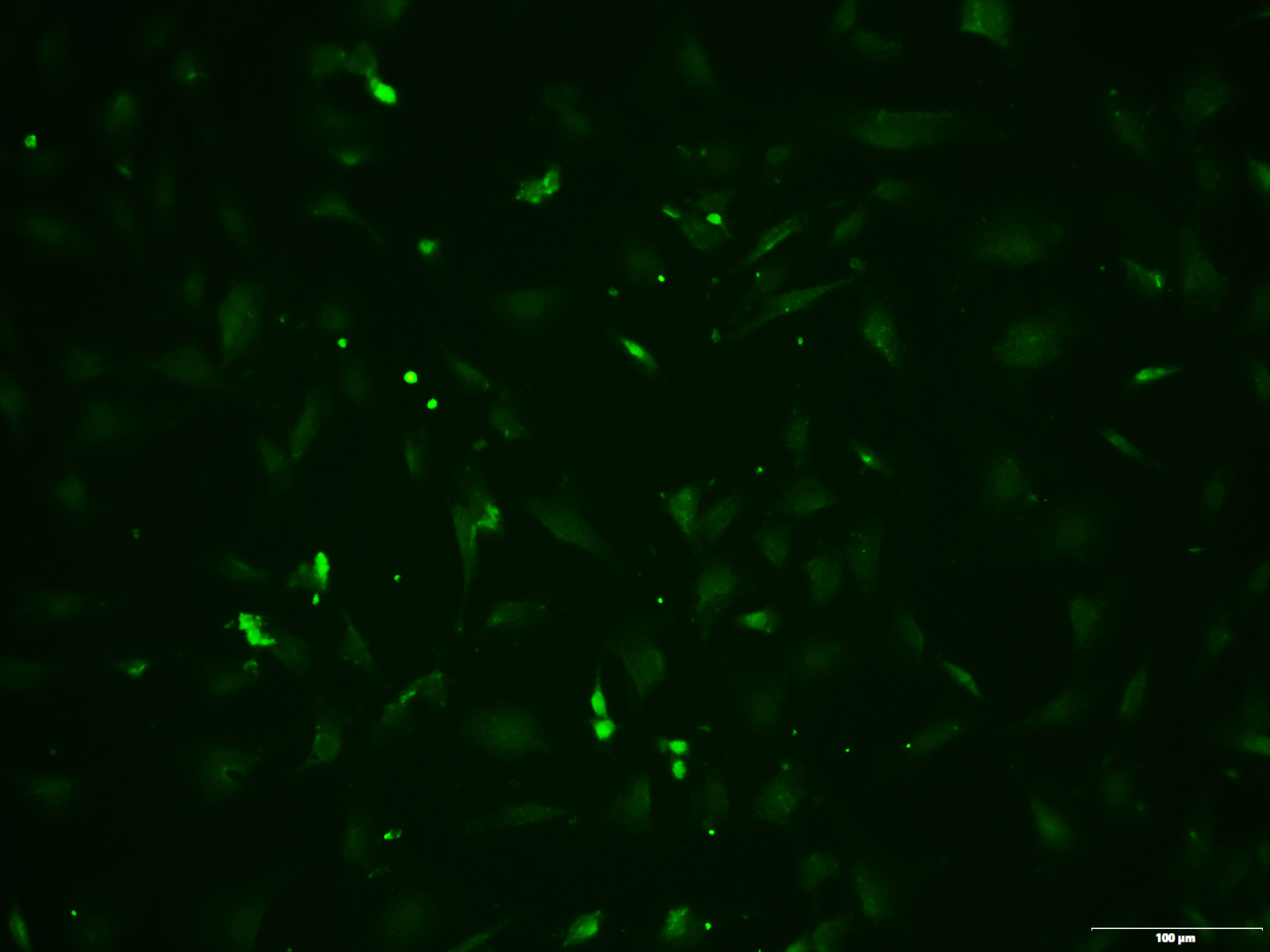

Supplement: Supplementary file 5 — Source data Fig. 4 [file 44319_2024_327_MOESM5_ESM.zip › Figure 4/4F/KO/1 (3).tif]

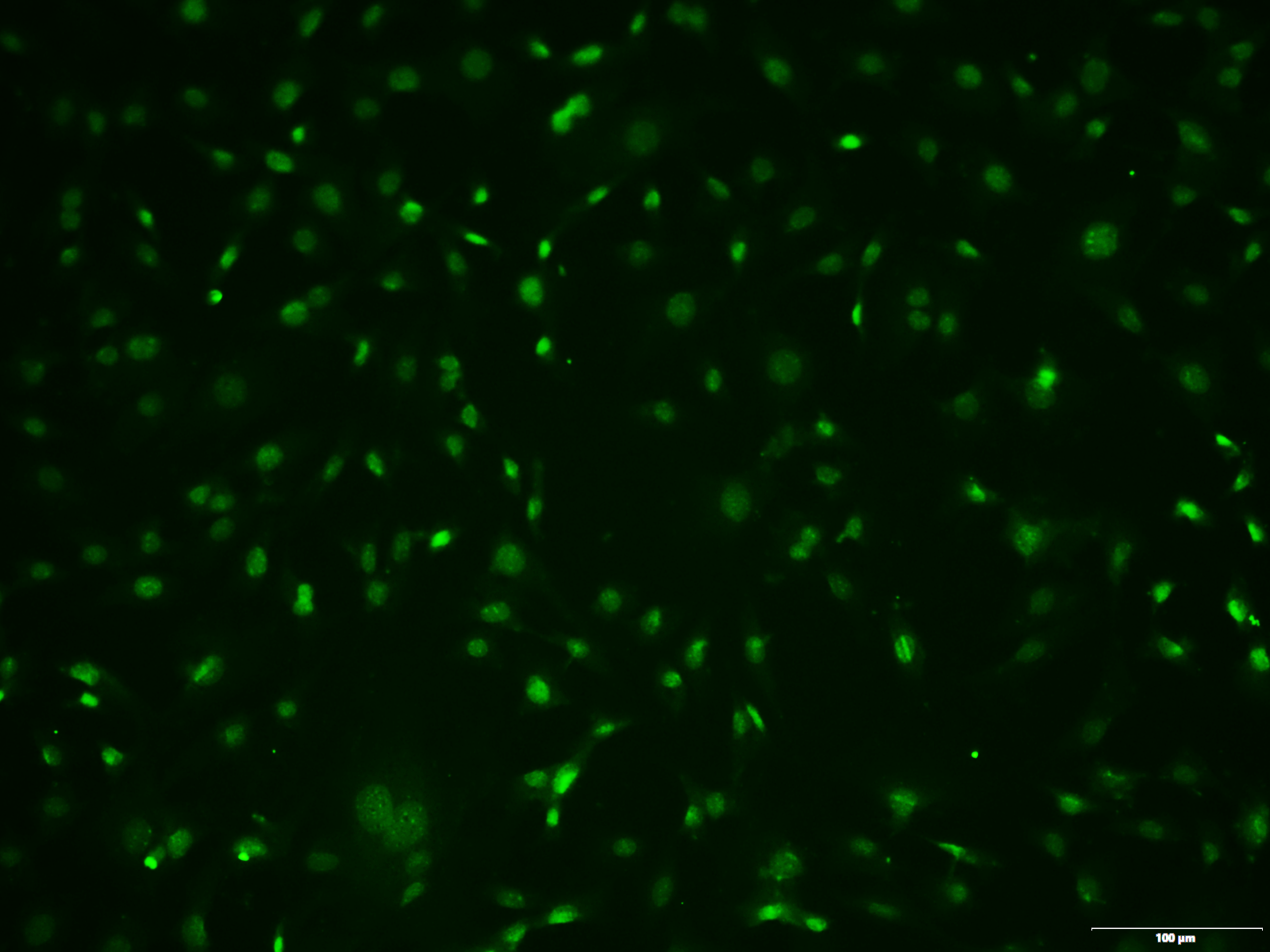

Supplement: Supplementary file 5 — Source data Fig. 4 [file 44319_2024_327_MOESM5_ESM.zip › Figure 4/4F/WT/1 (1).tif]

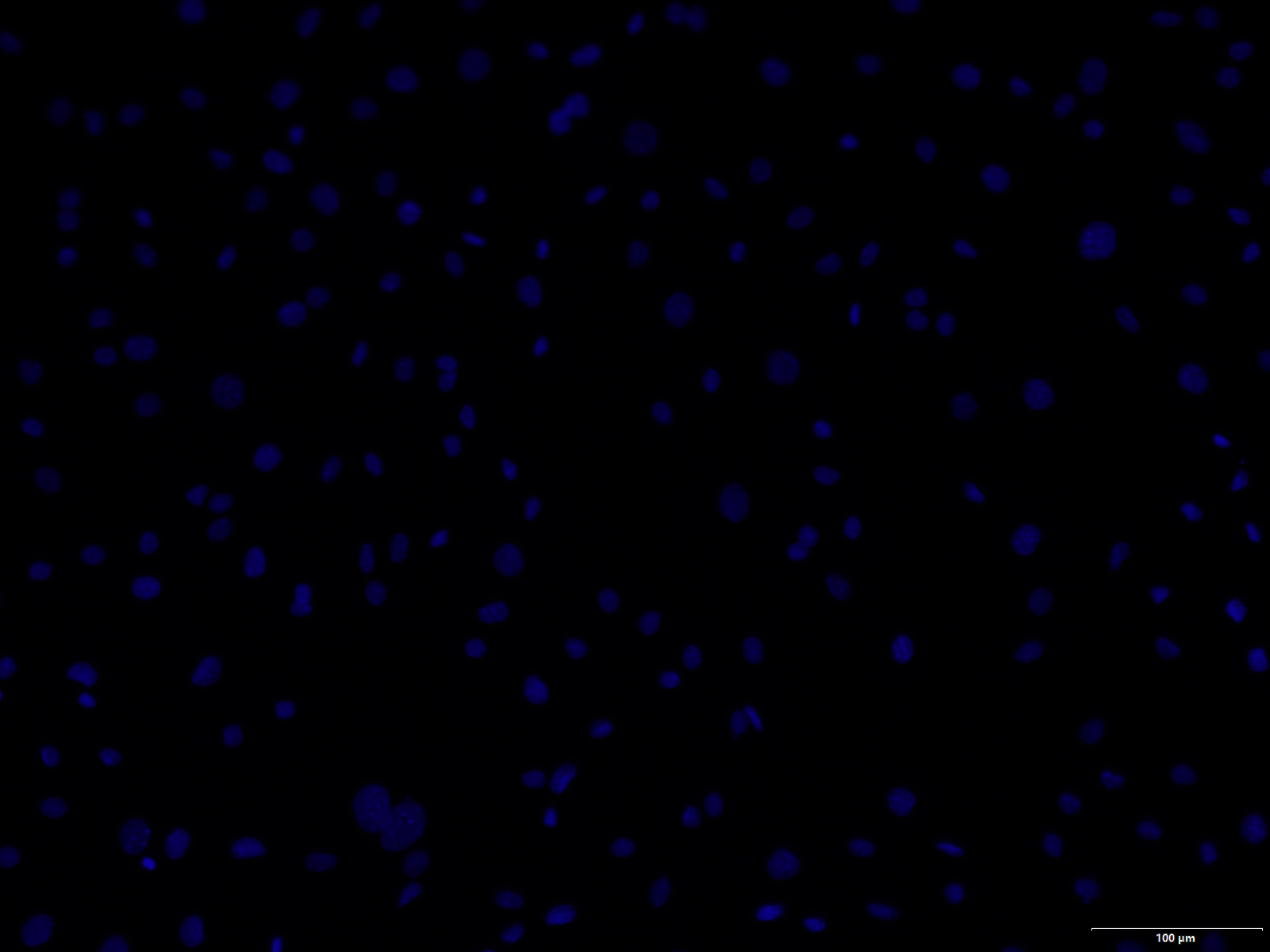

Supplement: Supplementary file 5 — Source data Fig. 4 [file 44319_2024_327_MOESM5_ESM.zip › Figure 4/4F/WT/1 (2).tif]

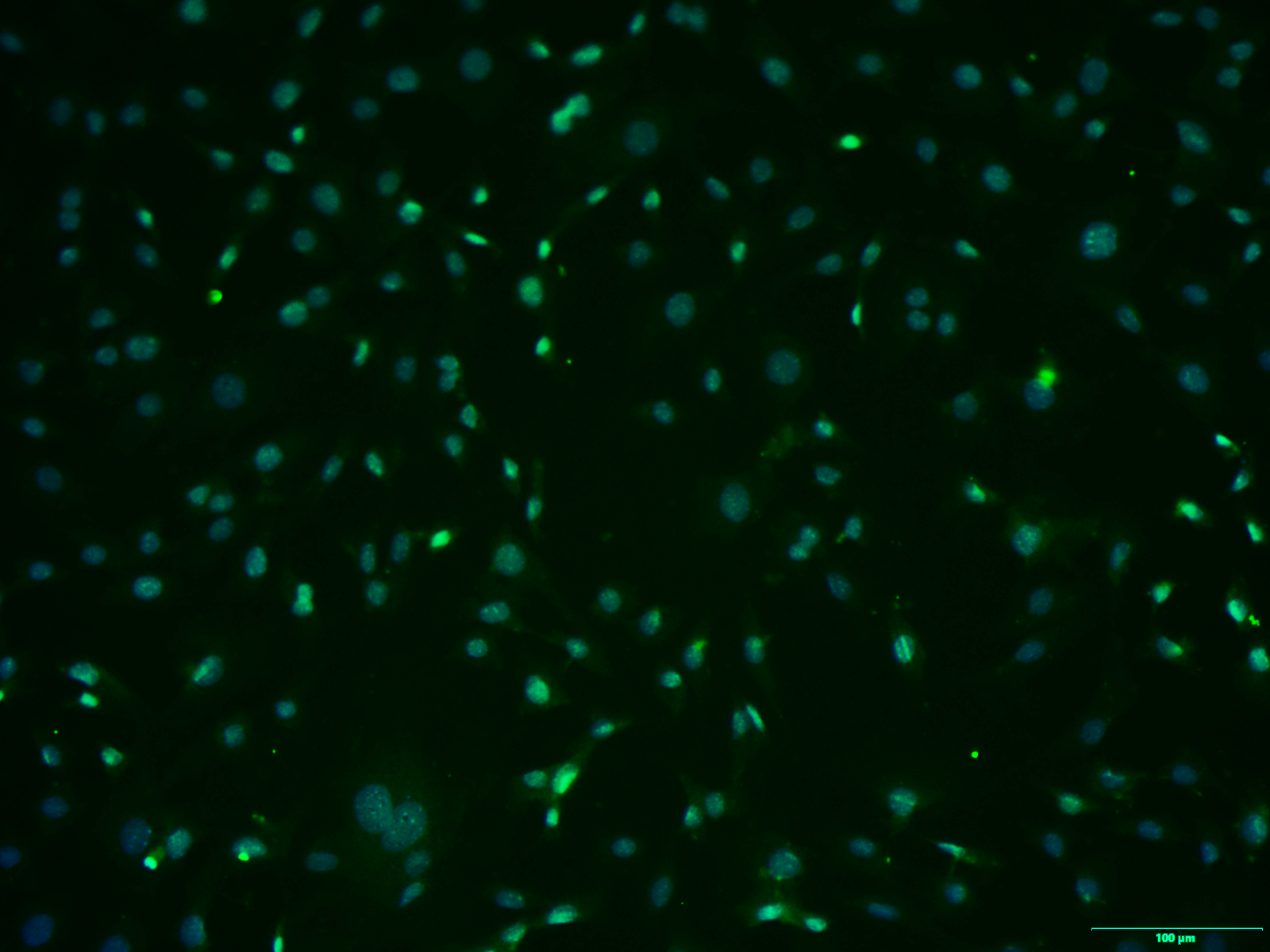

Supplement: Supplementary file 5 — Source data Fig. 4 [file 44319_2024_327_MOESM5_ESM.zip › Figure 4/4F/WT/1 (3).tif]

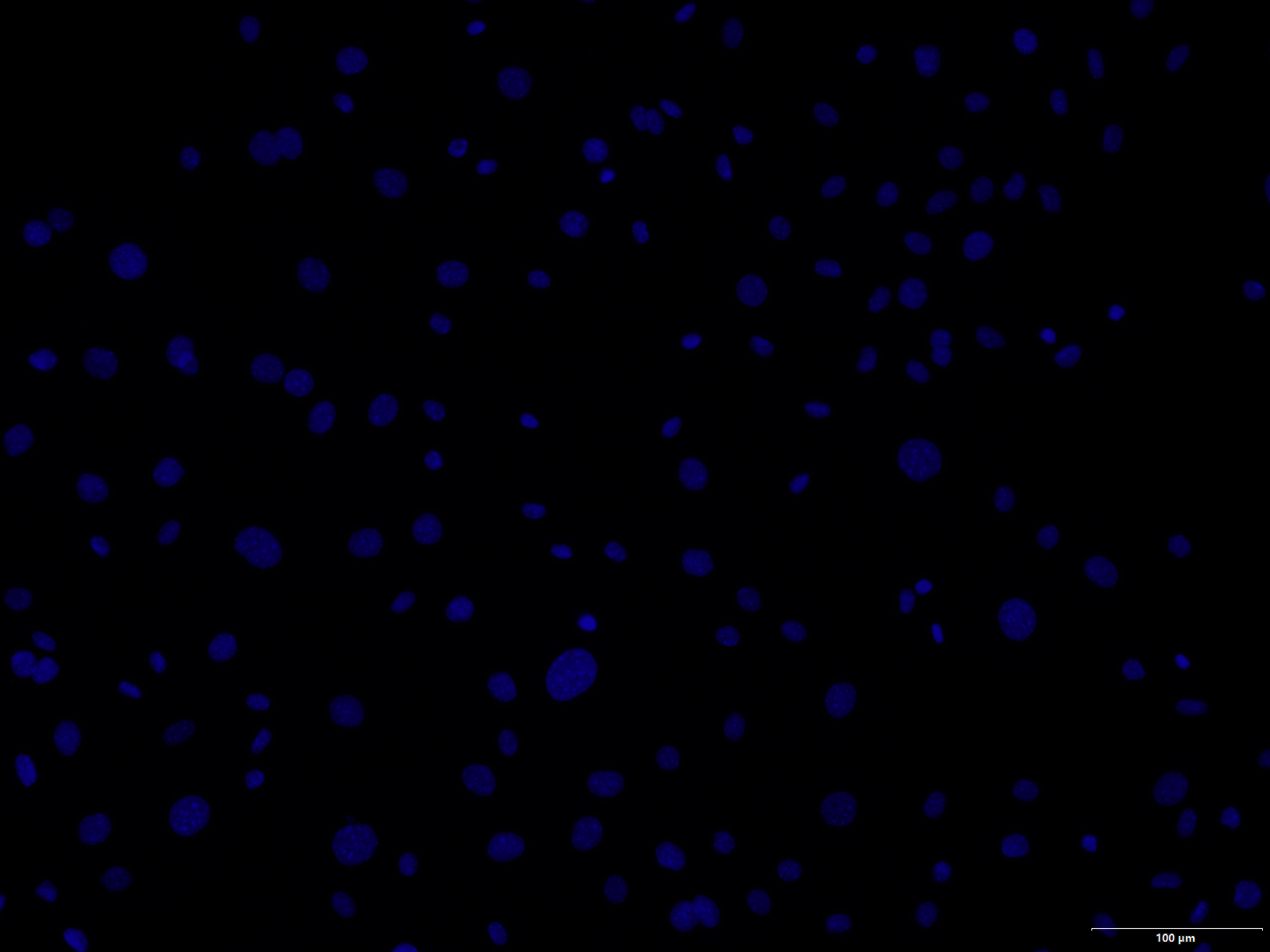

Supplement: Supplementary file 5 — Source data Fig. 4 [file 44319_2024_327_MOESM5_ESM.zip › Figure 4/4F/WT+CsA/1 (1).tif]

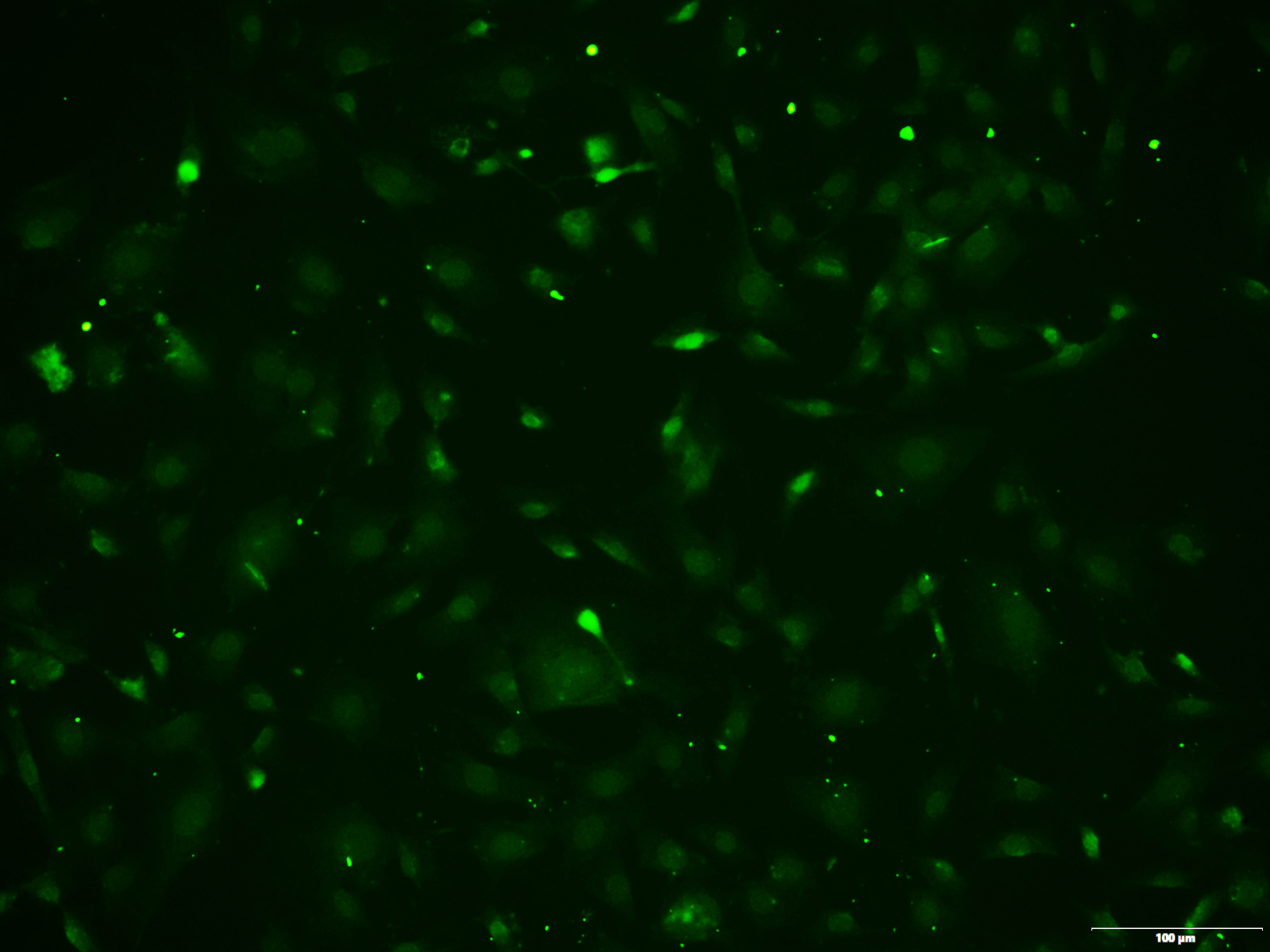

Supplement: Supplementary file 5 — Source data Fig. 4 [file 44319_2024_327_MOESM5_ESM.zip › Figure 4/4F/WT+CsA/1 (2).tif]

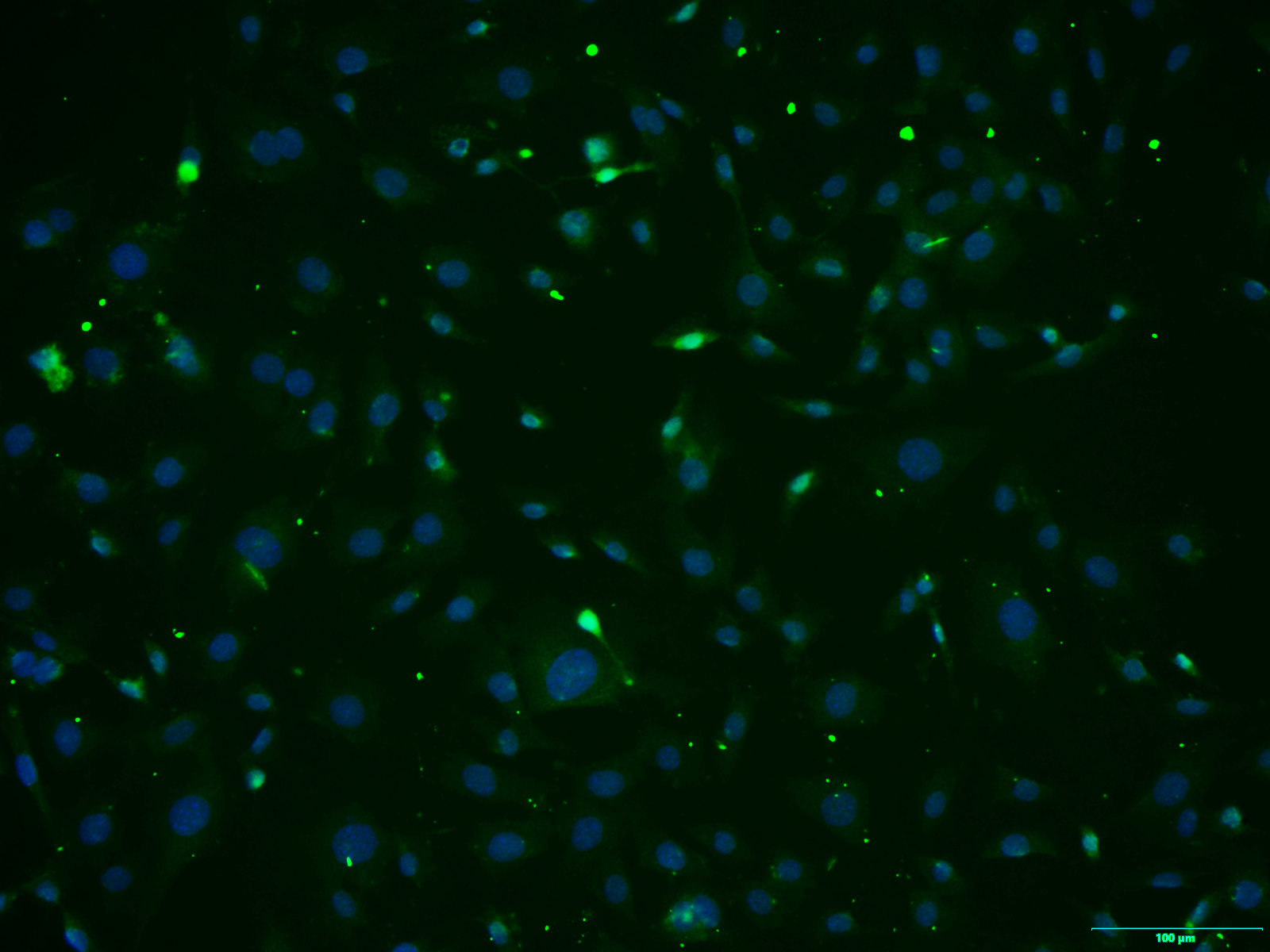

Supplement: Supplementary file 5 — Source data Fig. 4 [file 44319_2024_327_MOESM5_ESM.zip › Figure 4/4F/WT+CsA/1 (3).tif]

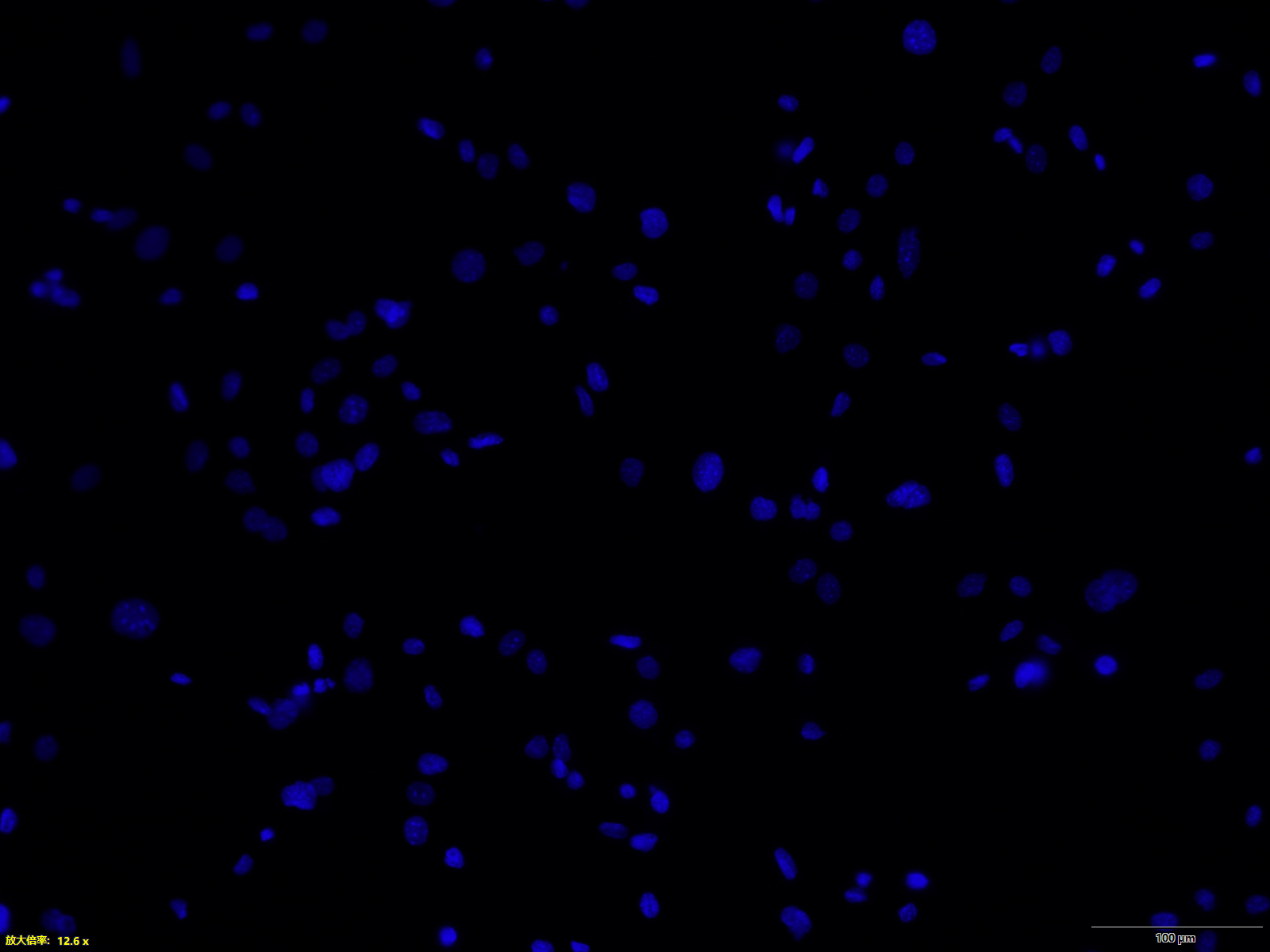

Supplement: Supplementary file 6 — Source data Fig. 5 [file 44319_2024_327_MOESM6_ESM.zip › Figure 5/5C/KO/1 (1).tif]

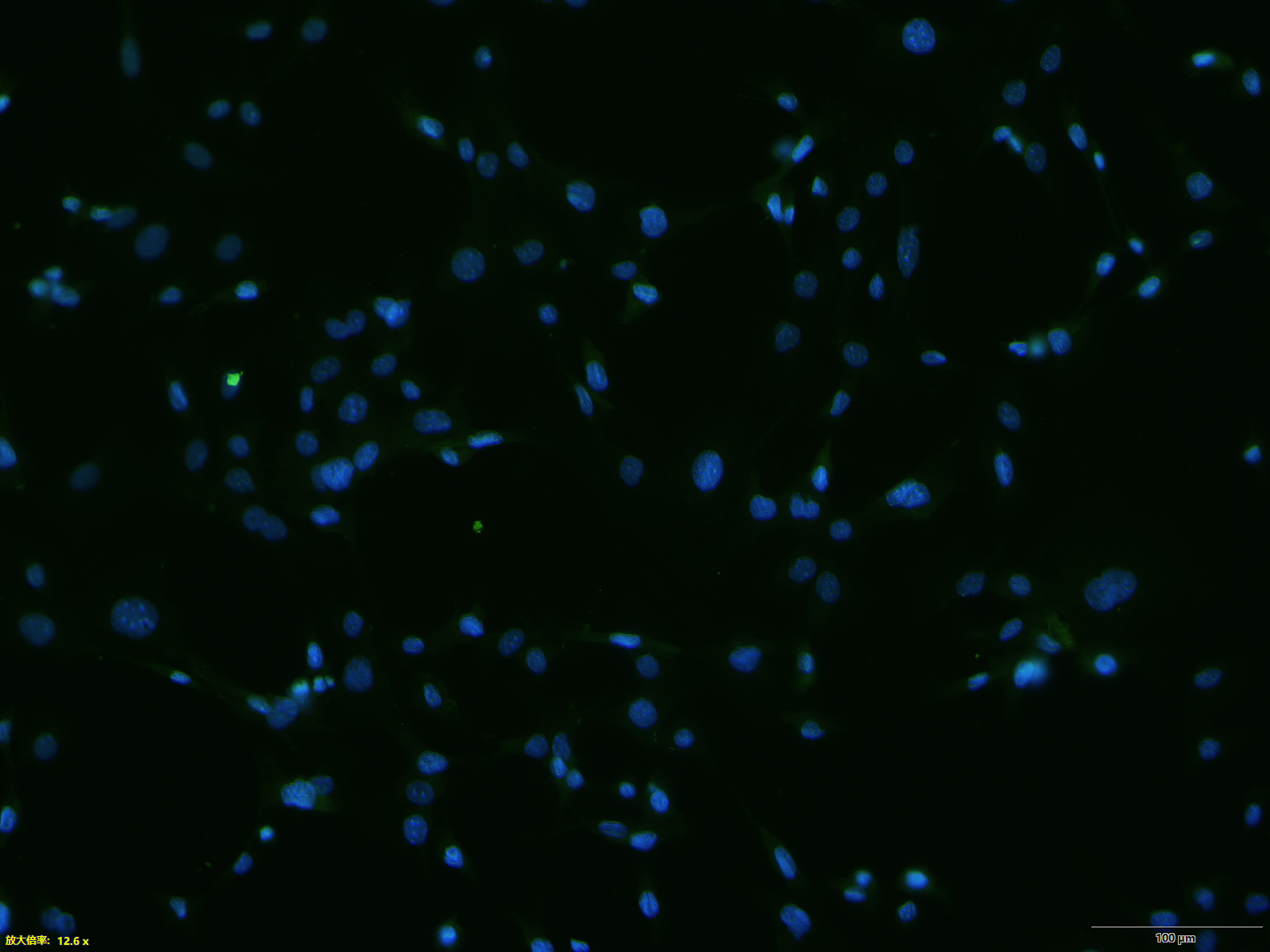

Supplement: Supplementary file 6 — Source data Fig. 5 [file 44319_2024_327_MOESM6_ESM.zip › Figure 5/5C/KO/1 (2).tif]

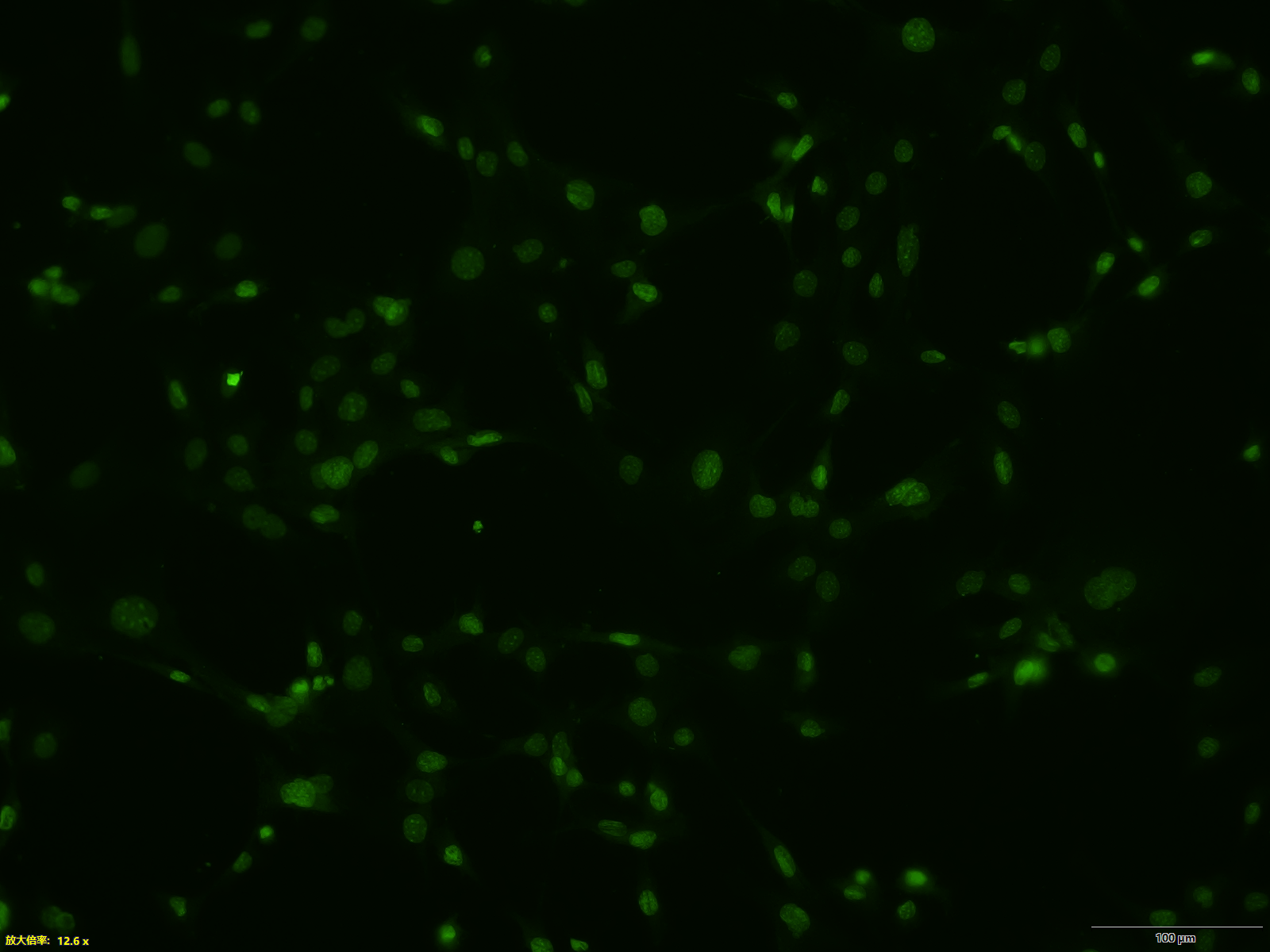

Supplement: Supplementary file 6 — Source data Fig. 5 [file 44319_2024_327_MOESM6_ESM.zip › Figure 5/5C/KO/1 (3).tif]

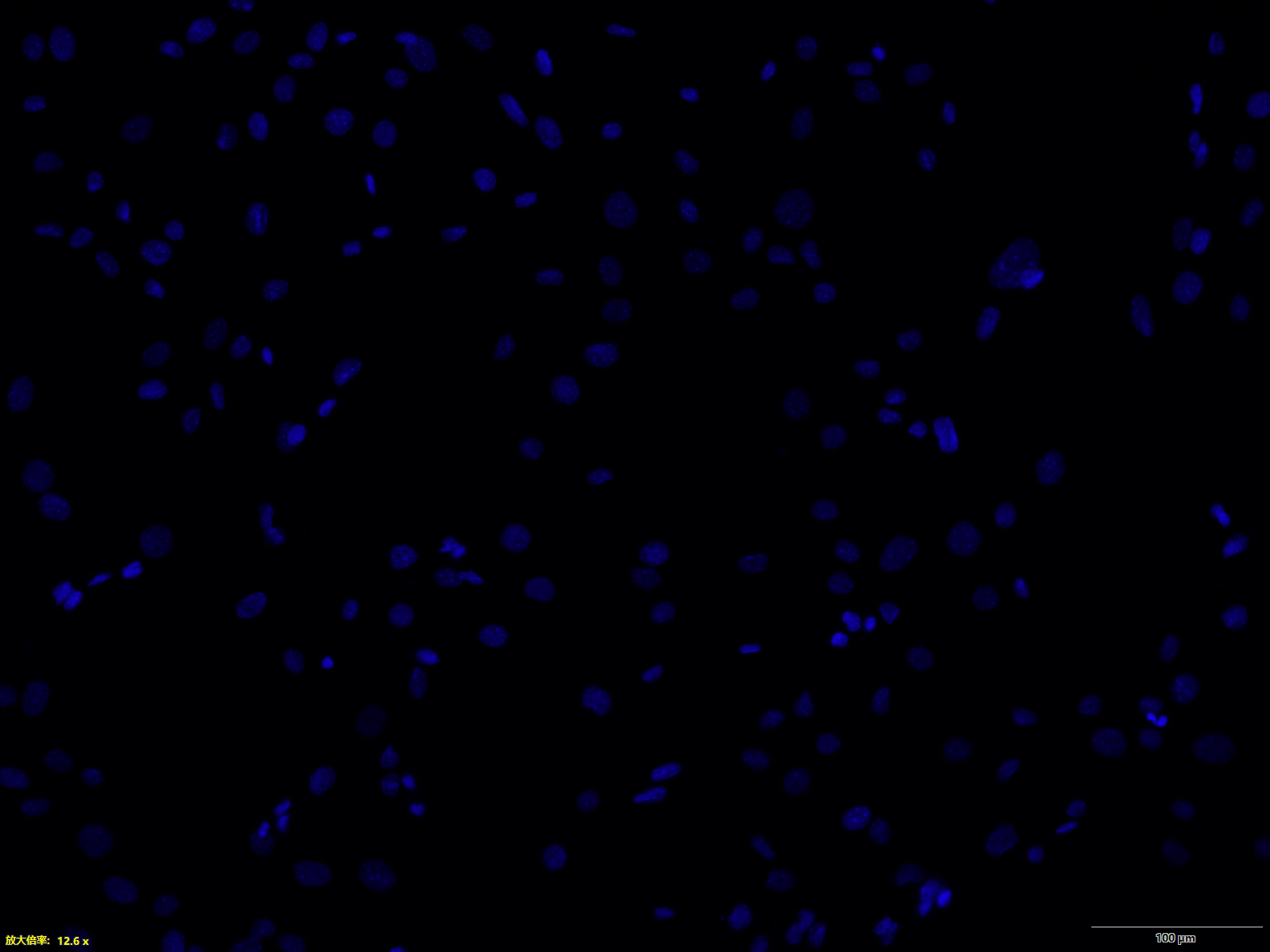

Supplement: Supplementary file 6 — Source data Fig. 5 [file 44319_2024_327_MOESM6_ESM.zip › Figure 5/5C/WT/1 (1).tif]

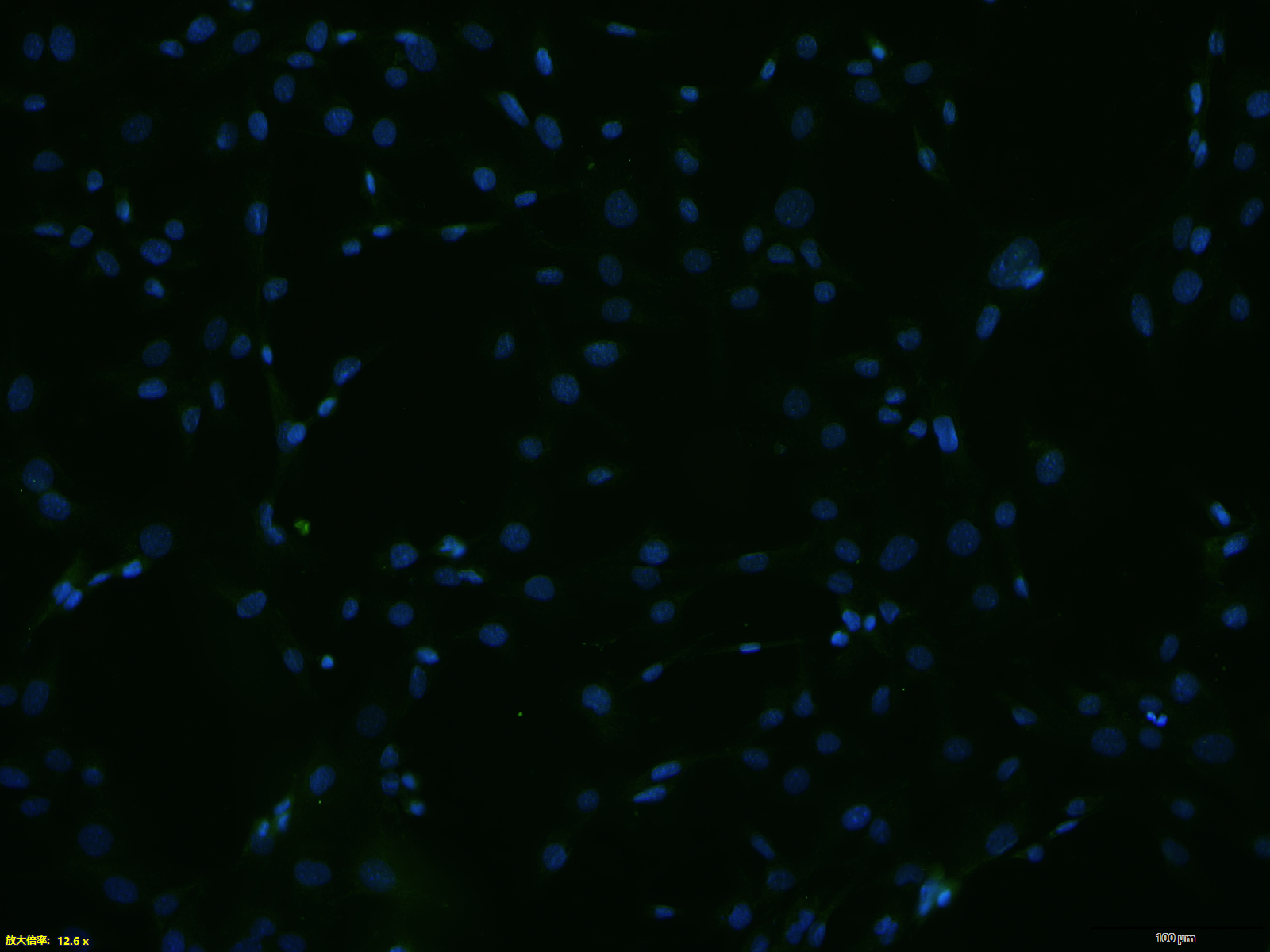

Supplement: Supplementary file 6 — Source data Fig. 5 [file 44319_2024_327_MOESM6_ESM.zip › Figure 5/5C/WT/1 (2).tif]

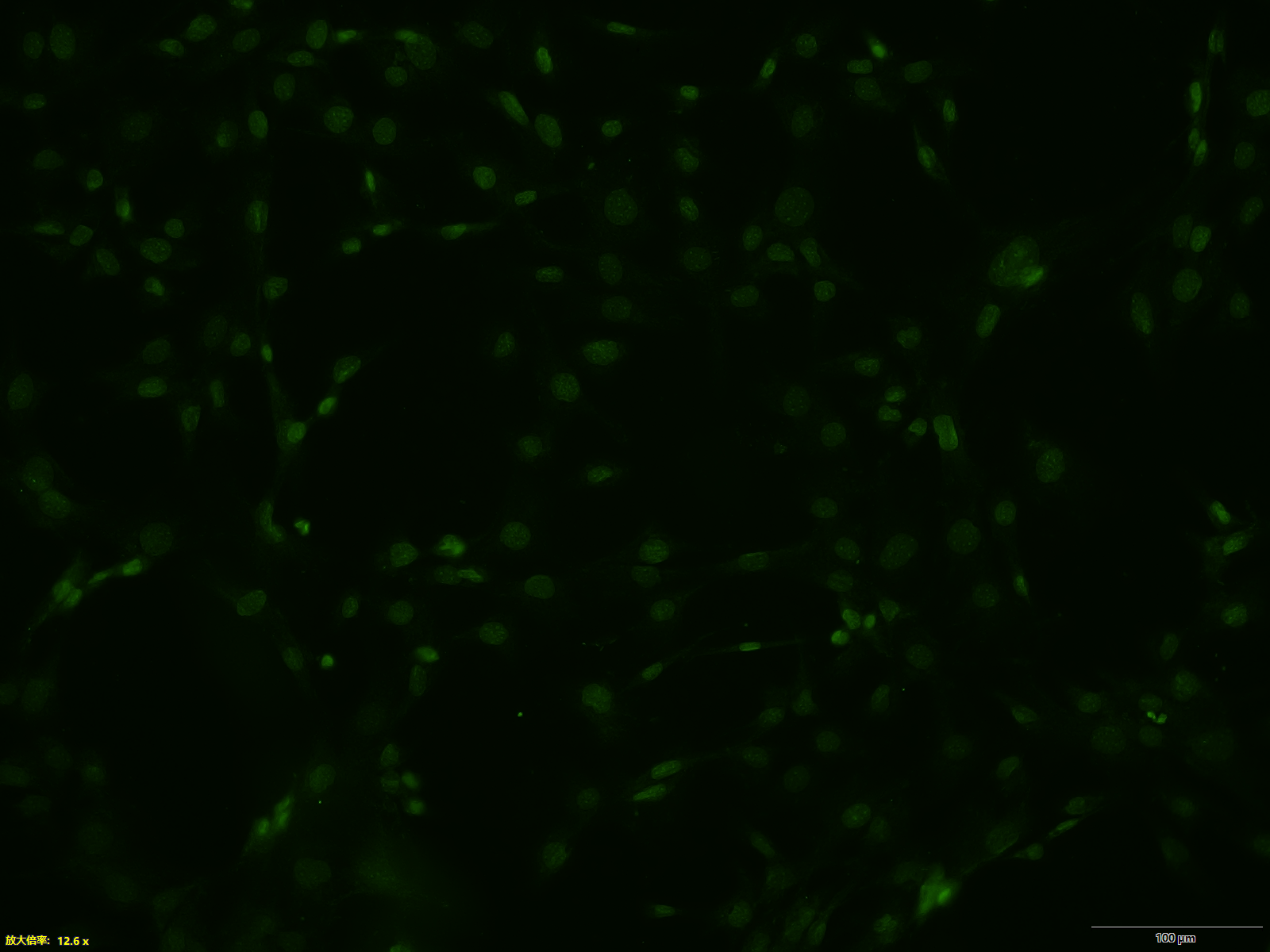

Supplement: Supplementary file 6 — Source data Fig. 5 [file 44319_2024_327_MOESM6_ESM.zip › Figure 5/5C/WT/1 (3).tif]

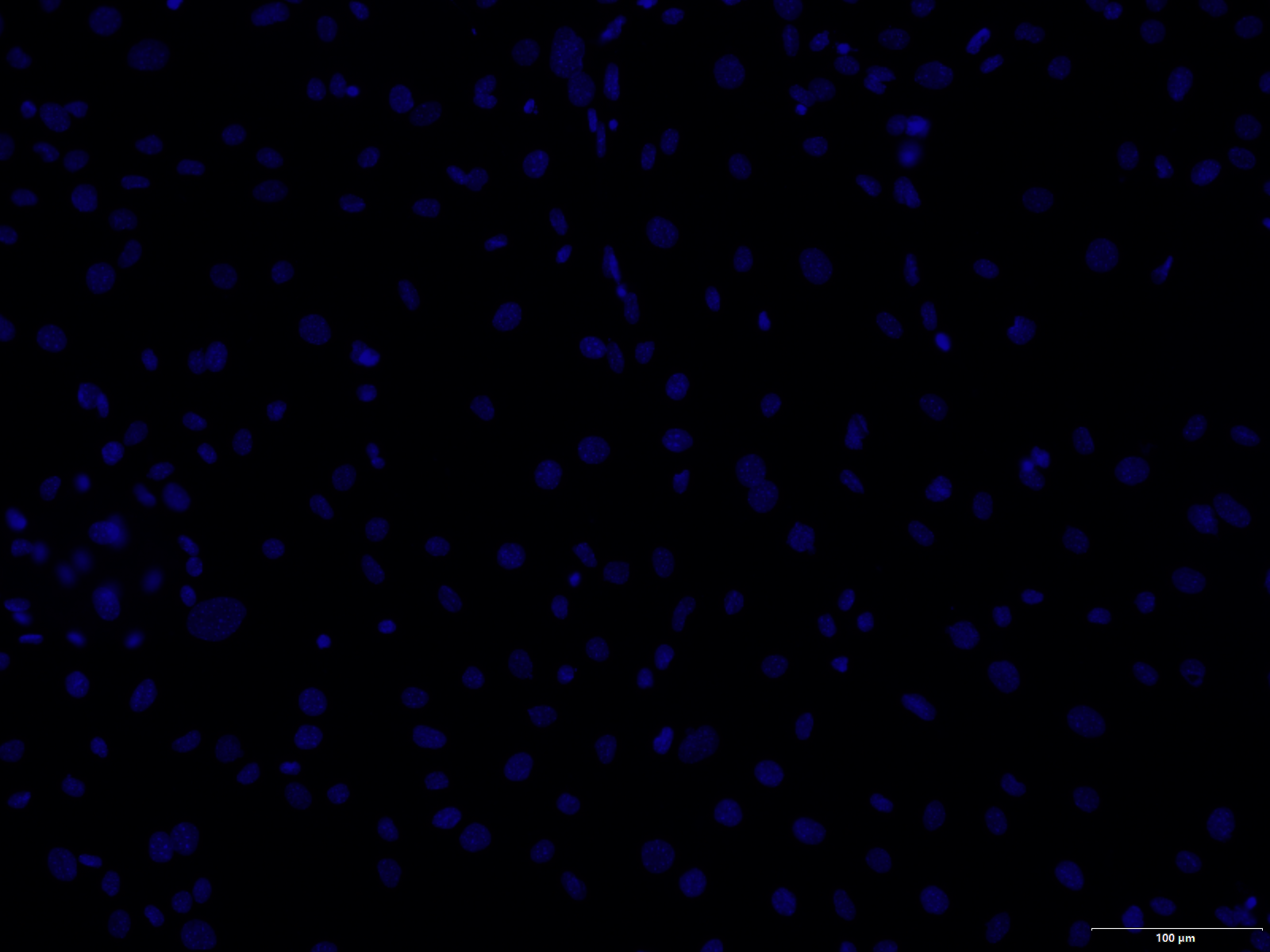

Supplement: Supplementary file 6 — Source data Fig. 5 [file 44319_2024_327_MOESM6_ESM.zip › Figure 5/5E/4F/KO/1 (1).tif]

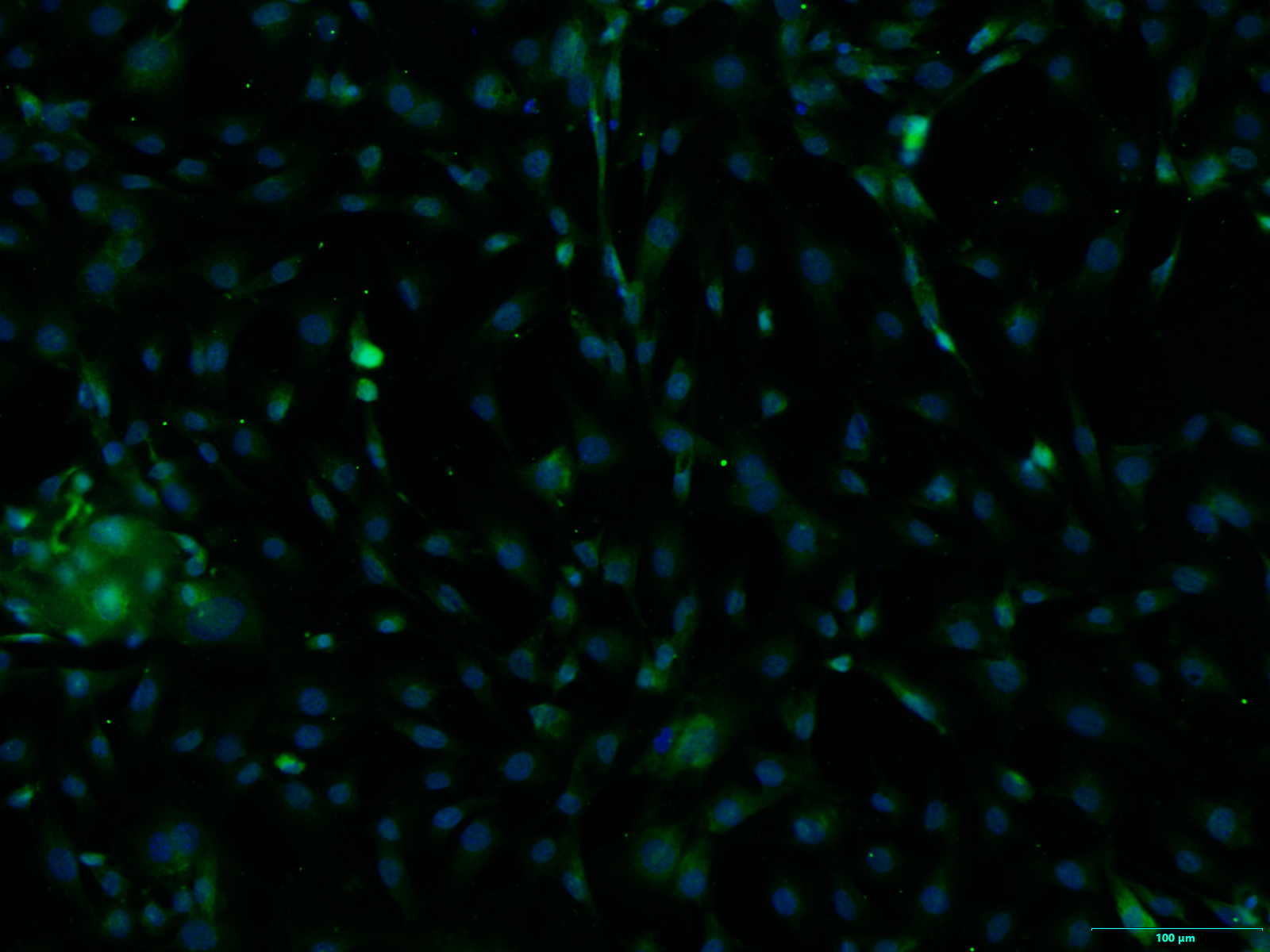

Supplement: Supplementary file 6 — Source data Fig. 5 [file 44319_2024_327_MOESM6_ESM.zip › Figure 5/5E/4F/KO/1 (2).tif]

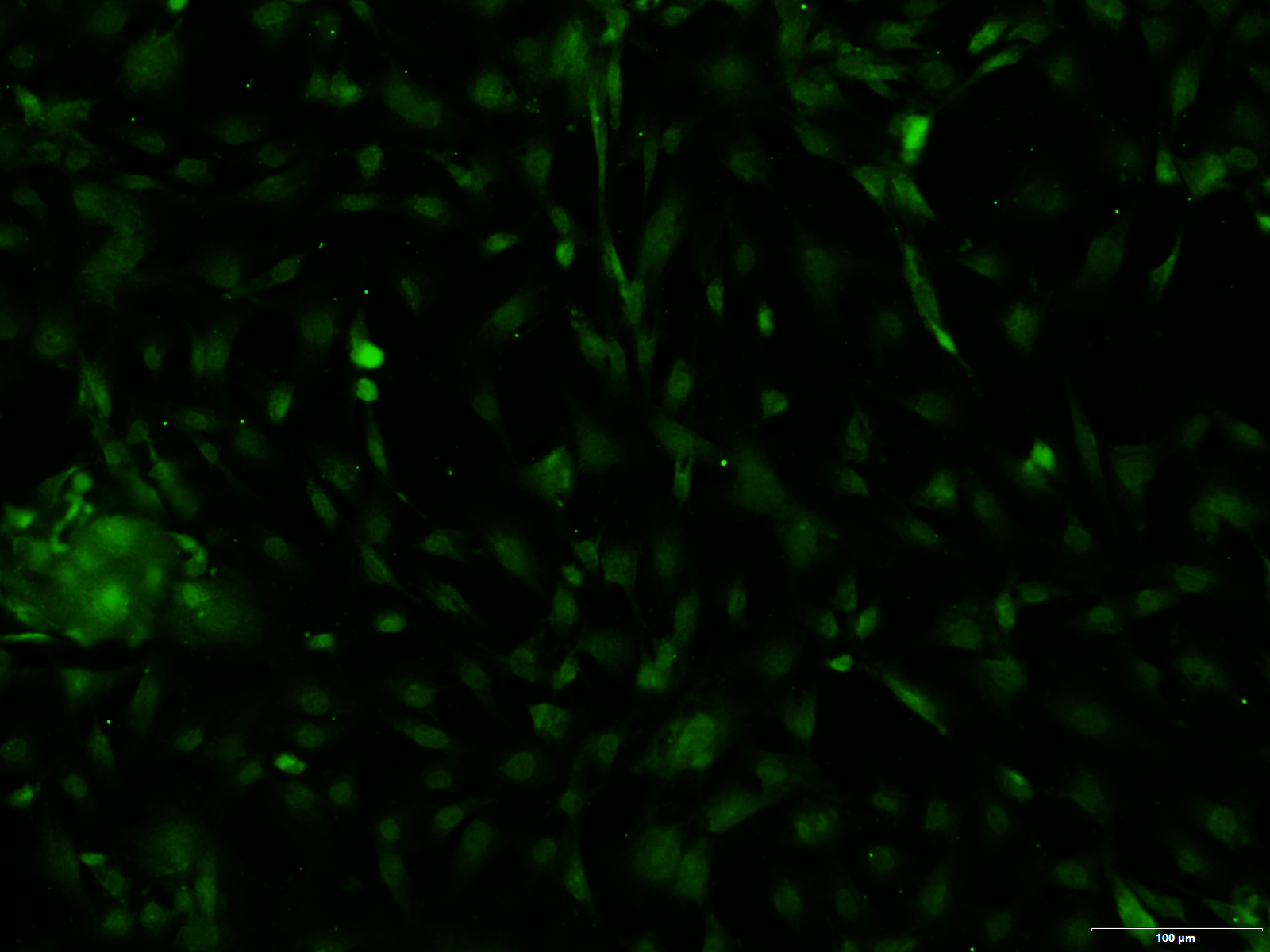

Supplement: Supplementary file 6 — Source data Fig. 5 [file 44319_2024_327_MOESM6_ESM.zip › Figure 5/5E/4F/KO/1 (3).tif]

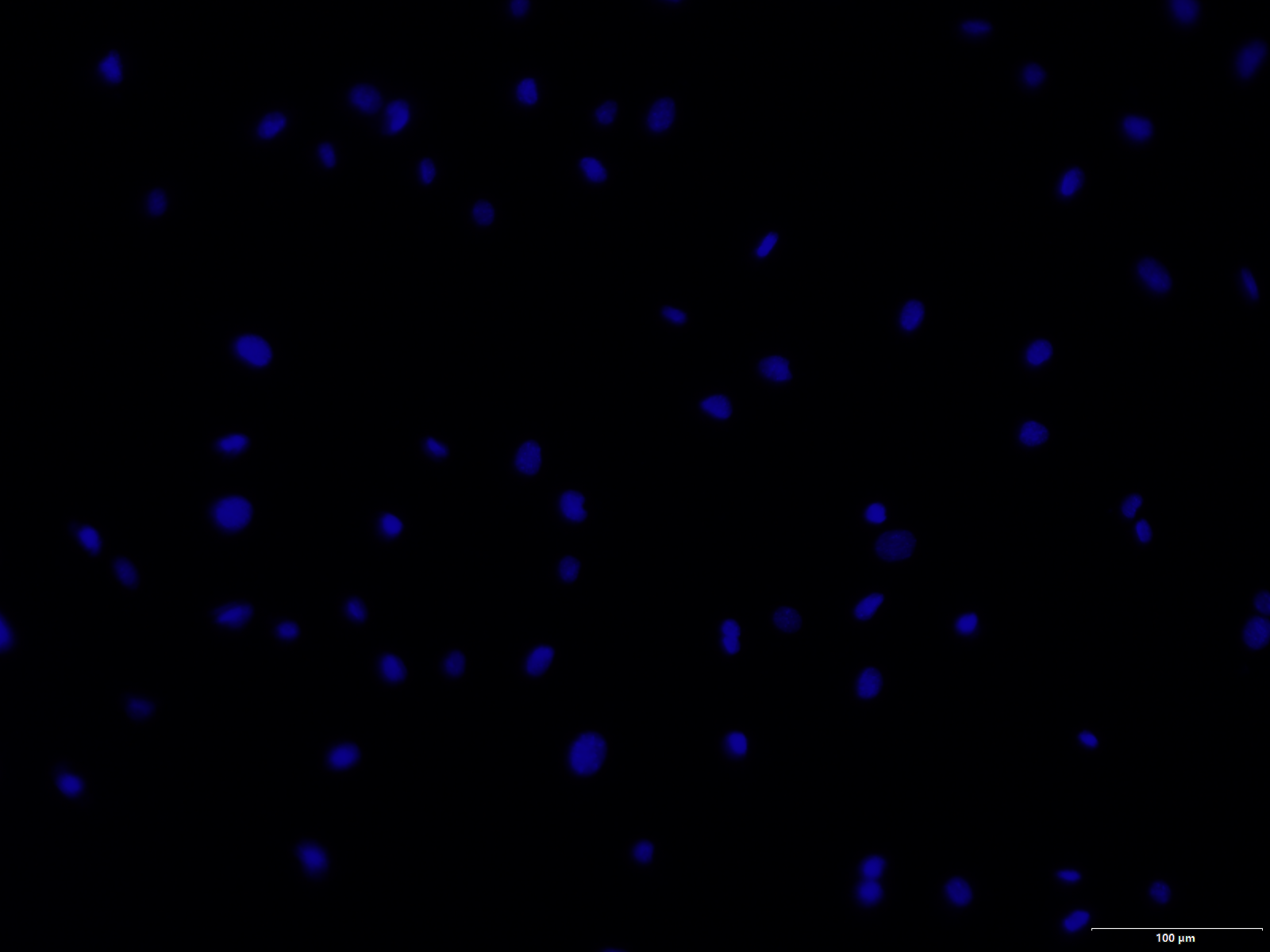

Supplement: Supplementary file 6 — Source data Fig. 5 [file 44319_2024_327_MOESM6_ESM.zip › Figure 5/5E/4F/WT/1 (1).tif]

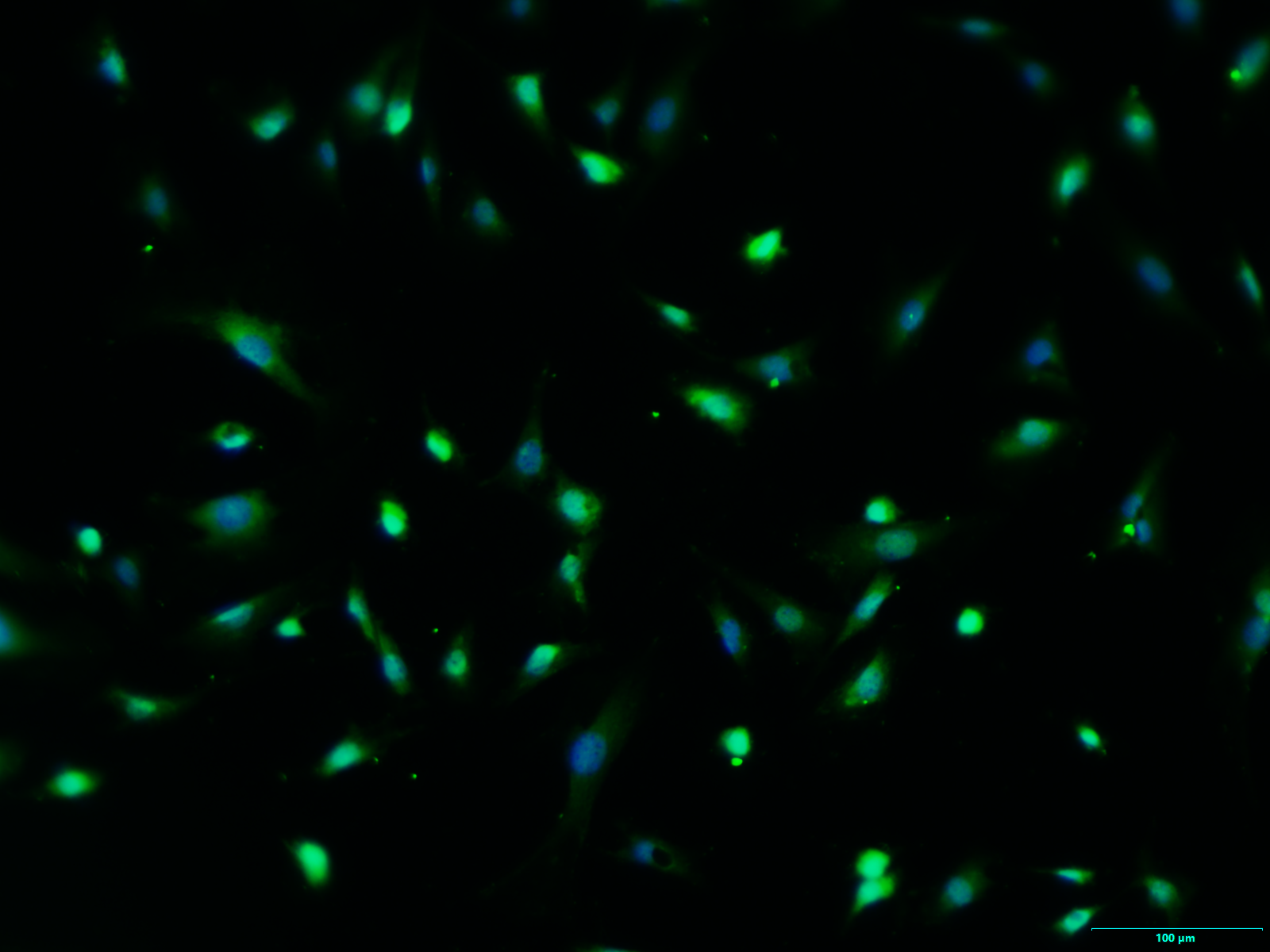

Supplement: Supplementary file 6 — Source data Fig. 5 [file 44319_2024_327_MOESM6_ESM.zip › Figure 5/5E/4F/WT/1 (2).tif]

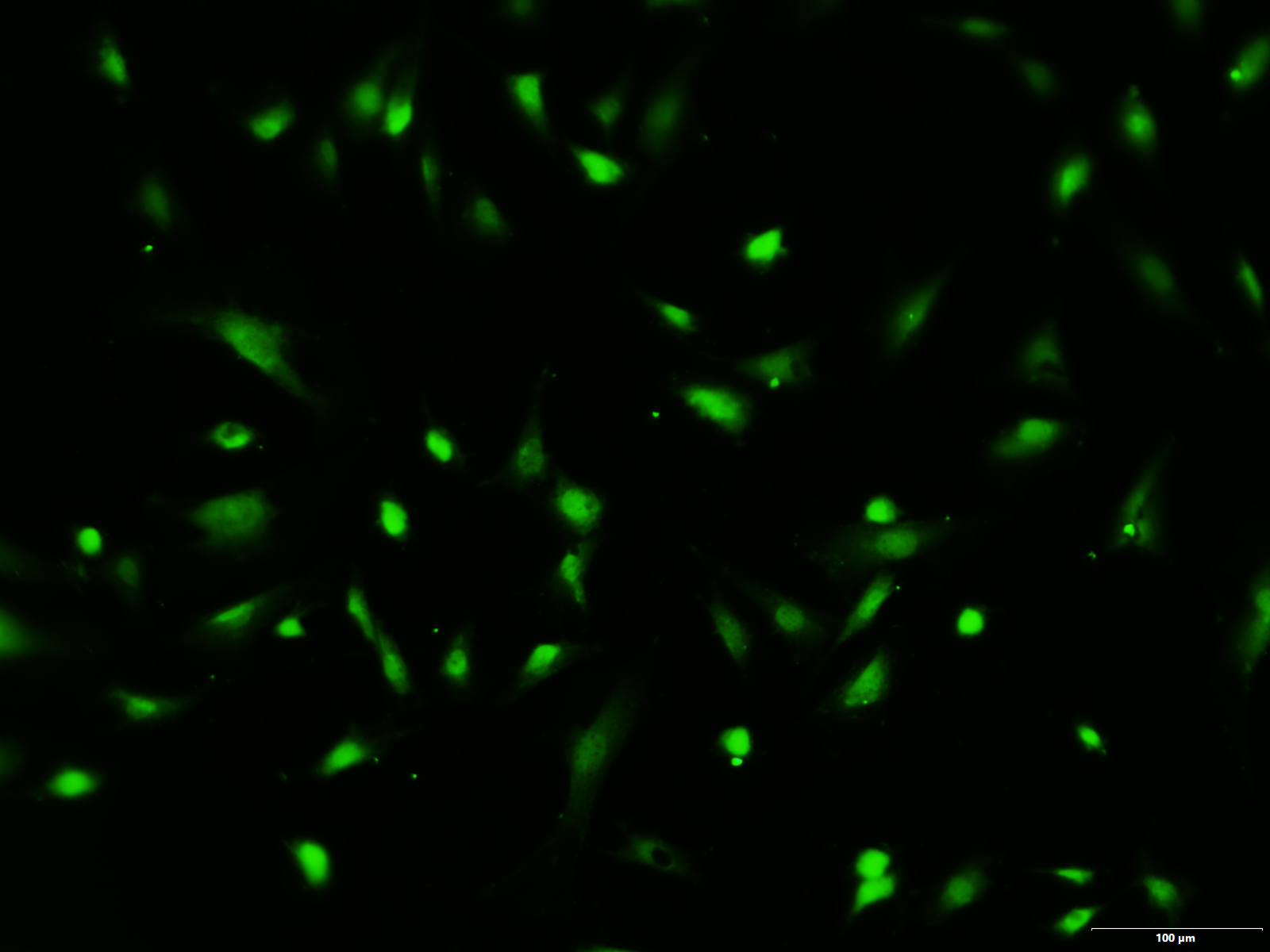

Supplement: Supplementary file 6 — Source data Fig. 5 [file 44319_2024_327_MOESM6_ESM.zip › Figure 5/5E/4F/WT/1 (3).tif]

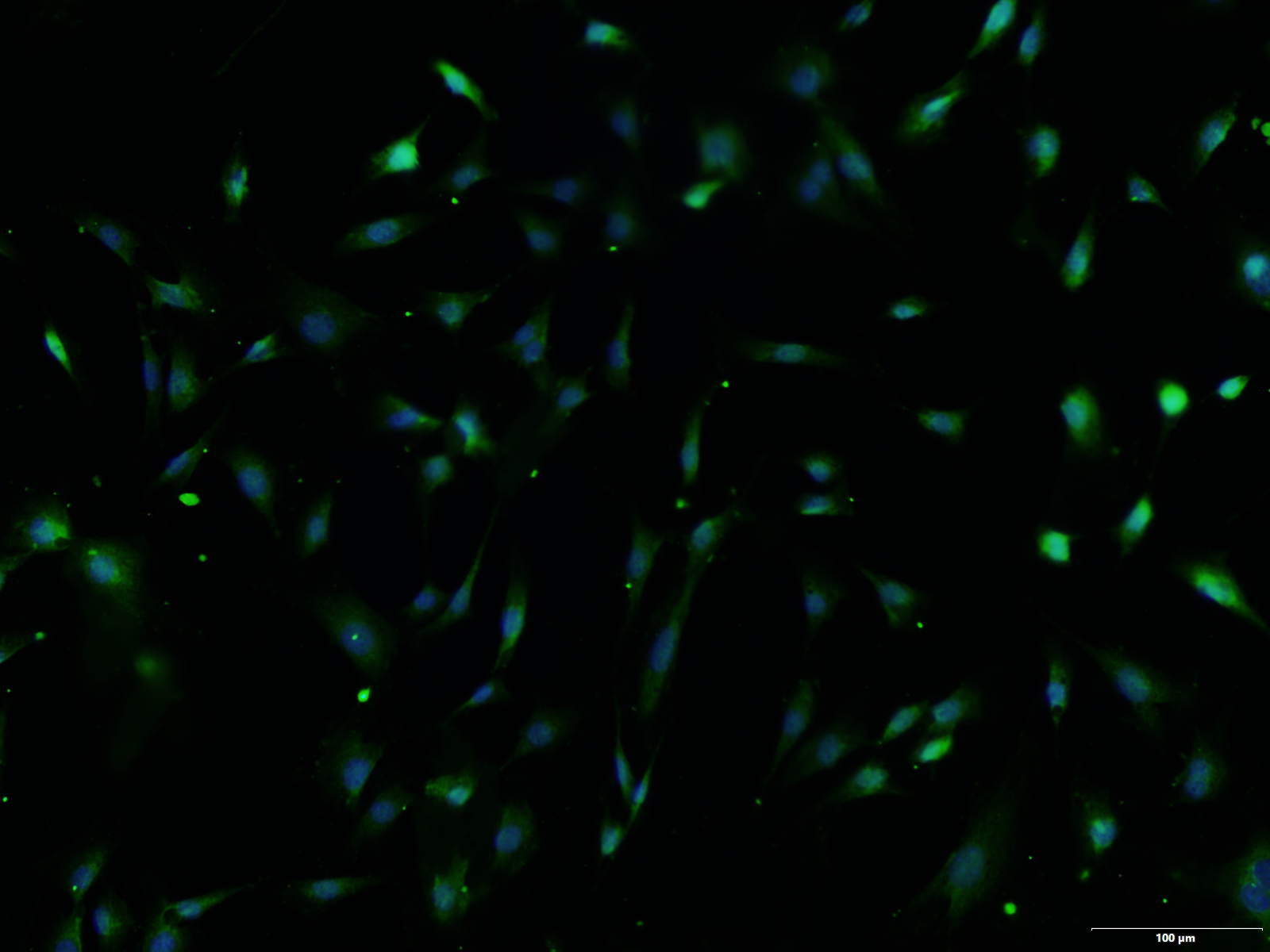

Supplement: Supplementary file 6 — Source data Fig. 5 [file 44319_2024_327_MOESM6_ESM.zip › Figure 5/5E/4F/WT+CsA/1 (1).tif]

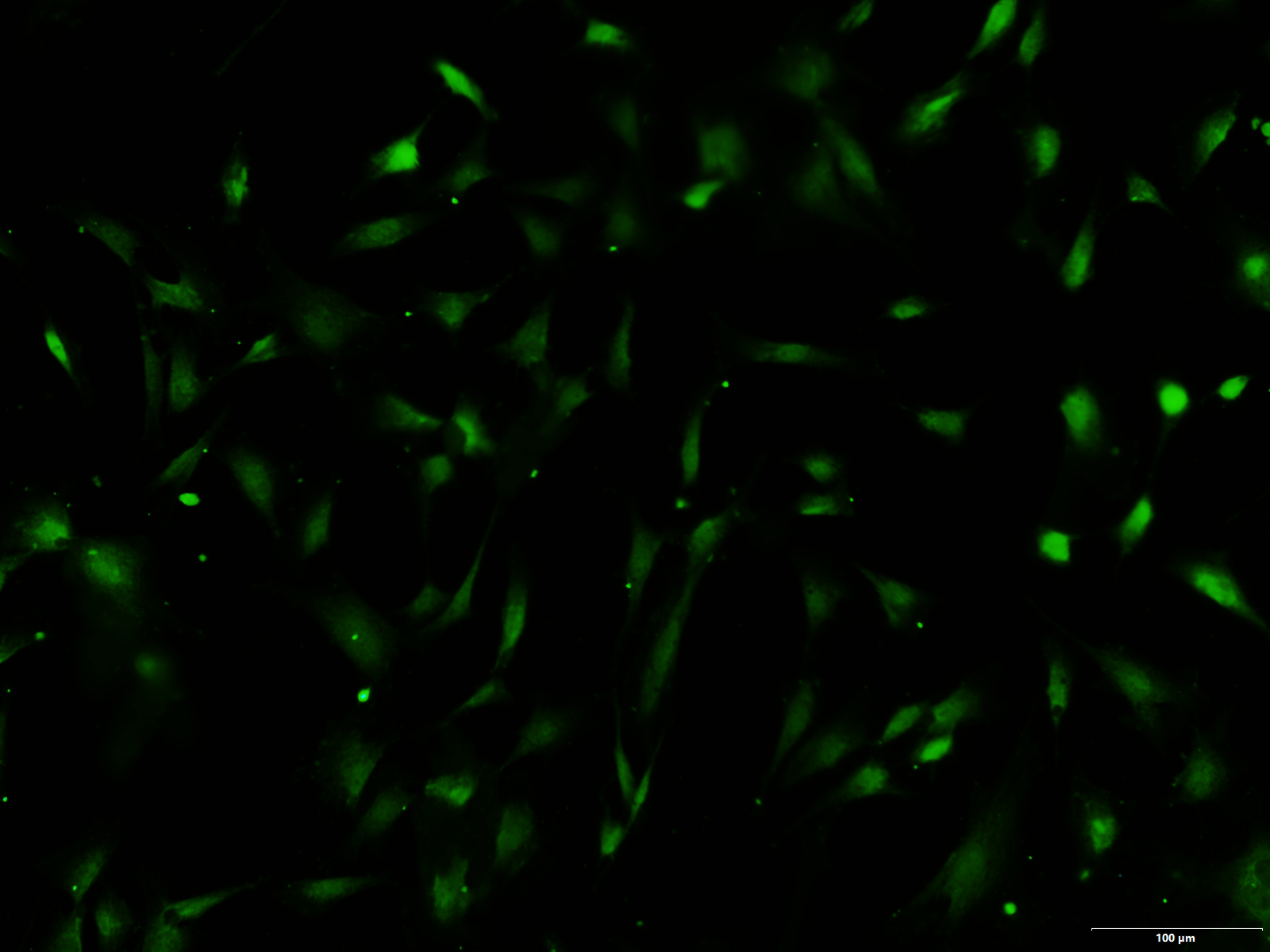

Supplement: Supplementary file 6 — Source data Fig. 5 [file 44319_2024_327_MOESM6_ESM.zip › Figure 5/5E/4F/WT+CsA/1 (2).tif]

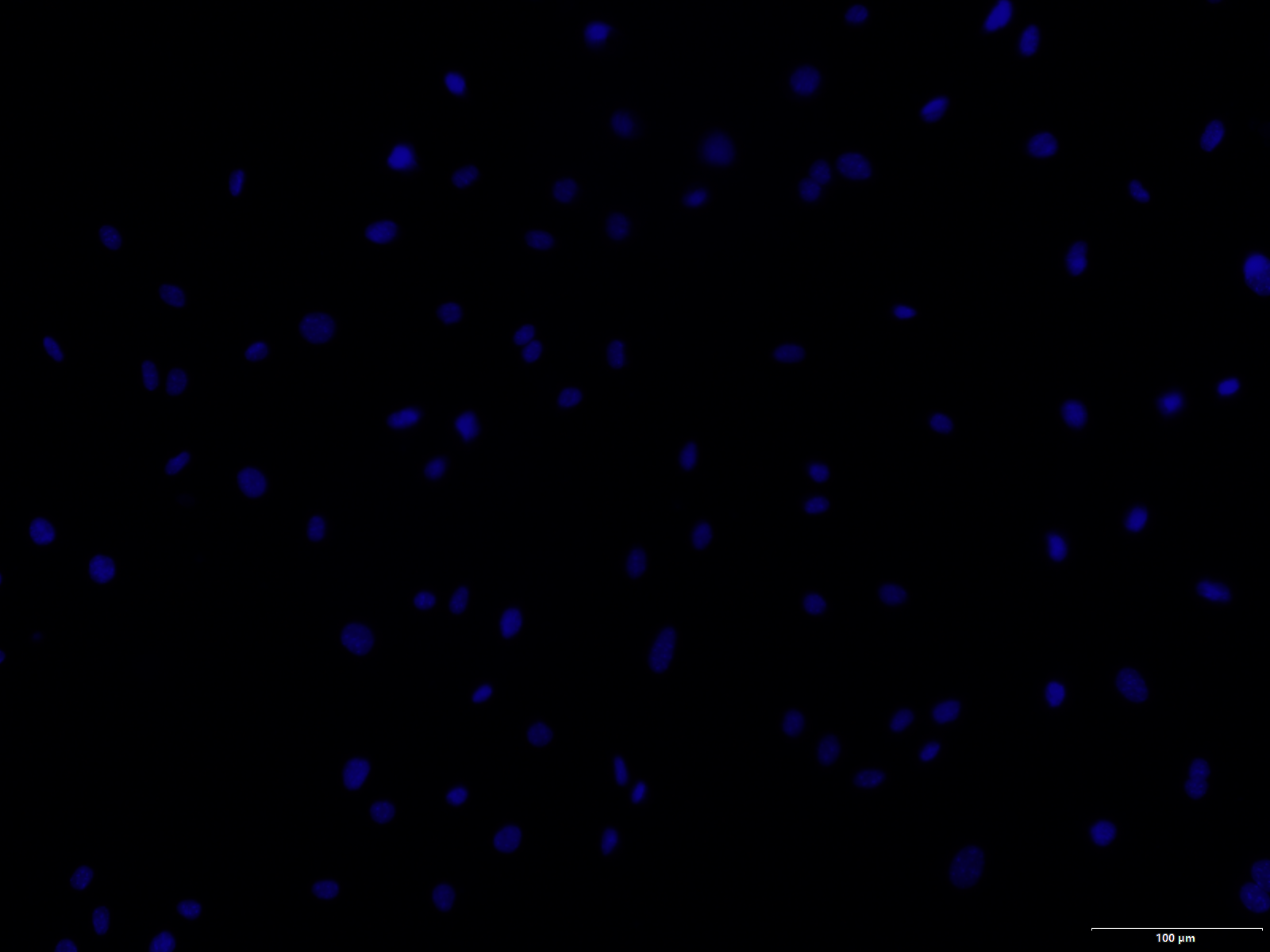

Supplement: Supplementary file 6 — Source data Fig. 5 [file 44319_2024_327_MOESM6_ESM.zip › Figure 5/5E/4F/WT+CsA/1 (3).tif]

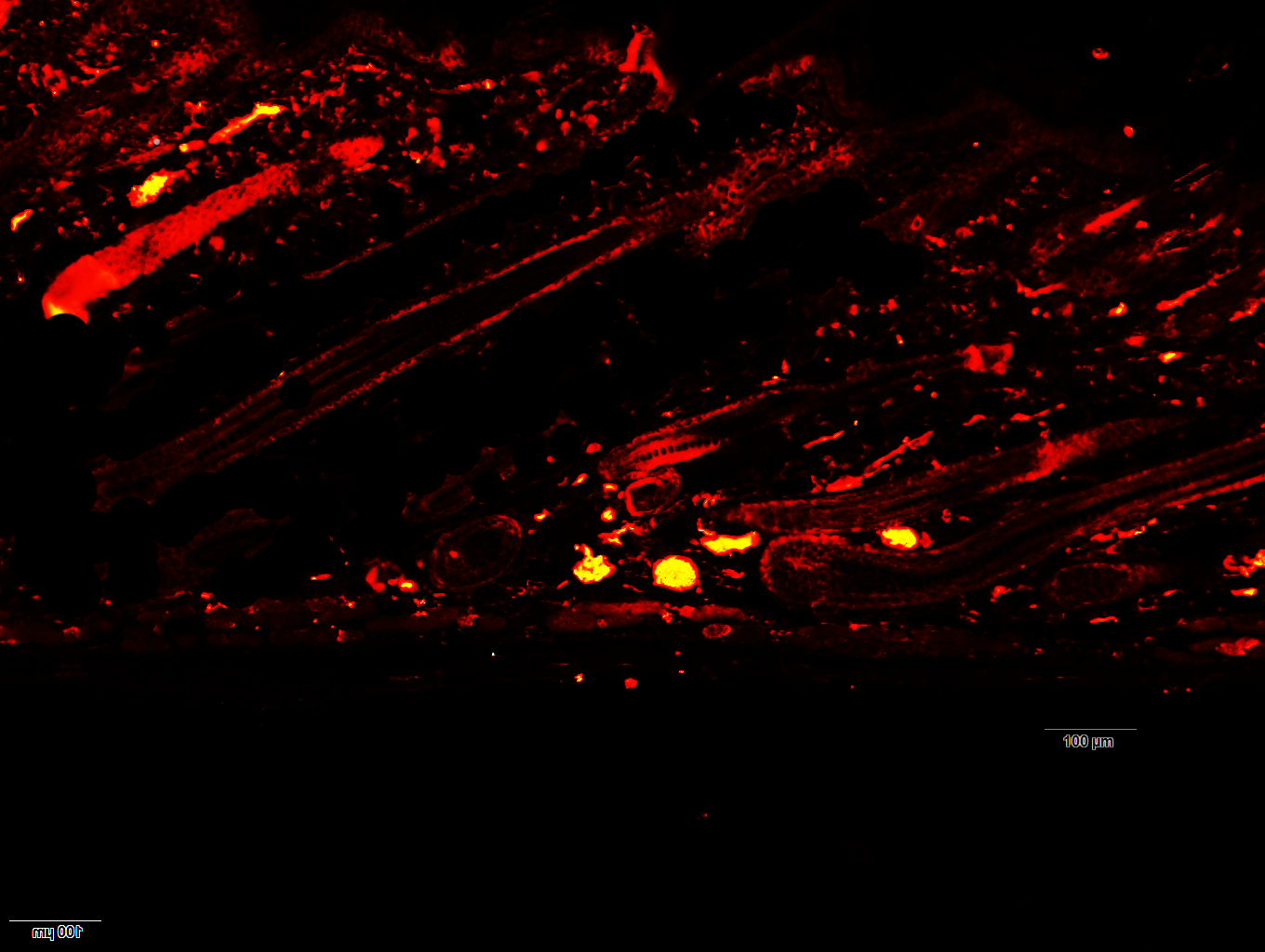

Supplement: Supplementary file 7 — Source data Fig. 6 [file 44319_2024_327_MOESM7_ESM.zip › Figure 6/6A/1 (1).tif]

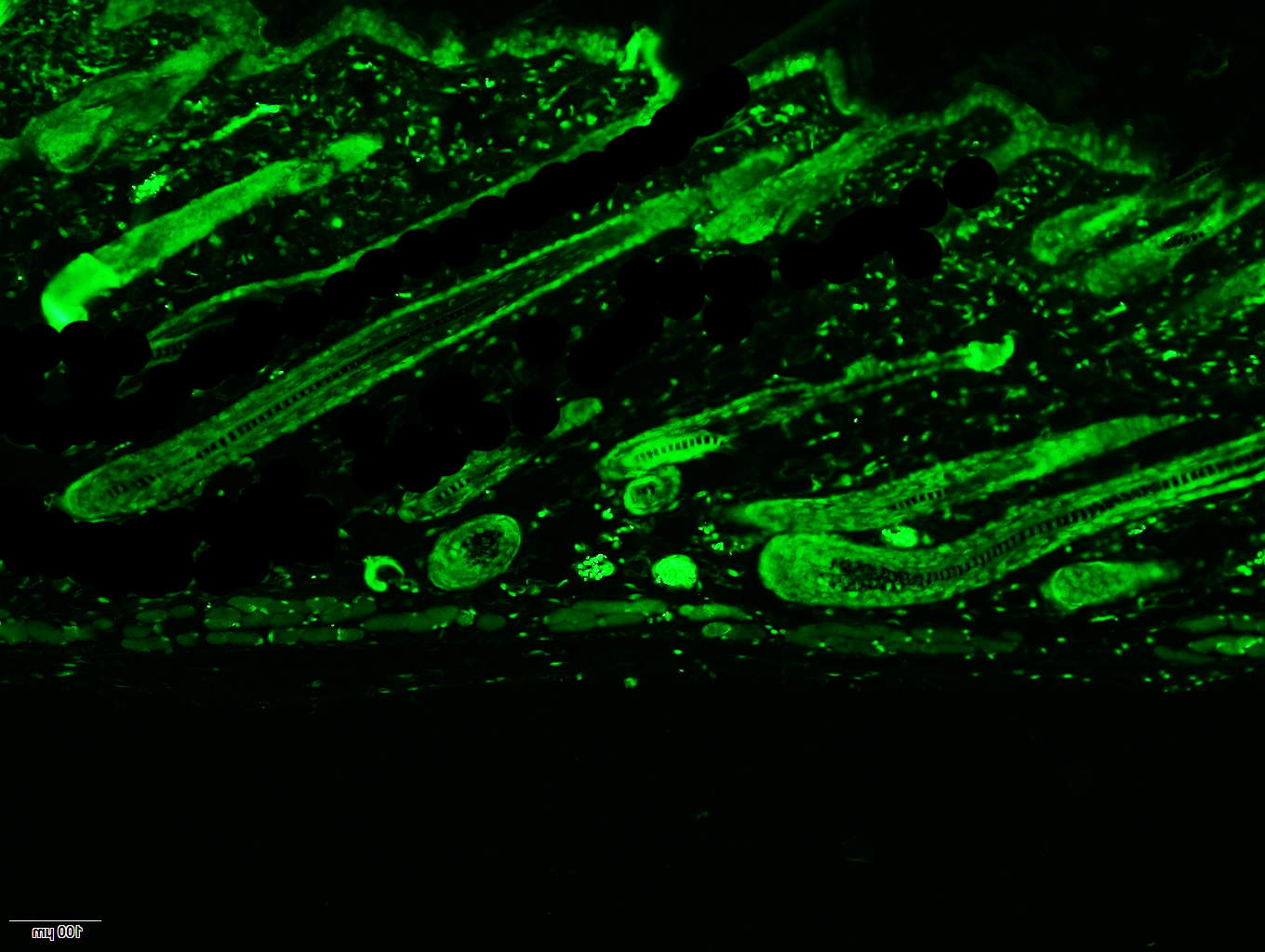

Supplement: Supplementary file 7 — Source data Fig. 6 [file 44319_2024_327_MOESM7_ESM.zip › Figure 6/6A/1 (2).tif]

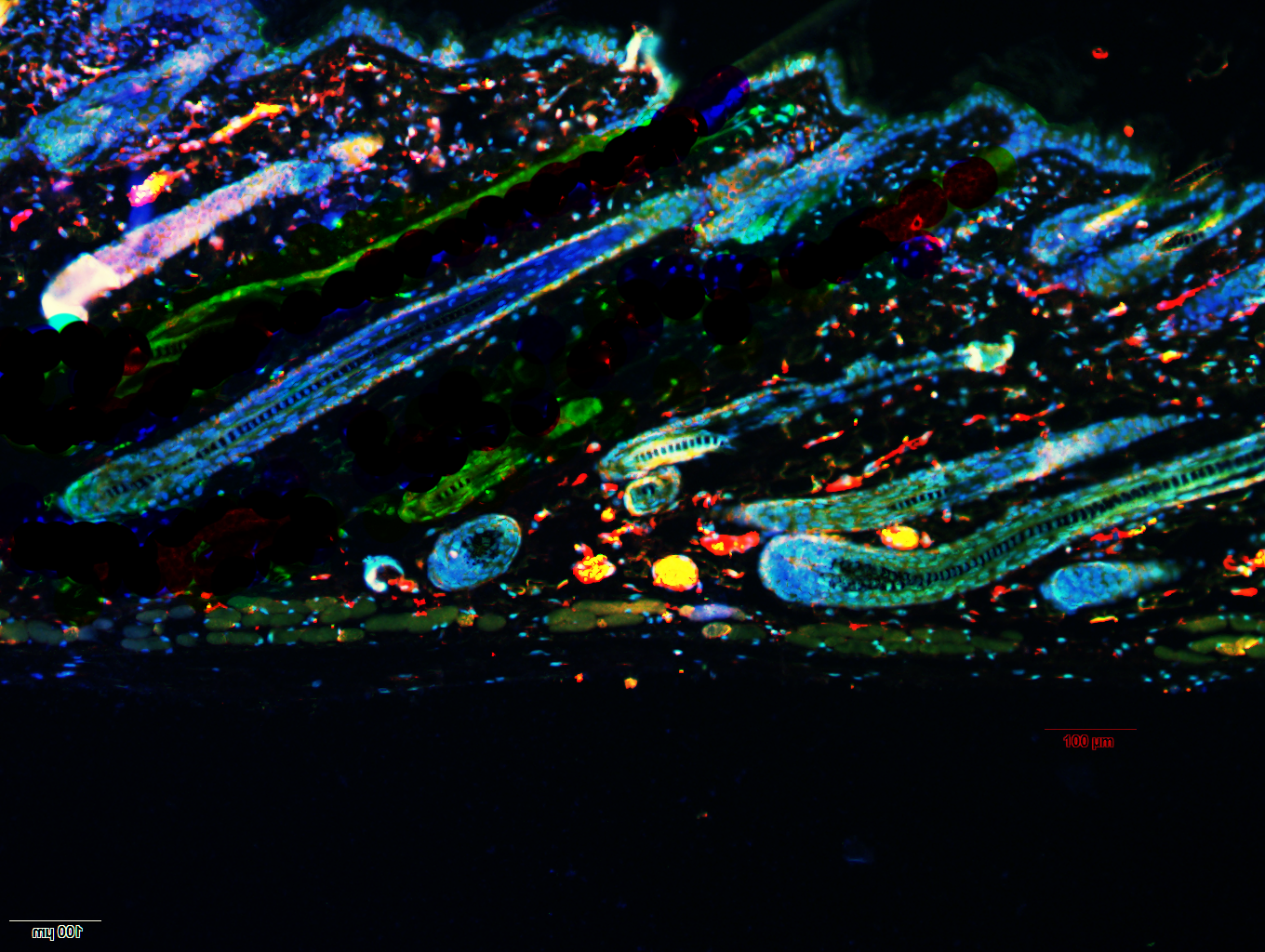

Supplement: Supplementary file 7 — Source data Fig. 6 [file 44319_2024_327_MOESM7_ESM.zip › Figure 6/6A/1 (3).tif]

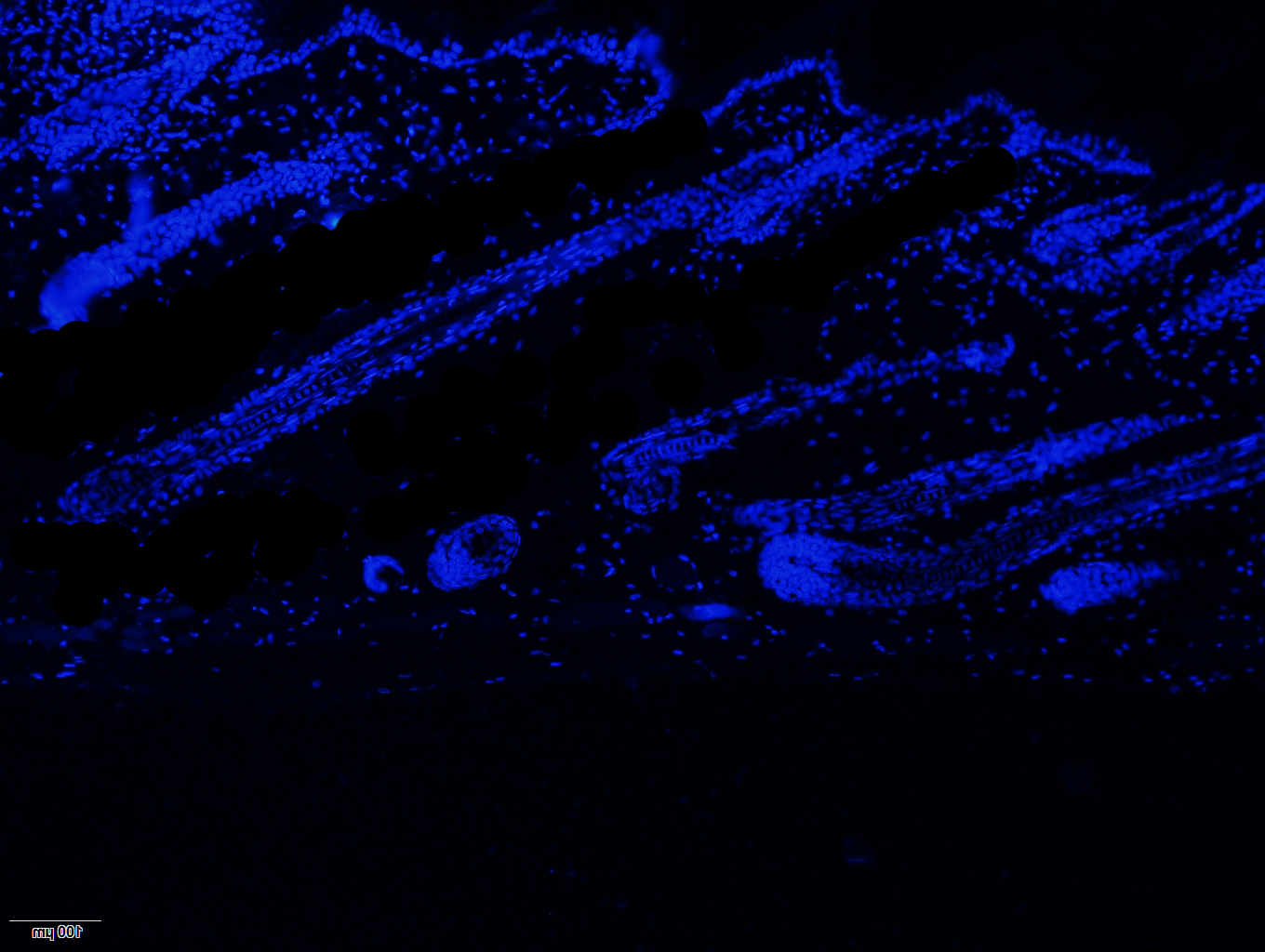

Supplement: Supplementary file 7 — Source data Fig. 6 [file 44319_2024_327_MOESM7_ESM.zip › Figure 6/6A/1 (4).tif]

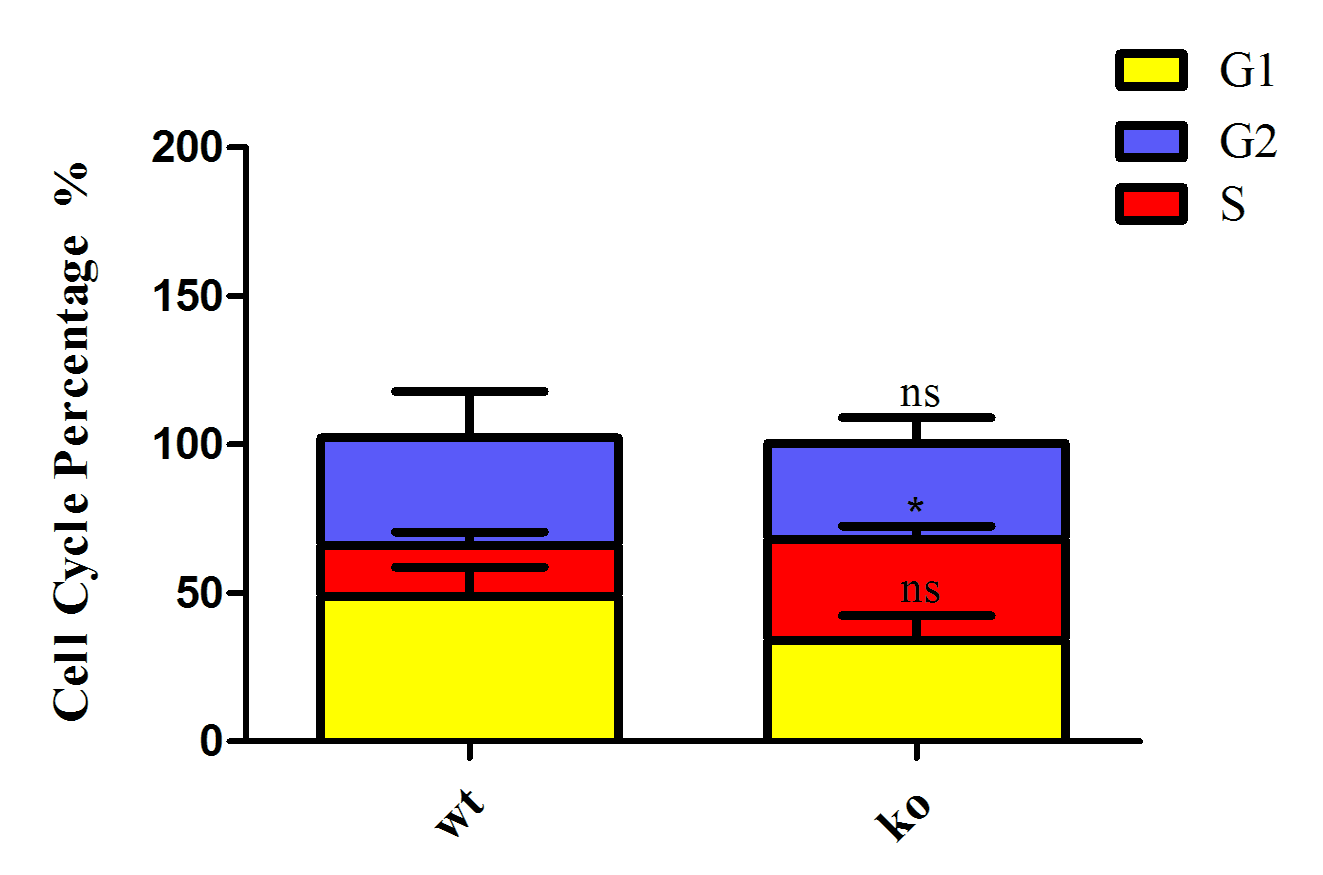

Supplement: Supplementary file 7 — Source data Fig. 6 [file 44319_2024_327_MOESM7_ESM.zip › Figure 6/6D/Statistical Analysis/Cell cycle percentage%.tif]

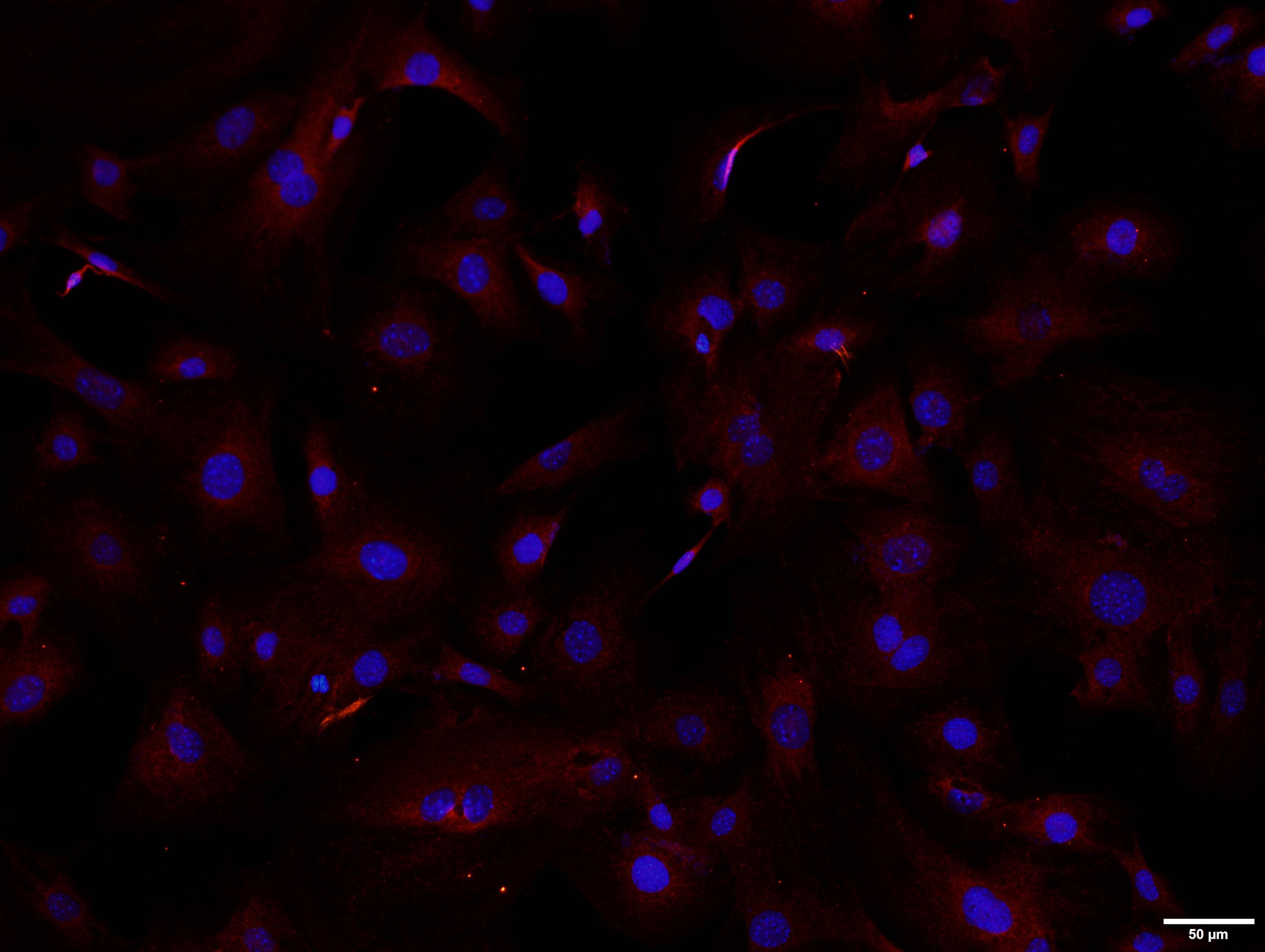

Supplement: Supplementary file 8 — Source data Fig. 7 [file 44319_2024_327_MOESM8_ESM.zip › Figure 7/7A/Control/1 (1).jpg]
