# Supplementary figures and images for: Gpr54 deletion accelerates hair cycle and hair regeneration (part 2 of 2)
Source: EMBO Rep. 2024 Nov 25;26(1):200–17. doi: 10.1038/s44319-024-00327-y (PMC11724127; doi:10.1038/s44319-024-00327-y)

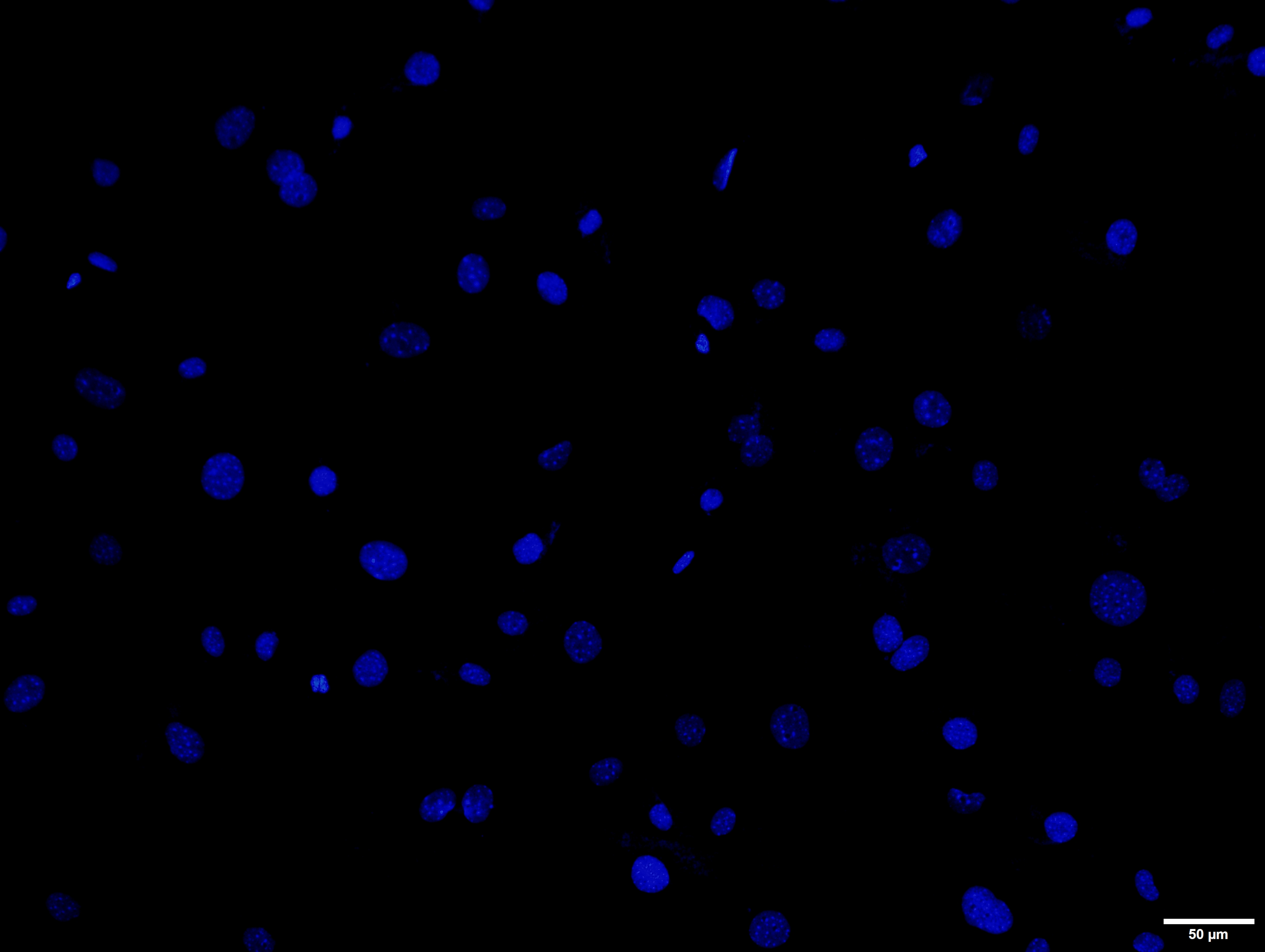

Supplement: Supplementary file 8 — Source data Fig. 7 [file 44319_2024_327_MOESM8_ESM.zip › Figure 7/7A/Control/1 (2).jpg]

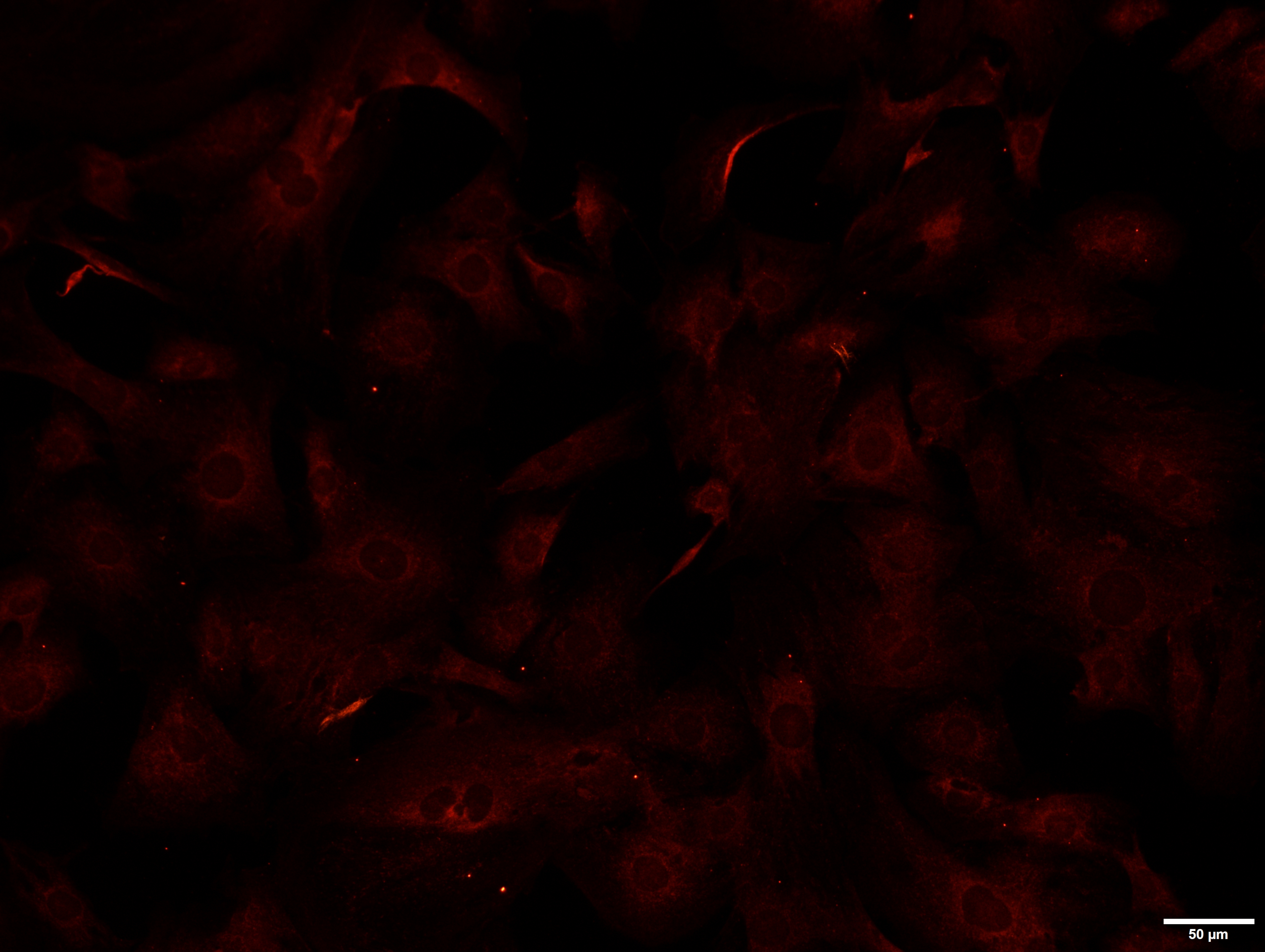

Supplement: Supplementary file 8 — Source data Fig. 7 [file 44319_2024_327_MOESM8_ESM.zip › Figure 7/7A/Control/1 (3).jpg]

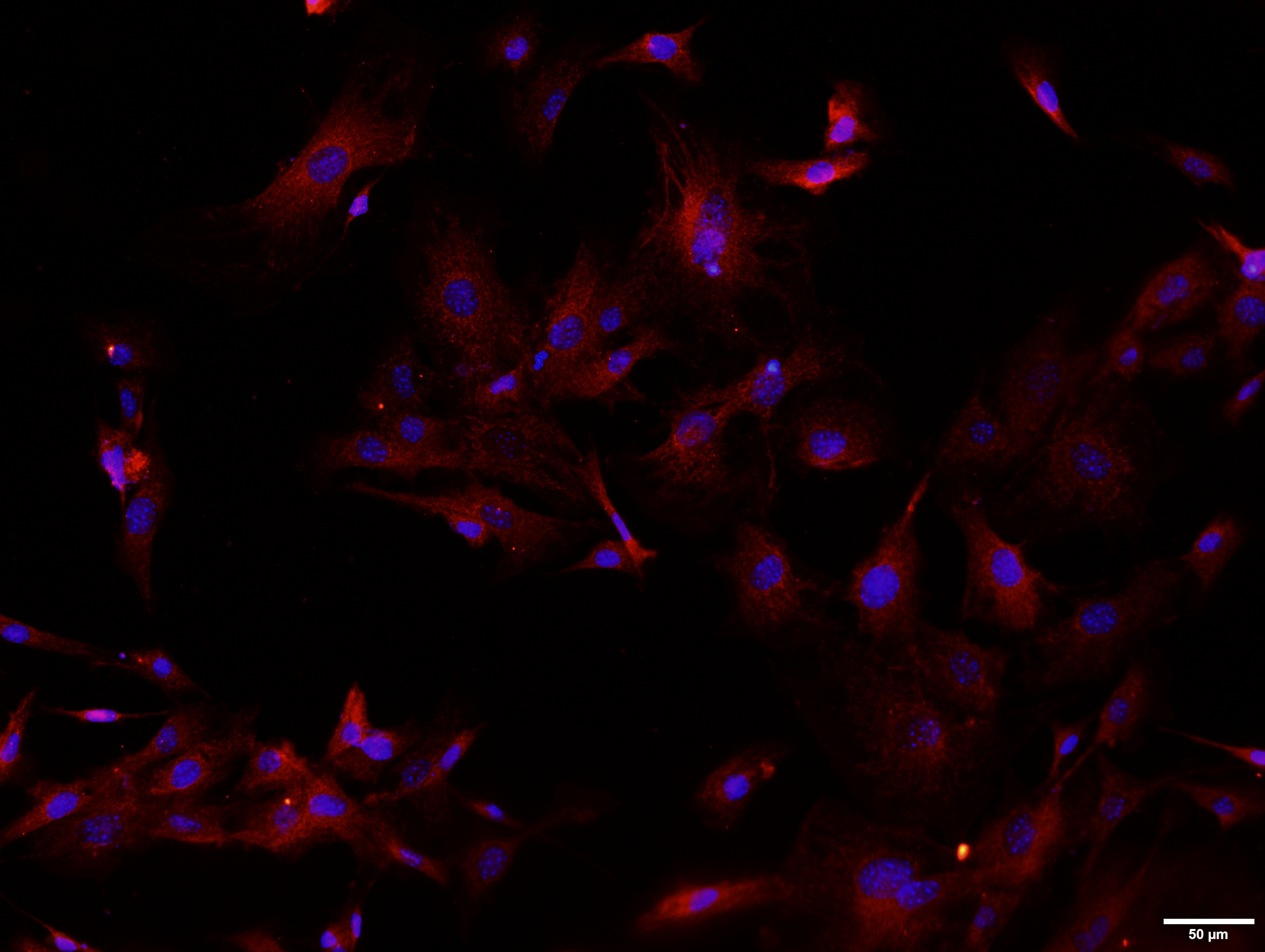

Supplement: Supplementary file 8 — Source data Fig. 7 [file 44319_2024_327_MOESM8_ESM.zip › Figure 7/7A/kp234-0.25μM/1 (1).jpg]

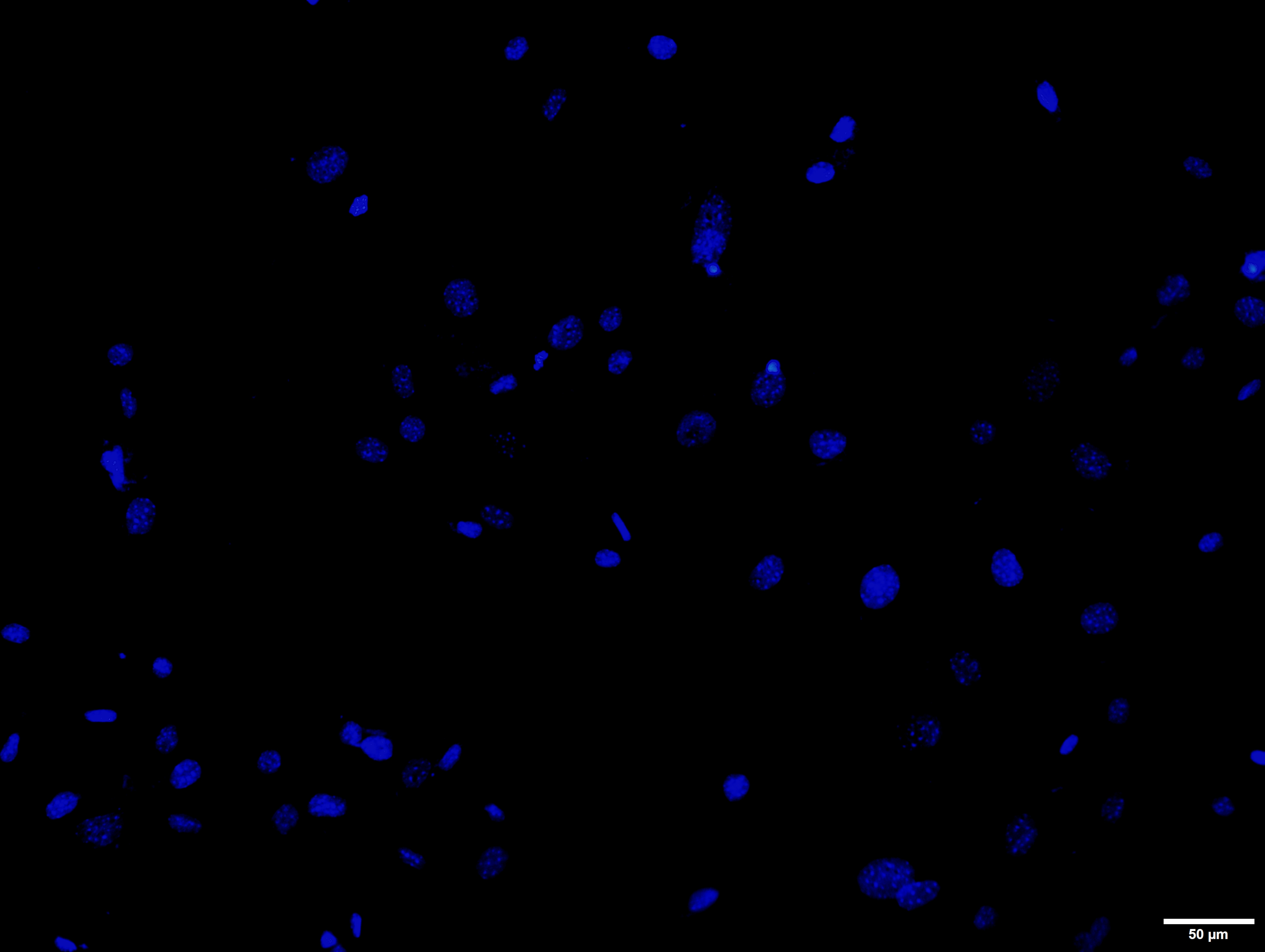

Supplement: Supplementary file 8 — Source data Fig. 7 [file 44319_2024_327_MOESM8_ESM.zip › Figure 7/7A/kp234-0.25μM/1 (2).jpg]

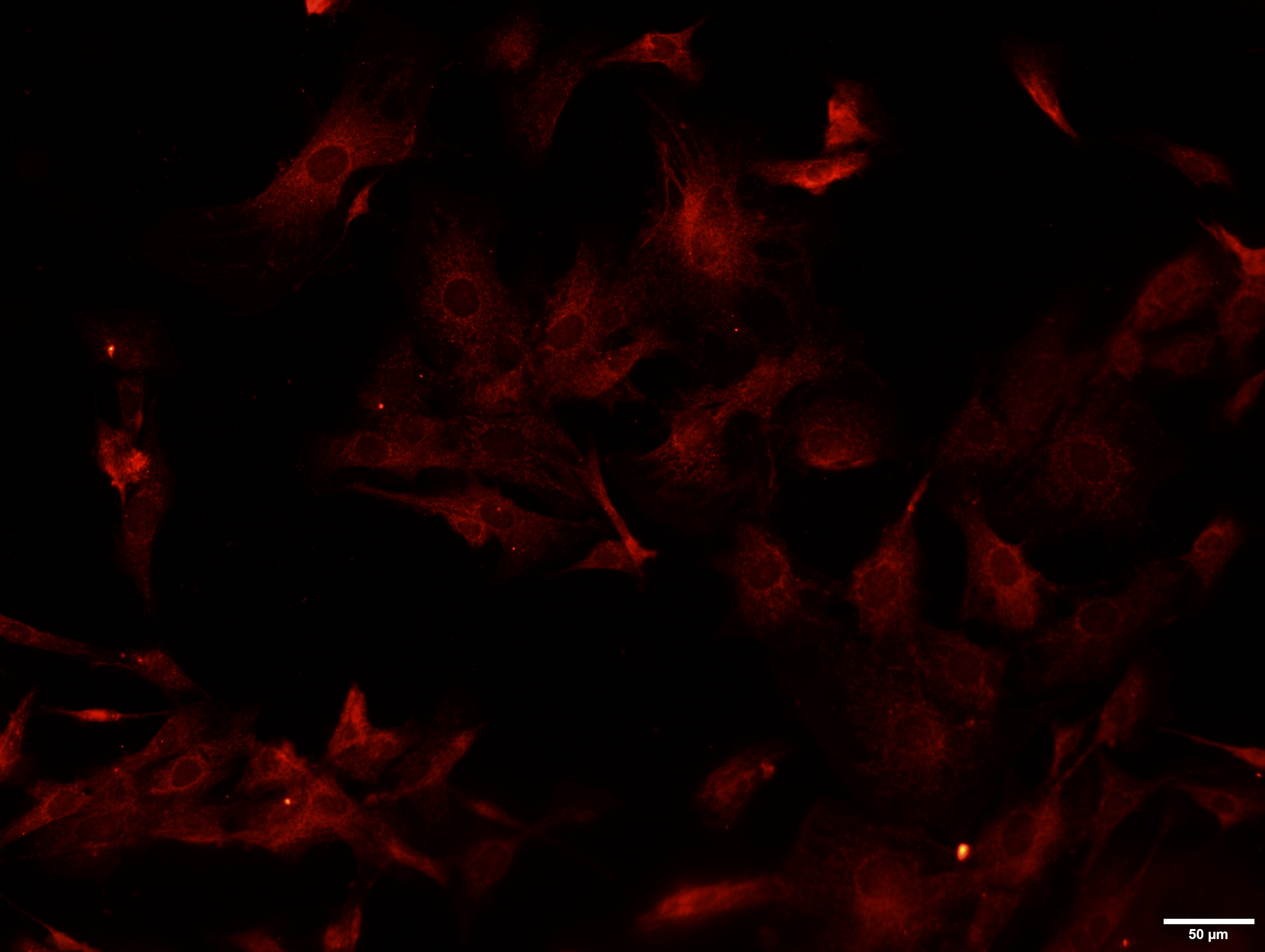

Supplement: Supplementary file 8 — Source data Fig. 7 [file 44319_2024_327_MOESM8_ESM.zip › Figure 7/7A/kp234-0.25μM/1 (3).jpg]

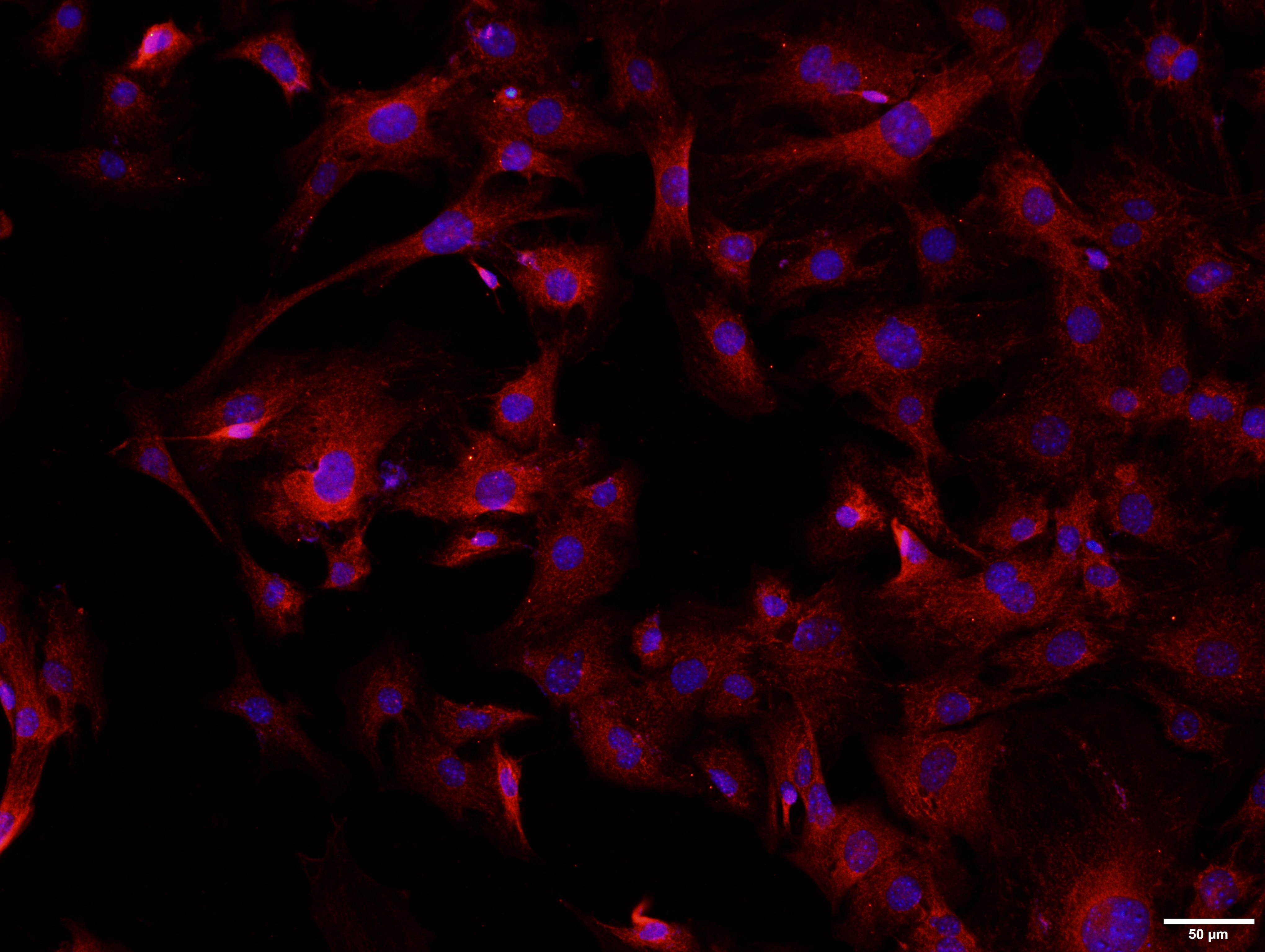

Supplement: Supplementary file 8 — Source data Fig. 7 [file 44319_2024_327_MOESM8_ESM.zip › Figure 7/7A/kp234-0.5μM/1 (1).jpg]

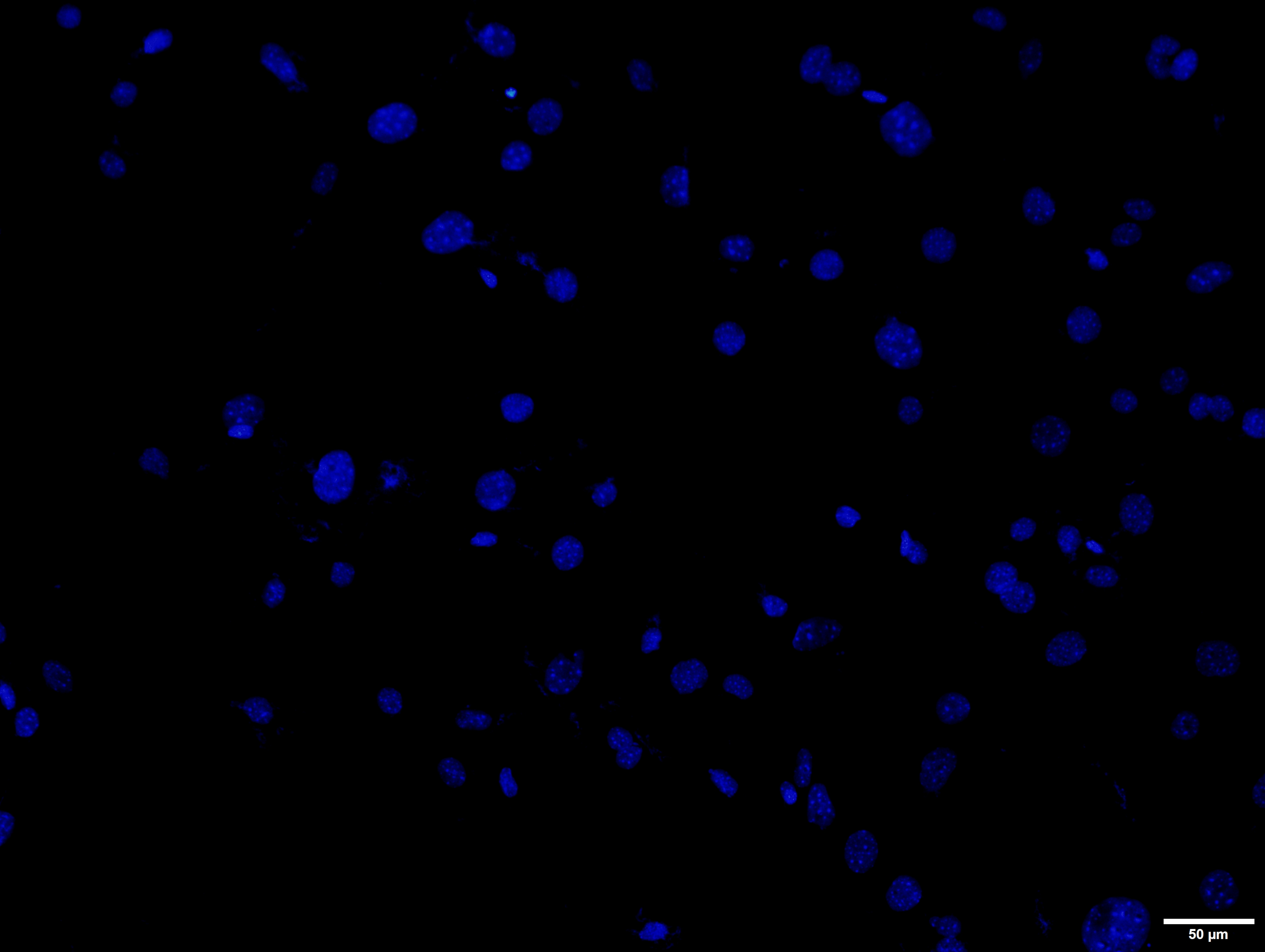

Supplement: Supplementary file 8 — Source data Fig. 7 [file 44319_2024_327_MOESM8_ESM.zip › Figure 7/7A/kp234-0.5μM/1 (2).jpg]

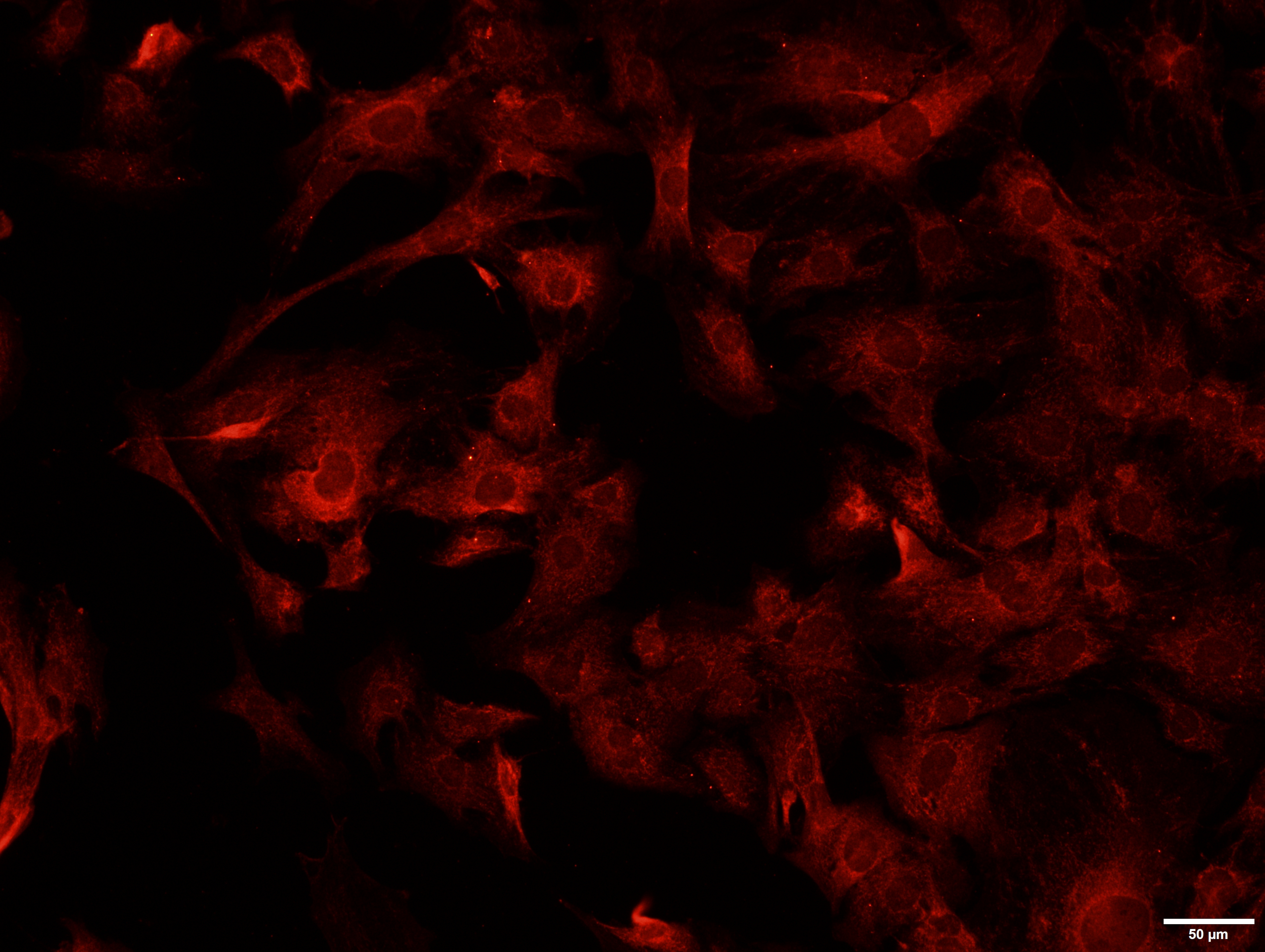

Supplement: Supplementary file 8 — Source data Fig. 7 [file 44319_2024_327_MOESM8_ESM.zip › Figure 7/7A/kp234-0.5μM/1 (3).jpg]

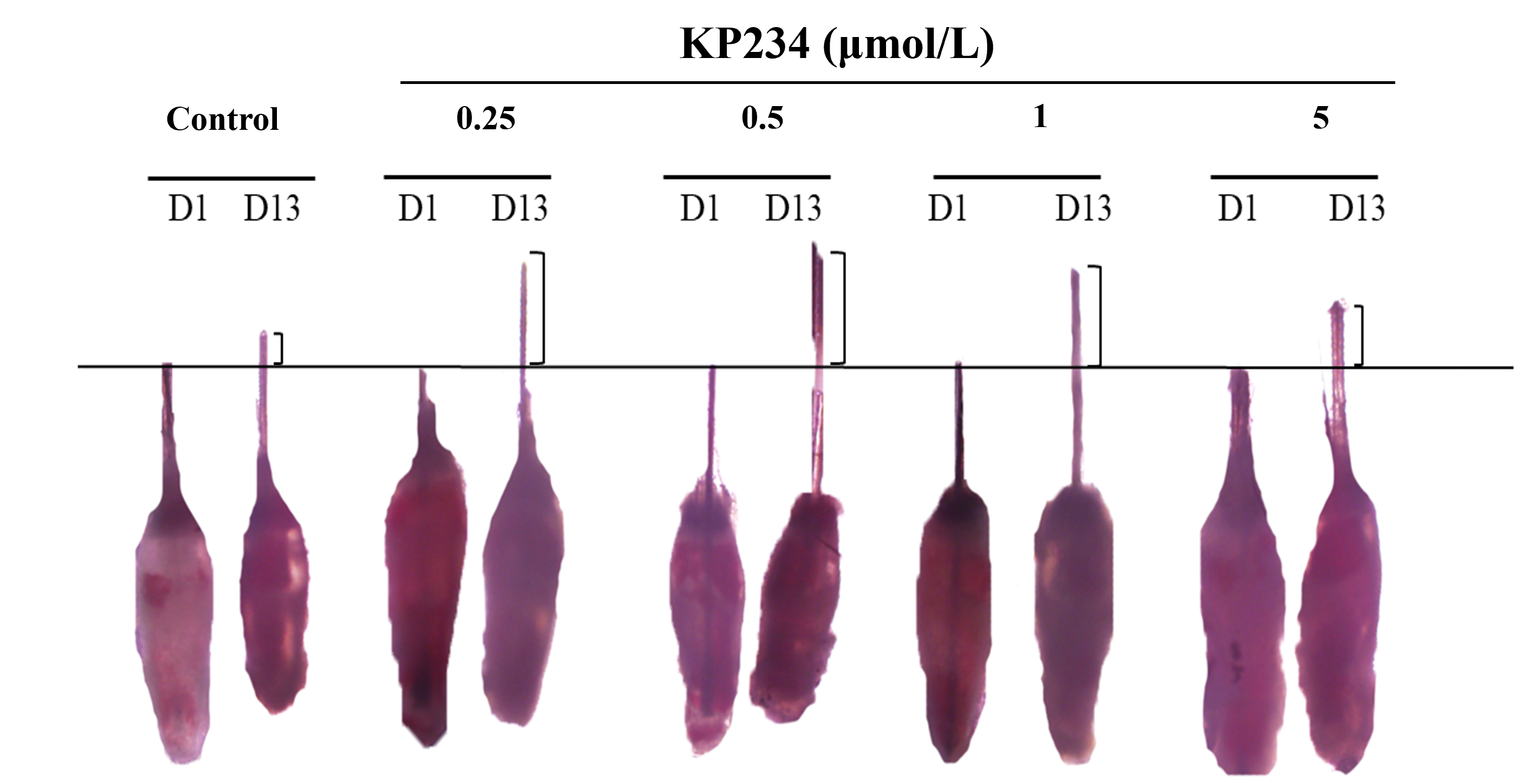

Supplement: Supplementary file 8 — Source data Fig. 7 [file 44319_2024_327_MOESM8_ESM.zip › Figure 7/7C/1.tif]

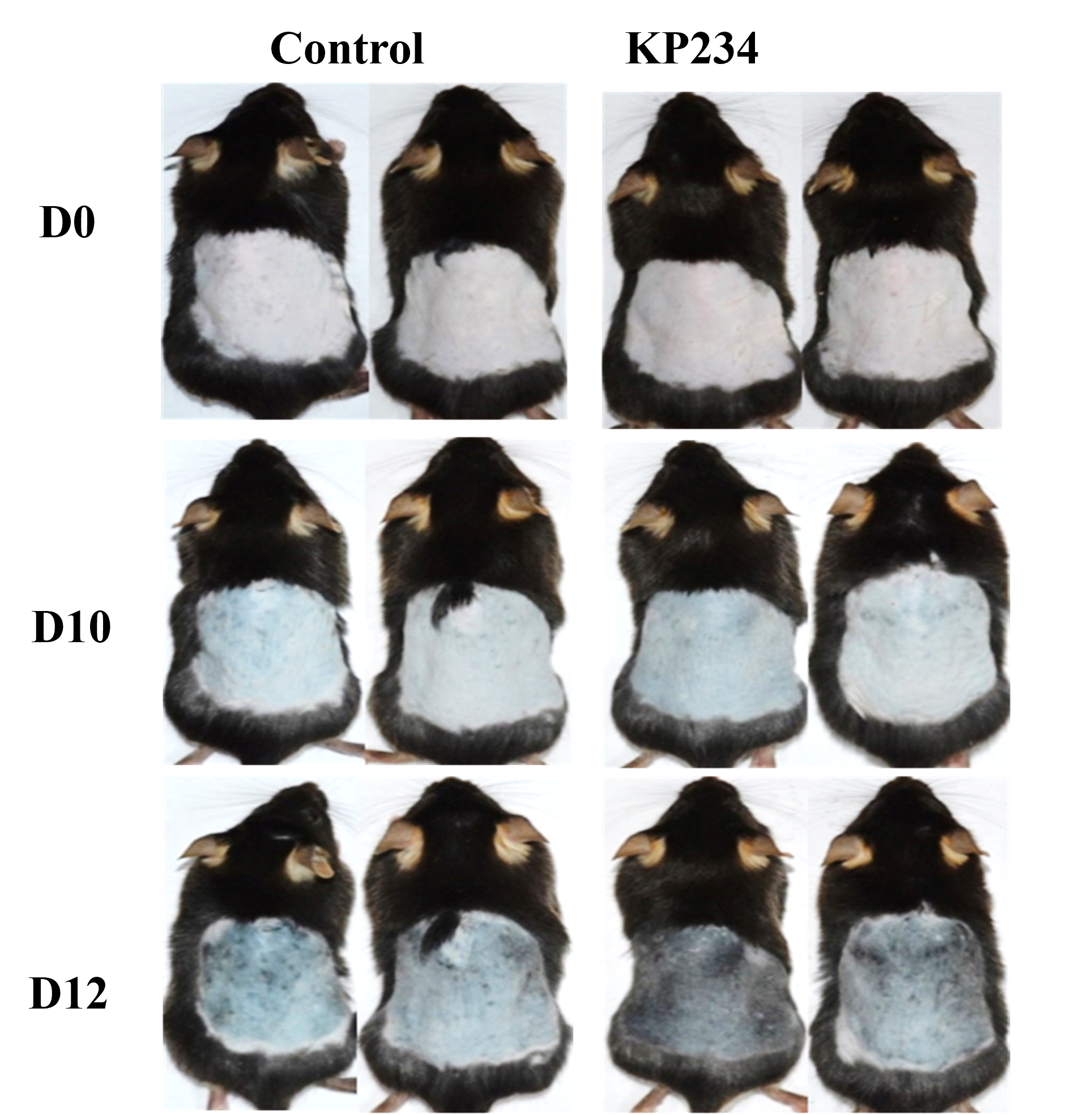

Supplement: Supplementary file 8 — Source data Fig. 7 [file 44319_2024_327_MOESM8_ESM.zip › Figure 7/7E/1.tif]

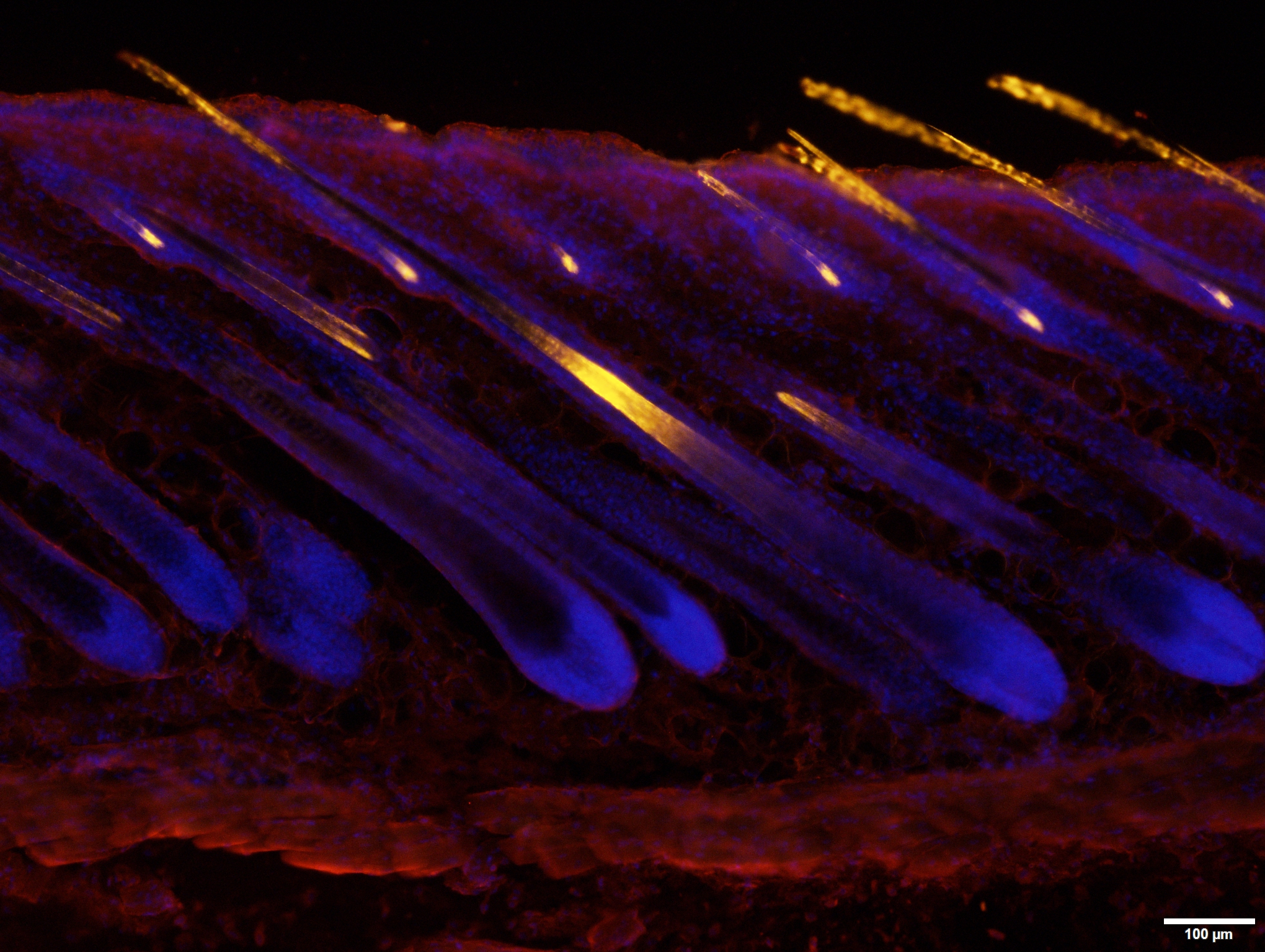

Supplement: Supplementary file 9 — EV Figures Source Data [file 44319_2024_327_MOESM9_ESM.zip › source data-Supplemental Figures/Figure EV1/EV1A/1 (1).jpg]

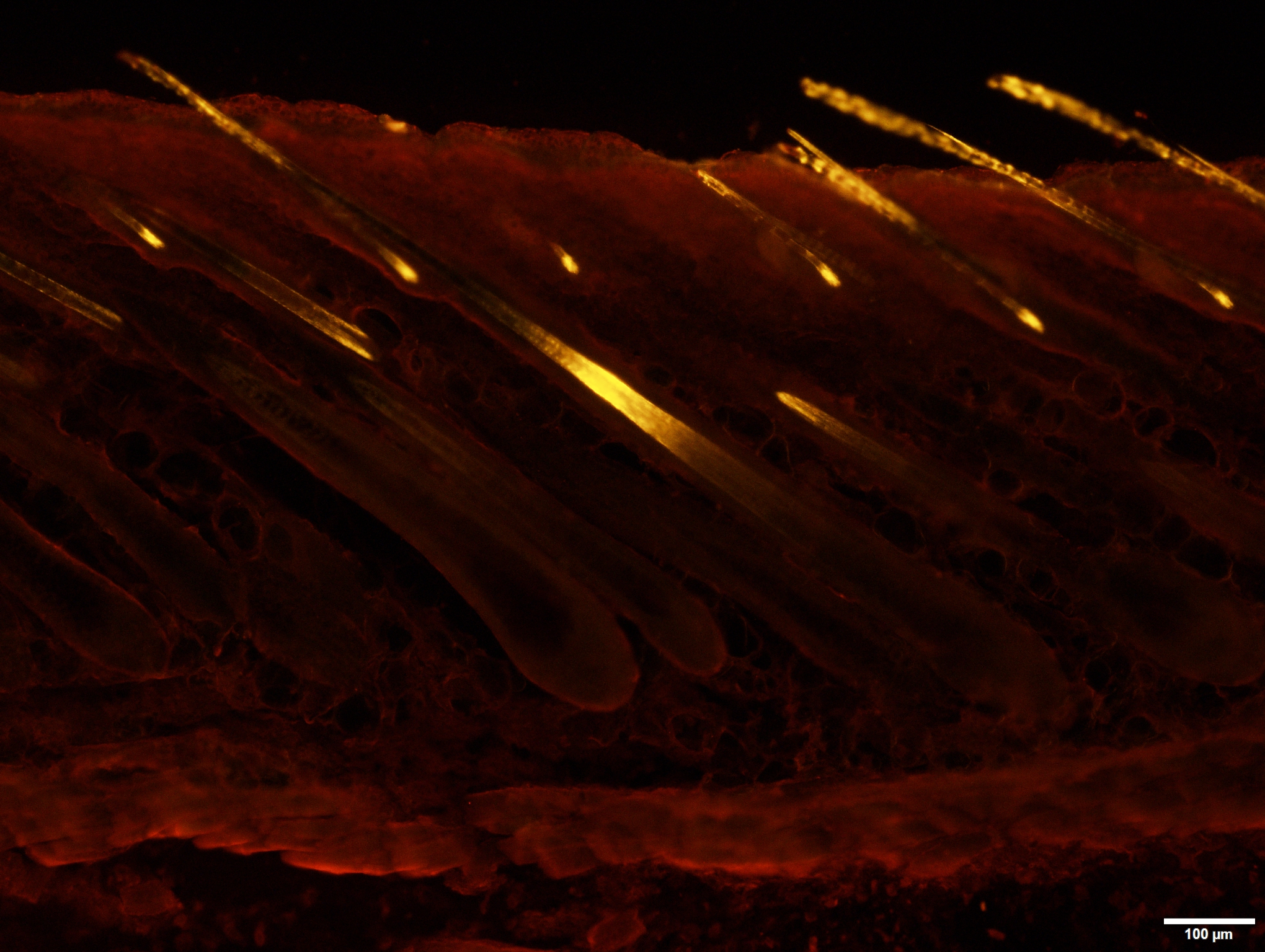

Supplement: Supplementary file 9 — EV Figures Source Data [file 44319_2024_327_MOESM9_ESM.zip › source data-Supplemental Figures/Figure EV1/EV1A/1 (2).jpg]

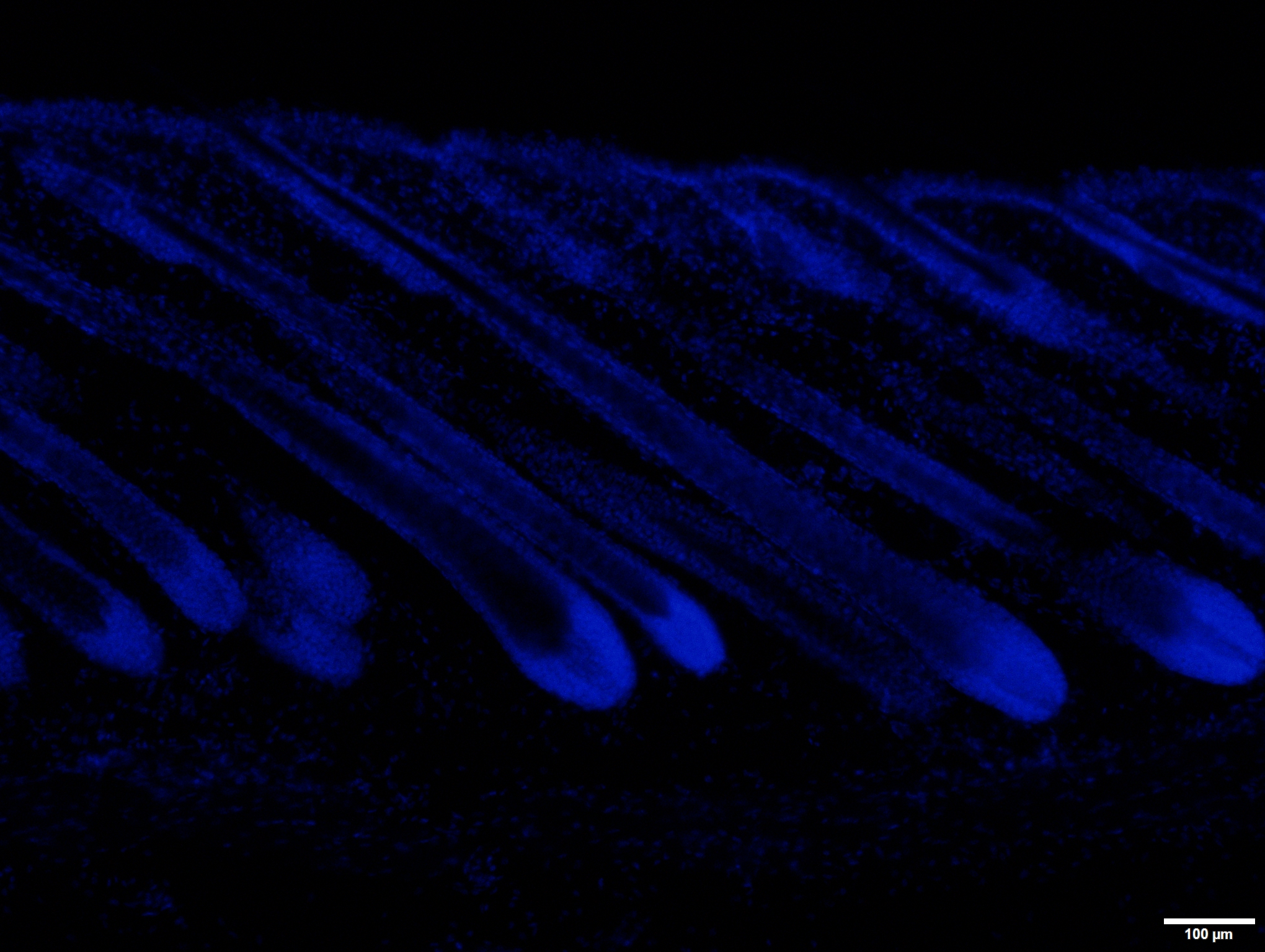

Supplement: Supplementary file 9 — EV Figures Source Data [file 44319_2024_327_MOESM9_ESM.zip › source data-Supplemental Figures/Figure EV1/EV1A/1 (3).jpg]

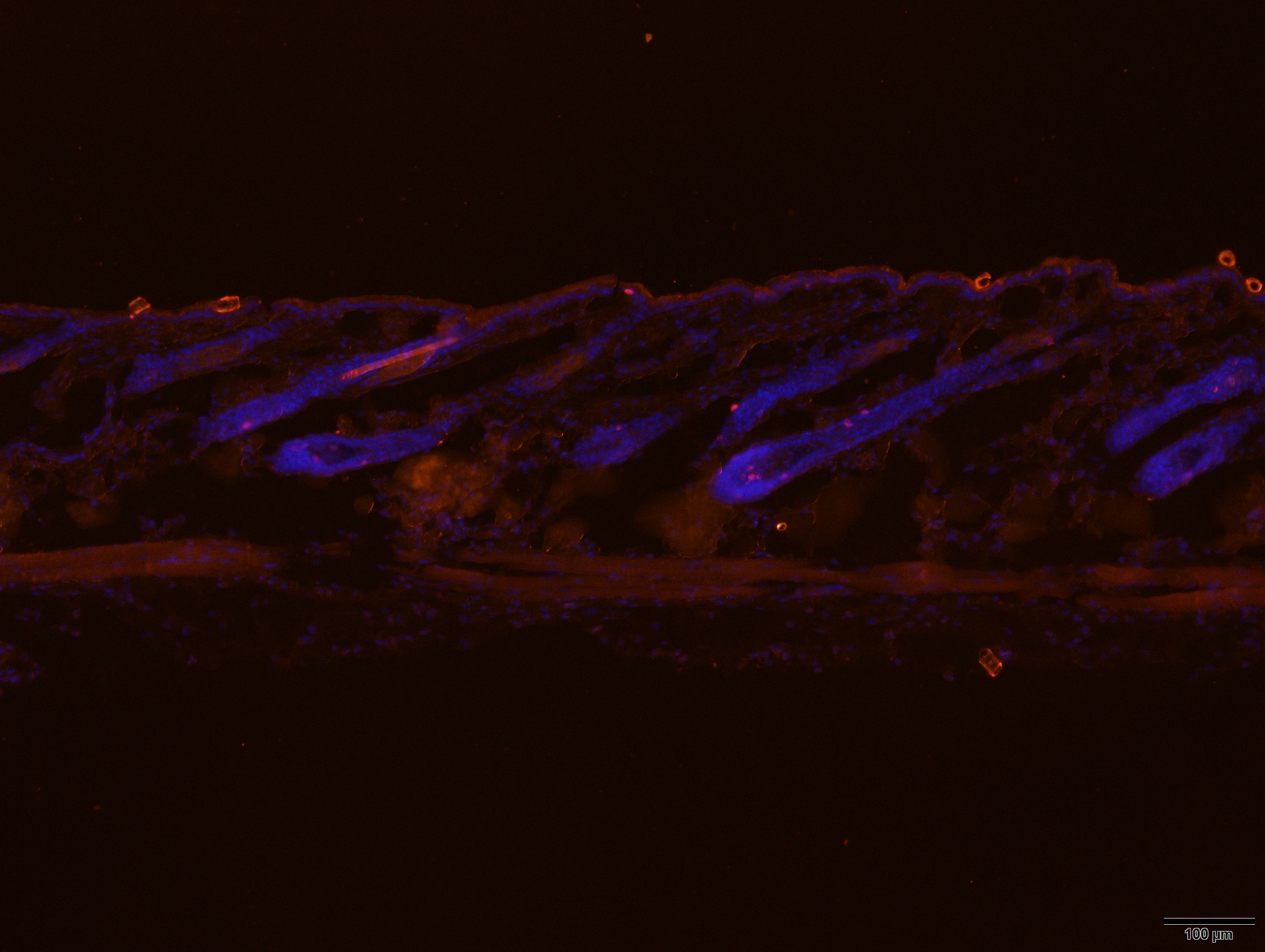

Supplement: Supplementary file 9 — EV Figures Source Data [file 44319_2024_327_MOESM9_ESM.zip › source data-Supplemental Figures/Figure EV1/EV1B/Day23-KI67/KO/1 (1).jpg]

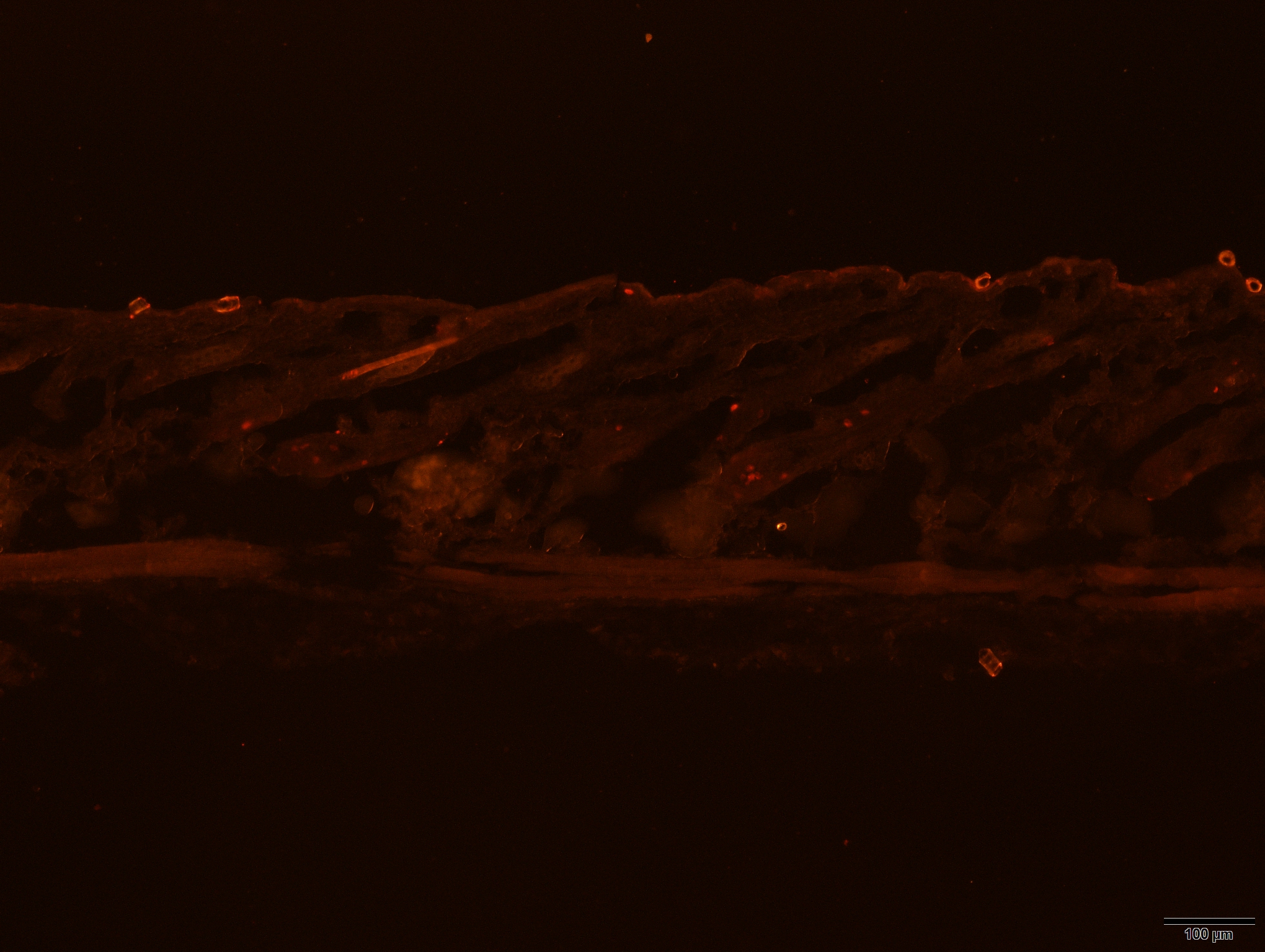

Supplement: Supplementary file 9 — EV Figures Source Data [file 44319_2024_327_MOESM9_ESM.zip › source data-Supplemental Figures/Figure EV1/EV1B/Day23-KI67/KO/1 (2).jpg]

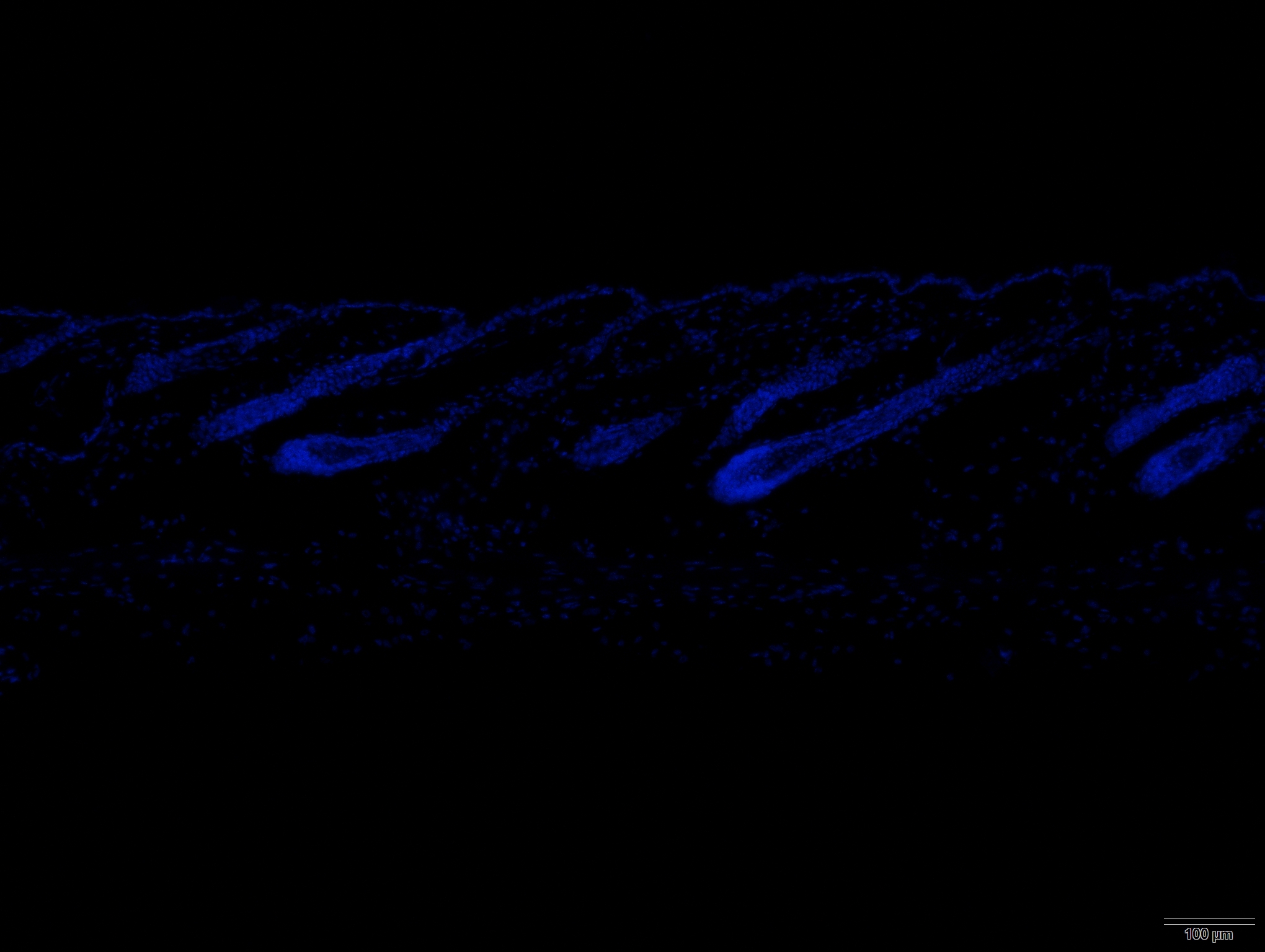

Supplement: Supplementary file 9 — EV Figures Source Data [file 44319_2024_327_MOESM9_ESM.zip › source data-Supplemental Figures/Figure EV1/EV1B/Day23-KI67/KO/1 (3).jpg]

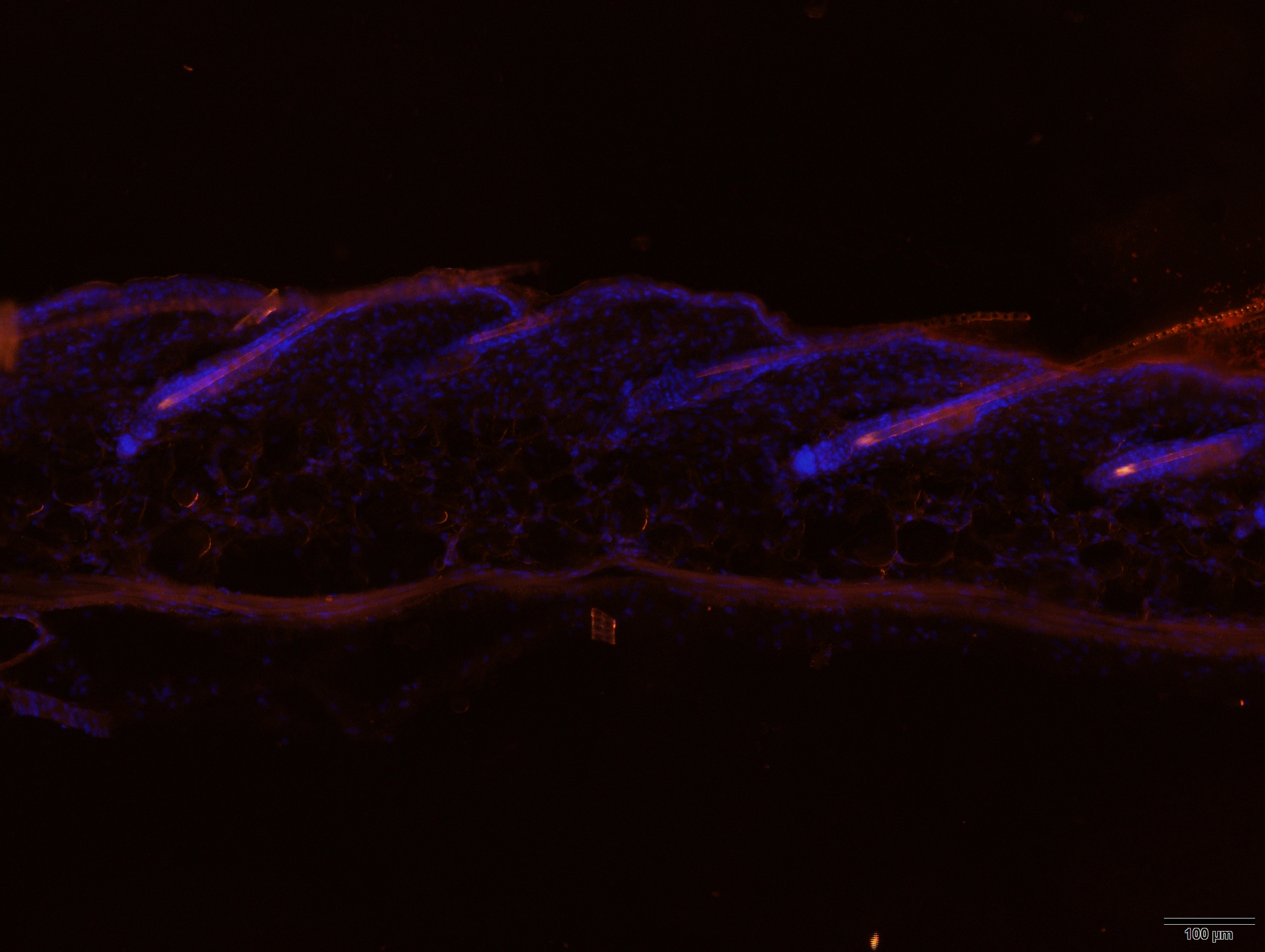

Supplement: Supplementary file 9 — EV Figures Source Data [file 44319_2024_327_MOESM9_ESM.zip › source data-Supplemental Figures/Figure EV1/EV1B/Day23-KI67/WT/1 (1).jpg]

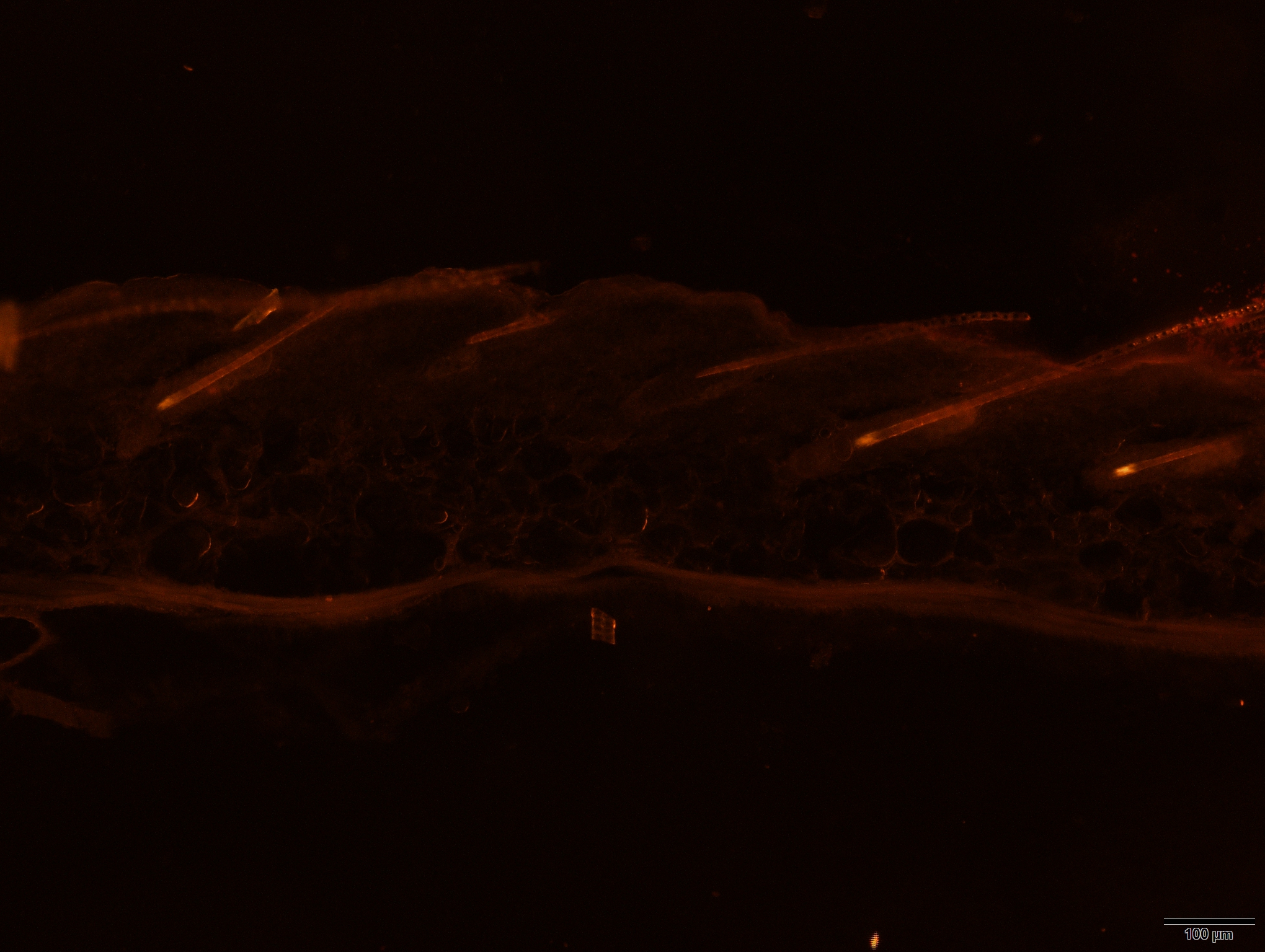

Supplement: Supplementary file 9 — EV Figures Source Data [file 44319_2024_327_MOESM9_ESM.zip › source data-Supplemental Figures/Figure EV1/EV1B/Day23-KI67/WT/1 (2).jpg]

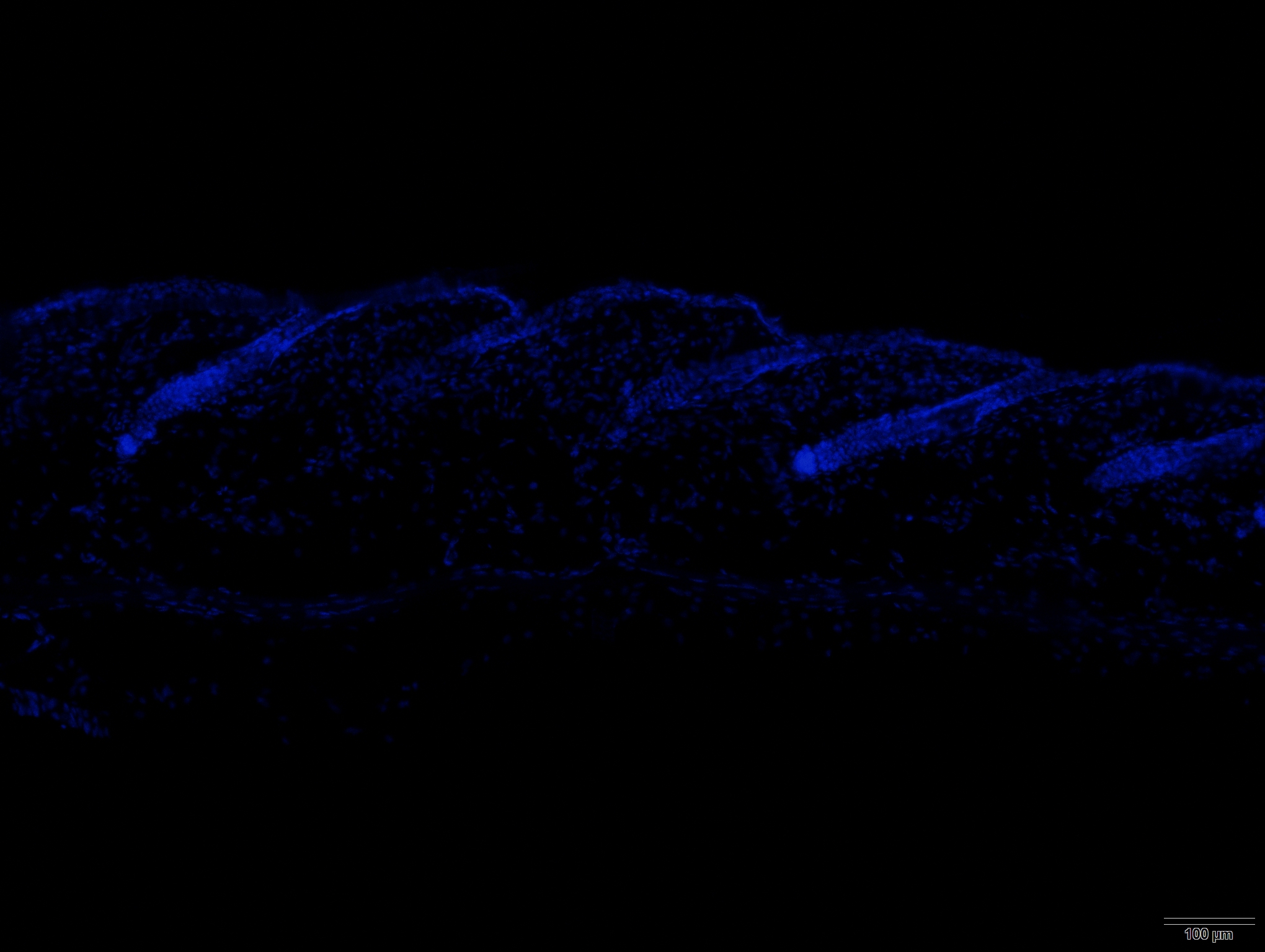

Supplement: Supplementary file 9 — EV Figures Source Data [file 44319_2024_327_MOESM9_ESM.zip › source data-Supplemental Figures/Figure EV1/EV1B/Day23-KI67/WT/1 (3).jpg]

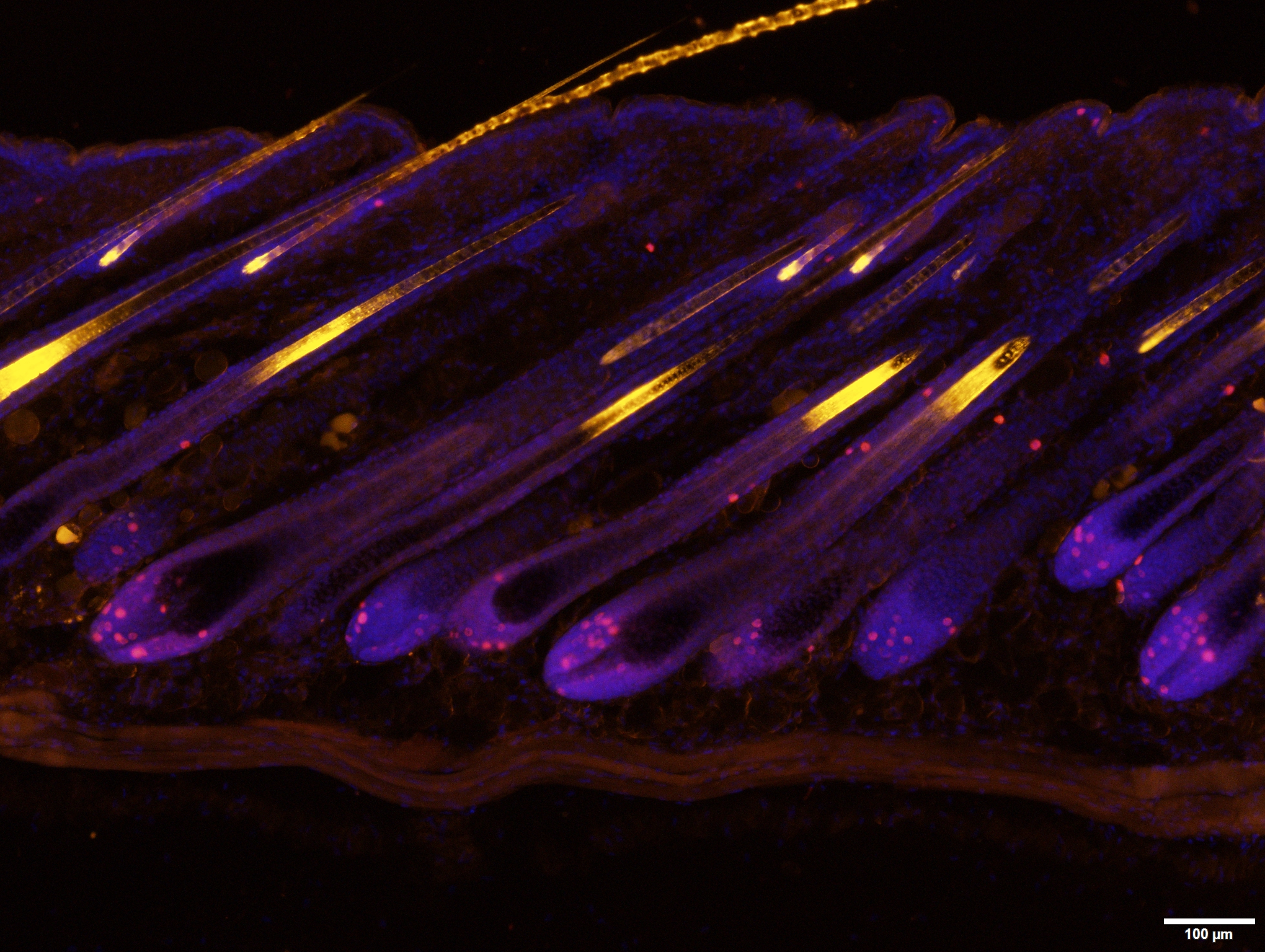

Supplement: Supplementary file 9 — EV Figures Source Data [file 44319_2024_327_MOESM9_ESM.zip › source data-Supplemental Figures/Figure EV1/EV1B/Day25-KI67/KO/1 (1).jpg]

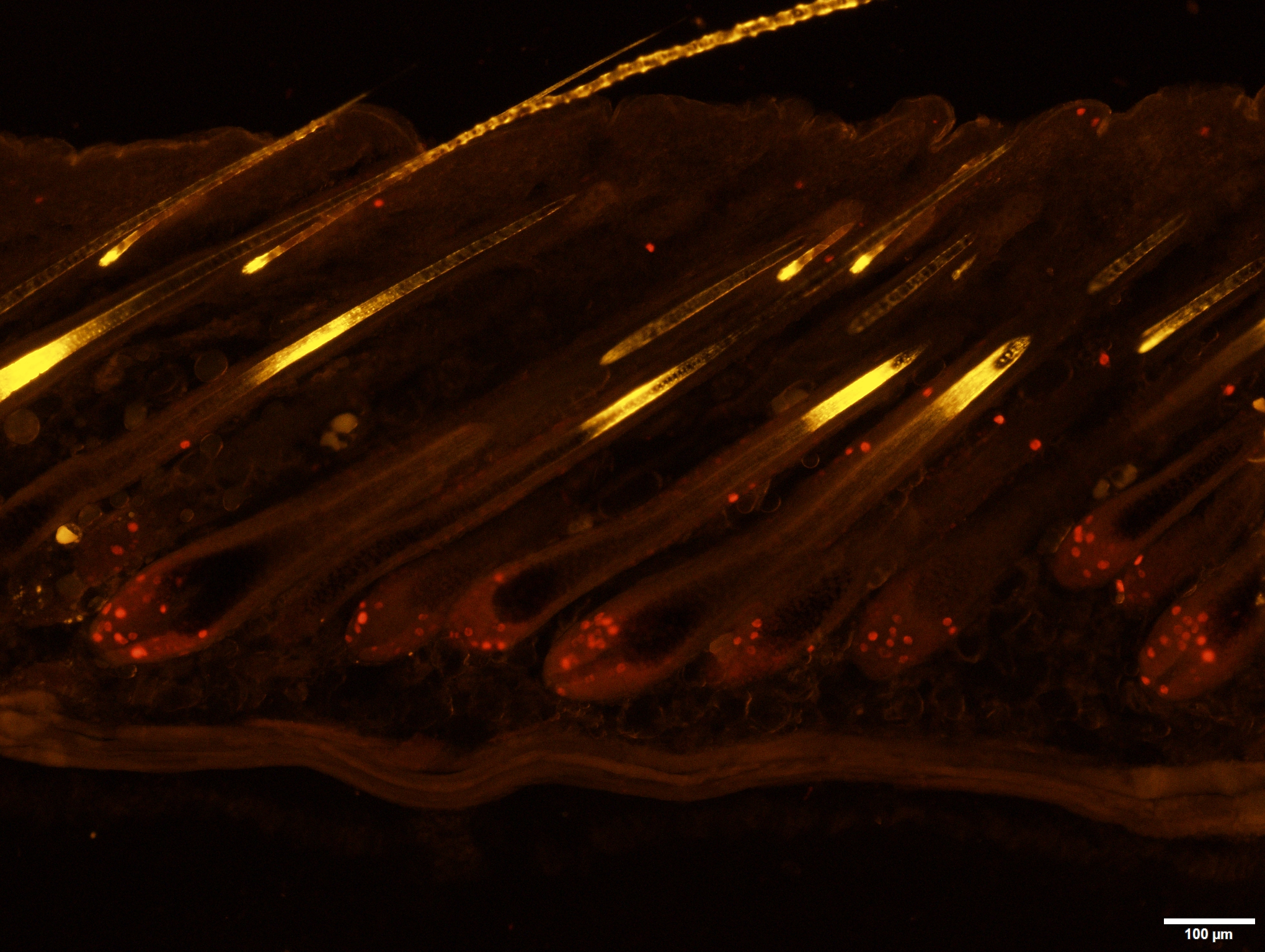

Supplement: Supplementary file 9 — EV Figures Source Data [file 44319_2024_327_MOESM9_ESM.zip › source data-Supplemental Figures/Figure EV1/EV1B/Day25-KI67/KO/1 (2).jpg]

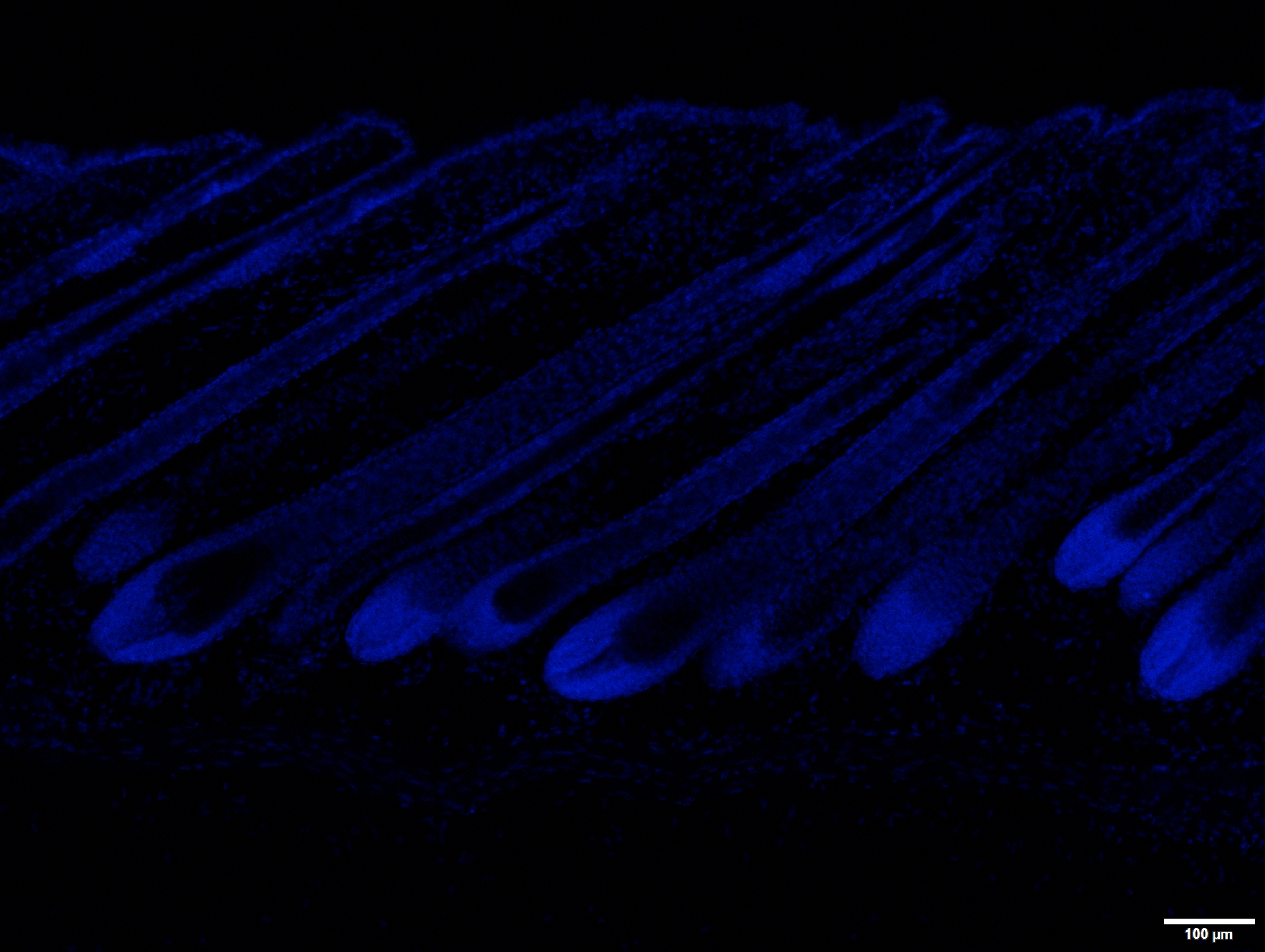

Supplement: Supplementary file 9 — EV Figures Source Data [file 44319_2024_327_MOESM9_ESM.zip › source data-Supplemental Figures/Figure EV1/EV1B/Day25-KI67/KO/1 (3).jpg]

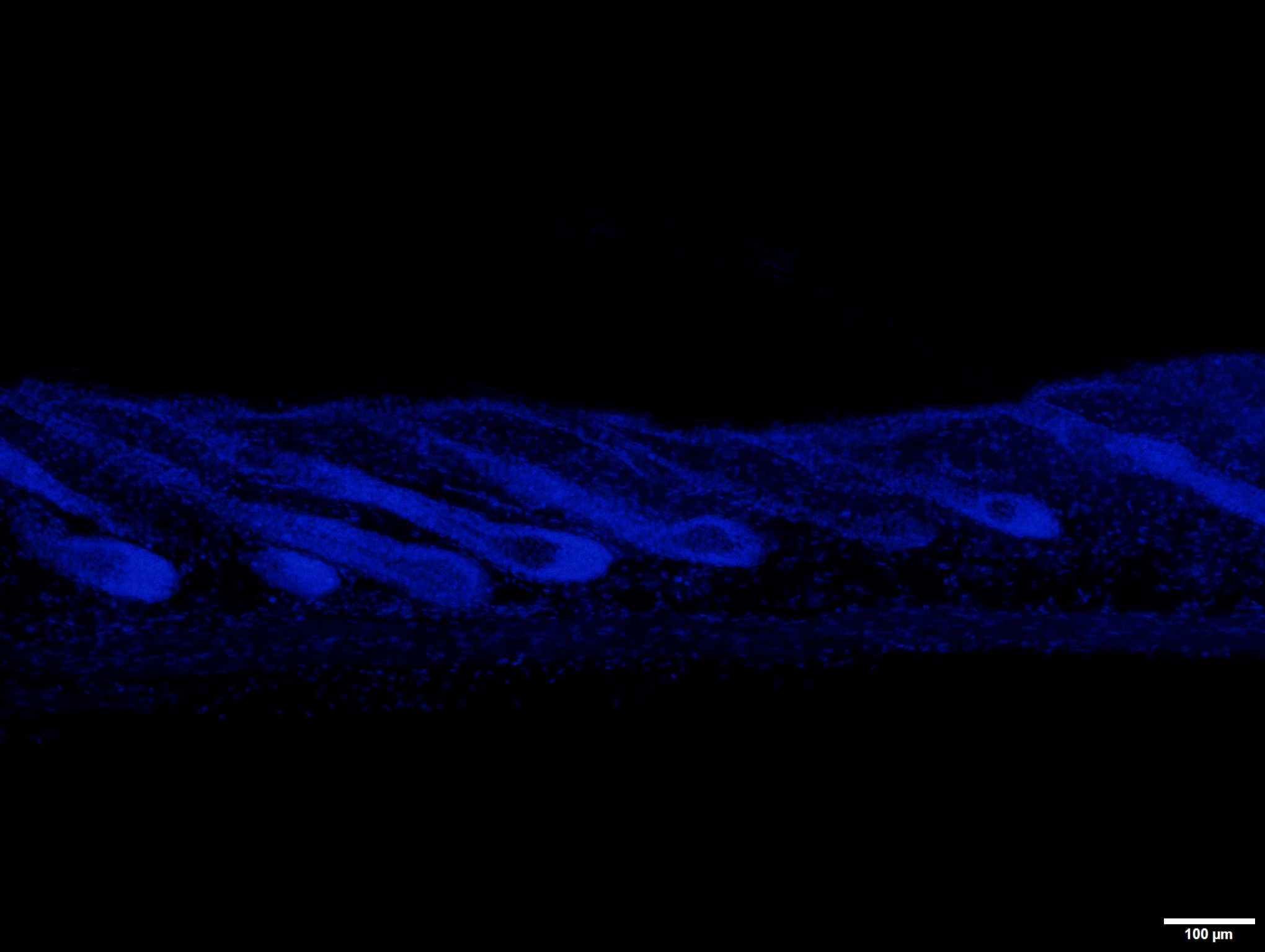

Supplement: Supplementary file 9 — EV Figures Source Data [file 44319_2024_327_MOESM9_ESM.zip › source data-Supplemental Figures/Figure EV1/EV1B/Day25-KI67/WT/1 (1).jpg]

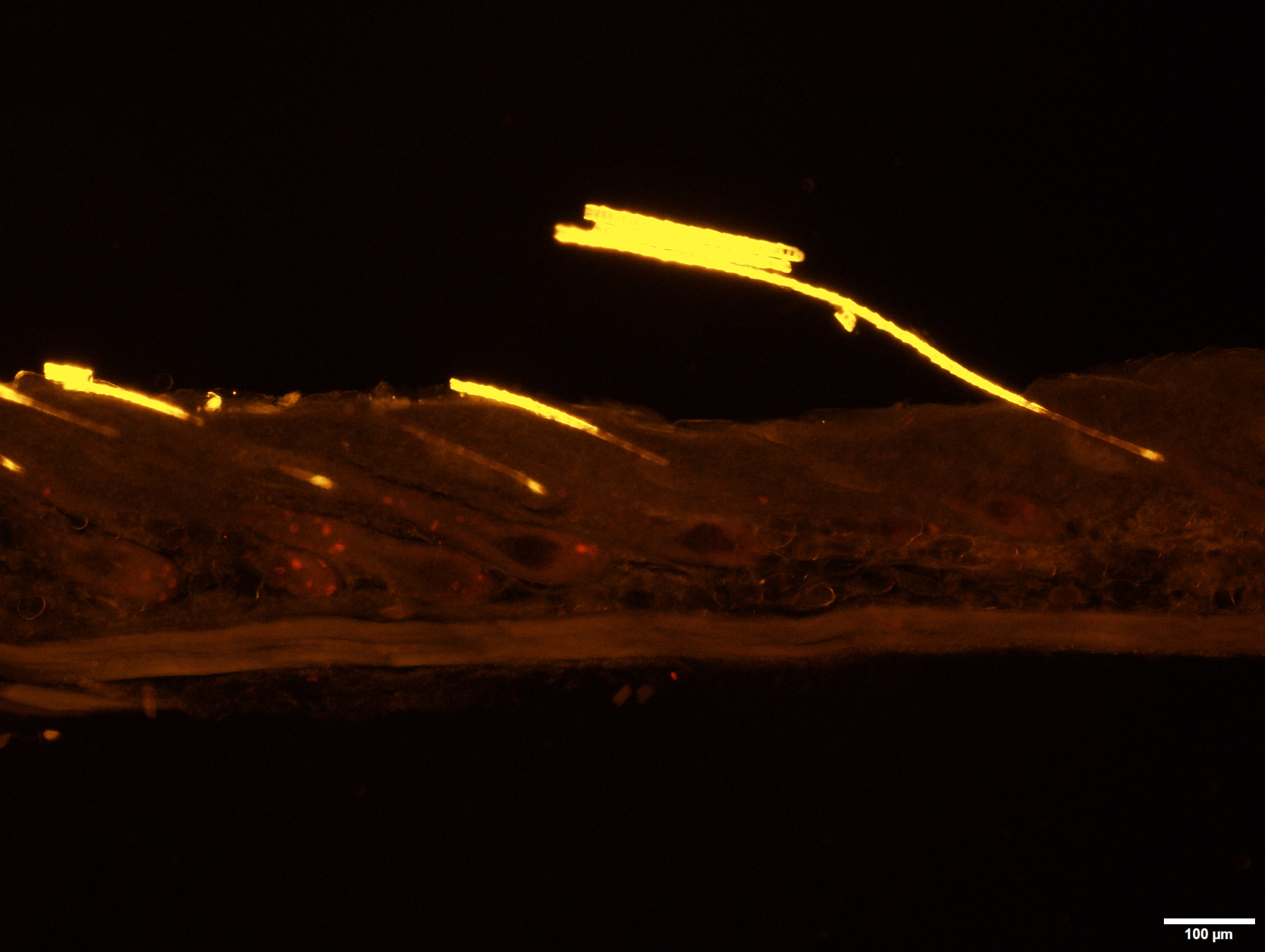

Supplement: Supplementary file 9 — EV Figures Source Data [file 44319_2024_327_MOESM9_ESM.zip › source data-Supplemental Figures/Figure EV1/EV1B/Day25-KI67/WT/1 (2).jpg]

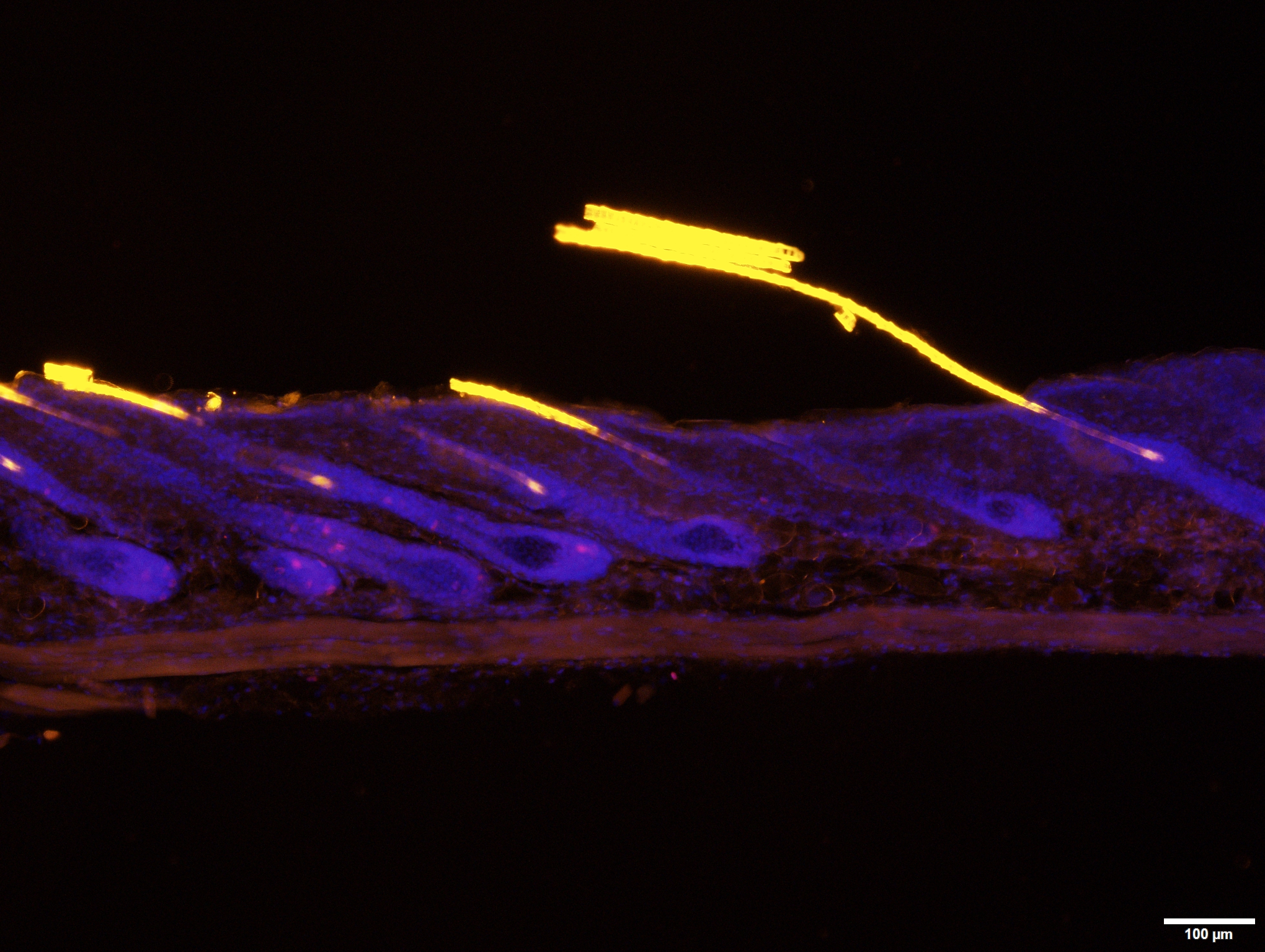

Supplement: Supplementary file 9 — EV Figures Source Data [file 44319_2024_327_MOESM9_ESM.zip › source data-Supplemental Figures/Figure EV1/EV1B/Day25-KI67/WT/1 (3).jpg]

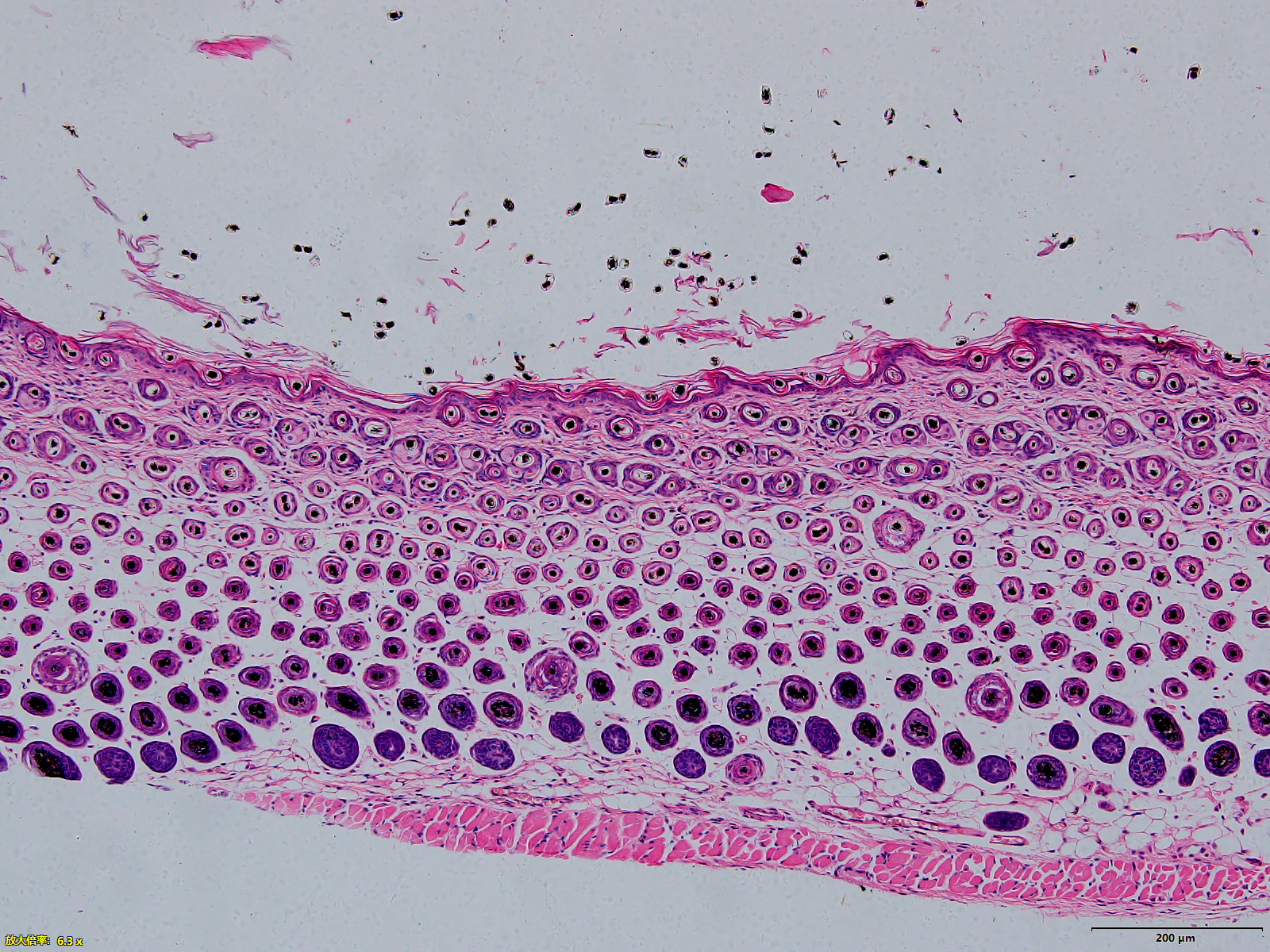

Supplement: Supplementary file 9 — EV Figures Source Data [file 44319_2024_327_MOESM9_ESM.zip › source data-Supplemental Figures/Figure EV1/EV1D/Anagen/KO/KO.tif]

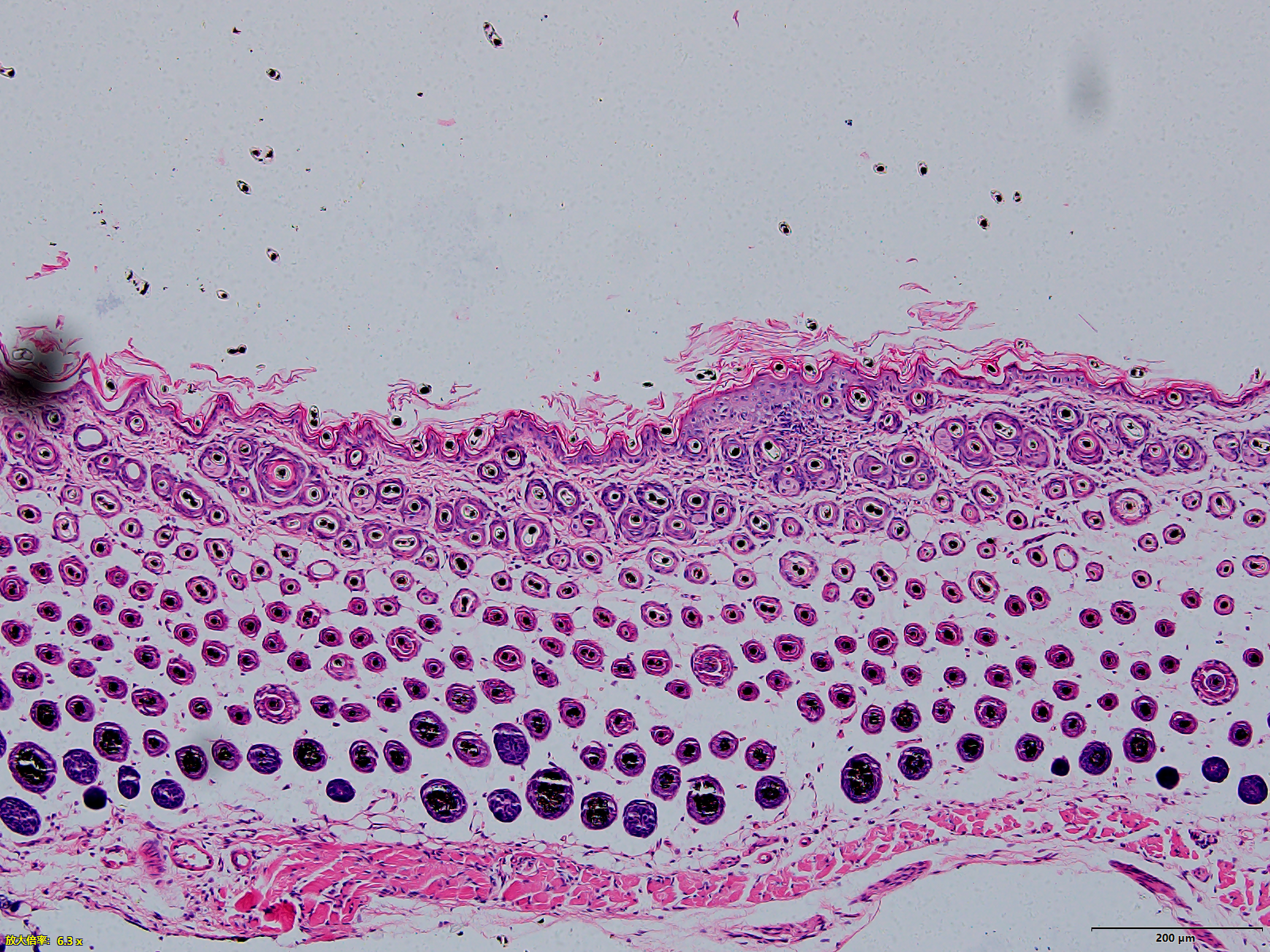

Supplement: Supplementary file 9 — EV Figures Source Data [file 44319_2024_327_MOESM9_ESM.zip › source data-Supplemental Figures/Figure EV1/EV1D/Anagen/WT/WT.tif]

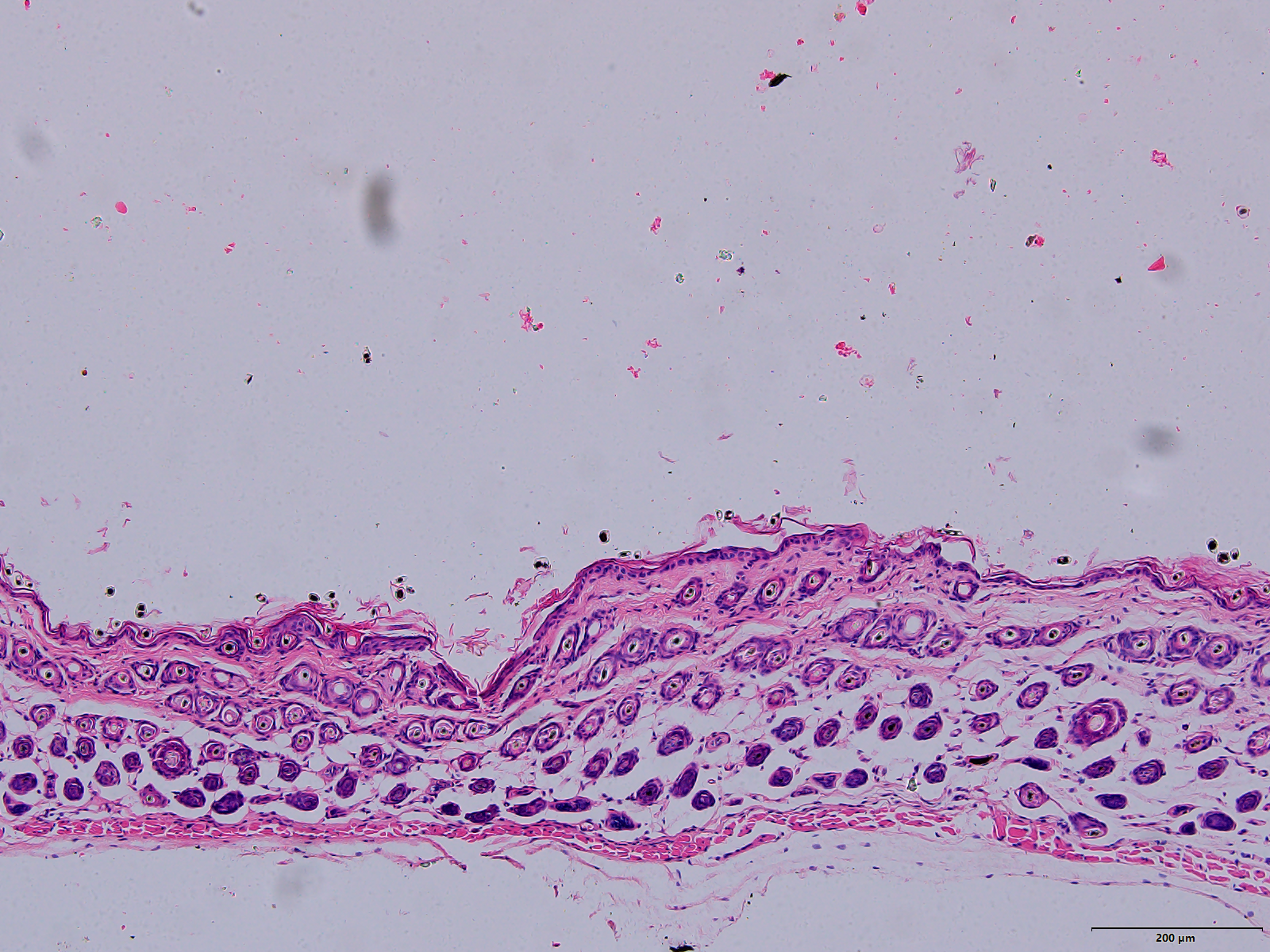

Supplement: Supplementary file 9 — EV Figures Source Data [file 44319_2024_327_MOESM9_ESM.zip › source data-Supplemental Figures/Figure EV1/EV1D/Catagen/KO/1.tif]

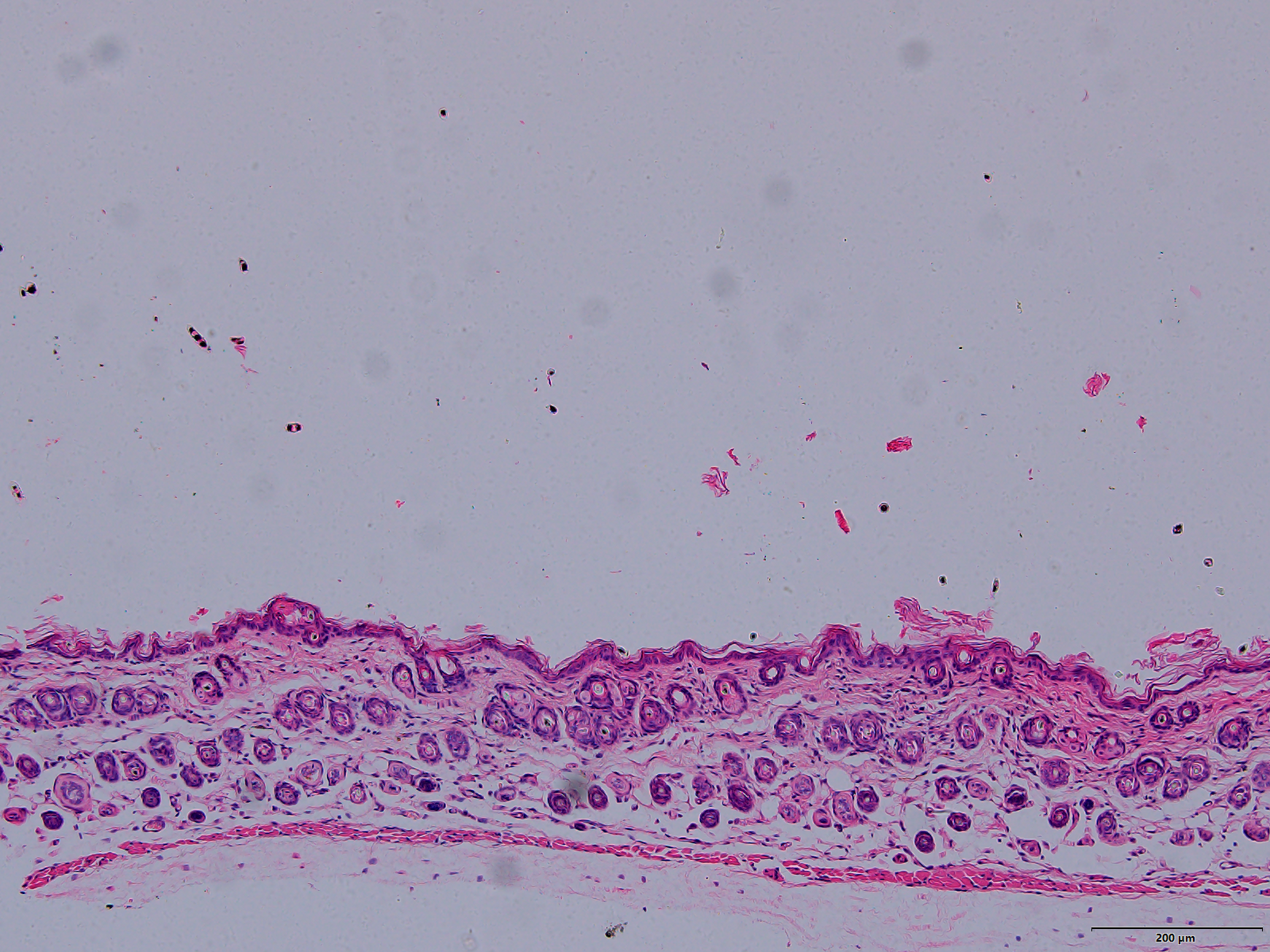

Supplement: Supplementary file 9 — EV Figures Source Data [file 44319_2024_327_MOESM9_ESM.zip › source data-Supplemental Figures/Figure EV1/EV1D/Catagen/WT/1.tif]

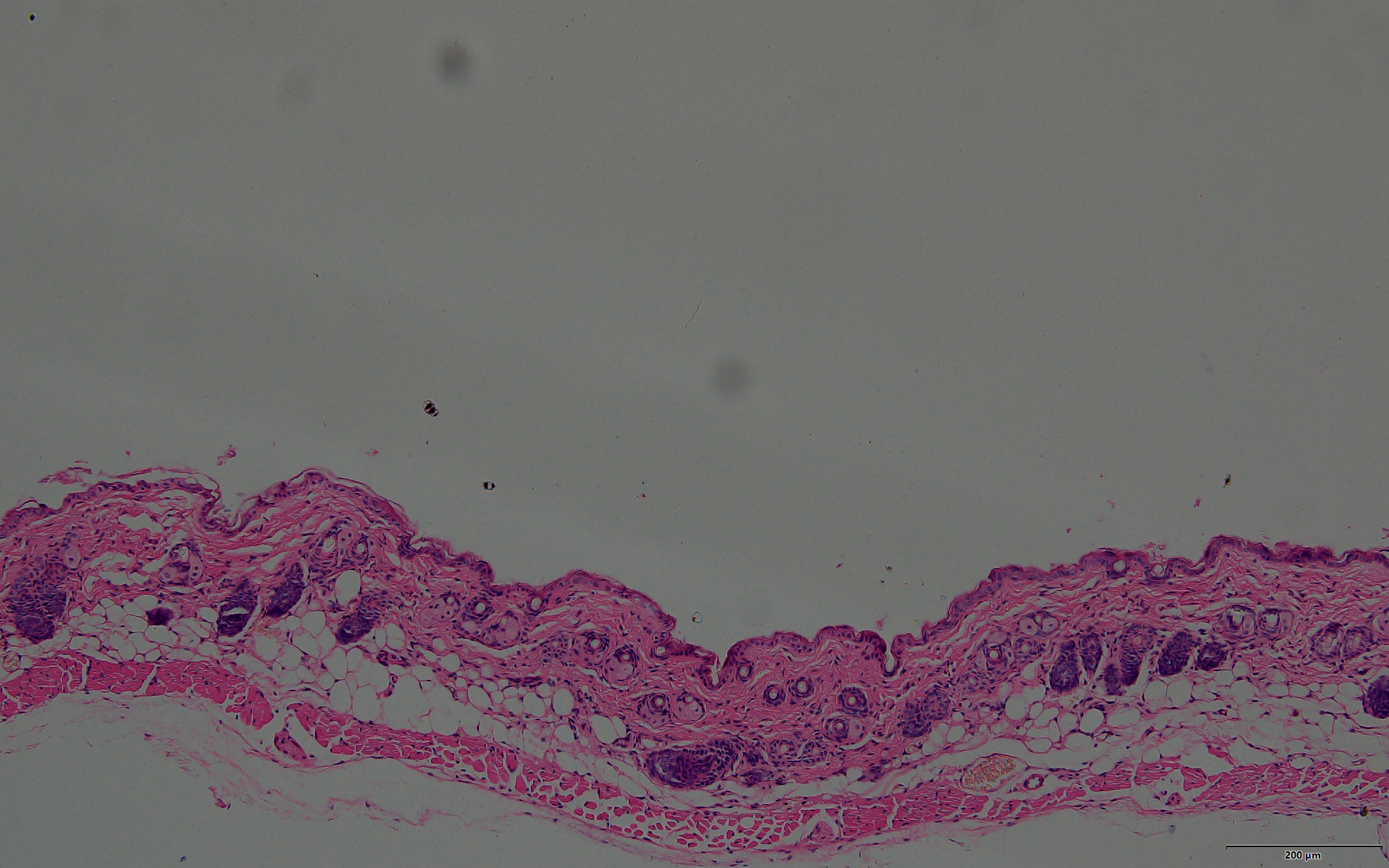

Supplement: Supplementary file 9 — EV Figures Source Data [file 44319_2024_327_MOESM9_ESM.zip › source data-Supplemental Figures/Figure EV1/EV1D/Telogen/KO/KO.tif]

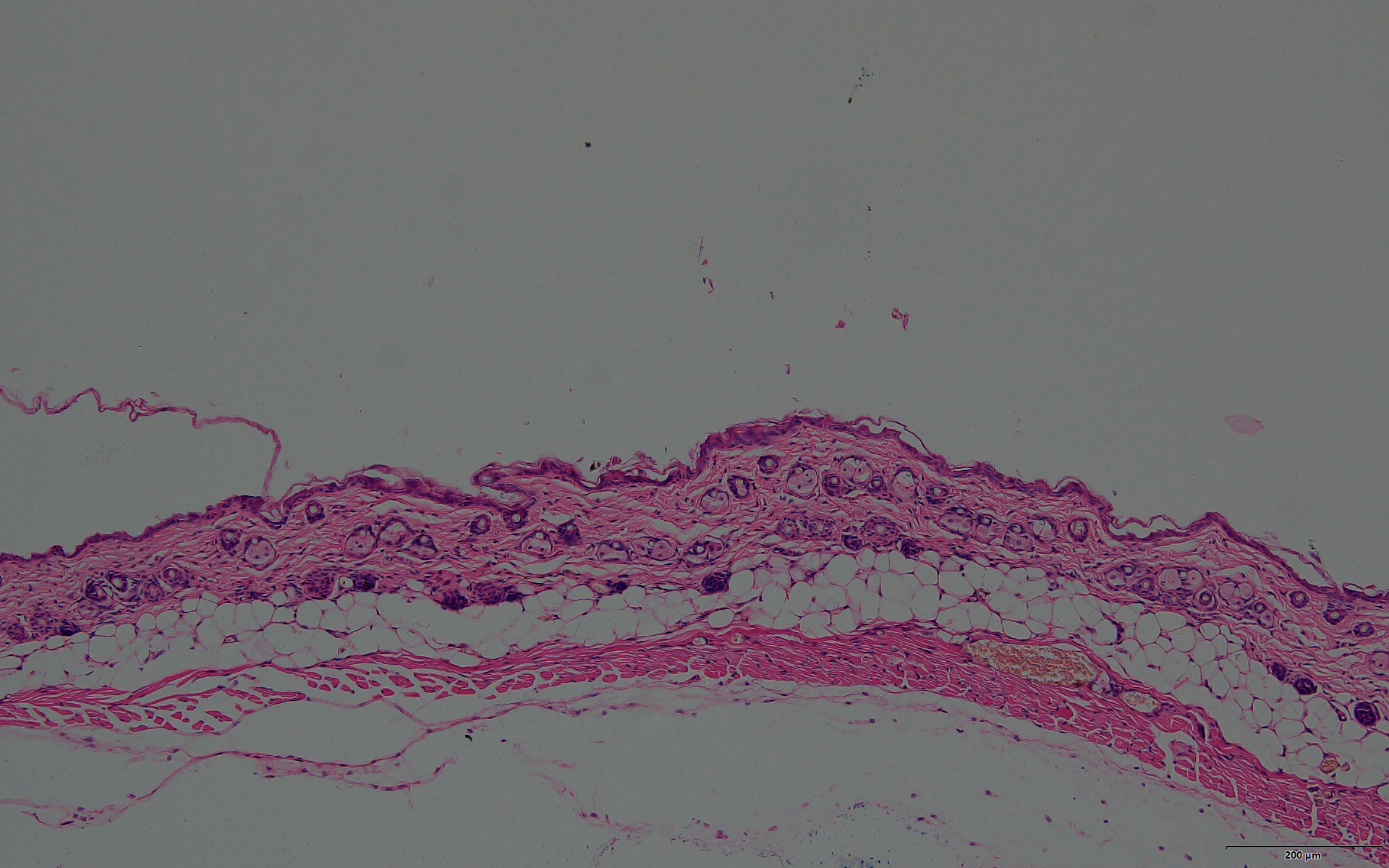

Supplement: Supplementary file 9 — EV Figures Source Data [file 44319_2024_327_MOESM9_ESM.zip › source data-Supplemental Figures/Figure EV1/EV1D/Telogen/WT/WT.tif]

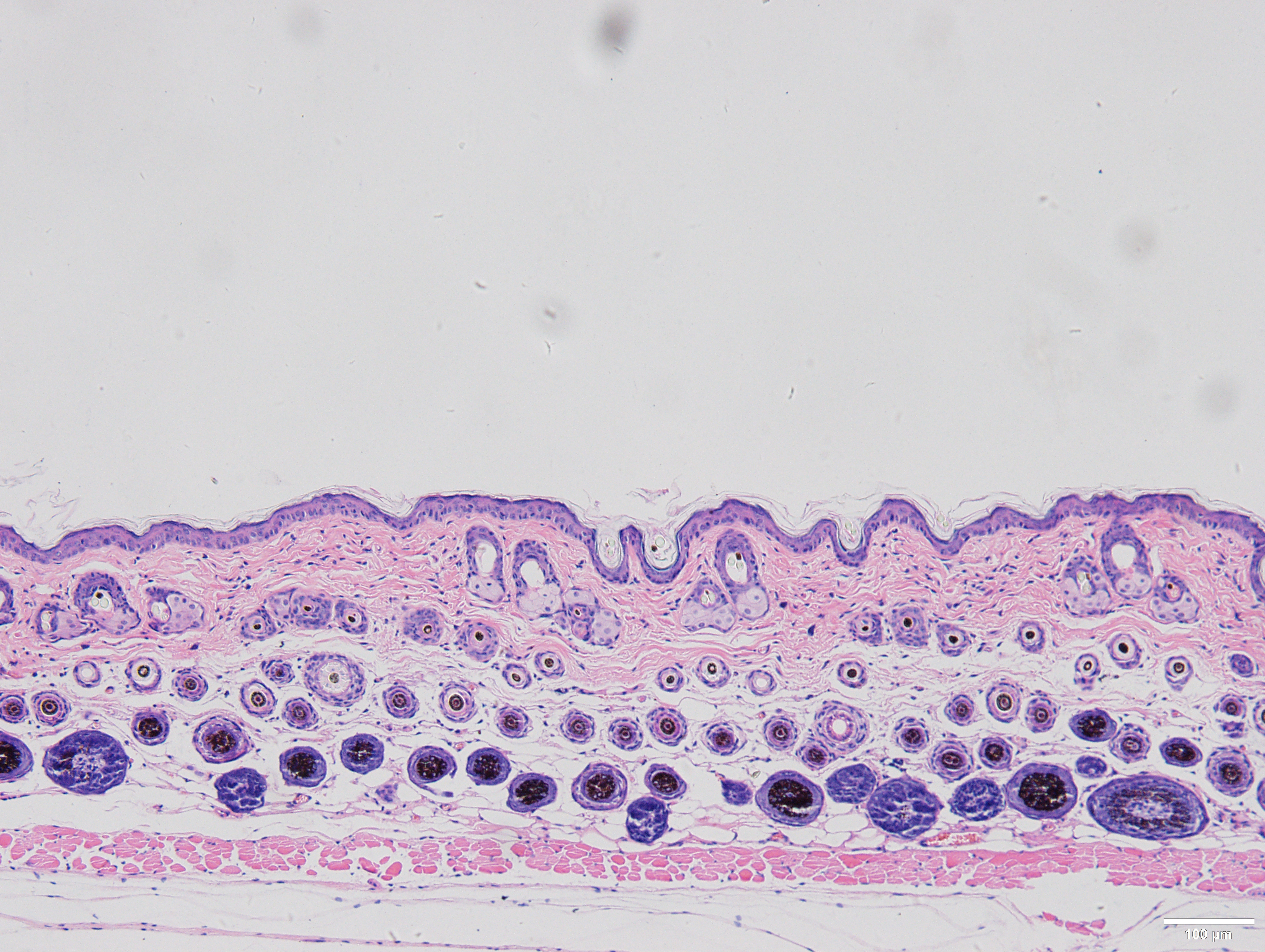

Supplement: Supplementary file 9 — EV Figures Source Data [file 44319_2024_327_MOESM9_ESM.zip › source data-Supplemental Figures/Figure EV2/EV2A/ko/ko (1).jpg]

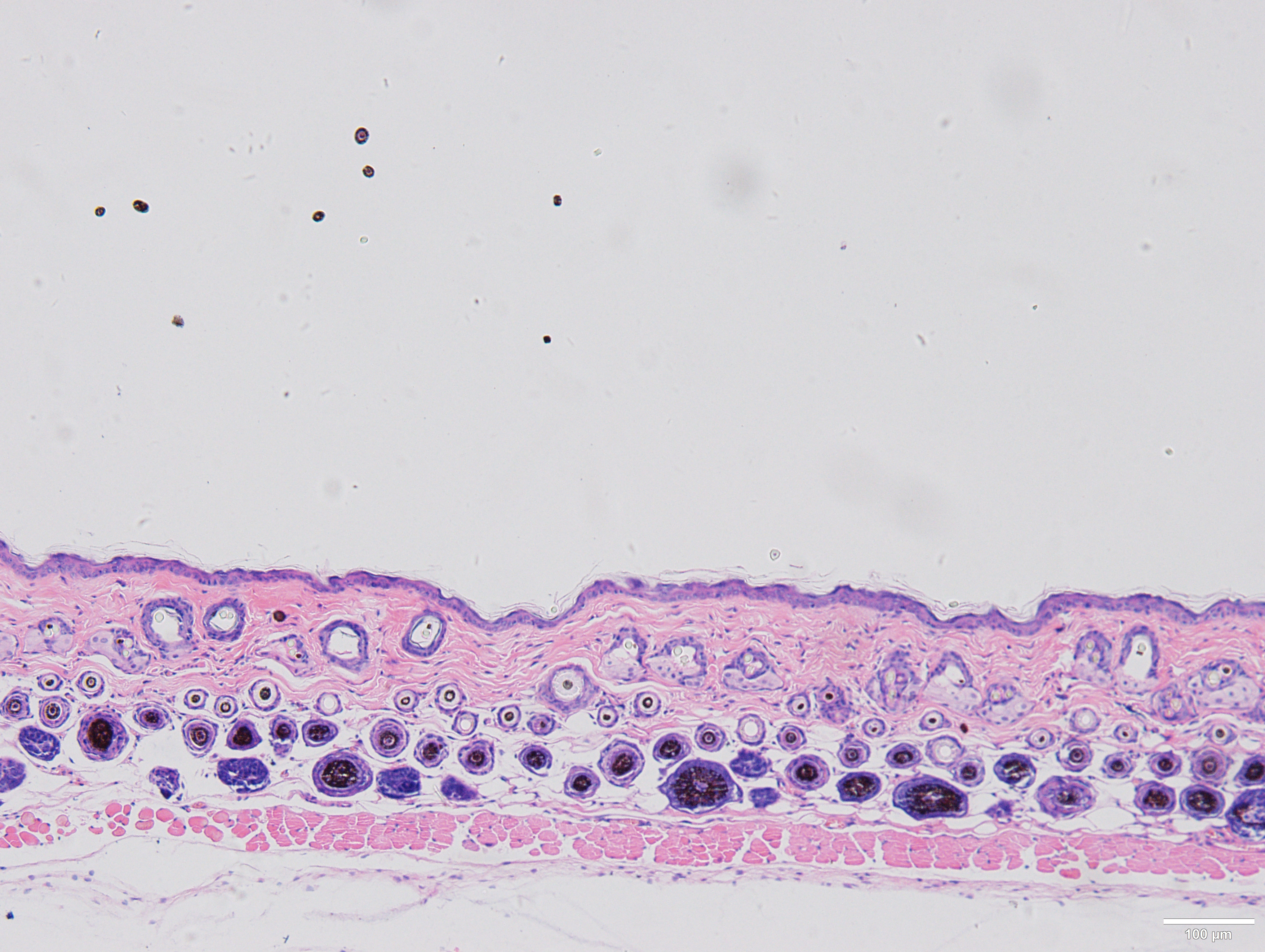

Supplement: Supplementary file 9 — EV Figures Source Data [file 44319_2024_327_MOESM9_ESM.zip › source data-Supplemental Figures/Figure EV2/EV2A/wt/wt (1).jpg]

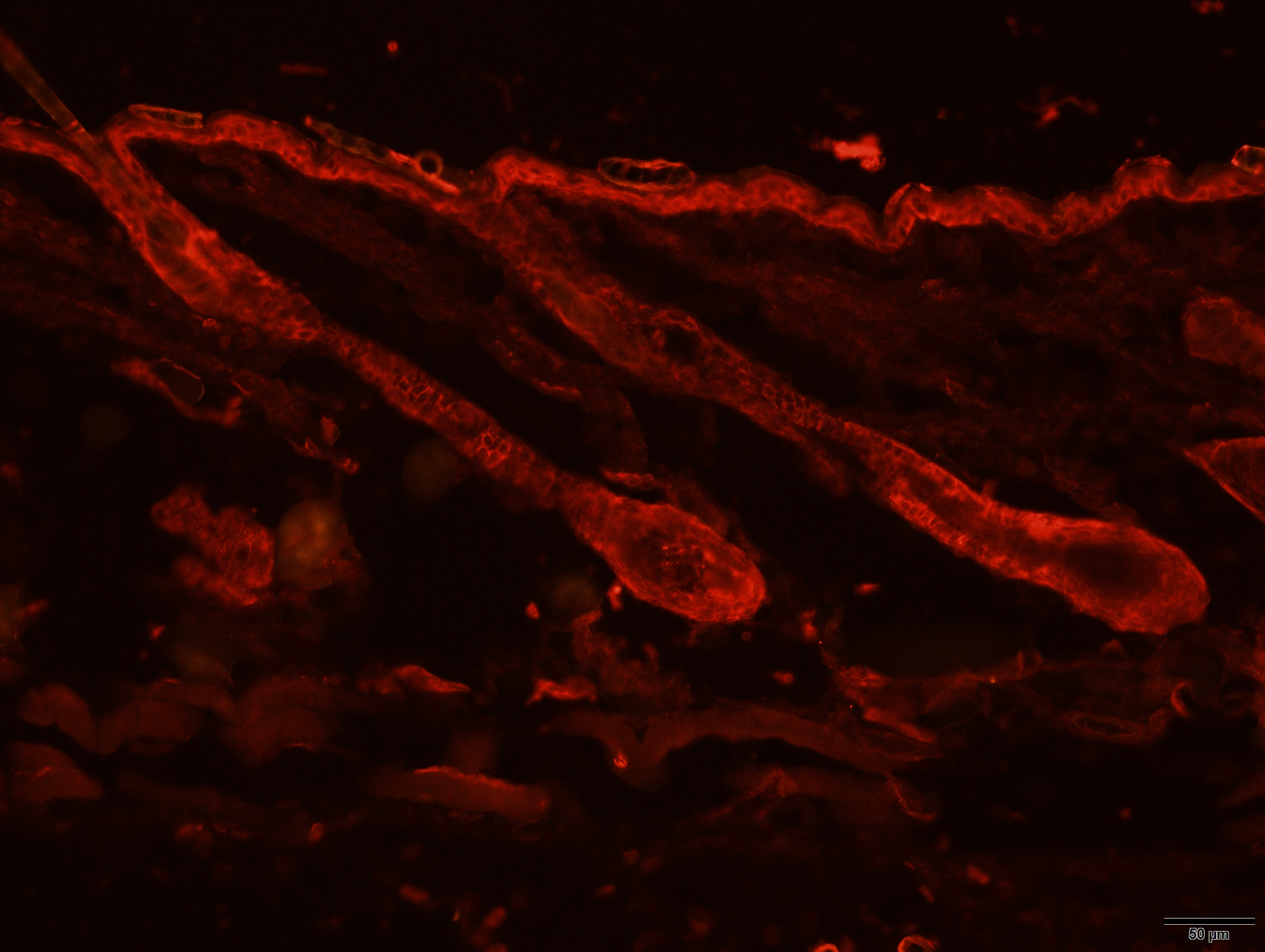

Supplement: Supplementary file 9 — EV Figures Source Data [file 44319_2024_327_MOESM9_ESM.zip › source data-Supplemental Figures/Figure EV2/EV2D/KO/1 (1).jpg]

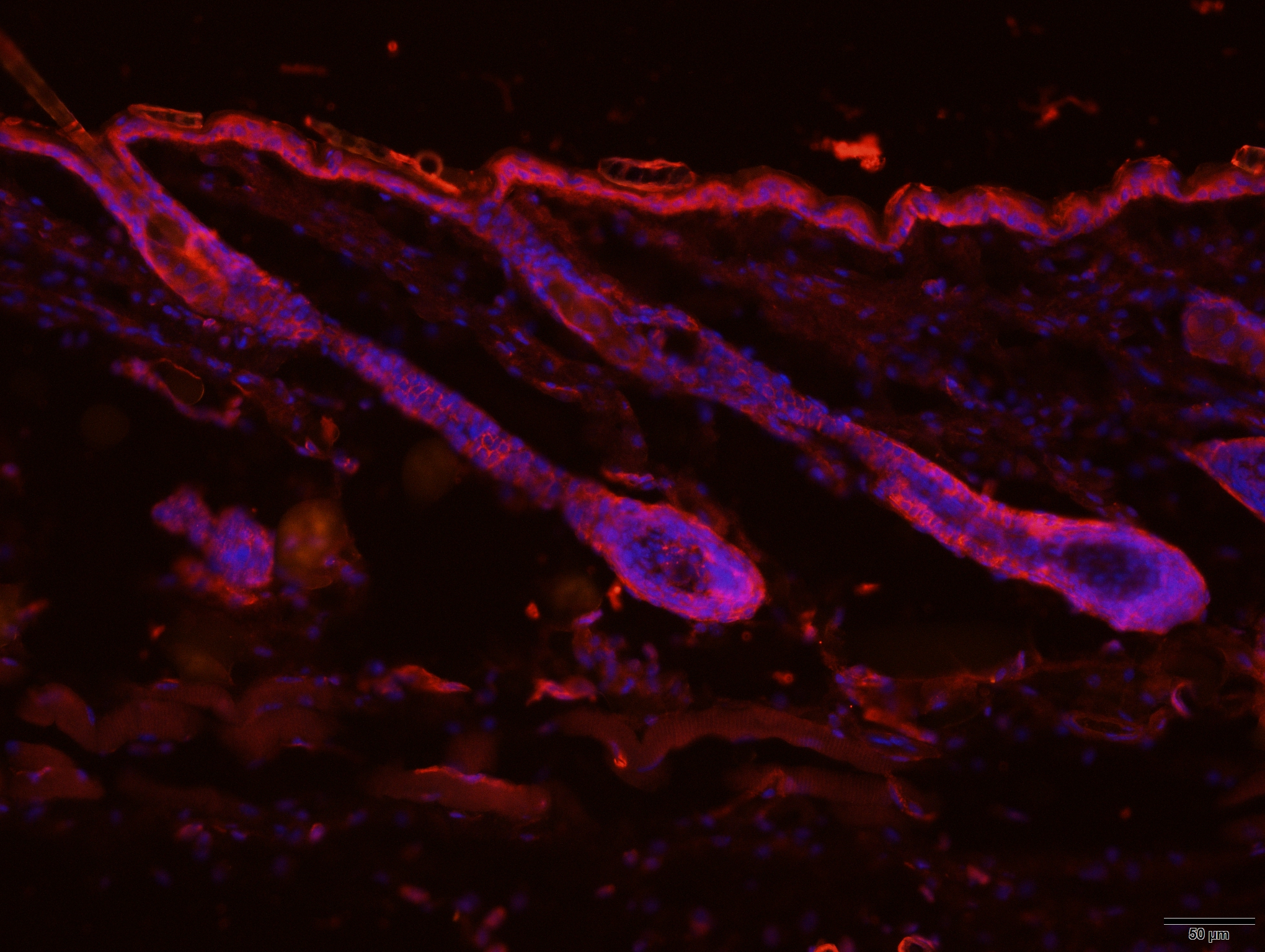

Supplement: Supplementary file 9 — EV Figures Source Data [file 44319_2024_327_MOESM9_ESM.zip › source data-Supplemental Figures/Figure EV2/EV2D/KO/1 (2).jpg]

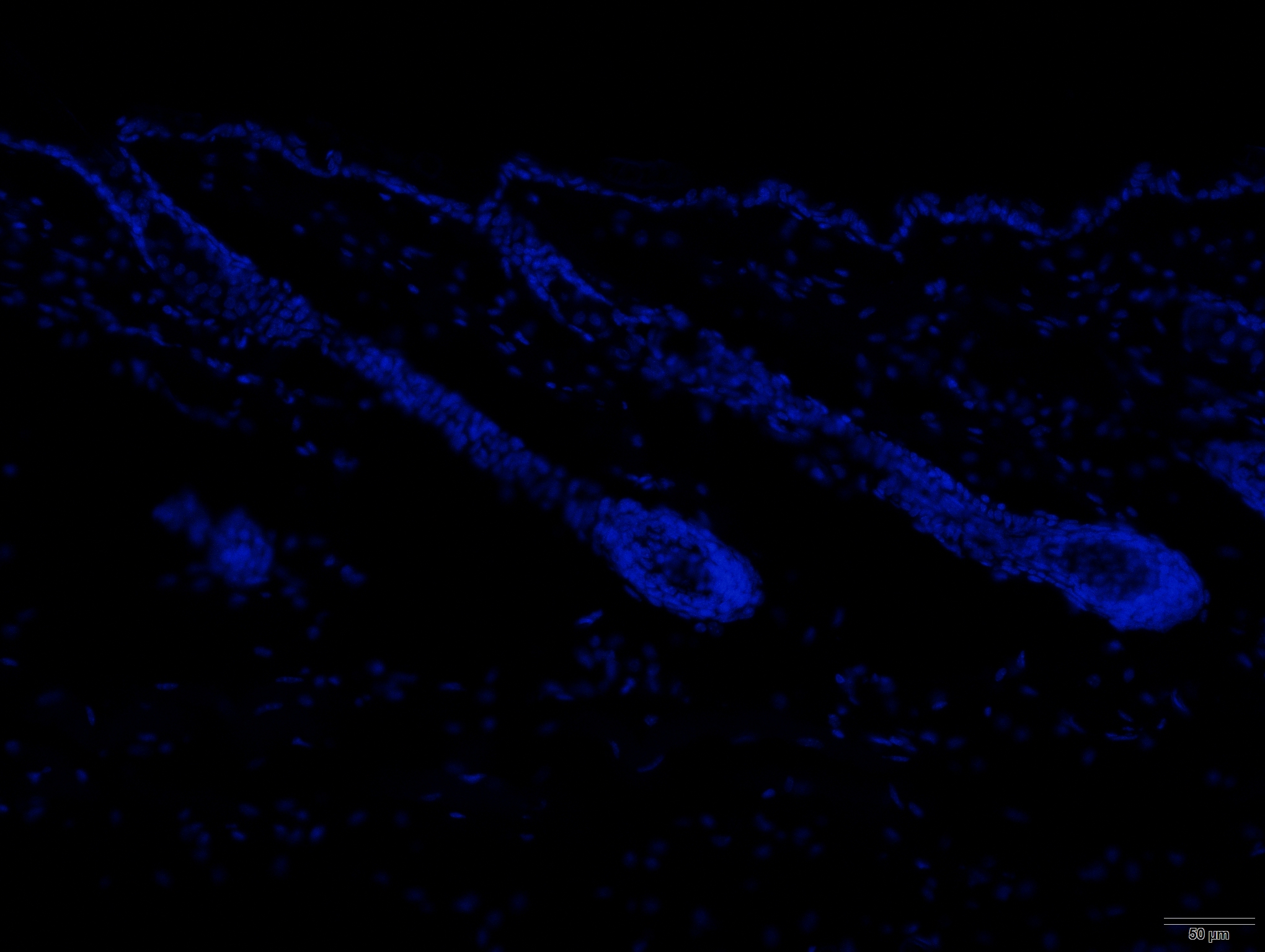

Supplement: Supplementary file 9 — EV Figures Source Data [file 44319_2024_327_MOESM9_ESM.zip › source data-Supplemental Figures/Figure EV2/EV2D/KO/1 (3).jpg]

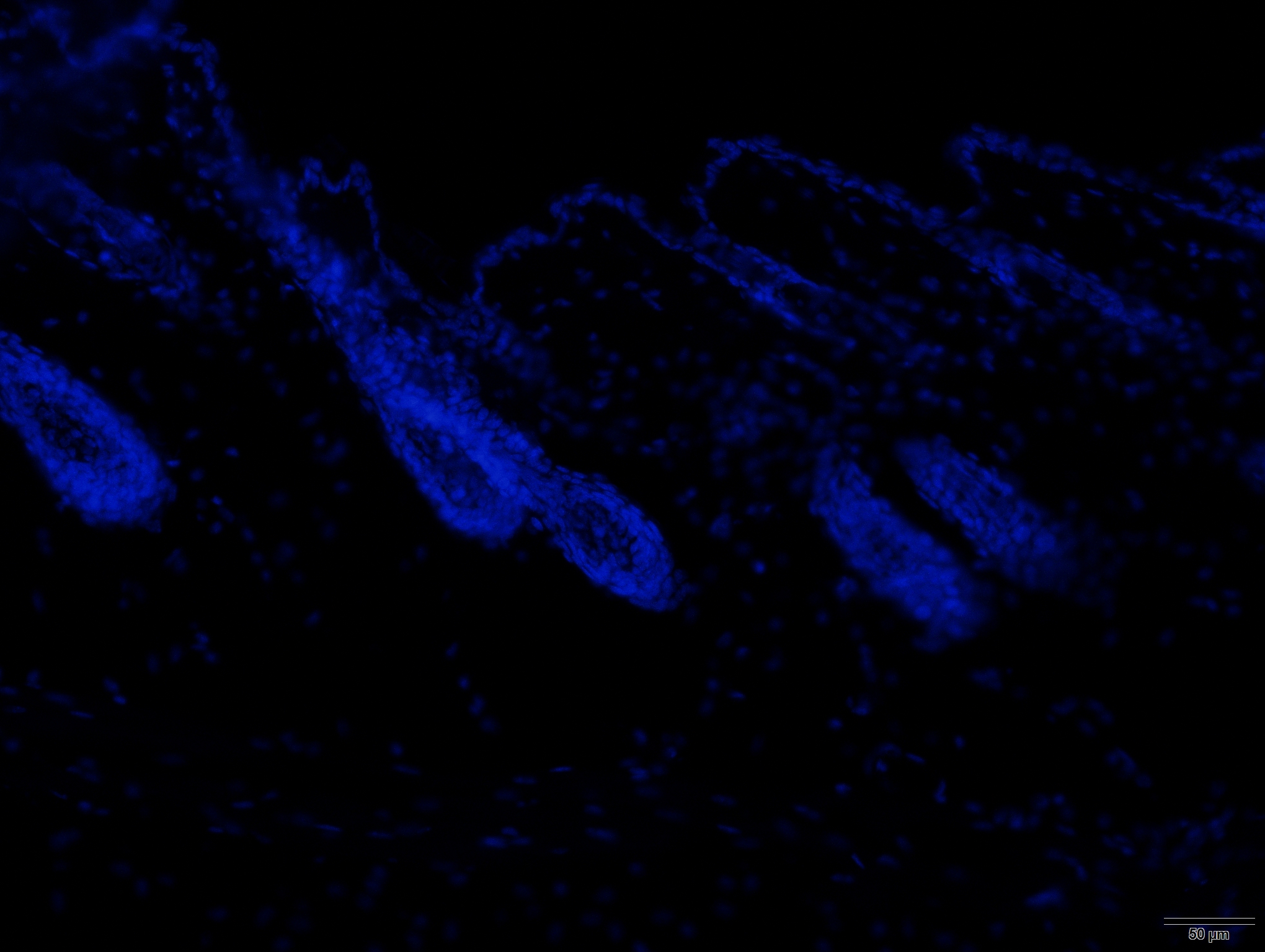

Supplement: Supplementary file 9 — EV Figures Source Data [file 44319_2024_327_MOESM9_ESM.zip › source data-Supplemental Figures/Figure EV2/EV2D/WT/1 (1).jpg]

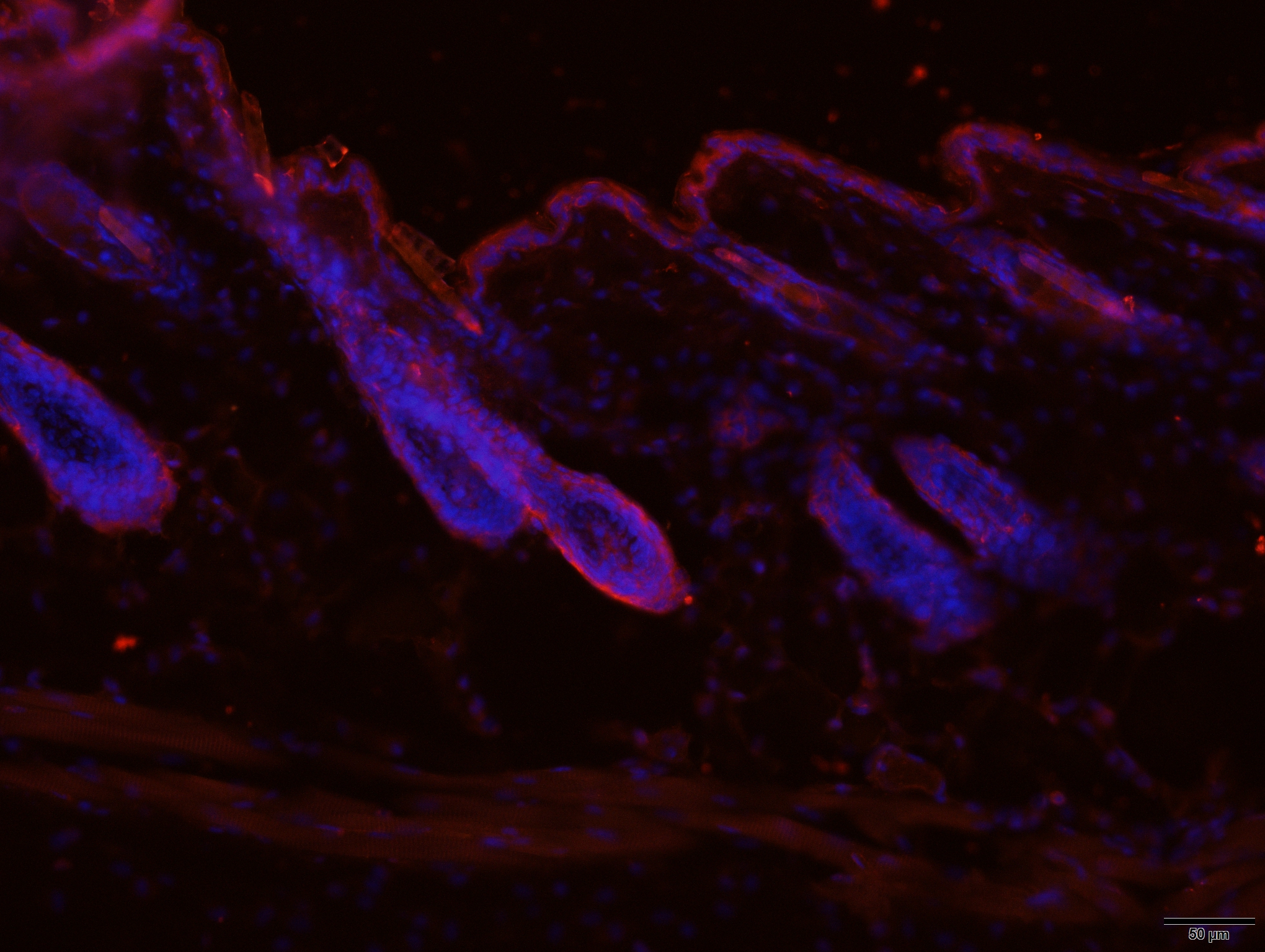

Supplement: Supplementary file 9 — EV Figures Source Data [file 44319_2024_327_MOESM9_ESM.zip › source data-Supplemental Figures/Figure EV2/EV2D/WT/1 (2).jpg]

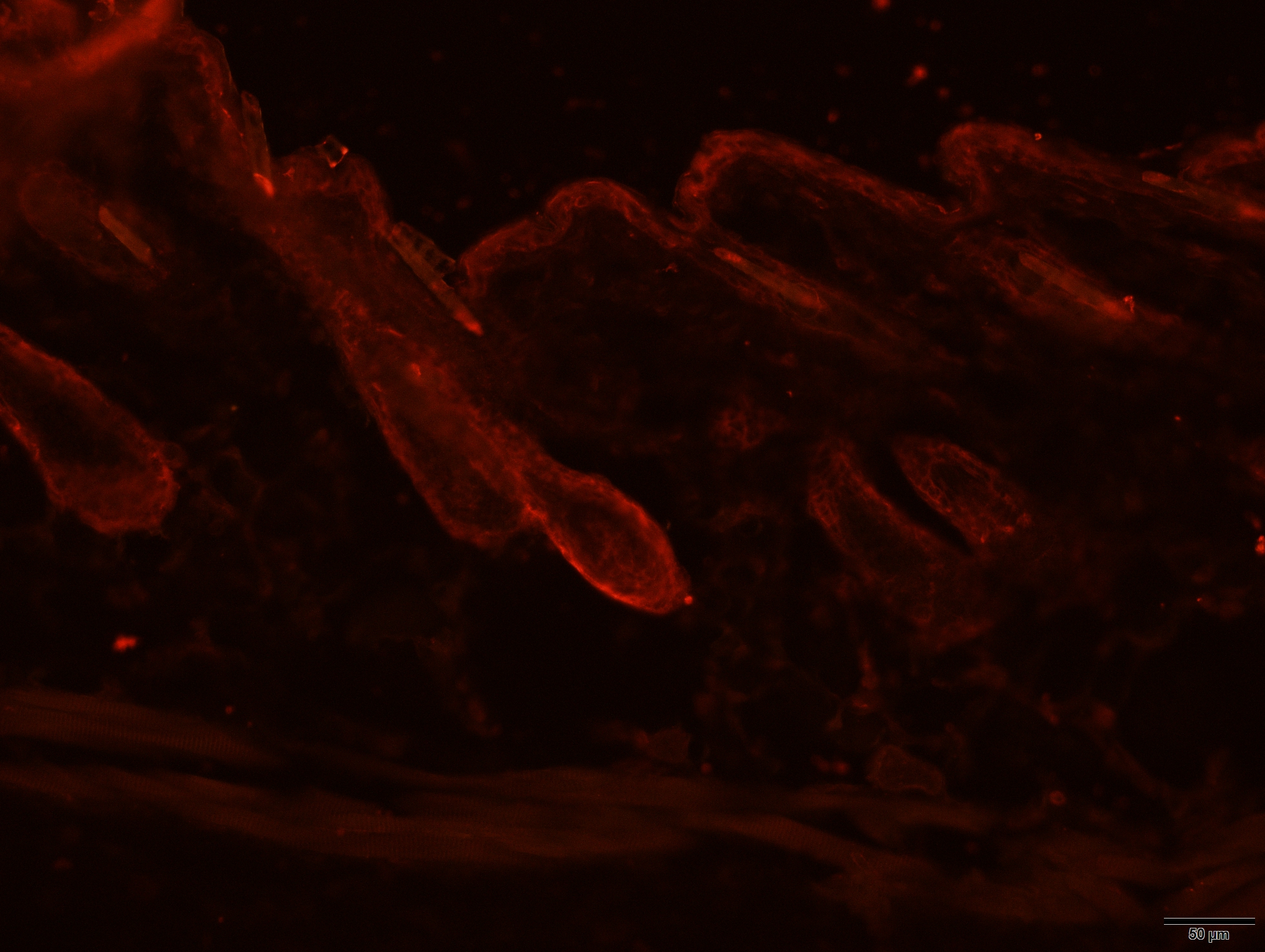

Supplement: Supplementary file 9 — EV Figures Source Data [file 44319_2024_327_MOESM9_ESM.zip › source data-Supplemental Figures/Figure EV2/EV2D/WT/1 (3).jpg]

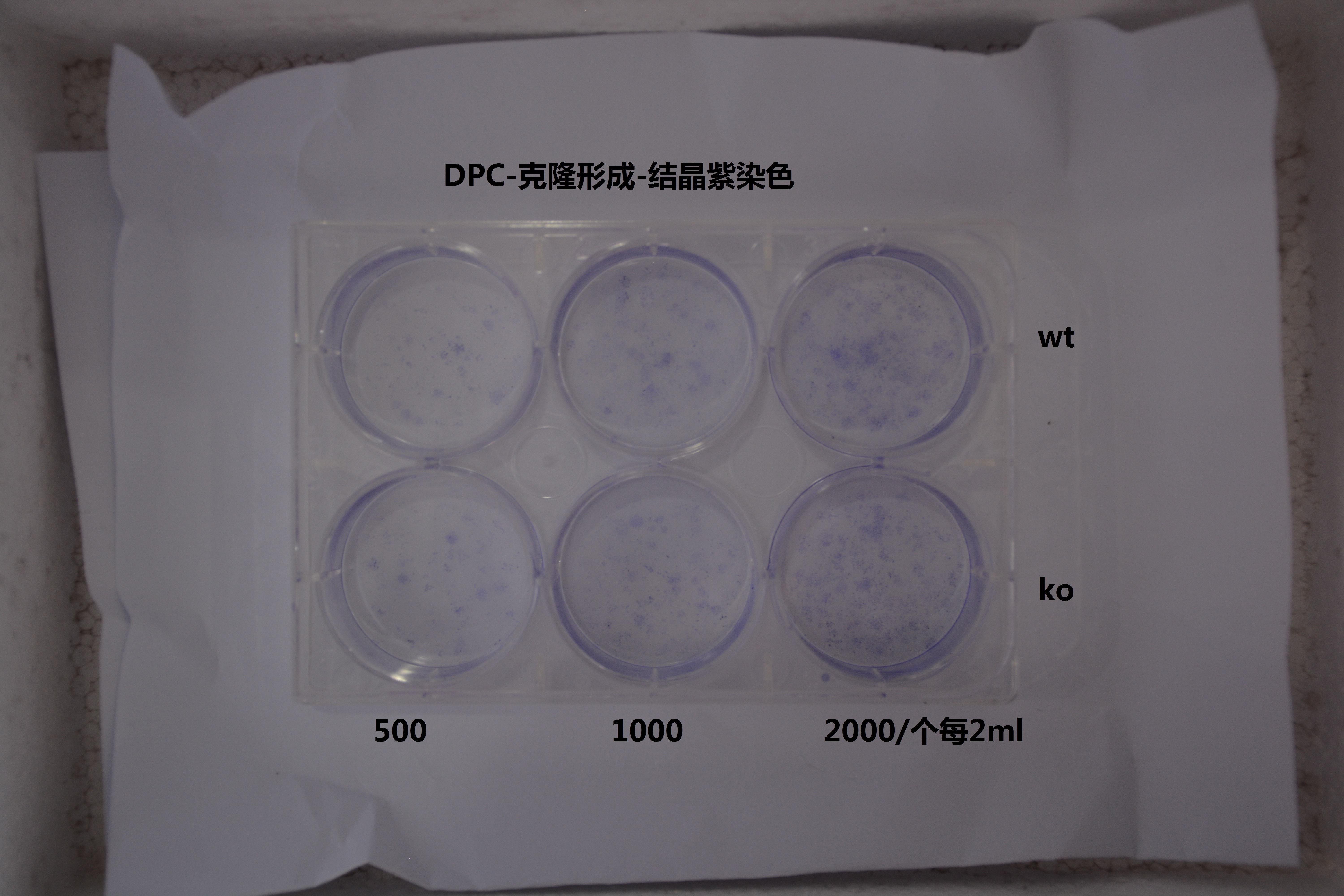

Supplement: Supplementary file 9 — EV Figures Source Data [file 44319_2024_327_MOESM9_ESM.zip › source data-Supplemental Figures/Figure EV3/EV3B/1.JPG]

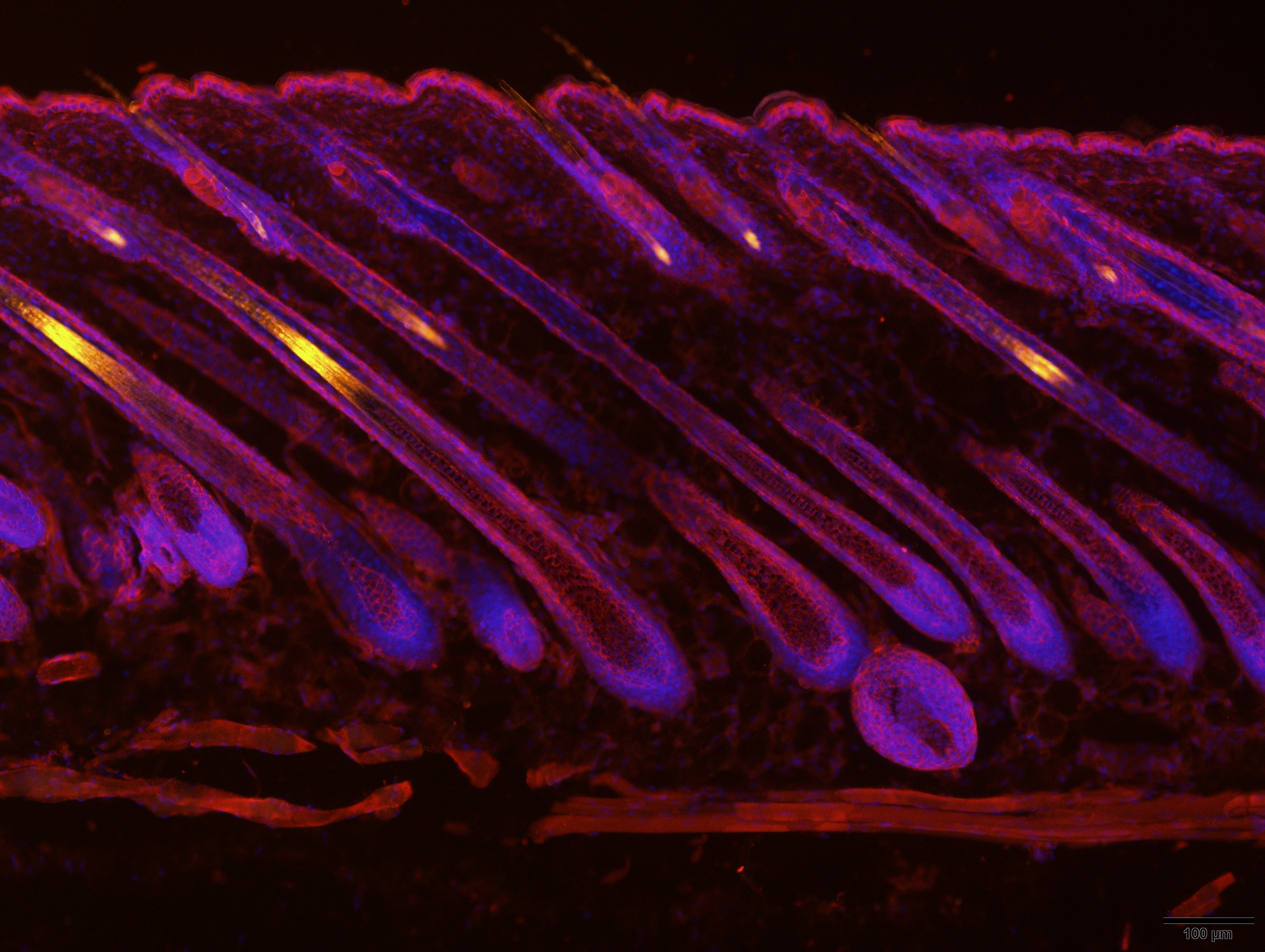

Supplement: Supplementary file 9 — EV Figures Source Data [file 44319_2024_327_MOESM9_ESM.zip › source data-Supplemental Figures/Figure EV4/EV4C/KO/1 (1).jpg]

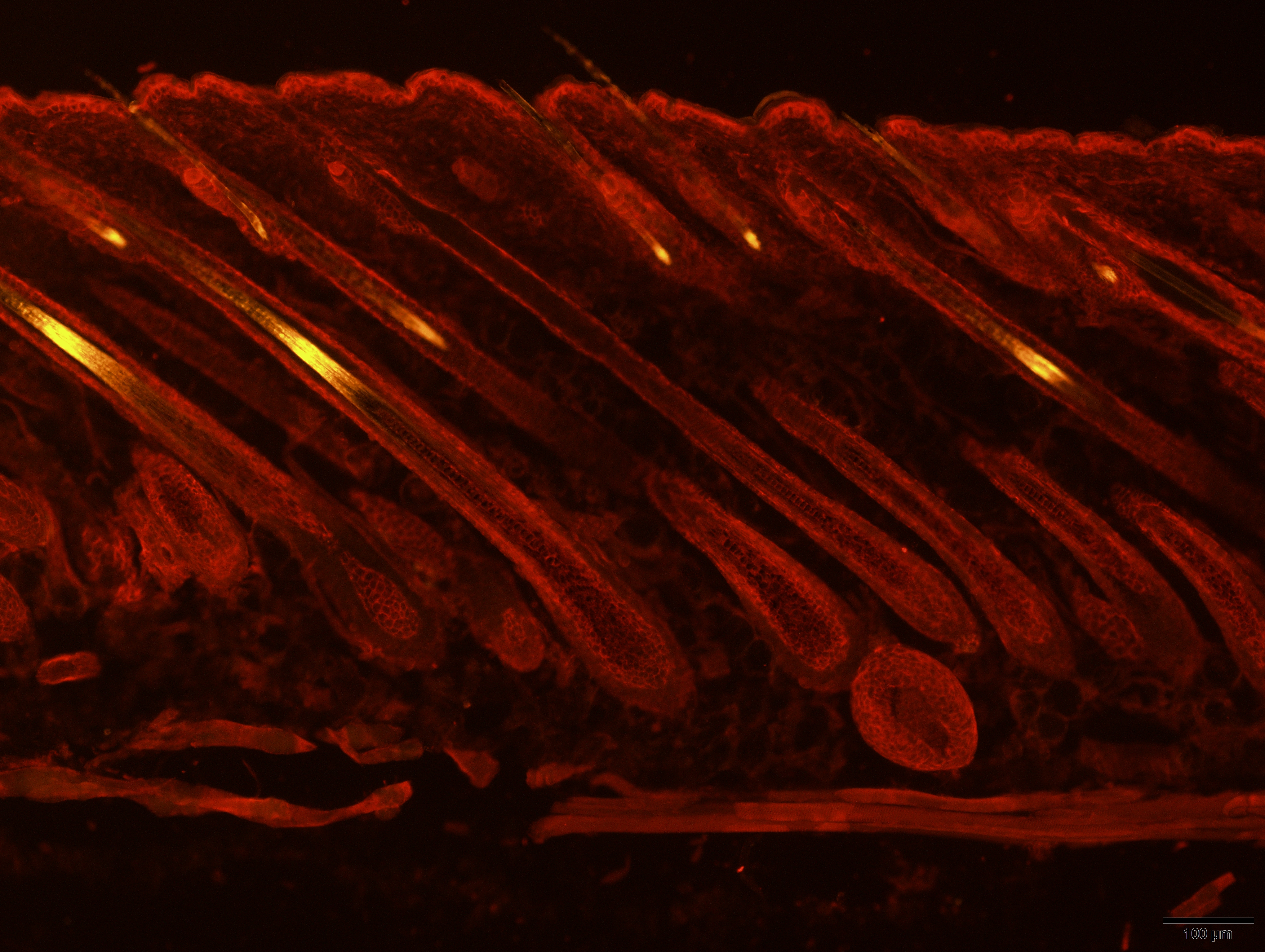

Supplement: Supplementary file 9 — EV Figures Source Data [file 44319_2024_327_MOESM9_ESM.zip › source data-Supplemental Figures/Figure EV4/EV4C/KO/1 (2).jpg]

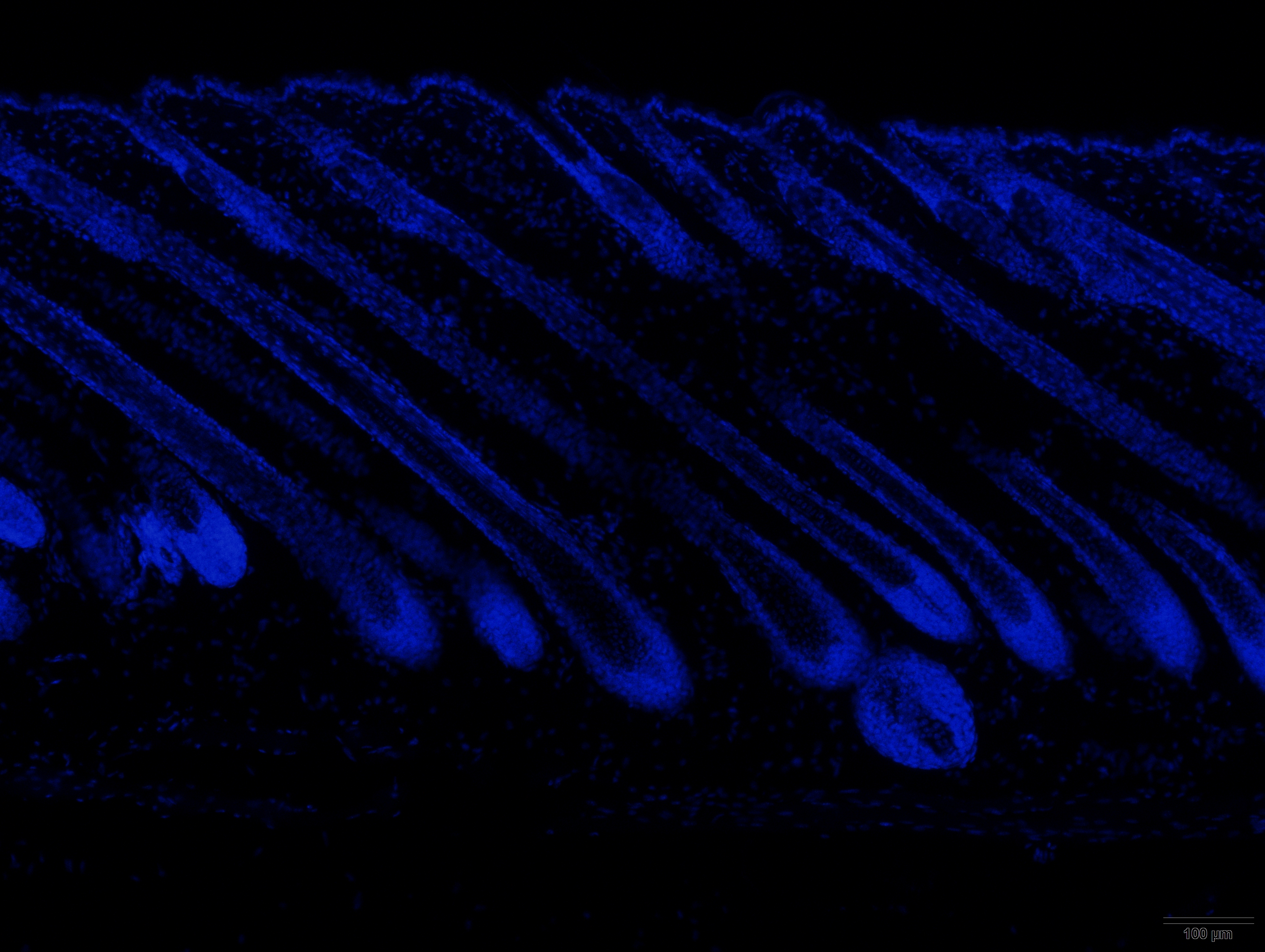

Supplement: Supplementary file 9 — EV Figures Source Data [file 44319_2024_327_MOESM9_ESM.zip › source data-Supplemental Figures/Figure EV4/EV4C/KO/1 (3).jpg]

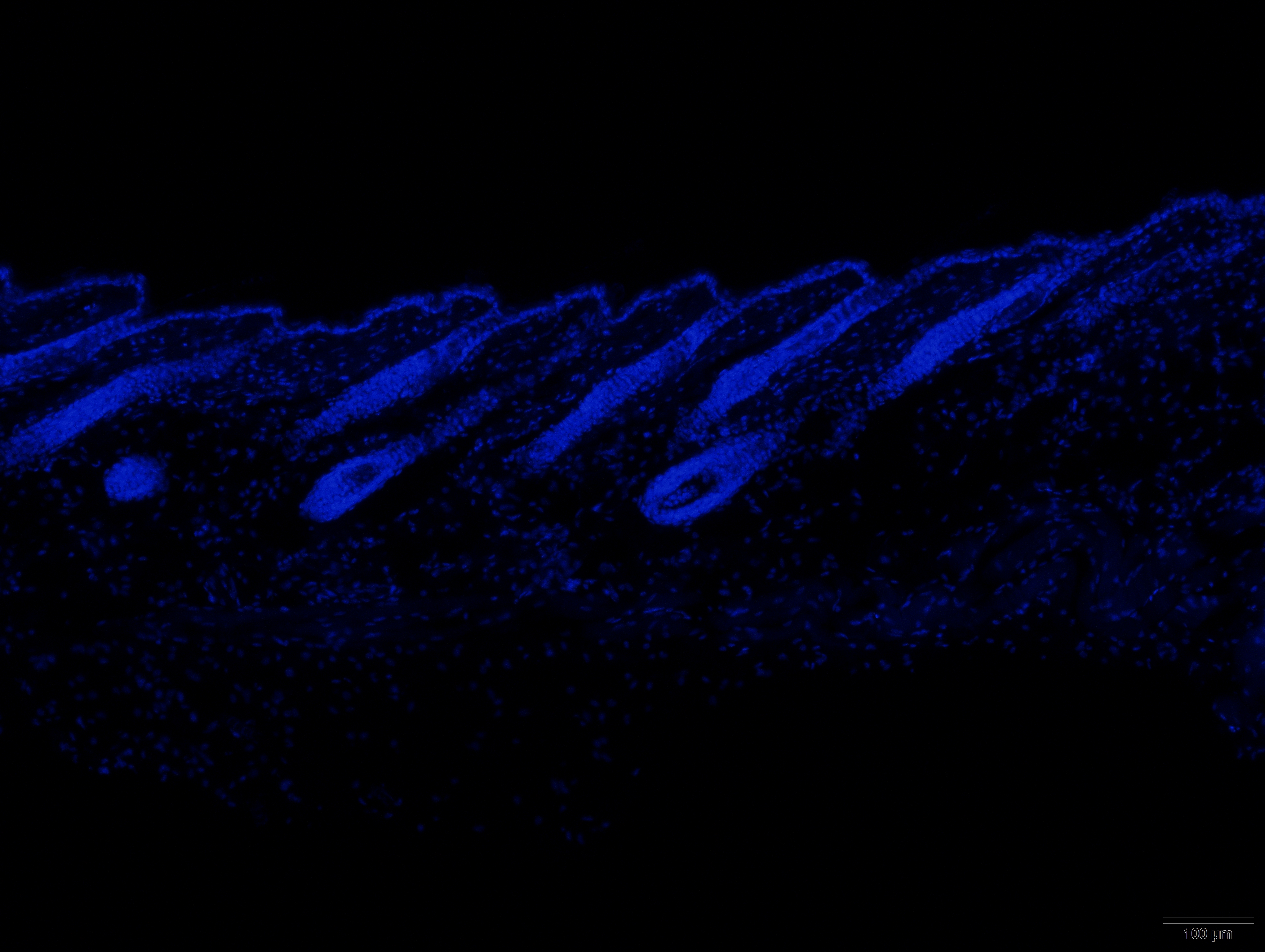

Supplement: Supplementary file 9 — EV Figures Source Data [file 44319_2024_327_MOESM9_ESM.zip › source data-Supplemental Figures/Figure EV4/EV4C/WT/1.jpg]

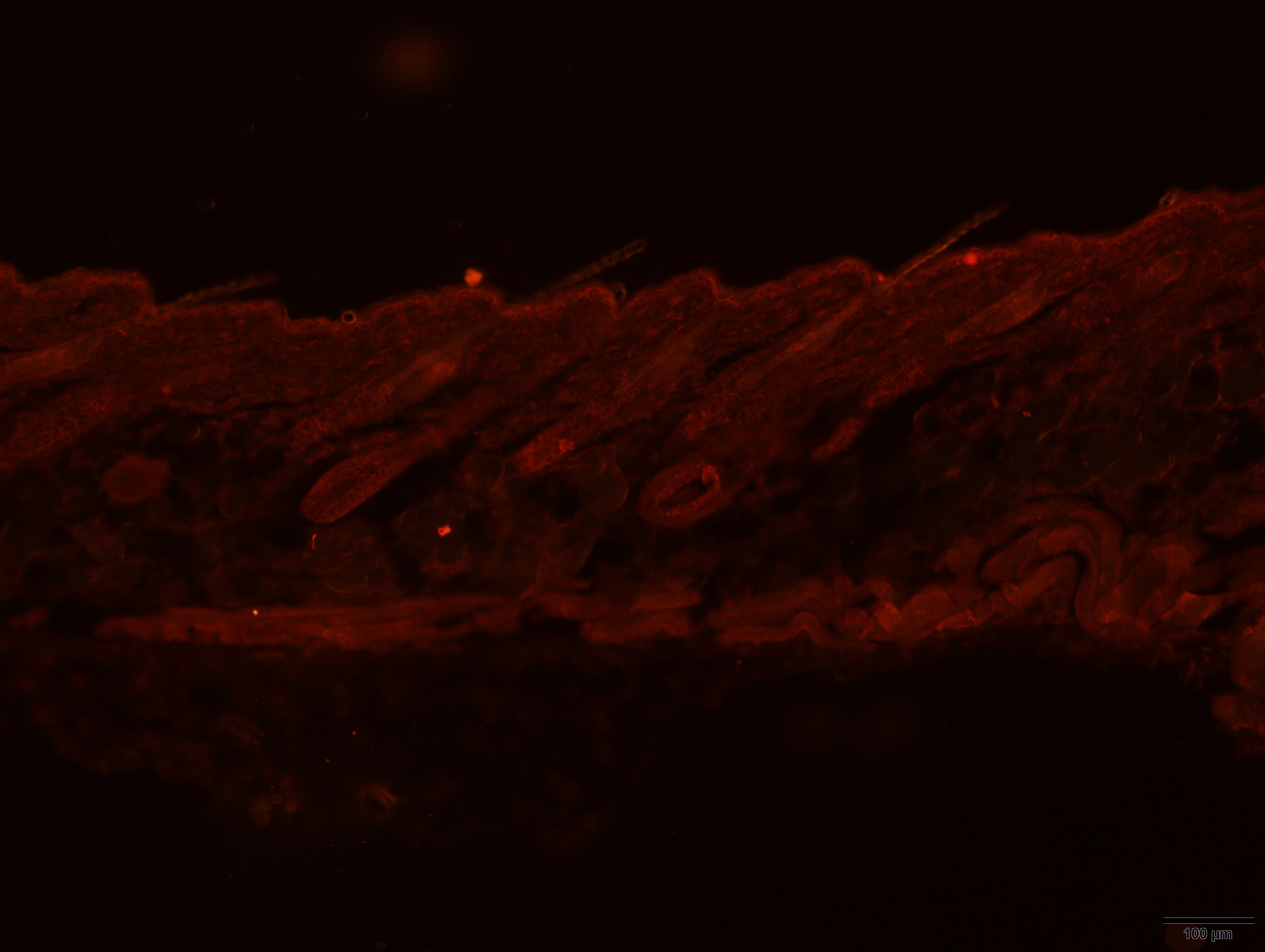

Supplement: Supplementary file 9 — EV Figures Source Data [file 44319_2024_327_MOESM9_ESM.zip › source data-Supplemental Figures/Figure EV4/EV4C/WT/2.jpg]

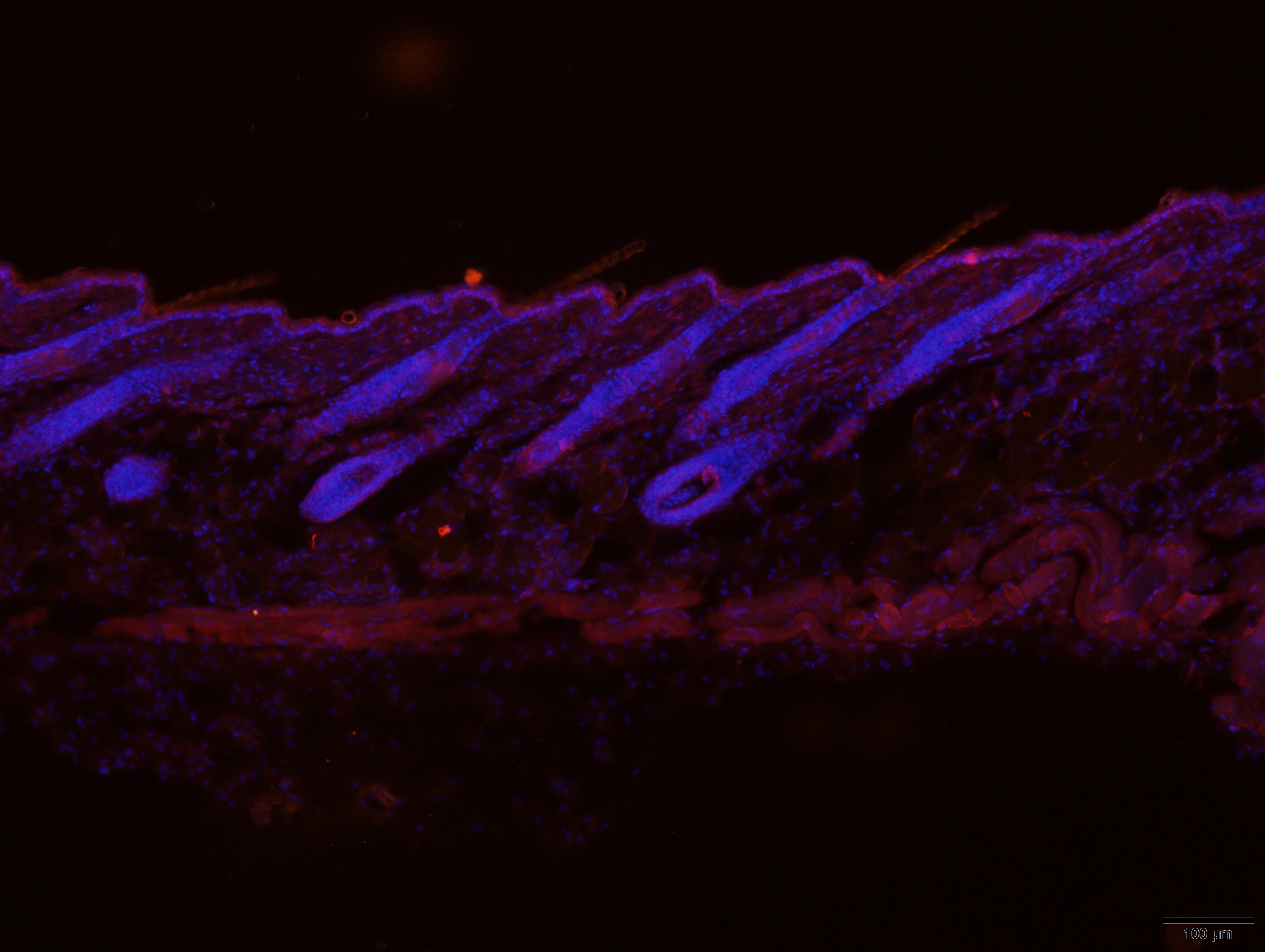

Supplement: Supplementary file 9 — EV Figures Source Data [file 44319_2024_327_MOESM9_ESM.zip › source data-Supplemental Figures/Figure EV4/EV4C/WT/3.jpg]

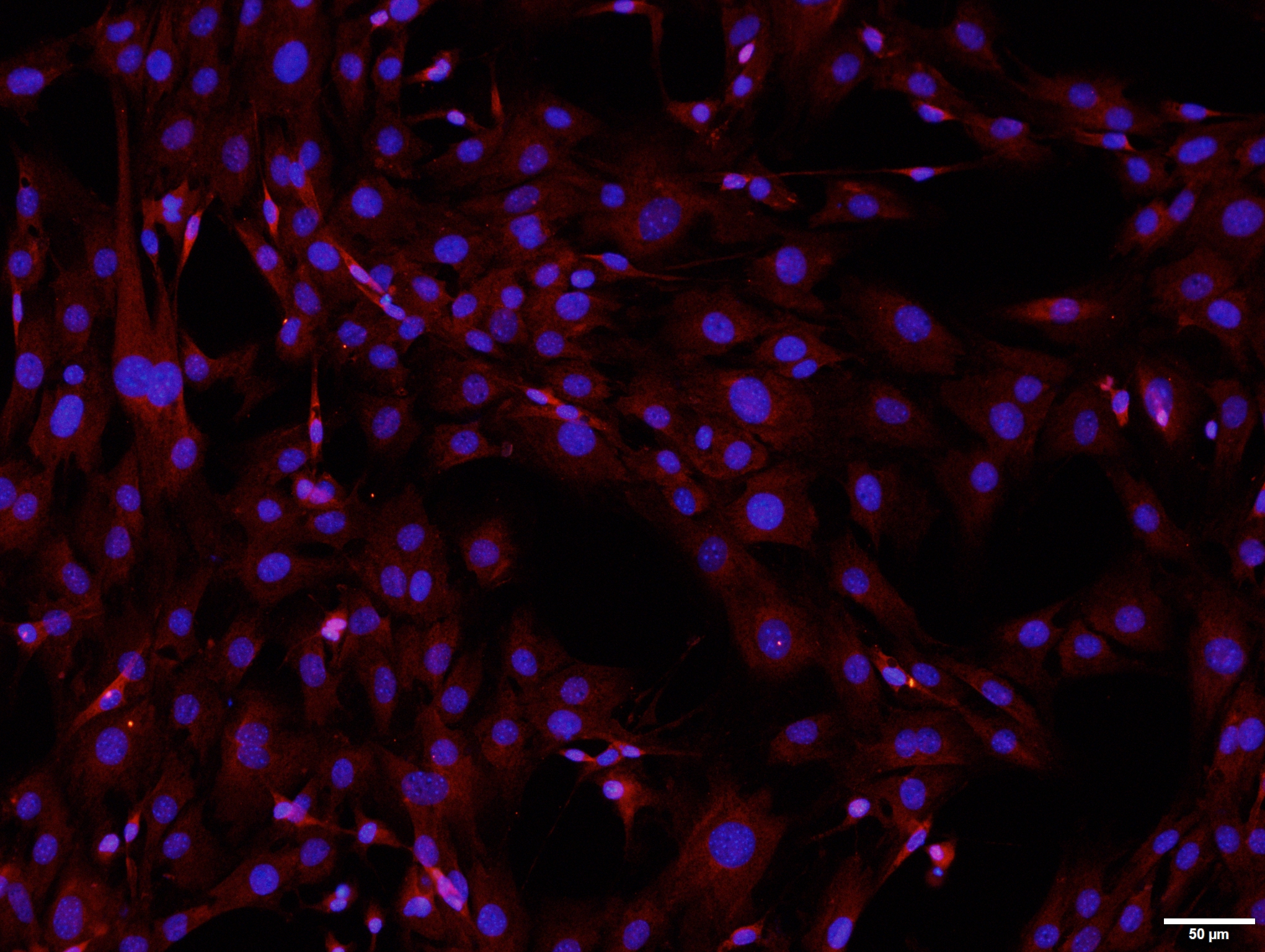

Supplement: Supplementary file 9 — EV Figures Source Data [file 44319_2024_327_MOESM9_ESM.zip › source data-Supplemental Figures/Figure EV5/EV5A/KO/1 (1).jpg]

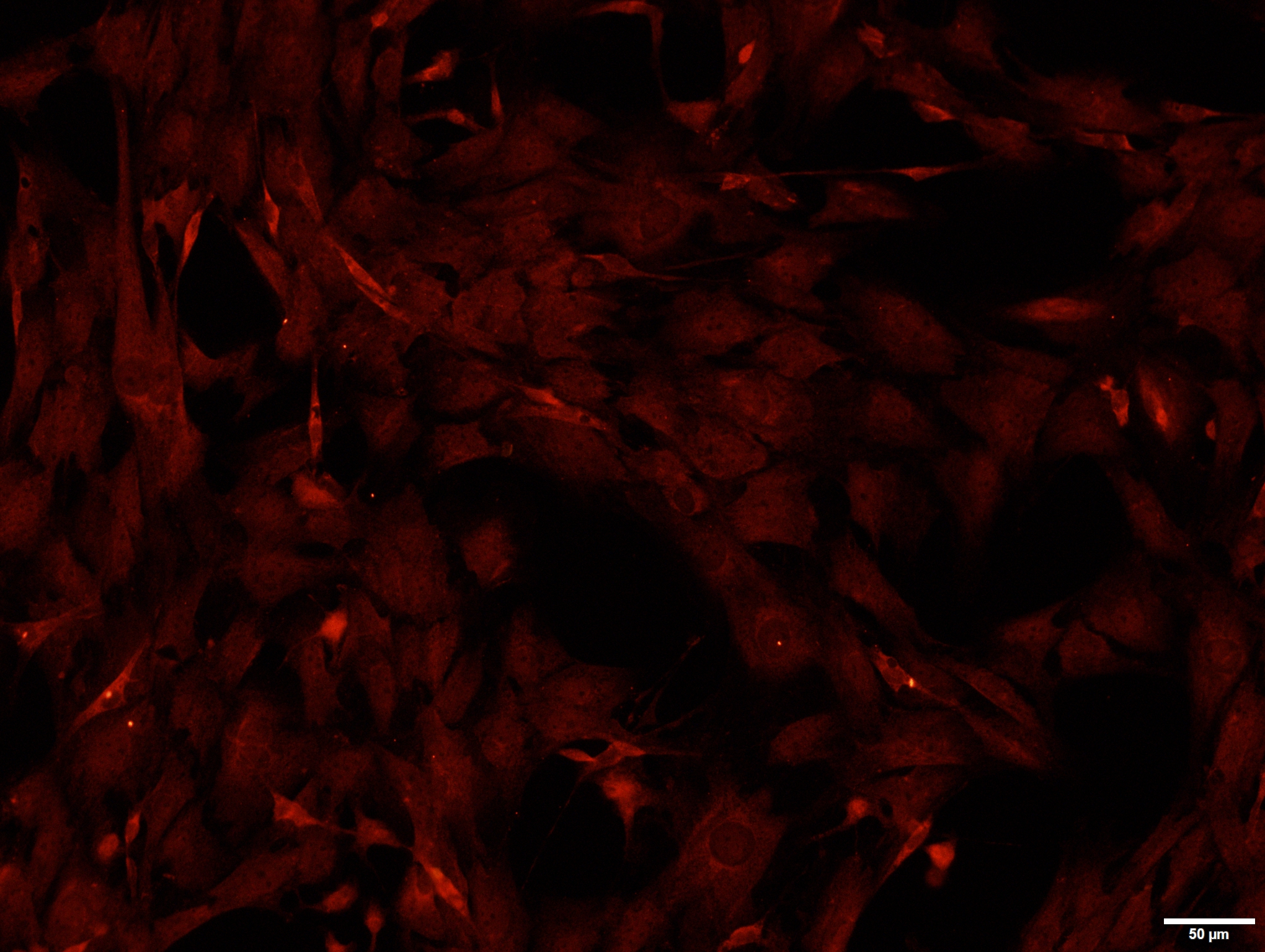

Supplement: Supplementary file 9 — EV Figures Source Data [file 44319_2024_327_MOESM9_ESM.zip › source data-Supplemental Figures/Figure EV5/EV5A/KO/1 (2).jpg]

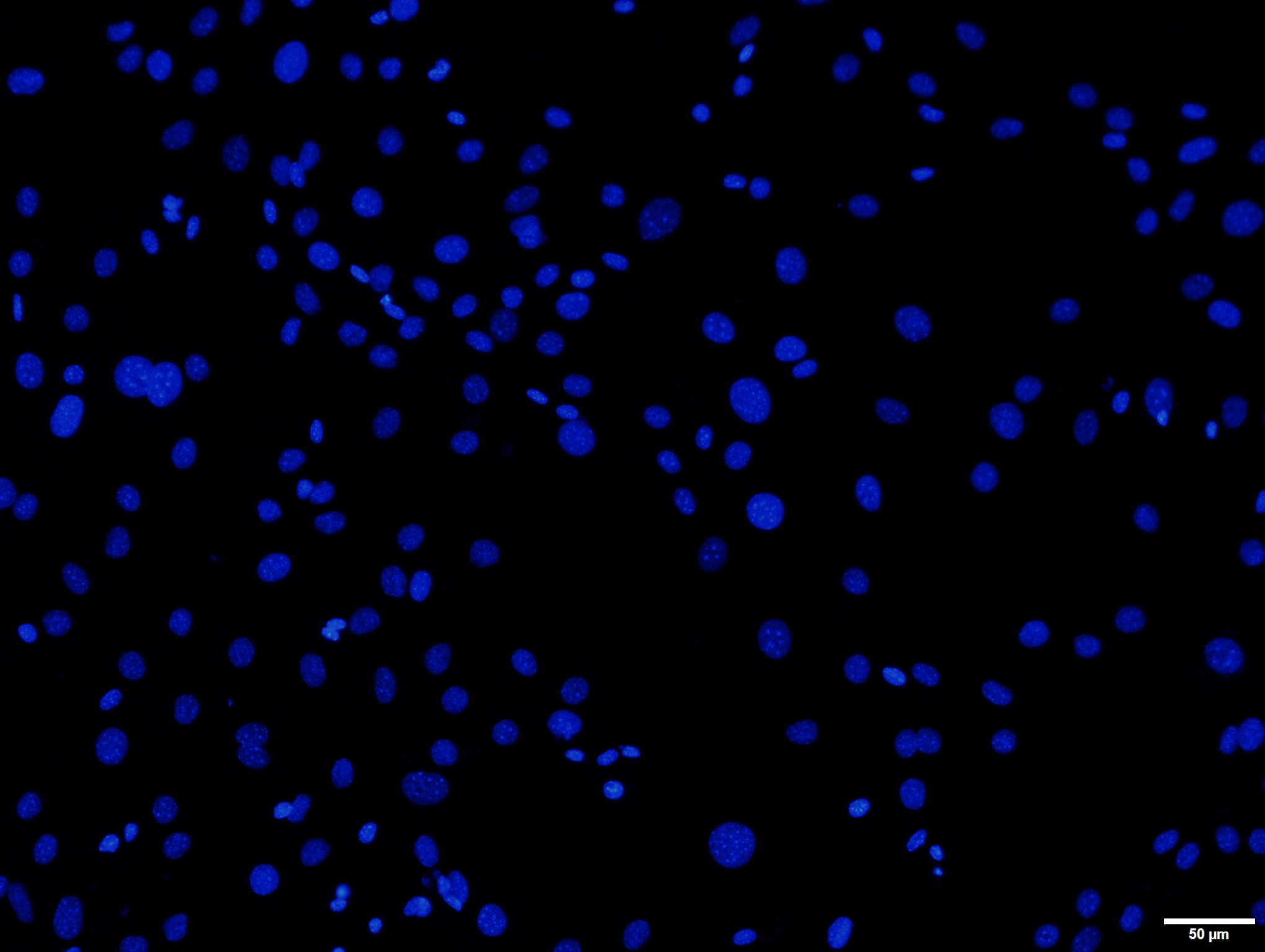

Supplement: Supplementary file 9 — EV Figures Source Data [file 44319_2024_327_MOESM9_ESM.zip › source data-Supplemental Figures/Figure EV5/EV5A/KO/1 (3).jpg]

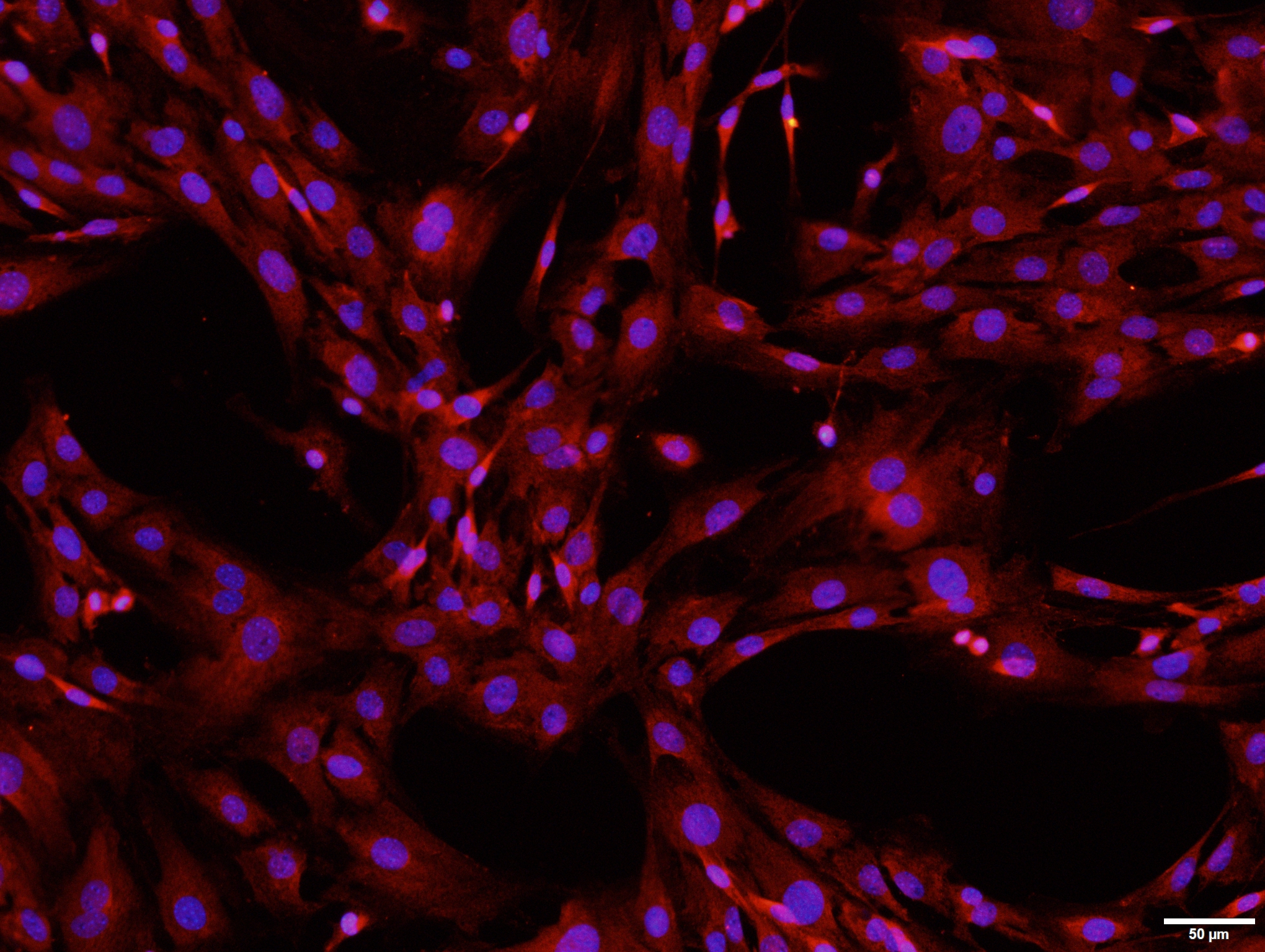

Supplement: Supplementary file 9 — EV Figures Source Data [file 44319_2024_327_MOESM9_ESM.zip › source data-Supplemental Figures/Figure EV5/EV5A/WT/1 (1).jpg]

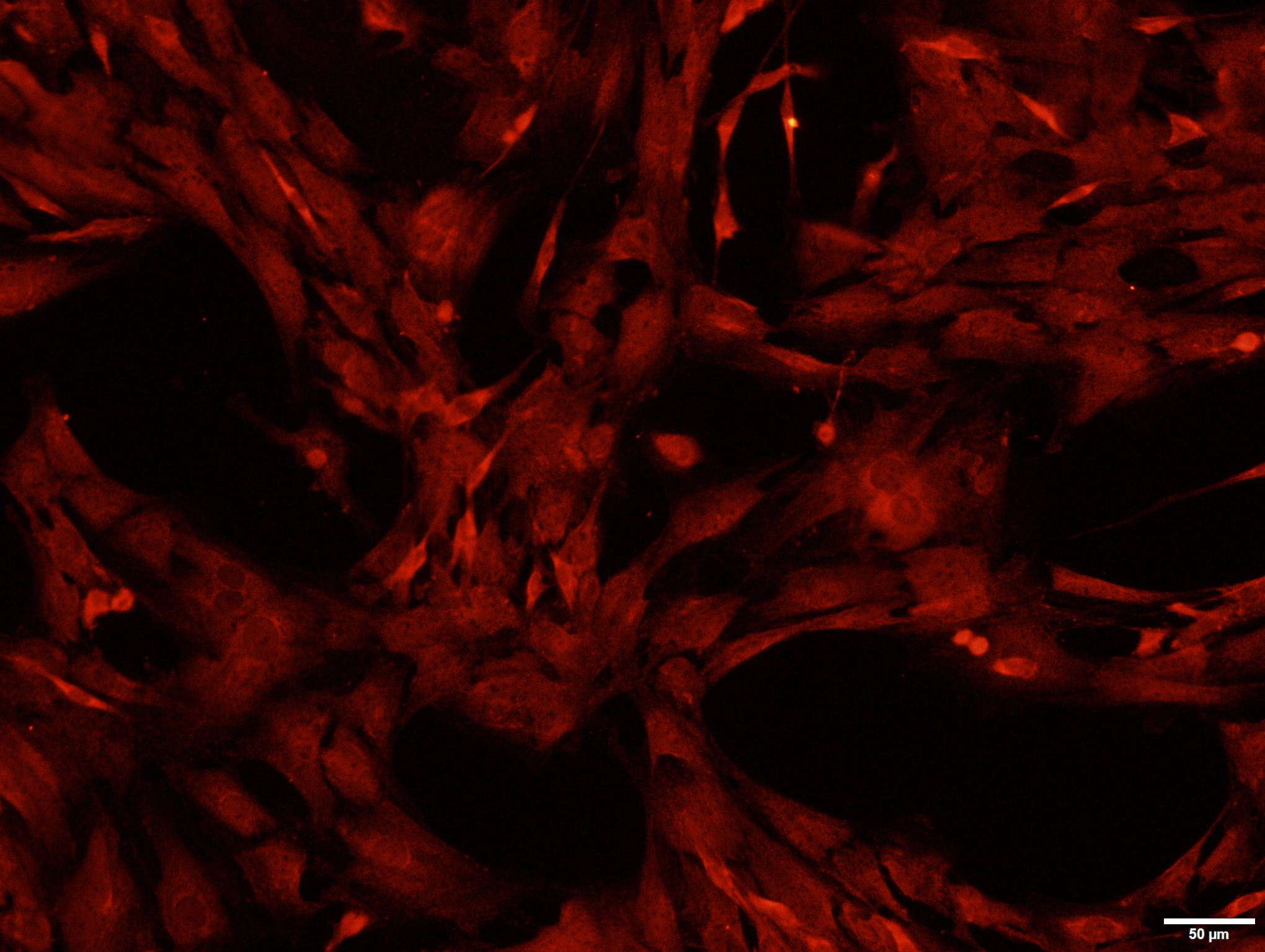

Supplement: Supplementary file 9 — EV Figures Source Data [file 44319_2024_327_MOESM9_ESM.zip › source data-Supplemental Figures/Figure EV5/EV5A/WT/1 (2).jpg]

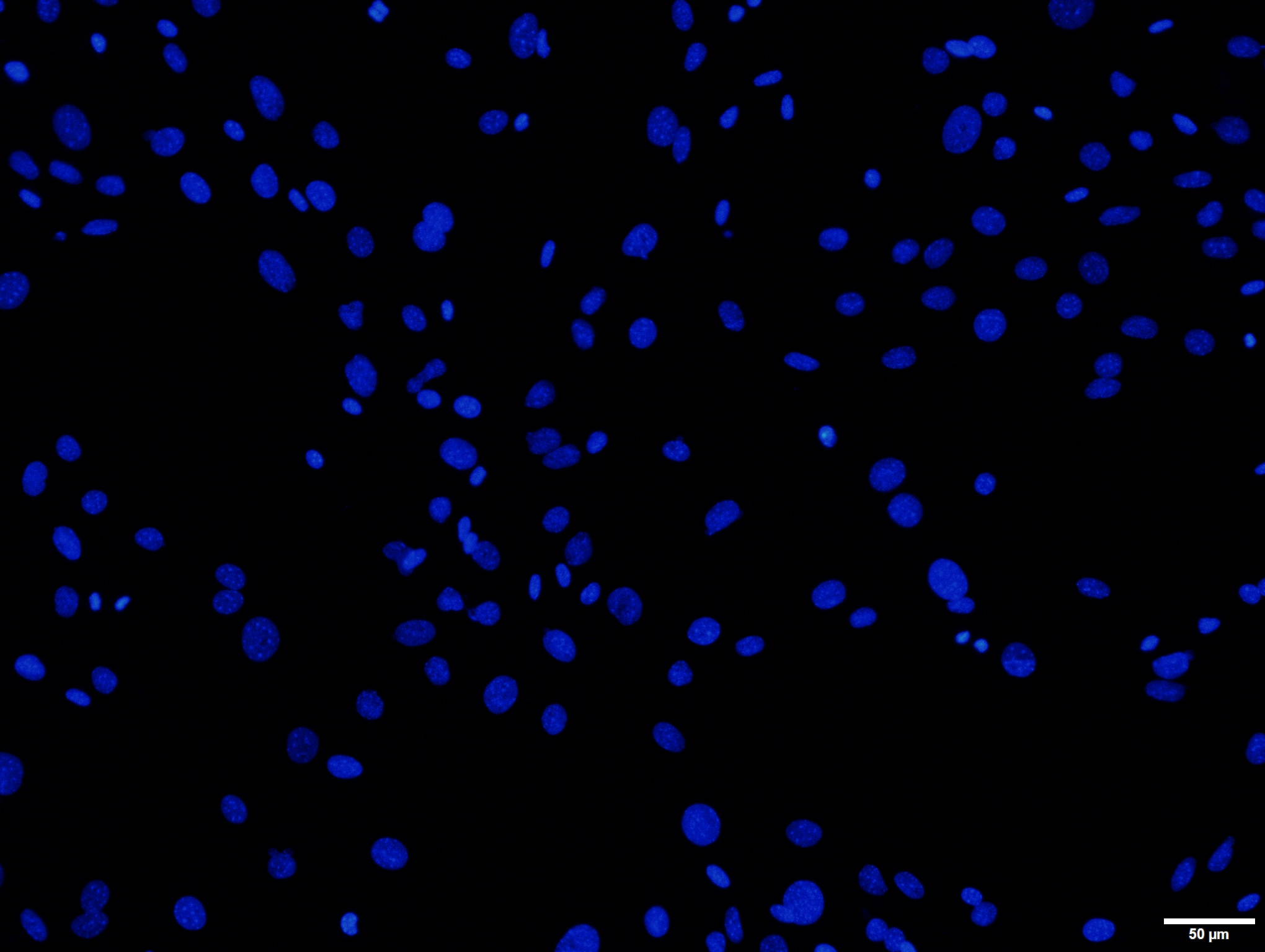

Supplement: Supplementary file 9 — EV Figures Source Data [file 44319_2024_327_MOESM9_ESM.zip › source data-Supplemental Figures/Figure EV5/EV5A/WT/1 (3).jpg]

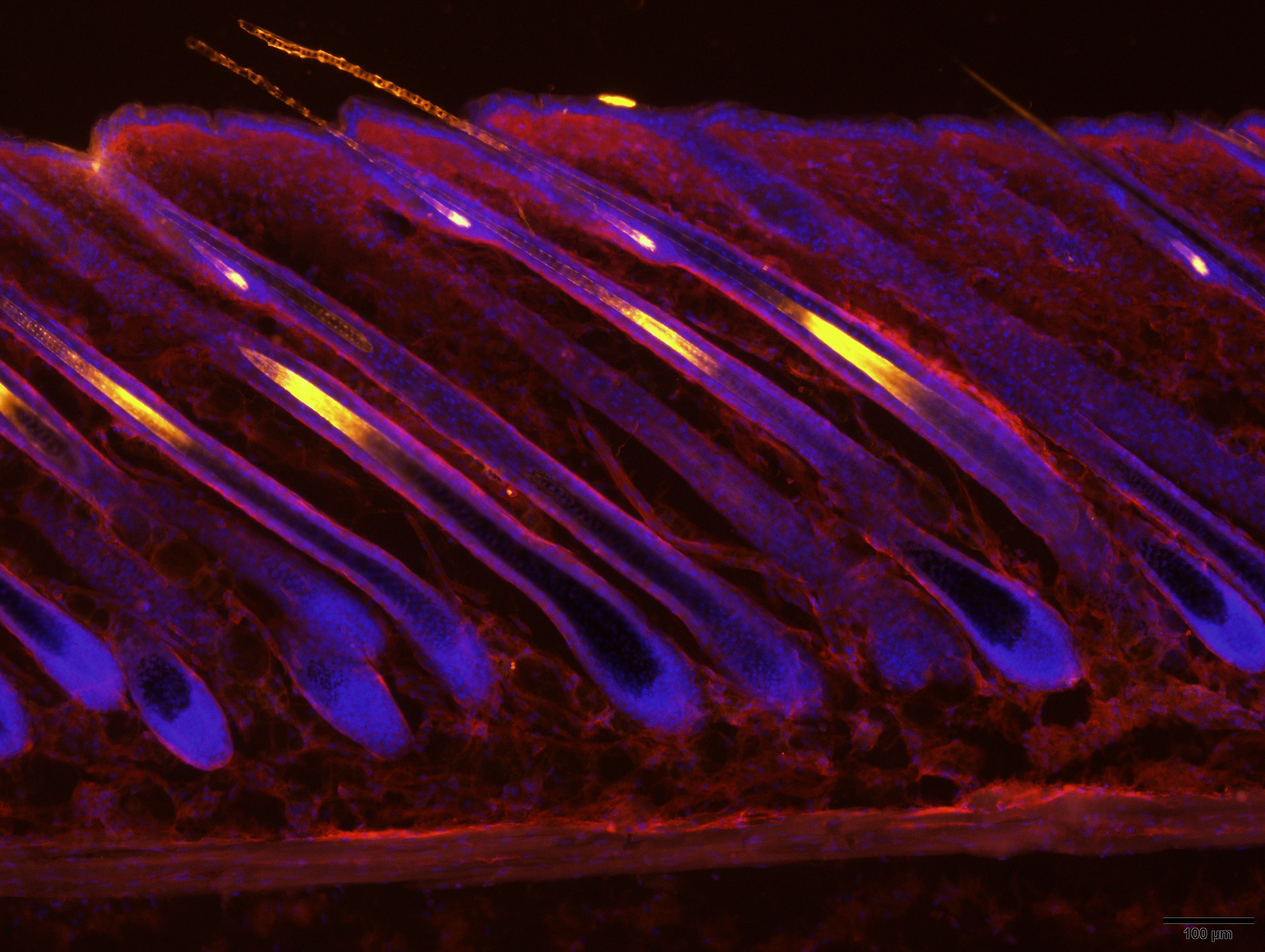

Supplement: Supplementary file 9 — EV Figures Source Data [file 44319_2024_327_MOESM9_ESM.zip › source data-Supplemental Figures/Figure EV5/EV5C/KO/1 (1).jpg]

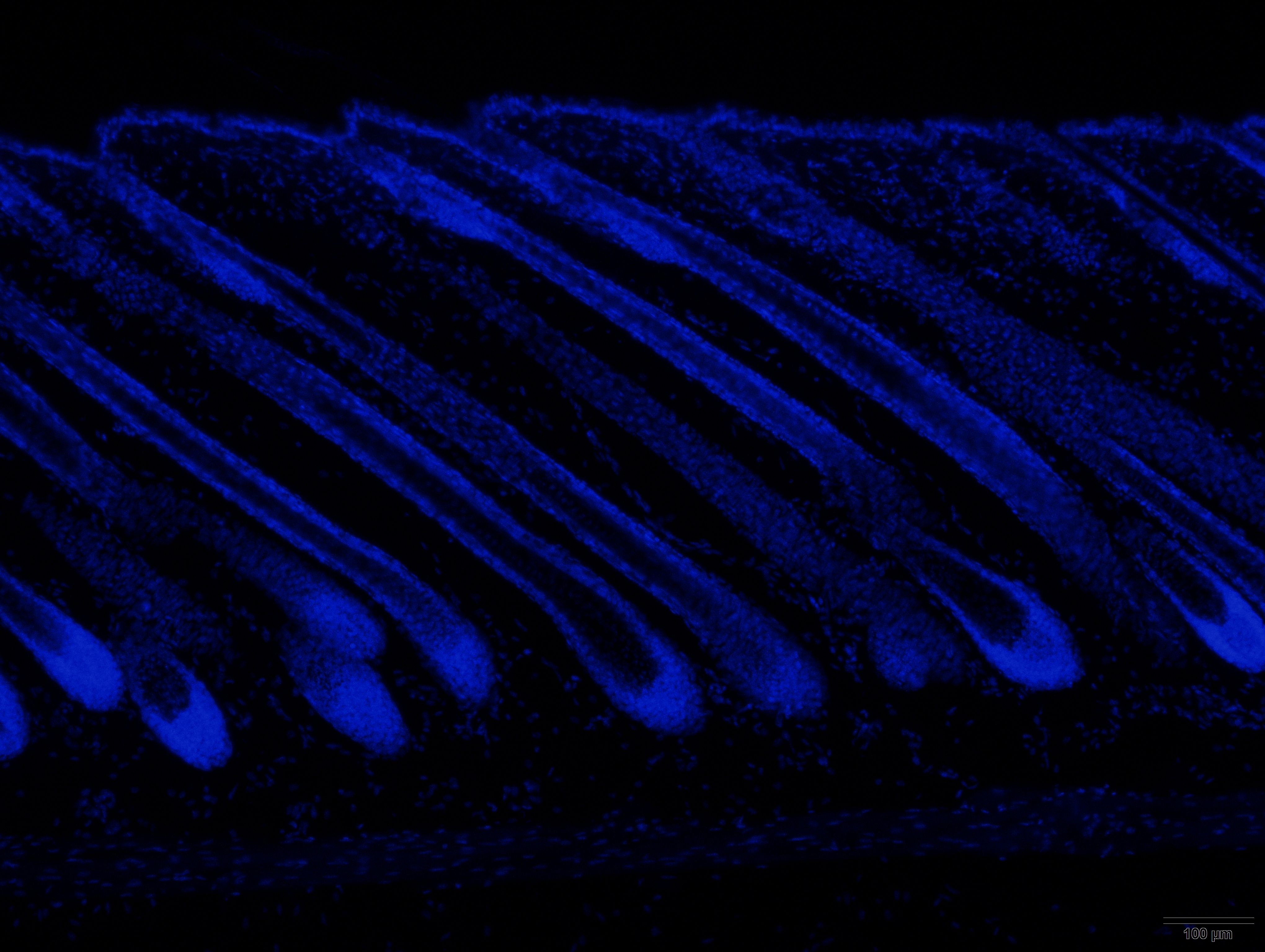

Supplement: Supplementary file 9 — EV Figures Source Data [file 44319_2024_327_MOESM9_ESM.zip › source data-Supplemental Figures/Figure EV5/EV5C/KO/1 (2).jpg]

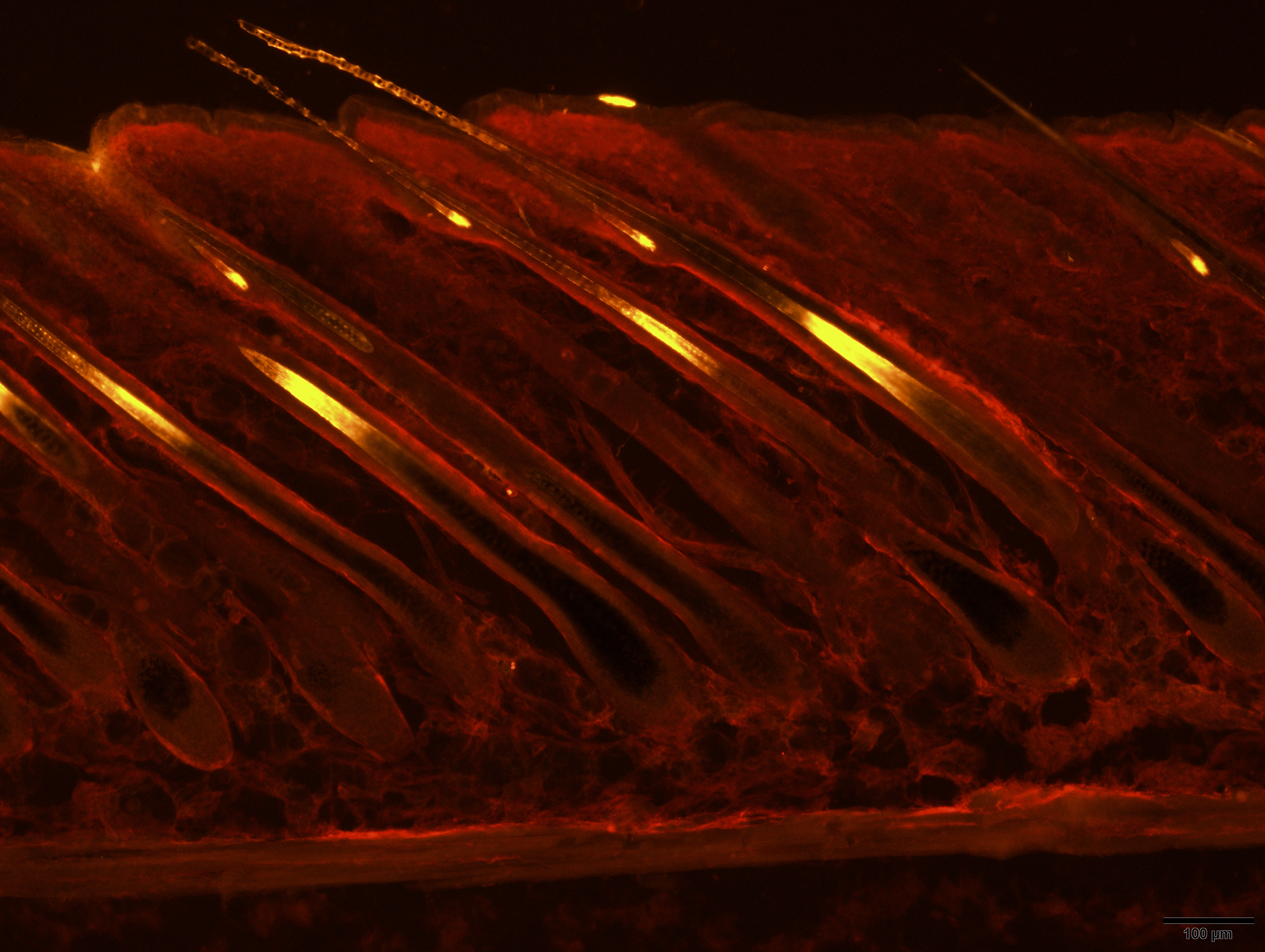

Supplement: Supplementary file 9 — EV Figures Source Data [file 44319_2024_327_MOESM9_ESM.zip › source data-Supplemental Figures/Figure EV5/EV5C/KO/1 (3).jpg]

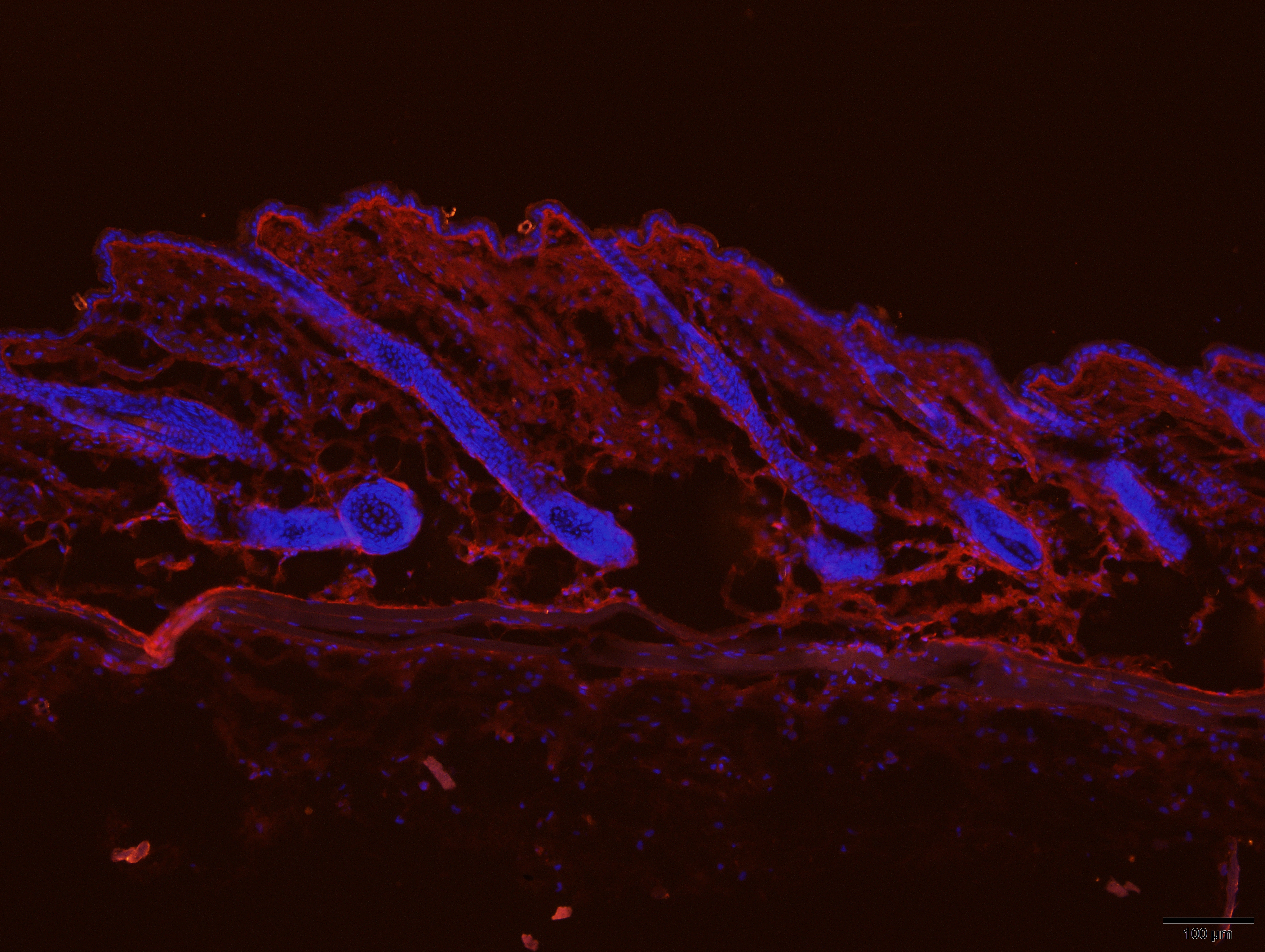

Supplement: Supplementary file 9 — EV Figures Source Data [file 44319_2024_327_MOESM9_ESM.zip › source data-Supplemental Figures/Figure EV5/EV5C/WT/1 (1).jpg]

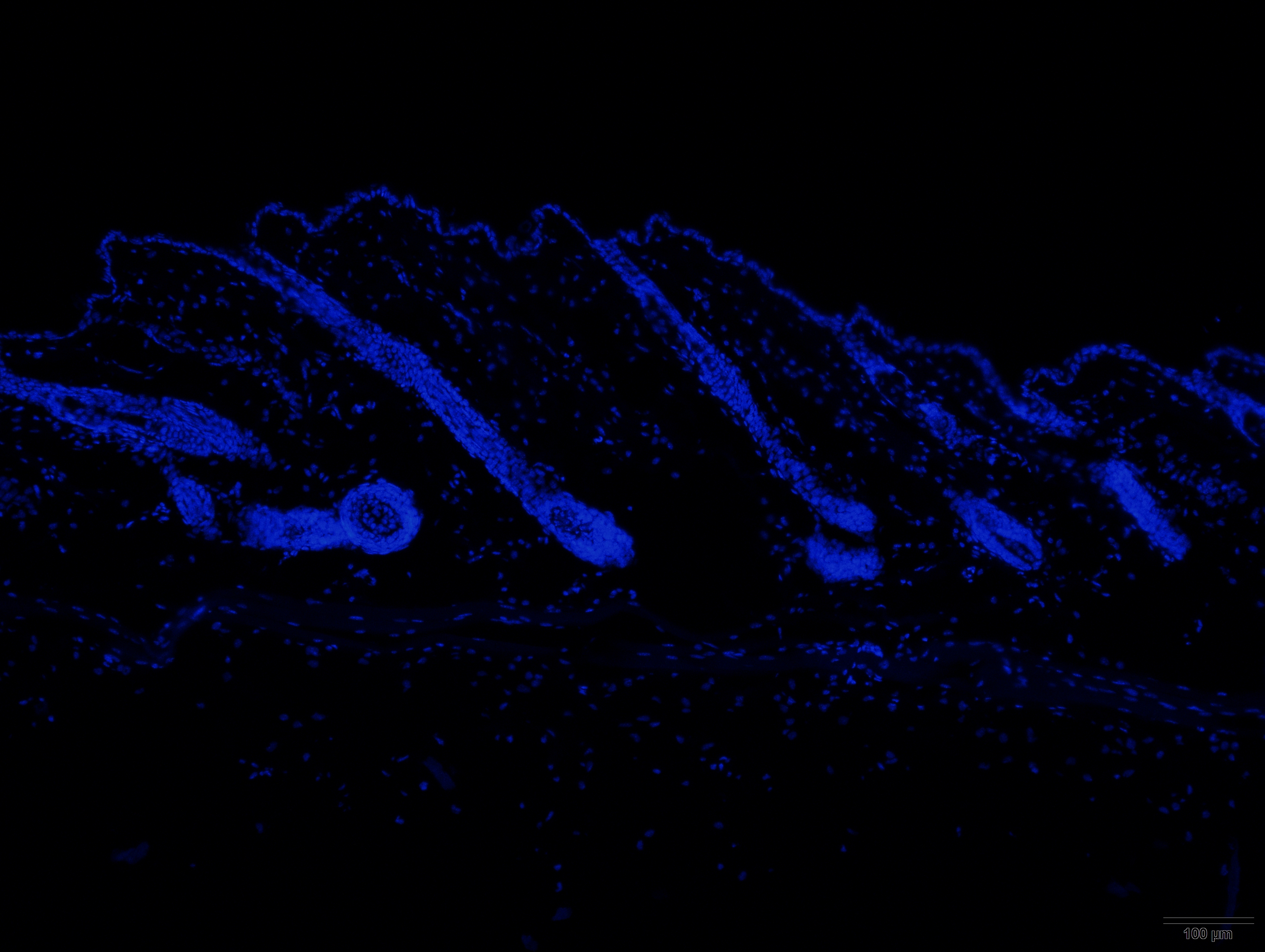

Supplement: Supplementary file 9 — EV Figures Source Data [file 44319_2024_327_MOESM9_ESM.zip › source data-Supplemental Figures/Figure EV5/EV5C/WT/1 (2).jpg]

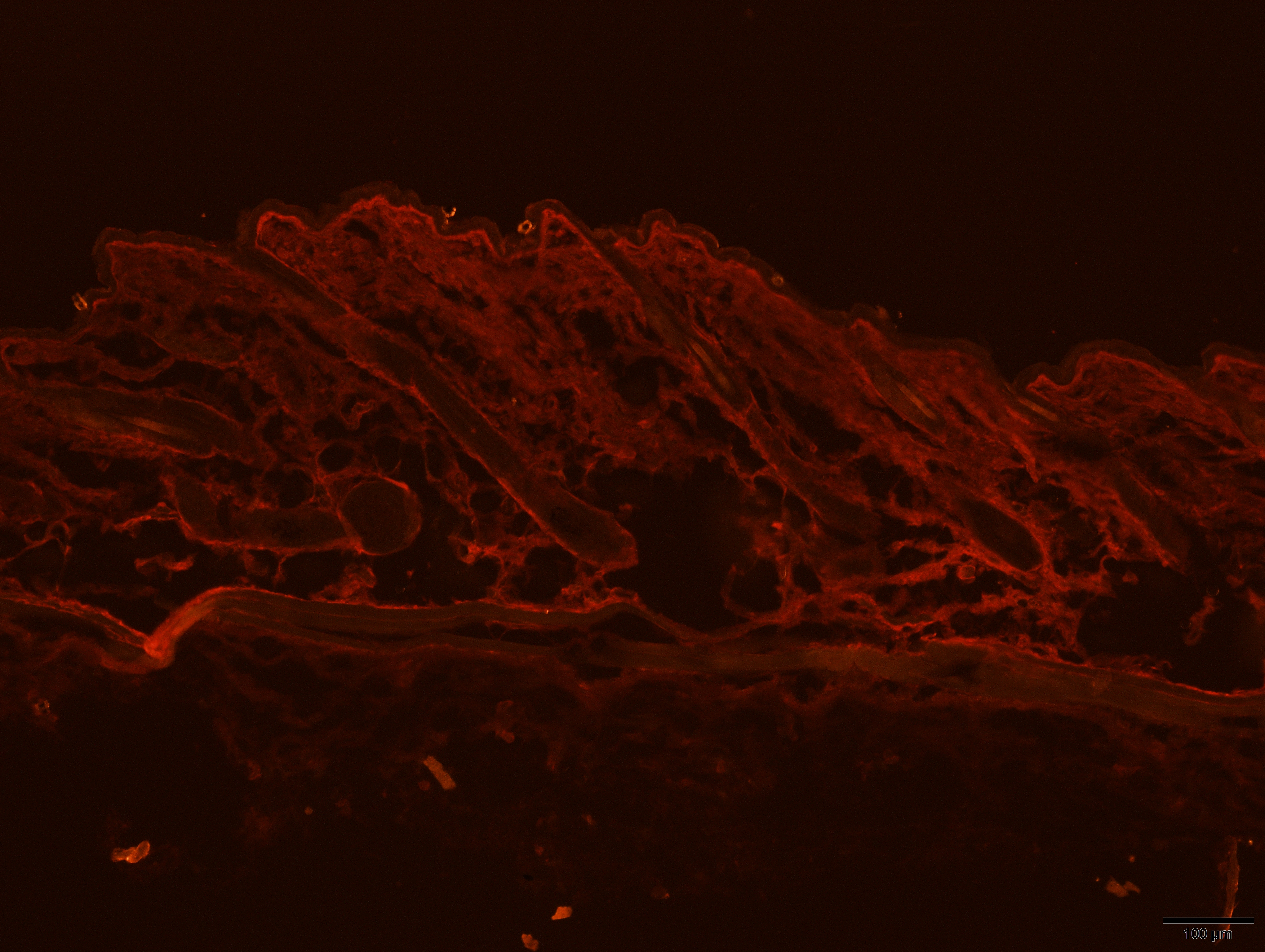

Supplement: Supplementary file 9 — EV Figures Source Data [file 44319_2024_327_MOESM9_ESM.zip › source data-Supplemental Figures/Figure EV5/EV5C/WT/1 (3).jpg]
