# Supplementary material for: Gpr54 deletion accelerates hair cycle and hair regeneration
Source: EMBO Rep. 2024 Nov 25;26(1):200–17. doi: 10.1038/s44319-024-00327-y (PMC11724127; doi:10.1038/s44319-024-00327-y)
Supplement: Supplementary file 10 — Expanded View Figures [file 44319_2024_327_MOESM10_ESM.pdf]

Expanded View Figures

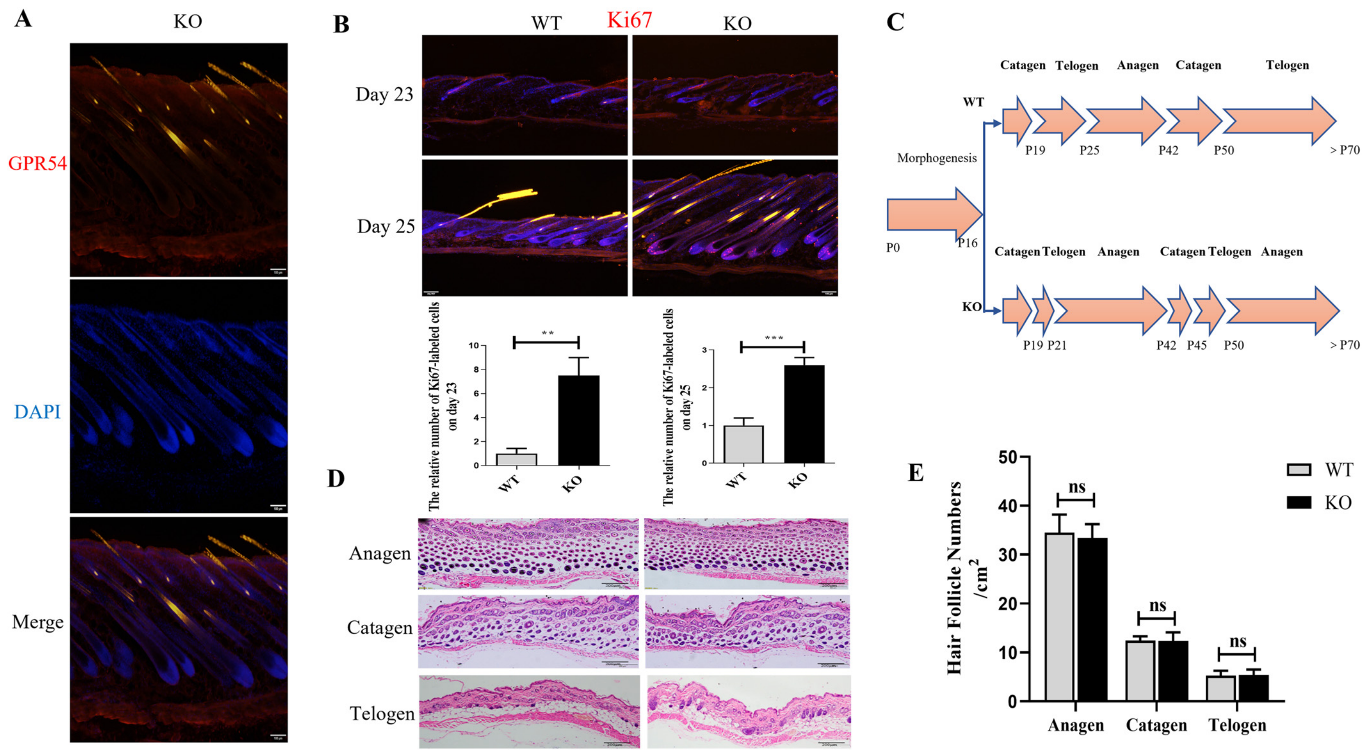

Figure EV1. *Gpr54* knockout accelerated the transition from telogen to anagen of mouse hair follicles.

(**A**) GPR54 expression was absent in *Gpr54* KO mouse hair follicles. The representative images of immunofluorescent staining for GPR54. Scale bar = 100 µm. (**B**) Cell proliferation in *Gpr54* WT and KO mouse hair follicles at day 23 and 25 after birth by using Ki67 immunofluorescent staining. Scale bar = 100 µm. Below the fluorescence images, the bar graphs show the quantitative analysis of the number of Ki67-positive cells in hair follicles of *Gpr54* WT and KO mice on day 23 and 25, respectively. Data are represented as mean ± SD of three independent experiments. A paired *t*-test was performed, $N = 3$. ***P* (P23) = 0.0020, ****P* (P25) = 0.0006, WT versus KO. (**C**) The flowchart dynamically displays the changes in the anagen, catagen, and telogen phases of the hair cycle in *Gpr54* WT and KO mice. (**D**) *Gpr54* knockout did not affect the number of hair follicles in mice. HE staining of hair follicle morphology in anagen, catagen, and telogen phases in *Gpr54* WT and KO mice. (**E**) A quantitative analysis for Fig. EV1D, comparing the number of hair follicles in the anagen phase between *Gpr54* WT and KO mice. The data are representative of three independent experiments (mean ± SD). A paired *t*-test was performed, $N = 3$. Ns indicates no significant difference. Source data are available online for this figure.

© The Author(s)

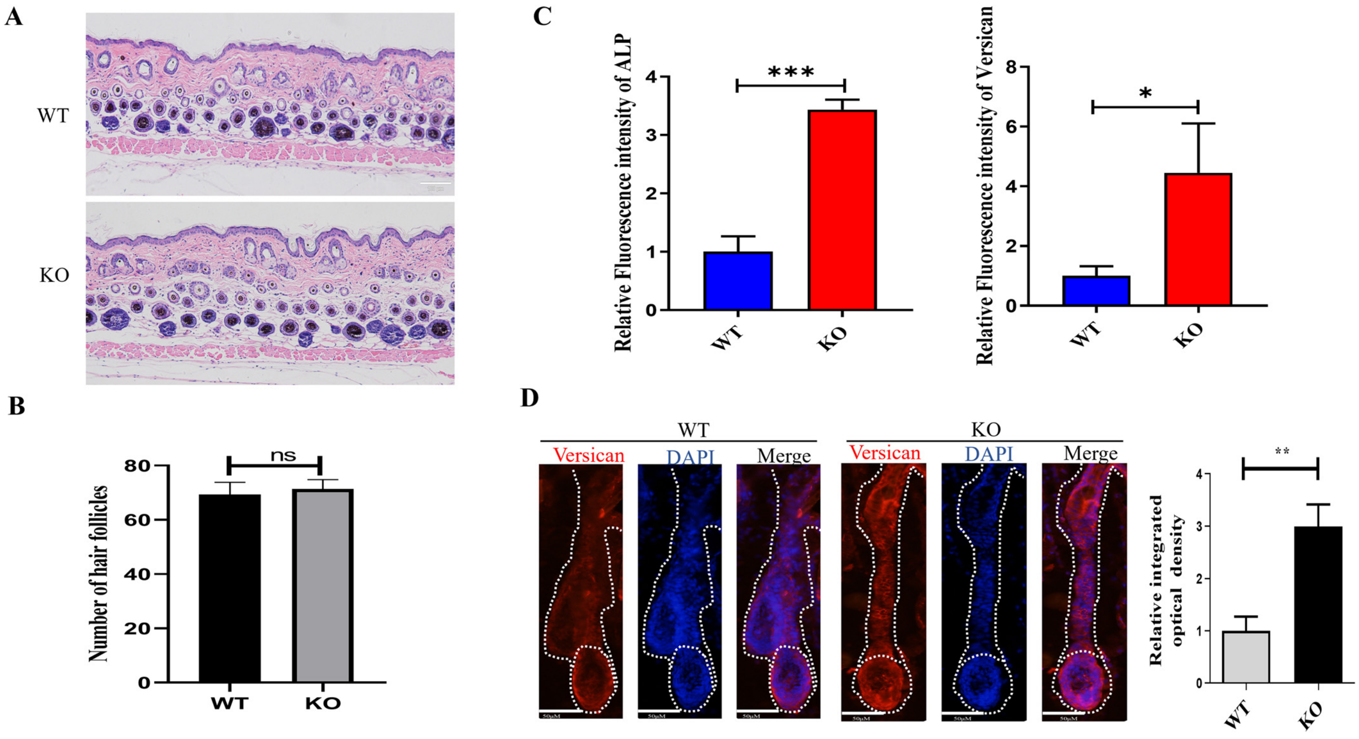

Figure EV2. ***Gpr54*** **knockout enhanced the activity of DPCs.**

(**A**) *Gpr54* knockout did not affect the number of hair follicles in transplants placed in BABL/C nude mice. HE staining of hair follicle morphology in transplants from *Gpr54* WT and KO mice. Scale bar = 100 μm. (**B**) A quantitative analysis for Fig. EV2A, comparing the number of hair follicles in transplants between *Gpr54* WT and KO mice. Data are expressed as mean ± SD of three independent experiments. A paired *t*-test was performed, *N* = 3. Ns indicates no significant difference. (**C**) The quantitative analysis for fluorescent intensity of ALP and Versican in Fig. 3B, C. Data are mean ± SD of three independent experiments. A paired *t*-test was performed, *N* = 3. ****P*(ALP) = 0.0002, **P*(Versican) = 0.0240, WT versus KO. (**D**) Immunofluorescence labeling of Versican in the hair follicles of *Gpr54* WT and KO mice at day 25 after birth. Scale bar = 50 μm. The bar chart on the right presents a quantitative analysis of the immunofluorescent staining of Versican. Data are mean ± SD of three independent experiments. A paired *t*-test was performed, with *N* = 3. ***P* = 0.0024, WT versus KO. Source data are available online for this figure.

© The Author(s)

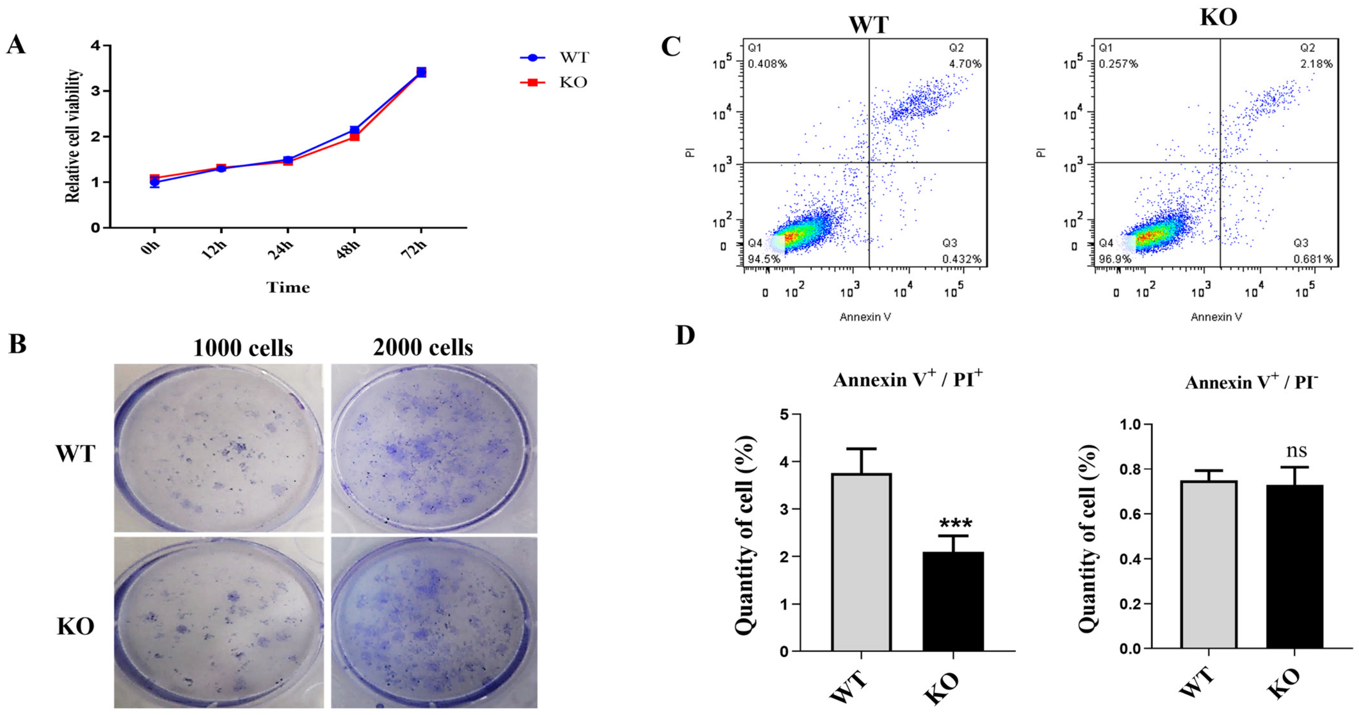

Figure EV3. ***Gpr54* knockout unchanged DPC cell number but reduced their late-stage apoptosis.**

(**A**) Cell proliferation in DPCs of *Gpr54* WT and KO mice assessed using the MTS assay. (**B**) The representative images of single-cell clone proliferation, stained with crystal violet. (**C**) Flow cytometry analysis with Annexin V-PI staining was performed to evaluate the percentage of apoptotic cells in DPCs of *Gpr54* WT and KO mice. (**D**) Quantitative analysis of the proportion of Annexin V^+ and PI^+ apoptotic cells among DPCs of *Gpr54* WT and KO mice. Data are representative of three independent experiments (mean ± SD). A paired *t*-test was performed, $N = 5$. ***$P = 0.0003$, WT versus KO. Source data are available online for this figure.

 © The Author(s)

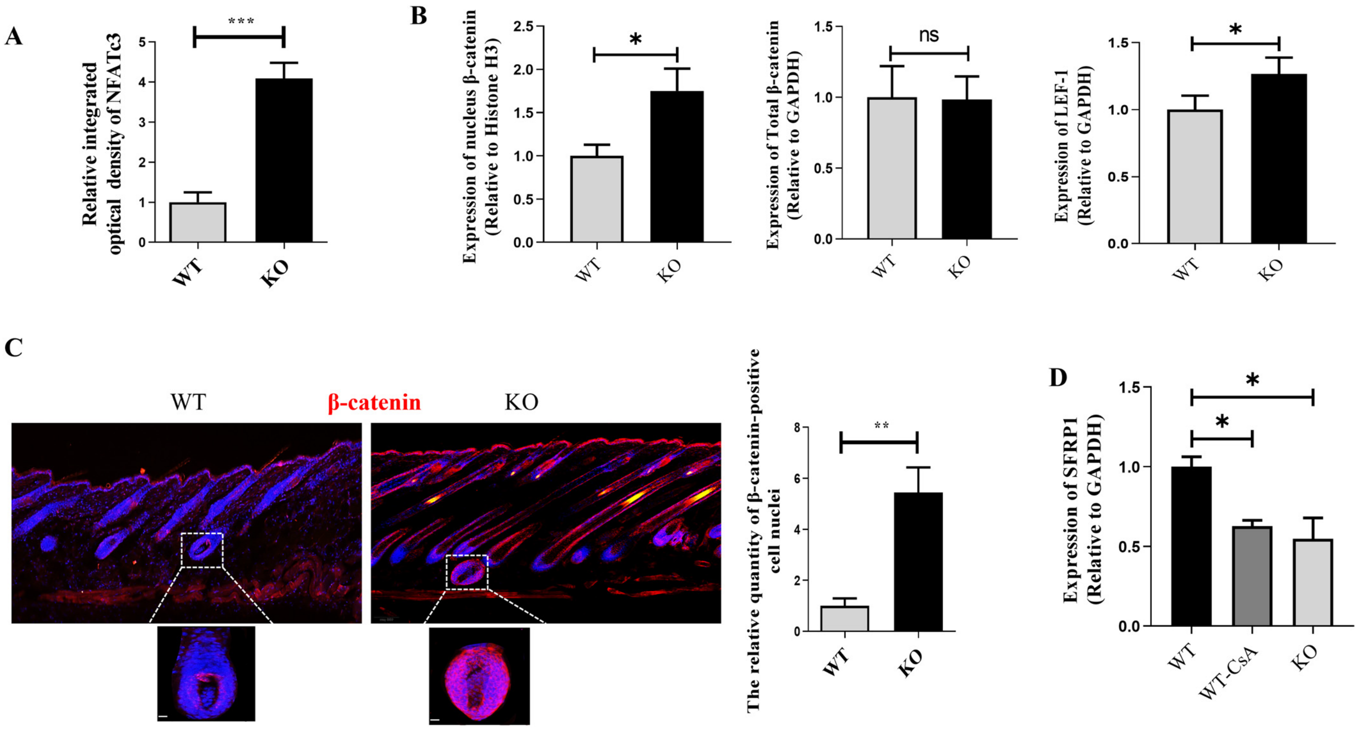

Figure EV4. ***Gpr54*** **deletion increased the activity of β-catenin in hair follicle cells.**

(**A**) A quantitative analysis for the immunofluorescent staining of NFATc3 in Fig. 4B. Data are mean ± SD of three independent experiments. A paired t-test was performed, $N = 3$. ***$P = 0.0003$, WT versus KO. (**B**) A quantitative analysis for the WB results of Fig. 5B. Data are mean ± SD of three independent experiments. A paired t-test was performed, $N = 3$. *P(β-catenin) = 0.0108, *P(LEF1) = 0.0444, WT versus KO. Ns indicates no significant difference. (**C**) Immunofluorescence staining of β-catenin in the hair follicles of *Gpr54* WT and KO mice at day 25 after birth. Scale bar = 100 μm. The bar graph on the right shows the quantitative analysis of the number of β-catenin-positive cells in the nucleus. Data are mean ± SD of three independent experiments. A paired t-test was performed, $N = 3$. **$P = 0.0017$, WT versus KO. (**D**) A quantitative analysis for the WB results of Fig. 5D. Data are mean ± SD of three independent experiments. A paired t-test was performed, $N = 3$, *P (WT+CsA vs. WT) = 0.0179, *P (WT vs. KO) = 0.0474. Source data are available online for this figure.

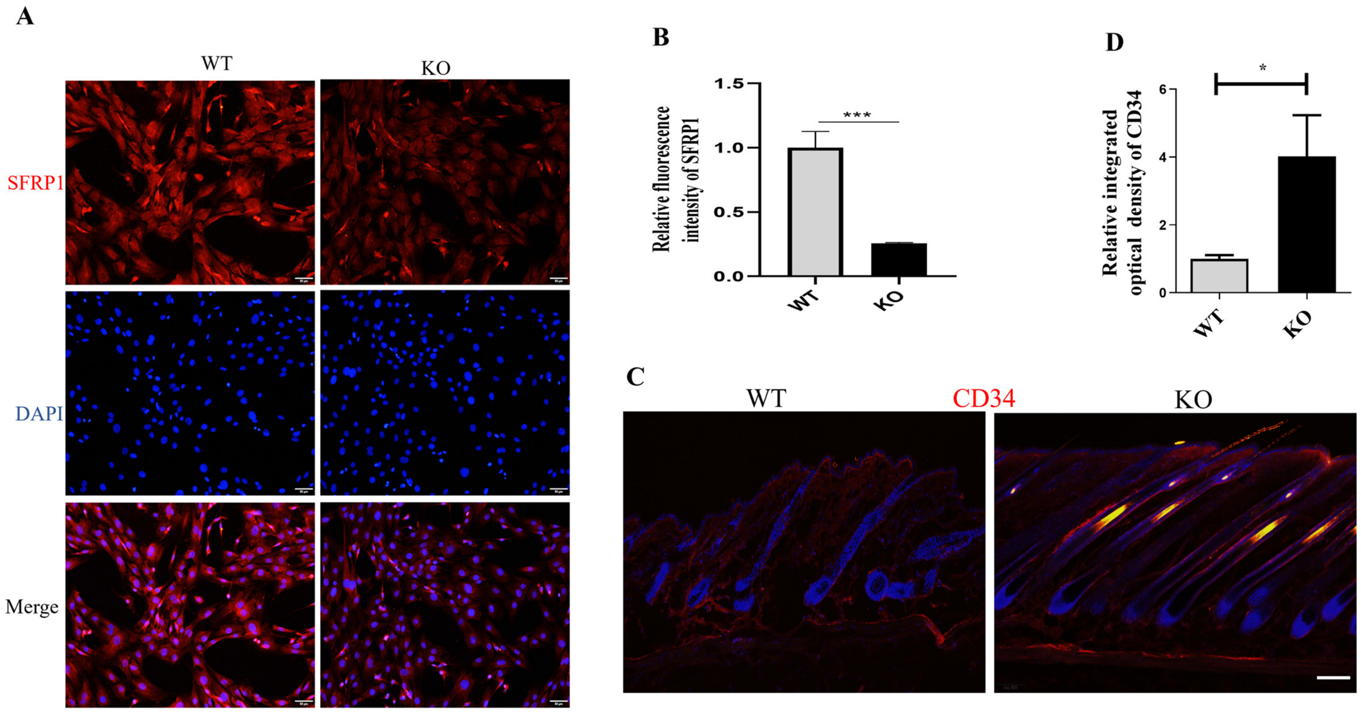

Figure EV5. ***Gpr54*** **deletion decreased SFRP1 in DPCs but increased CD34 in hair follicles.**

(**A**) The representative images of the immunofluorescence staining of SFRP1 in cultured *Gpr54* WT and KO DPCs. Scale bar = 50 µm. (**B**) The bar graph on the right represents the quantitative analysis of SFRP1 expression. Data are mean ± SD of three independent experiments. A paired *t*-test was performed, $N = 3$. ***$P = 0.0005$, WT versus KO. (**C**) *Gpr54* deletion increased the level of CD34 in hair follicle stem cells. The representative images of immunofluorescence staining of CD34 in the hair follicles of *Gpr54* WT and KO mice at day 25 after birth. Scale bar = 100 µm. (**D**) The bar graph represents the quantitative analysis for CD34 immunostaining in Fig. EV5C. Data are mean ± SD of three independent experiments. A paired *t*-test was performed, $N = 3$. *$P = 0.0128$, WT versus KO. Source data are available online for this figure.

 © The Author(s)
